# Design, construction, and validation of an in-situ groundwater trace element analyzer with applications in carbon storage

**DOI:** 10.1038/s41598-023-32788-x

**Published:** 2023-05-09

**Authors:** Daniel A. Hartzler, Chet R. Bhatt, Dustin L. McIntyre

**Affiliations:** 1 National Energy Technology Laboratory, 3610 Collins Ferry Road, Morgantown, WV 26507 USA; 2 NETL Support Contractor, 3610 Collins Ferry Road, Morgantown, WV 26507 USA; 3 Leidos Research Support Team, 3610 Collins Ferry Rd, Morgantown, WV 26505 USA

**Keywords:** Carbon capture and storage, Optical spectroscopy, Optical sensors, Environmental monitoring

## Abstract

It is estimated that carbon emissions should reach net-zero by 2050 to meet important climate targets. Carbon capture is likely necessary to reach these targets, requiring a long-term storage solution such as geological carbon sequestration. However, as with any subsurface activity, leakage can occur, potentially impacting groundwater quality near the storage site. Rapid detection is essential to mitigate damage to this resource. Since CO_2_ will acidify groundwater, the concentrations of acid soluble minerals and associated cations will increase. Thus, an in-situ, real-time element analysis system based on laser-induced breakdown spectroscopy (LIBS) is under development to monitor these elements. The system splits the traditional LIBS system into a miniature, all-optical sensor head built around a passively Q-switch laser fiber coupled to a control unit. Previous work has validated the LIBS technique for use at high pressure as well as the split system design. In this work, a fieldable prototype sensor is developed and tested in an onsite monitoring well where trace elements concentrations (approx. 0–3 ppm) were tracked over 20 days. These concentrations varied in response to local rainfall, diluting with increased rain, demonstrating the ability of a LIBS-based sensor to track trace elements under real-world conditions.

## Introduction

While United States CO_2_ emissions have dropped from 5.5 billion metric tons (GT) in 2010 to 5.0 GT in 2019 (decrease of ~ 0.05 GT/year), global emissions rose from 31 to 34 GT (increase of ~ 0.3 GT/year) over the same period^1^. In order to meet important climate targets outlined by the IPCC (Intergovernmental Panel on Climate Change), it is estimated that CO_2_ emissions need to reach net-zero around 2050, requiring an average decrease of ~ 1.2 GT/year^2^. Reaching net-zero will likely require carbon capture and storage (CCS) technologies^3^, either capturing CO_2_ at industrial point sources like fossil energy production facilities or direct air capture to offset non-point sources like aviation and shipping. While some use can be made of the captured CO_2_^4^, dealing with the volume of captured gas will likely require an approach like geologic carbon sequestration (GCS)^3^, where CO_2_ is injected into the subsurface at depths exceeding 2500 ft (~ 800 m), below which it exists as a supercritical fluid^5^. Candidate storage formations include saline aquifers such as the Mt. Simon sandstone formation underlying parts of the midwestern United States, which has an estimated storage potential between 24 and 355 GT of CO_2_^5^.

Loss of containment at a GCS site can cause subsurface brines or CO_2_ to migrate upward through the strata into aquifers and soils, potentially contaminating groundwater, a critical resource that millions in the United States rely on for drinking or economically for agriculture and industry. In 2015, an average of over 82 billion gallons (Bgal) (311 Gigalitre (GL)) per day of fresh groundwater were withdrawn in the US, the majority going to agriculture (70% irrigation and 3.5% livestock and aquaculture) and municipal/domestic supply (19% public supply and 3.9% self-supplied domestic)^6^. Unfortunately, groundwater contamination is already relatively common. The United States Geological Survey (USGS) National Water-Quality Assessment (NAWQA) Program found that up to 22% of sampled public-supply wells and 23% of self-supply (i.e., privately owned) domestic wells contained at least “one contaminant at levels of potential health concern”^7^. If leaking CO_2_ or subsurface brines reach these aquifers it is expected to contaminate the groundwater, increasing total dissolved solids (TDS) and, potentially, trace toxic metals^8–11^.

Fortunately, leakage can be detected by monitoring groundwater composition around GCS sites. Since subsurface brines can contain significant levels of dissolved minerals (e.g., TDS of Mt Simon brine can be over 250,000 ppm^12^) and CO_2_ induced acidification can dissolve formation minerals, contamination of aquifers from these sources can be detected as a sudden spike or other unexplained increase of cations associated with the brine or rock formations. For example, studies of CO_2_ released into the subsurface (both artificial and natural sources), have demonstrated significant increases in cation concentration over baseline levels, with Ca increasing by 10’s to 100’s of ppm and K and Mn increasing by 10’s to 1000’s of ppb^9–11,13^.

A wide variety of analysis techniques are used to measure trace elements in groundwater, each with advantages and disadvantages depending on the desired information, sample type, and operating environment. Two commonly used techniques for water trace element analysis are inductively coupled plasma mass spectrometry (ICP-MS) and ICP atomic emission spectroscopy (ICP-AES)^14^. Both techniques are highly sensitive, multi-element techniques with large dynamic ranges^15,16^. Disadvantages of ICP-MS / AES include high equipment cost, operator training requirements, limits to TDS for ICP-MS, and a lack of portability, requiring samples to be collected in the field and transported to a laboratory for analysis^15,16^. Depending on the type of analytical lab used (i.e., in-house or 3^rd^ party), results can take hours to weeks before being returned. Furthermore, water sample quality can degrade during collection and transport due to temperature and pressure-induced chemical changes, such as outgassing and mineral precipitation^17–20^.

X-ray fluorescence (XRF) is another technique that can be used for trace element analysis of water^21,22^. Advantages of this technique include availability, portability, and ease of use with handheld commercial off the shelf units readily available. While XRF has shown detection limits similar to LIBS for select heavy elements, the technique struggles to measure so called “light” elements with atomic numbers (Z) around ~ 14–19 and no sensitivity for elements below Z ≈ 12–13 without specialized equipment ^23–25^. An illustrative example can be found in reports by Bhatt et. al.^26^ and Johnson^23^. Bhatt et al. demonstrated (single pulse) LIBS detection limits of 2, 29, and 16 ppm for As (Z = 33), Se (Z = 34), and Hg (Z = 80) respectively, while Johnson demonstrated XRF detection limits of 4, 3, and 6 ppm for As, Se, and Hg respectively. By comparison, detection limits for S (Z = 16) were determined to be 16 ppm with LIBS and 1,750 ppm with XRF^23,26^. Note that the exact values of ‘Z’ were sensitivity issues arise with XRF are dependent on the instrument used, sample properties (e.g., density), etc. Additionally, XRF poses safety concerns due to the use of ionizing radiation.

Laser-induced breakdown spectroscopy (LIBS) is a rapidly developing atomic emission technique for elemental analysis. In LIBS, an intense pulsed laser is focused into or onto a sample, breaking down the target material into a plasma. As the plasma cools, its emission spectrum is measured, permitting the elements present to be identified and quantified based on the wavelength and intensity of their characteristic emission lines^27–29^. While not as sensitive as ICP-MS/AES or as well developed as XRF and the aforementioned techniques, major advantages of LIBS include (1) little to no sample preparation needed, (2) sensitivity to both light and heavy elements, and (3) potential for miniaturization and ruggedization for continuous, in-situ measurements in hostile environments.

The project aims to develop an in-situ, subsurface probe for high-frequency/real-time elemental analysis. This study describes work to design, construct, and test a fieldable prototype system. Lessons learned from this prototype will be applied to the next design iteration to improve ease of use and reliability.

While the intended purpose of this system is to monitor GCS sites for leakage, what has been developed is a general-purpose elemental analyzer for hostile environments that could be applied as an on-line sensor in applications that traditionally use off-line elemental analysis or could benefit from the new capability. Potential application areas include real-time industrial process monitoring and control, environmental field monitoring, and saltwater intrusion detection and early-warning among many other possibilities^30–32^.

### Prior work

Underwater LIBS (i.e., laser spark submerged in the bulk fluid) has been used to investigate aqueous solutions for several decades and has even been deployed on underwater remotely operated vehicles (ROVs) to the ocean floor at depths of up to 1400 m to investigate hydrothermal vent fluids^33–44^. While the ROV mounted systems can successfully operate in harsh, high-pressure environments, the equipment is large, heavy, and must have significant support (e.g., electrical power, temperature regulation, etc.) at the point of measurement. For instance, the ChemiCam system^43^ is enclosed in a 300 × 1300 mm (11.8 × 51″) cylindrical vessel and draws 140 W of electrical power, while the LIBSea II system^44^ enclosure is 190 × 588 mm (7.5 × 23″).

While the environment near deep ocean hydrothermal vents bares some similarities to the subsurface environment in terms of pressure and dissolved salts, minerals, and gasses^45–48^, sensor size is a major constraint for subsurface use. For example, downhole logging tools used in the oil industry are typically cylindrical and designed to operate in boreholes with minimum internal diameters of around 2–6″ (~ 5–15 cm)^49^. Furthermore, borehole diameter is an important consideration for monitoring well installation cost, thus, smaller instrumentation is preferred.

With GCS monitoring wells, one can expect a complex matrix containing significant amounts of dissolved CO_2_, salts, and other minerals and gasses at a pressure of hundreds of atmospheres/bar (1 atm ≈ 1 bar). It has been demonstrated that both the pressure and matrix effects from dissolved materials can have a significant effect on the strength. How the signal is affected can be complex and depends on several factors such as the analyte of interest and the specific matrix composition.

The influence of pressure on underwater laser induced plasmas and LIBS signals has been extensively studied^33–37,50–56^. When a laser pulse induces a plasma in liquid, a cavitation bubble is created which grows to a maximum size and then collapses. While the external hydrostatic pressure plays a major role in determining the final volume of the cavitation bubble, the (single pulse) LIBS signal is generated in the early stages of the bubble evolution when the internal pressure exerted by the plasma can surpass 1 GPa or 10^4^ bar, far in excess of pressures encountered in the deep ocean (~ 100 bar per 1 km depth)^52–55^. This is demonstrated by several studies showing that a few hundred bar of static pressure does not severely influence the signal strength of many elements (few percent up to a factor of ~ 2), with signal strength either increasing or decreasing with rising pressure, depending on the element^33,37,50,51,56^. It must be noted that how the “signal” is calculated matters as both the emission line peak intensity and line width can vary with pressure resulting in, for example, a peak intensity that decreases with rising pressure but an integrated peak area (which accounts for broadening) that increases with pressure^51^.

In addition to pressure, the chemical properties of dissolved gasses and minerals can also affect analyte emission signals in what is known as a matrix effect. For example, an aqueous solution of CaCl_2_ was pressurized up to 120 bar using either N_2_ or CO_2_, and different responses were observed between the two gasses, with Ca emission peak intensity dropping more rapidly with increasing N_2_ pressure as compared to CO_2_ pressure, potentially due to the significantly higher solubility of CO_2_ in water^56^. Note that only peak intensities, not integrated peak areas, were reported. Additionally, studies have shown that high salt concentration can alter analyte emission line intensity, for example, modifying the relative intensity of atomic and ionic lines or enhance the absolute signal strength, resulting in improved signal-to-noise ratios (SNRs) and decreased the limits of detection (LODs)^37,41,57^. It must be noted that at very high salt concentrations, calibration curves for some elements (e.g., K and Ca in NaCl solution) become nonlinear^57^. These results are particularly important for GCS leak detection, where large amounts of dissolved CO_2_ and salts could be encountered^47,58^.

As mentioned, it is expected that leaking CO_2_ will acidify aquifers it encounters. While the LIBS technique is not directly sensitive to solution pH, recent work has shown that underwater LIBS is capable of detecting dissolution of carbonates by CO_2_-induced acidification^59,60^. These studies demonstrated LIBS detection of Mg, Ca, Sr, and Mn carbonates leached from pellets as well as a sample of Mt. Simon sandstone with increasing CO_2_ pressure. The authors chose this series because each carbonate dissolves at progressively lower pH, thus indicating the solution pH though release of the corresponding metal ions. Carbonate pellets were observed to release Mg, Ca, and Sr at 50 bar CO_2_ and above while Mn was only observed at a pressure of 100 bar and above. A sample of Mt. Simon sandstone was observed to release approximately 25 to 60 ppm Ca as CO_2_ pressure rose from 50 to 250 bar. These studies demonstrate the ability of LIBS to detect mineral dissolution due to CO_2_ acidification of groundwater.

### System concept

**Figure 1:**
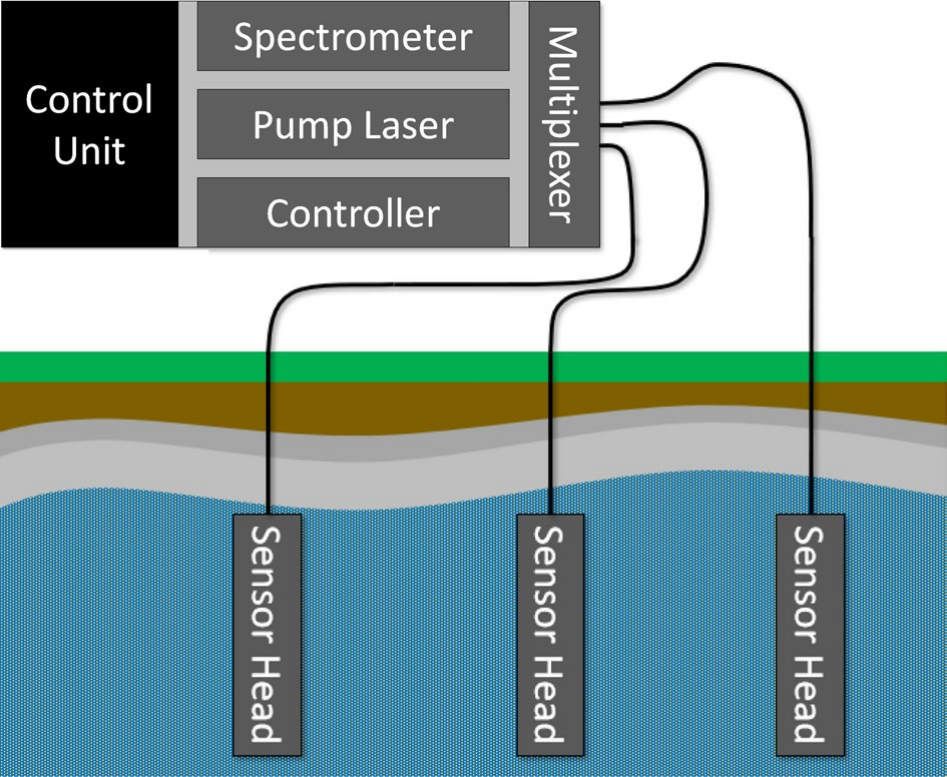
The LIBS system is split into two subsystems: (1) an above ground control unit connected via fiber optics to (2) one or more sensor heads.

The measurement system is split into two subsystems (see Fig. 1) connected by a fiber-optic umbilical; (1) a surface control unit containing the spectrometer, pump laser, and computer and (2) a rugged, miniature, LIBS based sensor head built around a passively Q-switched (PQSW) laser. This fiber-coupled design allows the large and fragile components to remain on the surface while only the low-cost sensor head needs to enter the hostile, downhole environment. Additionally, one control unit could be connected to multiple sensor heads, allowing a wider area to be monitored while reducing the amount of redundant equipment.

### System design

**Figure 2:**
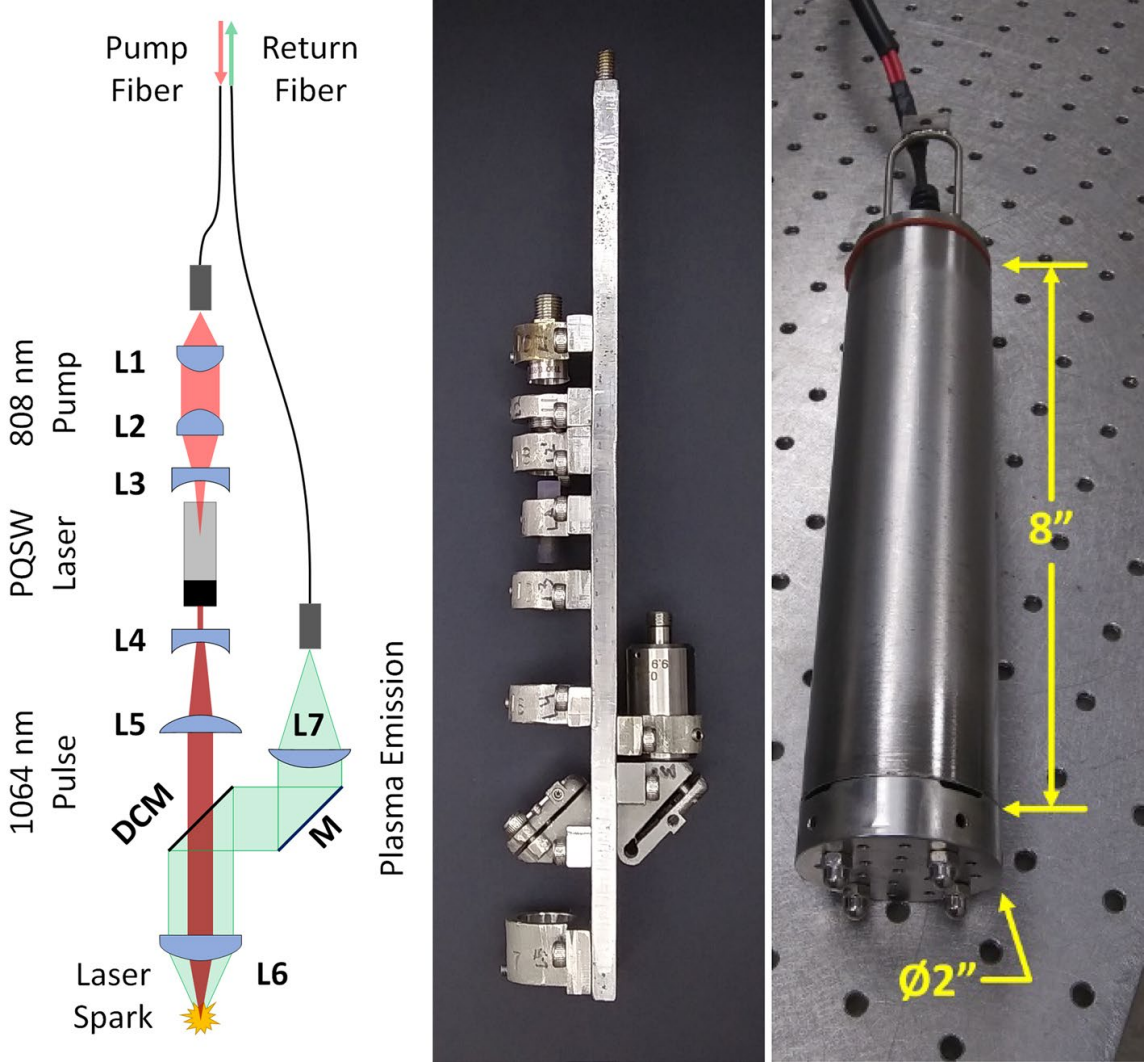
Sensor head. Optical schematic (Left), assembled optical rail (Middle), and assembled sensor head.

A prototype sensor head was built around a custom Nd/Cr:YAG doped PQSW laser producing ~ 4 mJ, 3 ns pulses at 1064 nm, an optical schematic is shown in Fig. 2 (see Hartzler et al.^61^ for a description of the laser). The pump laser is delivered to the probe via a fiber-optic cable (i.e., the “Pump Fiber”) where a telescope (Fig. 2, L1–L3, ThorLabs: A110TM-B and A397TM-B, Edmund Optics: 67–987) couples it into the PQSW laser. The PQSW laser output pulse is expanded 3x (L4 & L5, Edmund Optics: 67–987 and 67–498) before exiting the probe through the final focusing lens (L6, ThorLabs: AL1210-C). Expanding the laser beam reduces the focal point diameter (i.e., beam waist) and improves the laser spark^61^. Plasma emission is collected by the focusing lens (L6) and directed by a long wave pass (LWP) dichroic mirror (DCM, Semrock: FF776-DI01-12X16) to L7 (ThorLabs: F950SMA-A) and a second fiber optic cable (i.e., the “Return Fiber”) for transmission to the surface. Additionally, a small amount of residual, scattered 1064 nm radiation is picked up by the return fiber and used at the surface to trigger the detector. This scattered laser light primarily originates from a small reflection off the backside of the DCM which then strikes the enclosure wall.

It must be noted that the sensor head optics (L7 in particular, an achromatic doublet) strongly attenuate wavelengths below 400 nm. A different choice of optics would partially correct this issue, for instance, using fused silica lenses for L6 and L7 and using a drilled UV enhanced mirror (i.e., a hole drilled through the center) in place of the DCM. Ultimately, UV sensitivity is limited by the fiber optic. Light traveling through the fiber is subjected to Rayleigh scattering^62^, which is proportional to 1/λ^4^, thus, short wavelengths like UV are much more strongly scattered than visible or NIR wavelengths. Since the scattering loss is exponential in the length of the fiber, UV attenuation is particularly severe for long fibers. Finally, additional UV absorption losses can occur due to impurities contained in the glass.

The described optical system was designed to fit into a pressure-resistant, 2″ (51 mm) diameter by 8″ (203 mm) long enclosure (excluding attachment hoop) designed to withstand a ~ 3 bar pressure (Fig. 2), corresponding to a water depth of about 100 ft (~ 30 m). It should be noted that, currently, the design pressure is limited by an IP68 cable gland that allows the fiber optic to passthrough into the enclosure.

**Figure 3:**
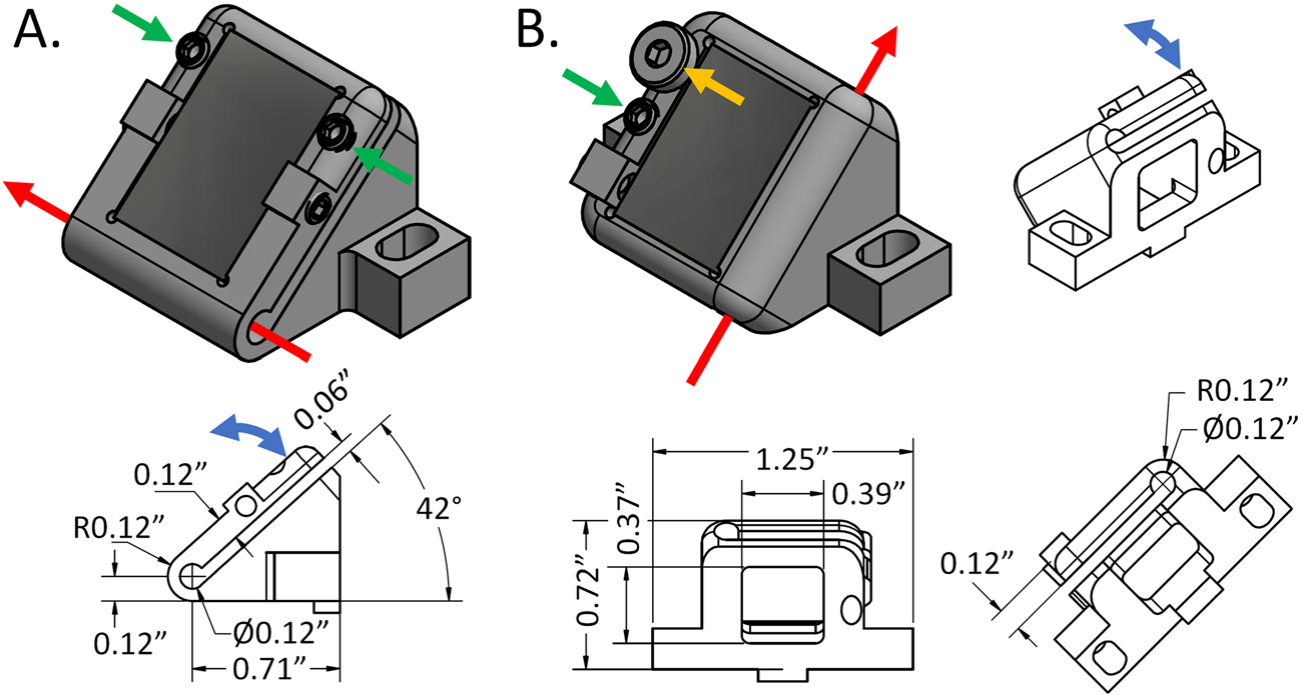
3D printed kinematic mounts. Red arrows indicate the axis of rotation while the blue arrows indicate the direction of motion of the mounted optic. Green and Yellow arrows indicate adjustment screw locations, set and machine screws respectively. (**A**) Aluminum mirror mount, “M” and (**B**) DCM mount (see Fig. 2).

Optics were mounted on a custom rail (Fig. 2, middle) in mounts which can each translate a few millimeters along the rail to facilitate alignment. Most of the prototype’s mechanics were made using traditional machining techniques. However, due to their small size and complexity, the mounts for the DCM and aluminum mirror (“M”) were 3D printed (i.e., made using additive manufacturing) to include a flexure hinge allowing for either a tip or tilt angular adjustment (see Fig. 3). The Initial adjustment angle for mount “M” (Fig. 3A) was set to less than 45° to permit a single “push” adjustment provided by a set screw to access angles both less than and greater than 45°. The DCM mount (Fig. 3B) was manufactured at an initial adjustment angle of 0° and used a “push/pull” mechanism to access angles greater than and less than 0°. The pull adjustment was provided by a machine screw passing through the movable portion of the mount and threaded into the immobile portion, bending the flexure inward when tightened. Push adjustments are provided by set screws threaded into the movable portion that push against the immobile portion, bending the flexures outward. Note that all metallic components were fabricated with 316 stainless steel.

The enclosure window consisted of a fused silica “beam sampler” (ThorLabs: BSF05-C), an optical flat uncoated on one side and antireflective (AR) coated on the other. The window was epoxied into the enclosure with the AR coating facing the interior. When immersed in water, this window provides a low back reflection of the laser from the AR coating on the inside (< 1.0% reflection) and small Fresnel reflection at the exterior glass-water interface (~ 0.2% reflection). This back reflection can damage lens L6, so care must be taken to minimize it.

A prototype surface control unit was constructed and enclosed within a 2’ × 3’ × 4’ (~ 60 × 90 × 120 cm) insulated, climate-controlled box. The control unit consisted of an 808 nm, fiber-coupled diode laser (Apollo Instruments: F700-808-6) and driver (Northrup Grumman: eDrive), a fast-gated Czerny-Turner spectrograph (Andor: Shamrock 303i with an iStar ICCD DH320T-25F-03), computer, and chiller. It also contained optics to couple the pump laser and plasma emission into or out of their respective fibers. The pump coupler used two aligned fiber optic collimators to transfer light from the pump laser’s pigtail fiber the downhole going pump fiber, while the plasma emission coupler consisted of a LWP dichroic mirror to separate the plasma emission from the residual 1064 nm light and a pulse detector (amplified photodiode with a 1064 nm bandpass filter) to trigger the spectrometer.

The fiber umbilical that connects the sensor head and control unit consists of two 30 m, 600 μm core, NA = 0.54, high OH fiber optic cables (ThorLabs: FP600URT) contained within a protective PVC sleeve and a 30 m wire rope attached to the sensor head by an attachment hoop (see Fig. 2) for support. The fibers enter the sensor head via a short length of epoxy-filled, 3.2 mm, PVC tube passing through an IP68 cable gland. Based on manufacturer attenuation data^63^, UV attenuation is high over the 30 m fiber, with estimated internal transmission dropping from ~ 60% at 400 nm to nearly zero at 300 nm, while visible transmission (400–700 nm) climbs to greater than 80% for wavelengths between 500 and 700 nm. Fiber internal transmission at the laser wavelengths, 808 nm and 1064 nm, is estimated to be above 97% and 91% respectively (excluding reflection losses). The total measured transmission at 808 nm was 86%, with losses due primarily to surface reflections at the two uncoated fiber faces and minor clipping / vignetting in the coupling optics.

## Methods

### Calibration

**Figure 4:**
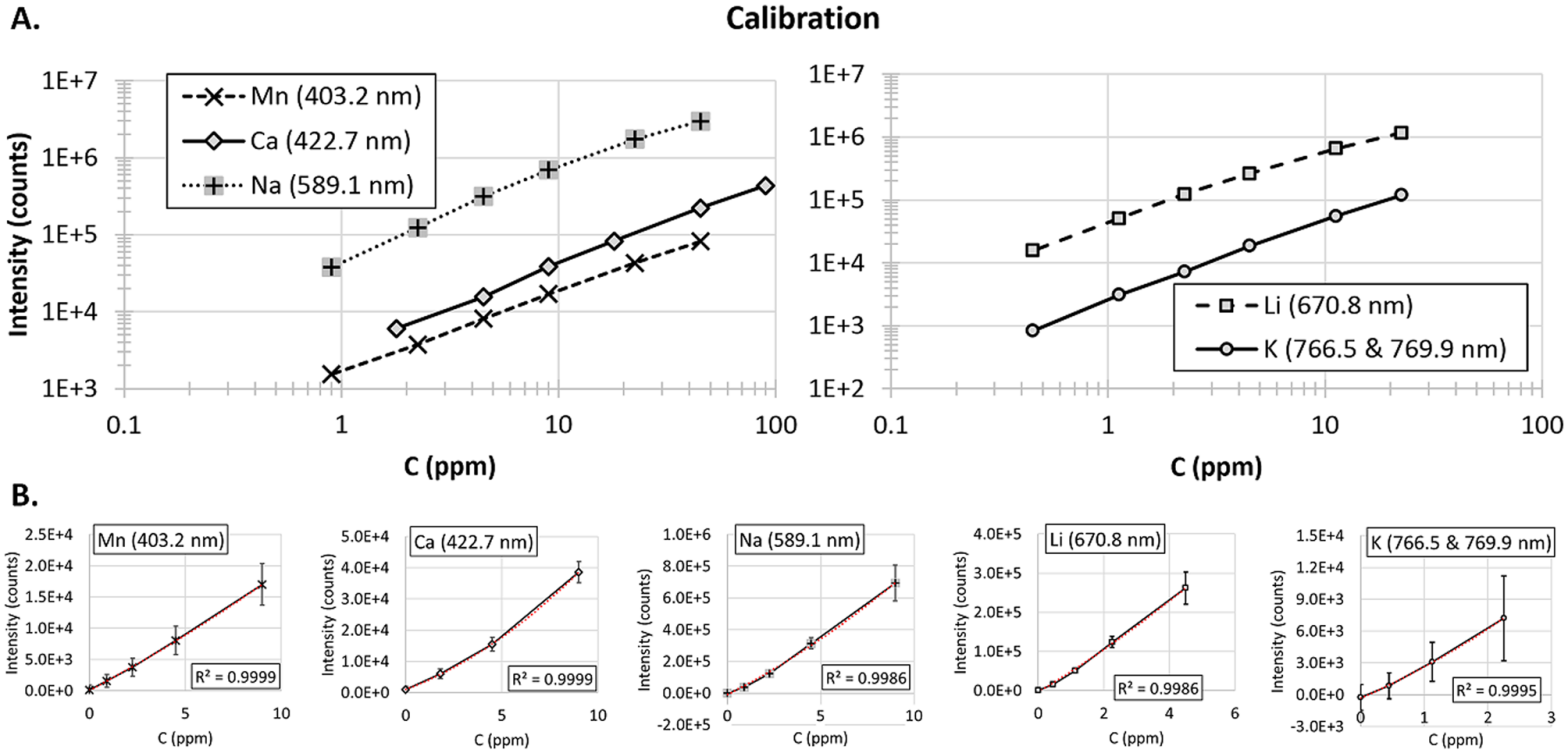
Calibration curves. (**A**) Full range calibration curves for six solutions (**A**–**F**) (see Table 1), excluding DI water/blank sample. (**B**) Quadratic fits at low concentrations, specifically solutions (**C**–**F**), & DI water (**D**–**F**, & DI water for K). Error bars show ± σ (standard deviation) of ten replicate measurements of 100 laser shots each.

A stock solution was made containing a mixture of Ca, Mn, Na, Li, and K chlorides (Fisher Scientific, reagent grade) in deionized (DI) water at cation concentrations of 900, 450, 450, 225, and 225 ppm respectively. Six calibration samples, A, B, C, D, E, and F (Table 1) were made from this stock solution by diluting it with DI water by factors of 10 (A), 20 (B), 50 (C), 100 (D), 200 (E), and 500 (F), see Fig. 4. DI water was used as a blank. Only the lower end of the concentration range, including the DI water blank, was used to construct the calibration curves (i.e., C–F & DI for Ca, Mn, Na, and Li and D–F & DI for K, see Fig. 4) as this concentration range better matched the concentrations found in the tested groundwater. A quadratic fit was used since a linear model provided a poor fit to the critical, low concentration values, which led to an overestimation of trace element concentrations. As shown in Fig. 4, the deviation from linear is not large and primarily affects the lowest one or two concentration points (i.e., DI water and sample F) where the signal background can have an outsized effect compared to higher analyte concentrations. Previous work has demonstrated that a quadratic calibration curve can offer better results than a linear curve^64^.

**Table 1:** Calibration solution composition spanning two orders of magnitude in concentration.

Calibration sample concentration (ppm)						
	A	B	C	D	E	F
Mn	45	22.5	9	4.5	2.25	0.9
Ca	90	45	18	9	4.5	1.8
Na	45	22.5	9	4.5	2.25	0.9
Li	22.5	11.25	4.5	2.25	1.125	0.45
K	22.5	11.25	4.5	2.25	1.125	0.45

Each solution, A–F, contained a mixture of the specified elements at the cationic concentrations given (in ppm).

Calibration spectra were collected in a laser safe enclosure at the test site. The prototype sensor head was immersed in a beaker containing 150 mL of solution (A-F and DI water), and 10 spectra consisting of 100 laser shots each were collected over three spectral ranges as follows; Range 1 = 330–470 nm (encompassing Mn and Ca emission lines), Range 2 = 570–700 nm (Na and Li emission lines), and Range 3 = 665–800 nm (K emission lines). Intensity values were calculated from the integrated area within 1.5 times the FWHM of the Mn 403.2 nm, Ca 422.7 nm, Na 589.1 nm, Li 670.8 nm, and the K 766.5 nm and 769.9 nm (summed) emission lines^65^. Backgrounds (excluding potassium) were taken as the average intensity of a spectral range free of emission lines (in both the calibration and groundwater data sets) near the analyte line and were subtracted before integration. Due to interference from luminescence originating in the sensor head optics, a 2nd order polynomial was fit to points on either side of the potassium emission lines, which was then subtracted before integration. The same methods were used in processing all data collected in this study.

Due to the strong attenuation wavelengths below ~ 400 nm by the sensor head optics, Mg was not measurable with the current setup. While it’s likely present in the groundwater as a trace element, the strongest emission lines of Mg lie in the UV portion of the spectrum (i.e., Mg I 285.21 nm and Mg II 279.55 & 280.27 nm)^65^ and thus could not be detected by our sensor. A weaker set of Mg emission lines around 383–384 nm^65^ were also not detected.

Limits of detection and quantification (LOD and LOQ) were calculated as follows^66^:

1 $$ \begin{gathered} LOD = {\raise0.7ex\hbox{${3\sigma }$} \!\mathord{\left/ {\vphantom {{3\sigma } m}}\right.\kern-0pt} \!\lower0.7ex\hbox{$m$}} \hfill \\ LOQ = {\raise0.7ex\hbox{${10\sigma }$} \!\mathord{\left/ {\vphantom {{10\sigma } m}}\right.\kern-0pt} \!\lower0.7ex\hbox{$m$}} = {\raise0.7ex\hbox{${10}$} \!\mathord{\left/ {\vphantom {{10} 3}}\right.\kern-0pt} \!\lower0.7ex\hbox{$3$}}LOD \hfill \\ \end{gathered} $$

where ‘m’ is the calibration curve slope, and ‘σ’ is the standard deviation of the background (over a 2.5–5 nm wavelength range of the DI water blank near the analyte line of interest). Note that a 3^rd^ order polynomial background was subtracted from this range prior to calculating ‘σ’, while ‘m’ was computed as the instantaneous slope of the quadratic calibration curve, $C\left( I \right)$, at an intensity value of ‘σ’ ($m = \left. {dC/dI} \right|_{I = \sigma }$). Also, note that these detection limits are for a single spectrum of one hundred laser shots. When averaging together multiple spectra, the LOD can be estimated, by using the relationship between the standard deviation (σ) and standard error of the mean (σ_mean_ = σ/√N)^67^, as follows:

2 $$ LOD_{N} = {\raise0.7ex\hbox{${3\left( {\sigma_{mean} } \right) }$} \!\mathord{\left/ {\vphantom {{3\left( {\sigma_{mean} } \right) } m}}\right.\kern-0pt} \!\lower0.7ex\hbox{$m$}} = {\raise0.7ex\hbox{${3\left( {\sigma /\sqrt N } \right)}$} \!\mathord{\left/ {\vphantom {{3\left( {\sigma /\sqrt N } \right)} m}}\right.\kern-0pt} \!\lower0.7ex\hbox{$m$}}{\raise0.7ex\hbox{${ = LOD_{0} }$} \!\mathord{\left/ {\vphantom {{ = LOD_{0} } {\sqrt N }}}\right.\kern-0pt} \!\lower0.7ex\hbox{${\sqrt N }$}} $$

where ‘N’ equals the number of replicates (i.e., number of spectra used to compute the average), ‘m’ and ‘σ’ are defined above, and ‘LOD_0_’ is the detection limit for a single spectrum of 100 laser shots. The LOD_0_ for the five calibrated elements are presented in Table 2.

**Table 2:** Detection and Quantification Limits (100 laser shots).

Element	Line (nm)	LOD_0_ (ppm)	LOQ_0_ (ppm)
Mn	403.2	1.0	3.3
Ca	422.7	0.41	1.37
Na	589.1	0.01	0.033
Li	670.8	0.01	0.033
K	766.5 + 769.9	0.22	0.73

## Results

### Subsurface testing

The prototype system was tested in a monitoring well located on the National Energy Technology Laboratory campus in Morgantown, WV, USA. The well has a depth of 30 ft (9.1 m) and a diameter of 4″ (10 cm). Groundwater started at a depth of 12 ft (3.7 m), and measurements were performed at a well depth of 27 ft (8.25 m) corresponding to a water depth of 15 ft (4.6 m). Measurements were performed over 20-days (April 15th–May 4th, 2021), excluding weekends, for a total of 14 days of data. Measurements were taken at approximately the same time each day over the three spectral ranges defined above. Each day, a total of 20 spectra were collected for Ranges 1 and 3 and 40 spectra for Range 2, with each spectrum being the sum of 100 laser shots. For the first half of the collection period (April 15th–23rd), the sensor head was raised ~ 3 ft above the level of the level of the groundwater at the end of each day, while from April 26th–May 4th, the sensor head was left submerged at a well depth of 27 ft. No water leaks were observed.

**Figure 5:**
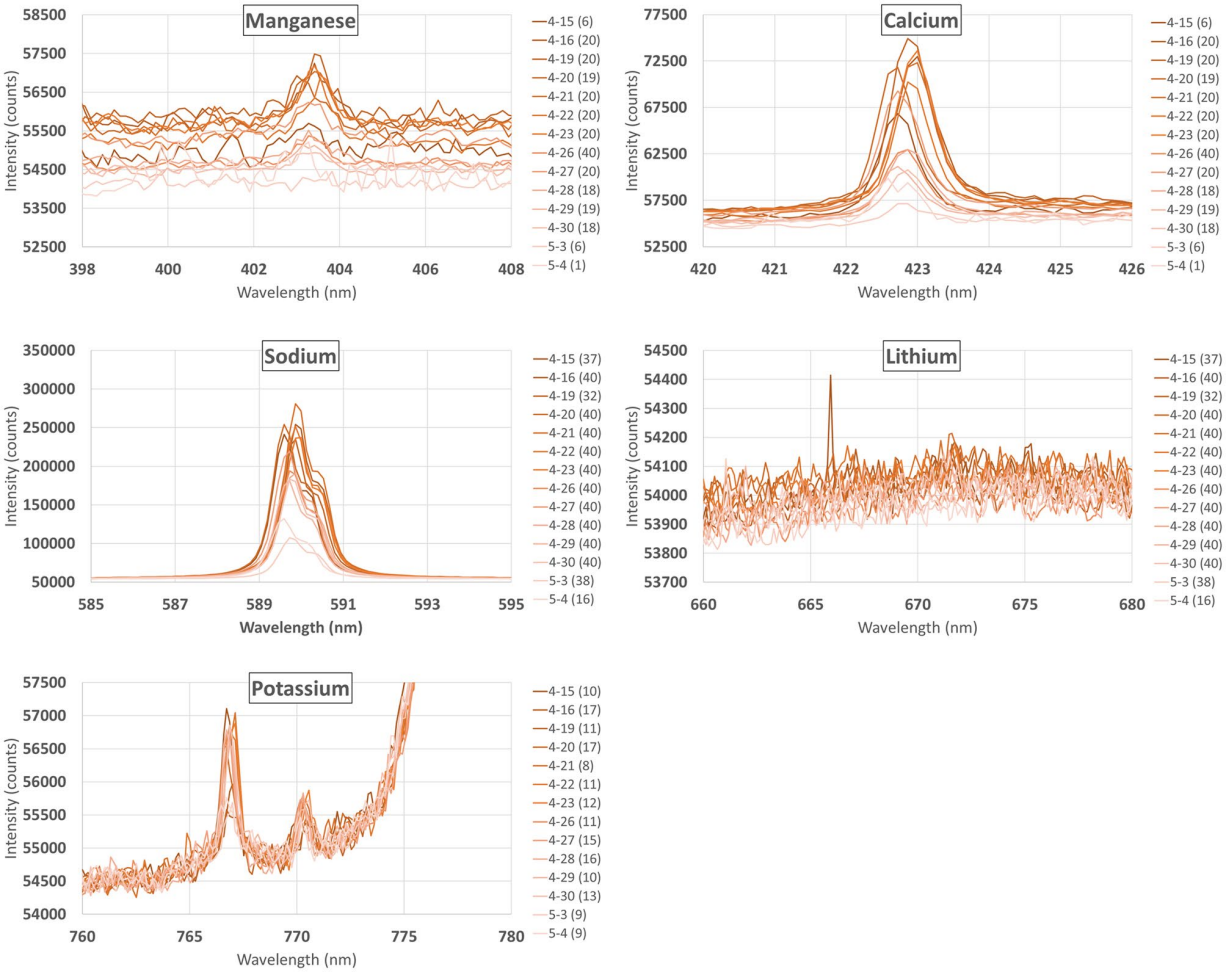
Averaged daily spectra of the calibrated elements (spectra affected by outgassing removed). Spectra are colored by collection date with the number of useable spectra given in parenthesis next to the date. K was affected by broad luminescence centered at ~ 780 nm (see Figure S3) and the Mn concentration was consistently below its LOQ. The full spectra are available in Supporting Information Figures S1–S3 and Tables S1–S3.

**Figure 6:**
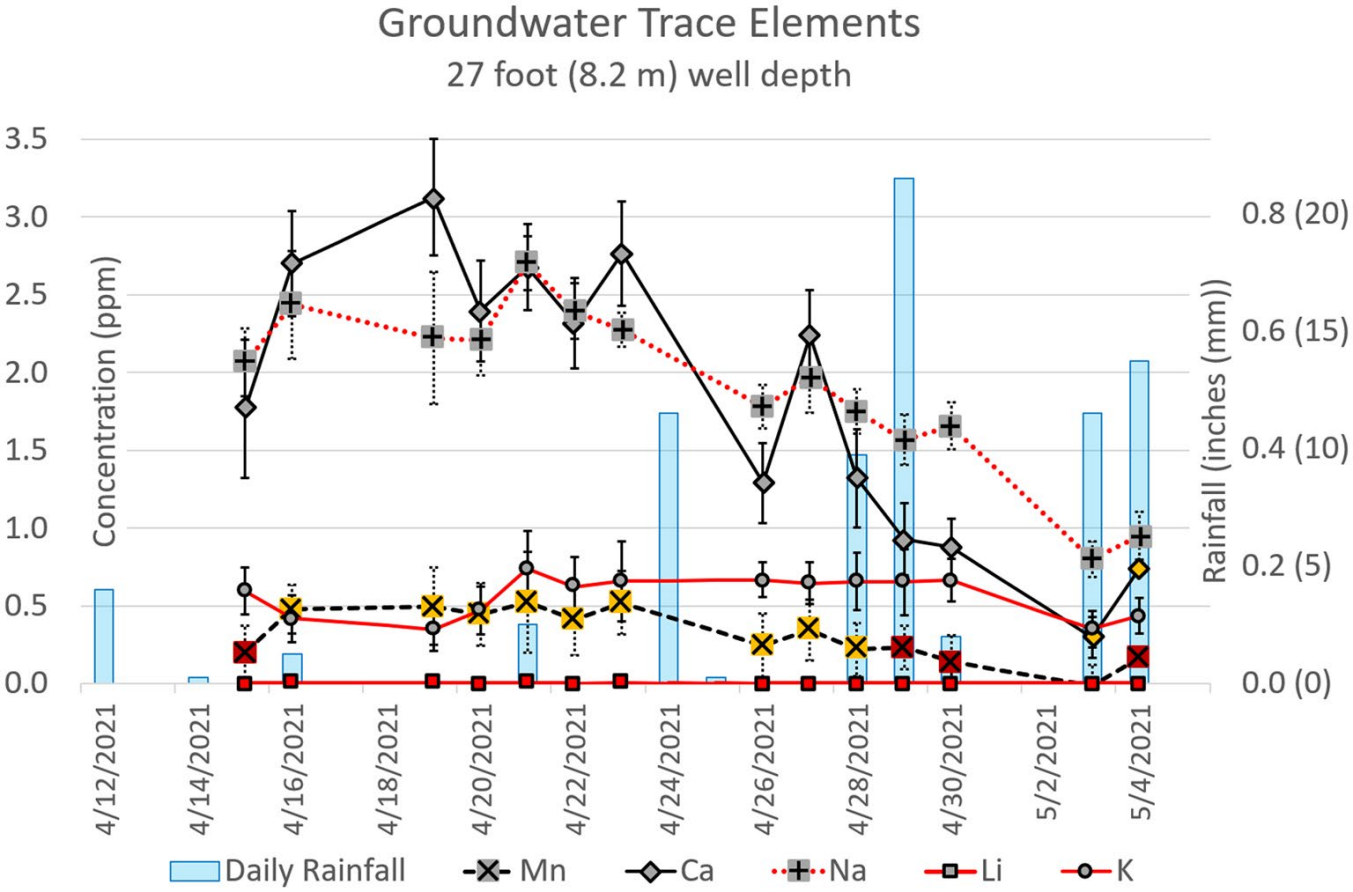
Groundwater trace element concentration over a 20-day period. Note that trace concentrations are mostly inversely proportional to local rainfall, becoming more dilute with increased precipitation. Error bars are ± σ (standard deviation) of the ‘N’ replicate measurements used for the daily average concentration (see Fig. 5). Note that on certain days, concentrations of Ca and Mn elements dropped below the LOQ (filled Yellow) or the LOD (filled Red). Li was not detected.

In-situ measurements detected trace amounts of Ca, Mn, Na, and K; however, Li was not detected (see Fig. 5). The measured concentrations of Ca and Na are correlated with the local rainfall (as measured by an onsite weather station) and decrease with increased rain, indicating dilution of the groundwater by rainwater (Fig. 6). This clearly demonstrates the connection between surface events and groundwater and shows the feasibility of the system to track real-world trace element variations.

Due to an issue caused by outgassing of the groundwater (described below), some of the 20 to 40 spectra collected each day in each spectral range had to be rejected, resulting in a variable LOD/LOQ from day to day based on the number of replicates (‘N’, Eq. (2)). When accounting for the variable LOD / LOQ together with the varying concentration, most of the measured elements remained above the LOQ throughout the 20 days with Ca dropping below the quantification limit on May 3^rd^ and 4^th^. However, because of its weak signal and low concentration, Mn remained below the LOQ throughout the measurement period and even dropped below the LOD on six days (April 15th and April 28th–May 4th).

### Lessons learned

Due to the downward-pointing design of the prototype sensor head, bubbles from groundwater degassing caused by the laser-induced shockwave accumulated on the sensor window, reducing or blocking the signal. This is despite the window being made flush with the enclosure face. Fortunately, not all the spectra collected each day were affected, and those that were could be manually removed. The criterion used to decide which spectra to reject was as follows: the strongest spectral line for a given range on a given day (specifically Ca—422 nm, Range 1; Na—589 nm, Range 2; and K—766 nm, Range 3) was compared across all 20 to 40 of the day’s spectra and any spectrum with an integrated peak intensity less than 50% the day’s strongest peak was rejected. The data sets from most days were only mildly affected. However, a few days experienced significant interference, with up to 70% of the day’s spectra rejected while one day (May 4th) had all but one spectrum in Range 1 (Mn and Ca) rejected (see Fig. 5). It must be noted that the worst two days (May 3rd and 4th) were preceded by several days of rain thus the groundwater likely contained more dissolved gas in addition to being severely diluted compared to previous days. While this demonstrates a serious issue with the current design, the main goal of this round of subsurface testing was to identify problems such as this. Future versions will be designed to address this issue by, for instance, turning the laser 90 degrees to fire out of the side of the enclosure. With the issue properly addressed, filtering of the data as described above should be unnecessary.

Additionally, as mentioned, for the first half of the measurement period the sensor head was raised above the groundwater level each day and lowered before the next day’s measurements. This revealed an issue caused by trapped air. The sensor head endcap (Fig. 2), made of stainless steel with multiple 3/16″ holes drilled through it, trapped enough air such that the laser spark was not in contact with the water. To clear the trapped air, the sensor head was raised out of the water and rapidly lowered several times. Future versions will need to address this issue as well. One possible solution is to provide one or more hydrophobic “channels” that allow gasses to be conducted out (as demonstrated by Meng et al.^68^) without encountering the resistance from surface tension that is likely preventing air from passing through the vent holes in the cap.

## Discussion

Overall, the prototype system performed well, and important lessons were learned that can be used to improve future designs. The most significant lessons were related to trapped gasses, either originating from groundwater outgassing or trapped surface air. Both these issues can be addressed in future designs by a number of means, such as redirecting 90 degrees to avoid accumulation bubbles or providing an escape path for trapped air. Also, in designing the sensor head, a number of translational adjustments were included, necessitating a rail and discrete, detachable mounts for all components. An optimized optical design and precision assembly would eliminate the need for most adjustments allowing for a significantly more compact mechanical design. For instance, since the largest optical components in the current design are ½” (12.7 mm) in diameter, the optical system could theoretically fit inside a ~ 1″ (~ 25.4 mm) inner diameter tube, making the sensor significantly smaller and permitting access to 2″ (50.8 mm) diameter well bores.

Improvements to the optical design would increase the system’s UV sensitivity. As previously mentioned, UV materials for the optical components could be employed to increase signal throughput for UV emitting elements like Mg. Additionally, tradeoffs are often made with elements like the DCM, for instance choosing one with good UV performance can require accepting poor performance at longer wavelengths.

Although the LIBS technique is inherently multi-analyte, the spectrograph’s resolution and range generally limit the ability to measure all analytes simultaneously except in certain cases. High spectral resolution is necessary to separate closely spaced emission lines of different analytes while a large spectral range is needed to detect as many analyte emission lines as possible. There is, however, an inverse relationship between resolution and range for the type of spectrograph used in this work. The typical way to deal with this is to sequentially measure multiple, narrower spectral regions by tuning the grating angle as was demonstrated in this study.

While measurements were only performed once per day in this study, the equipment could be configured to automatically collect spectra, thus providing a nearly continuous, real-time stream of data. Each set of measurements only took between 200 and 400 s in this study, and with three spectral ranges measured per data set (two at 200 s each and one at 400 s), the total measurement time was 800 s, or 13 min 20 s. Depending on the number of spectral ranges measured, number of spectra collected per range, number of laser shots per spectrum, the laser repetition rate, and desired sensitivity level, data could be collected at a rate of a few minutes to a few 10’s of minutes per data set. Thus, the system would be able to readily identify change in subsurface conditions on that time scale or longer.

## Conclusion

Previous work undertaken at NETL and elsewhere has demonstrated that a miniaturized, LIBS-based sensor could successfully operate in the subsurface environment and perform meaningful trace element measurements. In this study, we have used lessons learned from this previous work to design a full prototype system. The prototype sensor-head, designed to reach a depth of 100 ft (30 m), was successfully deployed to a subsurface depth of 27 ft (8 m), where it performed measurements over a 20-day period. The concentrations of Mn, Ca, Na, Li, and K were tracked over this period and varied over a range of approx. 0–3 ppm. Measured concentrations were correlated with the local rainfall, in that, trace element concentrations dropped with increased rain. This work clearly demonstrates the ability of subterranean LIBS to monitor groundwater resources continuously, in-situ, and in near real-time at the ppm concentration level.

## Supplementary Information

Supplementary Information.

## Data Availability

All data generated or analyzed during this study are included in this published article [and its supplementary information files].
