# Supplementary material for: Design, construction, and validation of an in-situ groundwater trace element analyzer with applications in carbon storage
Source: Sci Rep. 2023 May 9;13:7516. doi: 10.1038/s41598-023-32788-x (PMC10169803; doi:10.1038/s41598-023-32788-x)
Supplement: Supplementary file 1 — Supplementary Information. [file 41598_2023_32788_MOESM1_ESM.docx]

**Design, Construction, and Validation of an In-situ Groundwater Trace Element Analyzer with Applications in Carbon Storage**

Daniel A. Hartzler^1,2^; Chet R. Bhatt^1,2^; and Dustin L. McIntyre^1^

^1^National Energy Technology Laboratory, 3610 Collins Ferry Road, Morgantown, WV 26507, USA

^2^NETL Support Contractor, 3610 Collins Ferry Road, Morgantown, WV 26507, USA

**Supporting Information**

The following spectra are the daily average of all useable spectra collected between April 15 and May 5, 2021. Each day (excluding weekends) between 4-6 pm, 20 to 40 spectra across three spectral ranges were collected at a well depth of 27 ft, where each spectrum is the accumulated intensity of 100 individual laser shots. Due to the downward pointing probe design, data collection was affected by bubbles collecting on the probe window and interfering with laser spark generation and light collection, requiring some spectra to be thrown out. The number of useable spectra included in the daily average are given in parentheses after the collection dates in Figures S1 - S3 and Tables S1 - S3.

**Spectral Range 1: 330 – 470 nm**

**
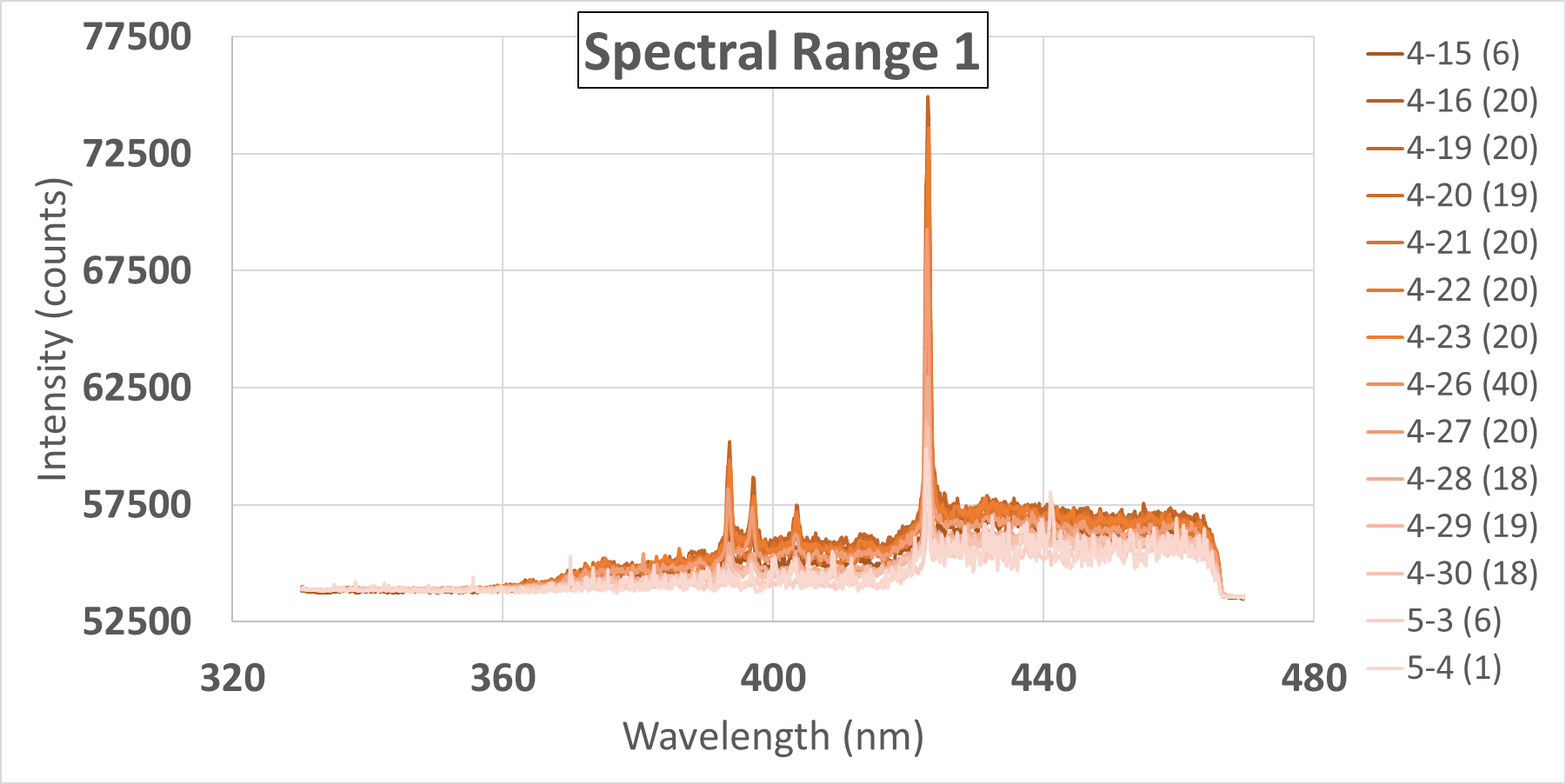
**

**Figure S1:** Range 1 – Data collected between 4-15 and 5-4 showing emission lines of calcium (Ca) and manganese (Mn).

**Table S1:** Range 1 - Column labels indicate the collection date and number of useable spectra included in the average.

| **wL(nm)** | **4-15 (6)** | **4-16 (20)** | **4-19 (20)** | **4-20 (19)** | **4-21 (20)** | **4-22 (20)** | **4-23 (20)** | **4-26 (40)** | **4-27 (20)** | **4-28 (18)** | **4-29 (19)** | **4-30 (18)** | **5-3 (6)** | **5-4 (1)** |
| --- | --- | --- | --- | --- | --- | --- | --- | --- | --- | --- | --- | --- | --- | --- |
| 330.2744 | 53807.33 | 53899.95 | 53975.3 | 53839.32 | 53861.5 | 53892.85 | 53893.6 | 53910.65 | 53857.9 | 53907 | 53876.16 | 53898.17 | 53922.33 | 53869 |
| 330.4088 | 53980.83 | 53910.4 | 53861.8 | 53912 | 53921 | 53868.8 | 53863.35 | 53943.08 | 53871.45 | 53906.89 | 53936.11 | 53934.83 | 53875 | 53938 |
| 330.5432 | 53896.33 | 53878.1 | 53832.05 | 53888.05 | 53904.1 | 53897 | 53902.7 | 53868.13 | 53906.65 | 53889.11 | 53922.53 | 53917.33 | 53896.33 | 53905 |
| 330.6776 | 53822.5 | 53850.1 | 53846.6 | 53883.89 | 53896.65 | 53905.1 | 53905.25 | 53850.48 | 53873.4 | 53884.61 | 53909.37 | 53898.72 | 53915.5 | 53886 |
| 330.8121 | 53808.5 | 53830.45 | 53861.9 | 53874 | 53888 | 53899 | 53901.95 | 53851.18 | 53871.7 | 53889.78 | 53899.95 | 53892.72 | 53910.17 | 53878 |
| 330.9466 | 53799 | 53822.4 | 53864.65 | 53875.21 | 53879.55 | 53884.1 | 53895.5 | 53847.78 | 53872.85 | 53890.78 | 53894.05 | 53882.83 | 53901 | 53841 |
| 331.081 | 53778.67 | 53825 | 53869.4 | 53862.16 | 53876.15 | 53877.8 | 53891.95 | 53844.43 | 53871.25 | 53893.5 | 53886 | 53874.61 | 53906.33 | 53847 |
| 331.2155 | 53769 | 53822.9 | 53864.1 | 53849.63 | 53867.7 | 53865.05 | 53878.45 | 53838.9 | 53865.6 | 53886.22 | 53875.63 | 53859.11 | 53890.33 | 53872 |
| 331.35 | 53766 | 53824.05 | 53861 | 53840.37 | 53865.4 | 53862.55 | 53879.75 | 53842.13 | 53859.8 | 53888.44 | 53875.47 | 53859.78 | 53888.5 | 53841 |
| 331.4845 | 53760 | 53820 | 53855.45 | 53833.63 | 53866.1 | 53857.85 | 53873.95 | 53839.7 | 53855.35 | 53888.61 | 53862.26 | 53854.22 | 53872.17 | 53884 |
| 331.6191 | 53763 | 53824.7 | 53857.6 | 53830 | 53863.7 | 53855.45 | 53872.8 | 53835.28 | 53855.05 | 53882.22 | 53860.37 | 53852.56 | 53882.83 | 53842 |
| 331.7536 | 53754.33 | 53815.55 | 53850.2 | 53831.42 | 53859.95 | 53846.35 | 53868.85 | 53835.05 | 53851.6 | 53878.89 | 53853.63 | 53848.33 | 53877.67 | 53857 |
| 331.8882 | 53751.67 | 53819 | 53848.85 | 53824.37 | 53859.1 | 53846.45 | 53864 | 53831.95 | 53849.85 | 53880.22 | 53852.26 | 53836.61 | 53871.83 | 53840 |
| 332.0227 | 53754.67 | 53815.4 | 53847.2 | 53822.68 | 53850.05 | 53844.6 | 53860.15 | 53829.9 | 53845.8 | 53876.89 | 53845.79 | 53843.89 | 53870.5 | 53854 |
| 332.1573 | 53757.67 | 53821.85 | 53848.65 | 53826.84 | 53850.4 | 53849.6 | 53863.3 | 53831.45 | 53849.3 | 53879.28 | 53852.16 | 53837 | 53879.83 | 53839 |
| 332.2919 | 53753.67 | 53824.55 | 53856.7 | 53826.74 | 53859.7 | 53851.2 | 53870.3 | 53836 | 53850.7 | 53877.61 | 53847.26 | 53842.06 | 53883.17 | 53861 |
| 332.4265 | 53747.33 | 53811.15 | 53836.9 | 53814.37 | 53847 | 53834.85 | 53858.15 | 53822.83 | 53838.05 | 53863.61 | 53843.53 | 53827.44 | 53861 | 53826 |
| 332.5611 | 53749.33 | 53805.75 | 53839.35 | 53815.21 | 53848.75 | 53836.7 | 53852.3 | 53821.93 | 53837.35 | 53860.94 | 53837.37 | 53828.89 | 53870 | 53833 |
| 332.6957 | 53745.5 | 53803.7 | 53831.75 | 53810.63 | 53843.7 | 53829.2 | 53850.65 | 53821.3 | 53838.85 | 53864.61 | 53837.42 | 53826 | 53857.67 | 53833 |
| 332.8303 | 53745.83 | 53802.05 | 53837.65 | 53811.74 | 53849.75 | 53834.4 | 53853.2 | 53820.78 | 53836.5 | 53864.67 | 53840.63 | 53833 | 53861 | 53834 |
| 332.965 | 53738.5 | 53802.35 | 53836.2 | 53811 | 53853.6 | 53826.7 | 53849.4 | 53816.7 | 53832.2 | 53859.67 | 53835.05 | 53820.72 | 53866.17 | 53818 |
| 333.0996 | 53745 | 53803.45 | 53832.3 | 53810.05 | 53845.75 | 53832.75 | 53851.95 | 53818.15 | 53830.8 | 53860.17 | 53833.95 | 53829.67 | 53855.67 | 53848 |
| 333.2343 | 53739 | 53797.7 | 53830.5 | 53805.79 | 53843.8 | 53831.05 | 53853.75 | 53818.35 | 53834.15 | 53860.61 | 53833.32 | 53822.22 | 53860.33 | 53813 |
| 333.369 | 53737.17 | 53794.3 | 53832.4 | 53813 | 53842.25 | 53833.95 | 53850.2 | 53813.05 | 53832.55 | 53858.22 | 53834.05 | 53825.28 | 53863.83 | 53827 |
| 333.5037 | 53735.67 | 53794.65 | 53827.95 | 53806.58 | 53846.15 | 53822.95 | 53843.8 | 53809.93 | 53831.2 | 53856.61 | 53832.74 | 53821.28 | 53853 | 53837 |
| 333.6384 | 53728.33 | 53796.75 | 53827.8 | 53807.11 | 53841 | 53834.45 | 53840.1 | 53811.63 | 53831.1 | 53856.83 | 53833.47 | 53820.39 | 53850.5 | 53818 |
| 333.7731 | 53735 | 53795.05 | 53832.2 | 53806.63 | 53838.55 | 53827.9 | 53847.75 | 53810.58 | 53830 | 53854.67 | 53836 | 53822.44 | 53849.67 | 53830 |
| 333.9078 | 53740.33 | 53791.85 | 53830.8 | 53809.32 | 53842.6 | 53826.85 | 53844.8 | 53812.8 | 53832.3 | 53859.39 | 53832.05 | 53822.89 | 53852.17 | 53834 |
| 334.0425 | 53748.17 | 53795.55 | 53830.2 | 53809.58 | 53841.5 | 53838.25 | 53851.75 | 53813.93 | 53839.95 | 53860.56 | 53833.84 | 53826.56 | 53853.17 | 53844 |
| 334.1773 | 53746.33 | 53797 | 53847.9 | 53828.37 | 53848.35 | 53848.7 | 53855.3 | 53822.23 | 53842.9 | 53859.78 | 53836.79 | 53827.5 | 53854.67 | 53836 |
| 334.3121 | 53745.5 | 53802.4 | 53837.15 | 53823.47 | 53850.7 | 53836.9 | 53852.3 | 53818.85 | 53837.25 | 53859.67 | 53841 | 53842.94 | 53859.83 | 53836 |
| 334.4468 | 53746.67 | 53805.85 | 53839.65 | 53855.63 | 53876.5 | 53841.2 | 53855 | 53830.7 | 53859.45 | 53866.67 | 53842.79 | 53847.61 | 53870.83 | 53822 |
| 334.5816 | 53765 | 53822.95 | 53835.4 | 53866.42 | 53865.8 | 53846.9 | 53855.65 | 53829.95 | 53875.55 | 53889.28 | 53857.05 | 53853.44 | 53861.33 | 53830 |
| 334.7164 | 53780.33 | 53820 | 53846.2 | 53833.21 | 53873.55 | 53851.5 | 53863.45 | 53847.48 | 53878.95 | 53888.06 | 53857.11 | 53856.56 | 53922.83 | 53826 |
| 334.8512 | 53755 | 53827.95 | 53855 | 53825.68 | 53848.7 | 53883.85 | 53867.9 | 53861.83 | 53861.6 | 53897 | 53861.37 | 53837.83 | 53882.33 | 53859 |
| 334.9861 | 53782 | 53828.05 | 53857.05 | 53840.47 | 53858.25 | 53877.45 | 53880.9 | 53885.28 | 53875.15 | 53896.17 | 53866.37 | 53867.67 | 53887.67 | 54105 |
| 335.1209 | 53763.5 | 53840.35 | 53869.35 | 53895.74 | 53876.7 | 53866.1 | 53853.35 | 53862.3 | 53895.15 | 53905.67 | 53859.47 | 53864.89 | 53857 | 53984 |
| 335.2557 | 53781.33 | 53842.95 | 53878.75 | 53871 | 53875.95 | 53893.4 | 53883.9 | 53851.73 | 53875.7 | 53948.94 | 53850.47 | 53858.11 | 53861 | 53848 |
| 335.3906 | 53822.33 | 53838.25 | 53874.35 | 53866.37 | 53884.6 | 53871.25 | 53874.3 | 53863.28 | 53852.35 | 53939.28 | 53844.16 | 53909.67 | 53882 | 53840 |
| 335.5255 | 53880.5 | 53821.15 | 53858.15 | 53865.32 | 53872.6 | 53869.5 | 53862.9 | 53852.6 | 53879.8 | 53959.89 | 53849.42 | 53883.78 | 53909.5 | 53948 |
| 335.6603 | 53761.17 | 53839.25 | 53870.8 | 53884.89 | 53893.3 | 53870.8 | 53865.7 | 53865.2 | 53873.35 | 53939.5 | 53874.32 | 53875.89 | 53891.83 | 53899 |
| 335.7952 | 53765.17 | 53831.35 | 53906 | 53859.47 | 53902.85 | 53901.05 | 53890.4 | 53867.43 | 53872.1 | 53905.56 | 53865.79 | 53880.06 | 53881.17 | 53820 |
| 335.9301 | 53784.5 | 53852.55 | 53887.15 | 53881.53 | 53885.15 | 53872.5 | 53895.95 | 53864.03 | 53884.25 | 53917.44 | 53857.37 | 53874.33 | 53907.5 | 53825 |
| 336.065 | 53823.17 | 53828.45 | 53868.2 | 53872.11 | 53915.05 | 53863.75 | 53902.85 | 53845.85 | 53863.3 | 53907.89 | 53860.79 | 53843.67 | 53965.83 | 53822 |
| 336.2 | 53920.17 | 53837.8 | 53877.1 | 53867.37 | 53927.65 | 53891.55 | 53871.75 | 53871.53 | 53924.3 | 53915.78 | 53861.63 | 53848.94 | 53924 | 53838 |
| 336.3349 | 53945.17 | 53852.2 | 53886.2 | 53874.47 | 53897.8 | 53888.25 | 53909.9 | 53846.7 | 53880 | 53939.89 | 53859.42 | 53868.94 | 53872.33 | 53959 |
| 336.4698 | 53810 | 53914.4 | 53920.05 | 53850.21 | 53900.4 | 53884.65 | 53900.15 | 53866.65 | 53875.7 | 53947.28 | 53891.32 | 53889.17 | 53897 | 54001 |
| 336.6048 | 53805.67 | 53887.6 | 53898.85 | 53845.37 | 53913.7 | 53905.25 | 53906.7 | 53862.5 | 53883.65 | 53920.11 | 53869.21 | 53875.78 | 53905 | 53831 |
| 336.7398 | 53846.67 | 53871.25 | 53878.3 | 53853.84 | 53923.9 | 53887.85 | 53938.65 | 53843.18 | 53873.65 | 53902.83 | 53883.95 | 53875.06 | 53882.17 | 53809 |
| 336.8747 | 53947.5 | 53859.4 | 53918.9 | 53855.42 | 53943.1 | 53871.55 | 53888.7 | 53845.18 | 53894.1 | 53893.22 | 53864.42 | 53865.56 | 53892.5 | 53820 |
| 337.0097 | 53836.33 | 53870.55 | 53889.7 | 53853.37 | 53936.15 | 53896.25 | 53890.15 | 53840.28 | 53927.7 | 53929.5 | 53876 | 53852.06 | 53888 | 53811 |
| 337.1447 | 53789.33 | 53831.55 | 53917.65 | 53863 | 53900.5 | 53854.9 | 53888.55 | 53865.73 | 53922.6 | 53900.56 | 53894.21 | 53870.78 | 53909 | 53826 |
| 337.2797 | 53782.17 | 53837.75 | 53892.8 | 53910.21 | 53966.35 | 53862.5 | 53918.4 | 53852.1 | 53911.05 | 53882.83 | 53869.58 | 53879.33 | 53921.5 | 53823 |
| 337.4148 | 53822 | 53868.1 | 53881.95 | 53852.68 | 53894.6 | 53861.15 | 53927 | 53865.95 | 53880 | 53904.17 | 53892.11 | 53842.06 | 53865.83 | 53853 |
| 337.5498 | 53834.5 | 53860.8 | 53873.1 | 53831.89 | 53883.25 | 53880.3 | 53917.5 | 53884 | 53878.3 | 53924.67 | 53874.11 | 53850.17 | 53884.17 | 53940 |
| 337.6848 | 53810.67 | 53843.85 | 53884.1 | 53843.68 | 53934.3 | 53898 | 53960.6 | 53889.43 | 53870.75 | 53907.39 | 53882.95 | 53869.39 | 53881.83 | 53840 |
| 337.8199 | 53808.33 | 53841.7 | 53867.95 | 53850.58 | 53958 | 53872.1 | 53948.7 | 53877.85 | 53887.85 | 53892.44 | 53883.84 | 53879.28 | 53868.67 | 53855 |
| 337.955 | 53809.67 | 53848.35 | 53877.3 | 53871.26 | 53988.25 | 53886.05 | 53877.75 | 53877.08 | 53875.7 | 53882.17 | 53893.11 | 53868.06 | 53929.33 | 53834 |
| 338.09 | 53763 | 53884.05 | 53892.8 | 53870.32 | 53973.2 | 53905.25 | 53869 | 53866.35 | 53933.05 | 53882.39 | 53924.68 | 53876.06 | 53964.33 | 54011 |
| 338.2251 | 53747.83 | 53851.85 | 53904.35 | 53865.26 | 53948.85 | 53910.35 | 53862.6 | 53863.25 | 53914.8 | 53868.28 | 53872.47 | 53887.56 | 53859.17 | 54207 |
| 338.3602 | 53730.83 | 53849.65 | 53902.85 | 53866.42 | 53912.3 | 53857.6 | 53893.5 | 53851.98 | 53855.4 | 53909.78 | 53867.63 | 53863.17 | 53864.5 | 53829 |
| 338.4953 | 53725.5 | 53835.05 | 53936.15 | 53854.47 | 53868.35 | 53860.95 | 53874.25 | 53851.6 | 53864.85 | 53947.72 | 53866.26 | 53900.44 | 53863.5 | 53818 |
| 338.6305 | 53732.5 | 53847.45 | 53933.5 | 53894.47 | 53875.4 | 53872.95 | 53875 | 53851.13 | 53865.4 | 53923.28 | 53892.58 | 53883.78 | 53892.67 | 53811 |
| 338.7656 | 53744.67 | 53911.75 | 53967 | 53862.68 | 53892.6 | 53913.55 | 53888.4 | 53842.93 | 53858.4 | 53915.83 | 53869.26 | 53911.39 | 53877.17 | 53824 |
| 338.9007 | 53780.83 | 53884 | 53895.15 | 53879.68 | 53876.05 | 53877.2 | 53918.8 | 53839.73 | 53881.5 | 53908.22 | 53856.53 | 53913.61 | 53864.83 | 53793 |
| 339.0359 | 53753.67 | 53855.6 | 53845.15 | 53860.58 | 53882.35 | 53882.75 | 53921.65 | 53873.03 | 53890.3 | 53912.33 | 53908.89 | 53878 | 53864.83 | 53809 |
| 339.1711 | 53787 | 53906.3 | 53851.1 | 53878 | 53893.15 | 53860.95 | 53895.85 | 53884.23 | 53885.1 | 53917 | 53882.74 | 53894.89 | 53892.17 | 53823 |
| 339.3062 | 53830.5 | 53932.25 | 53861.75 | 53840.74 | 53893.2 | 53882.4 | 53871.35 | 53881.5 | 53872.35 | 53970.67 | 53879.63 | 53901.83 | 53892 | 53814 |
| 339.4414 | 53815.17 | 53895.1 | 53867.15 | 53833.47 | 53891.1 | 53898.25 | 53878.8 | 53864.3 | 53865.8 | 53908.61 | 53880.21 | 53856.56 | 53854.5 | 53823 |
| 339.5766 | 53754.33 | 53846.45 | 53875.1 | 53850.21 | 53902.6 | 53928.25 | 53921.2 | 53846.1 | 53882.35 | 53902.72 | 53877.68 | 53863.39 | 53871.83 | 53808 |
| 339.7118 | 53771.5 | 53860.25 | 53890 | 53884.95 | 53878.65 | 53902.35 | 53928.6 | 53847.4 | 53909.65 | 53900.11 | 53931.95 | 53883.78 | 53858.67 | 53795 |
| 339.847 | 53749.17 | 53836.3 | 53879 | 53897.26 | 53883.25 | 53924 | 53907.45 | 53847.73 | 53863.85 | 53907.94 | 53895.63 | 53861.72 | 53900.67 | 53825 |
| 339.9822 | 53861.17 | 53852.1 | 53914.65 | 53864.58 | 53894.9 | 53920.2 | 53890.15 | 53861.9 | 53884.1 | 53914.28 | 53858.53 | 53915.33 | 53939.83 | 53833 |
| 340.1175 | 53898 | 53837.4 | 53879.25 | 53893.68 | 53867.05 | 53898.3 | 53921.4 | 53846.15 | 53900.95 | 53905.5 | 53876.95 | 53870.44 | 53904.5 | 53819 |
| 340.2527 | 53804.83 | 53878.65 | 53889.65 | 53899.74 | 53902.6 | 53896.5 | 53965.75 | 53850.35 | 53874 | 53914.06 | 53875.58 | 53851.22 | 53939 | 53844 |
| 340.388 | 53783 | 53847.85 | 53914.65 | 53881.84 | 53939.95 | 53879.95 | 53955.25 | 53844.4 | 53868.8 | 53929.83 | 53864.16 | 53860.89 | 53950.5 | 53888 |
| 340.5233 | 53817.33 | 53855.8 | 53904.15 | 53896.53 | 53902.65 | 53868.55 | 53927.8 | 53857.3 | 53873.75 | 53923.28 | 53866.84 | 53878.72 | 53892.67 | 53991 |
| 340.6585 | 53787.17 | 53875.7 | 53910 | 53916.95 | 53881.8 | 53880.4 | 53916.25 | 53873.38 | 53879.1 | 53903.44 | 53874.53 | 53854.28 | 53875.33 | 54043 |
| 340.7938 | 53784 | 53850.35 | 53896.55 | 53849.68 | 53893.15 | 53902.15 | 53898.35 | 53856.18 | 53895.95 | 53899.61 | 53860.37 | 53847.89 | 53854.33 | 53816 |
| 340.9291 | 53802.33 | 53848.35 | 53899.65 | 53860.21 | 53882.15 | 53908.65 | 53870.55 | 53884.95 | 53875.8 | 53955.56 | 53872.11 | 53875.5 | 53850.67 | 53851 |
| 341.0644 | 53758.17 | 53834.45 | 53900.7 | 53855.68 | 53858.9 | 53900.65 | 53899.55 | 53871.73 | 53897.8 | 53907 | 53868 | 53857.56 | 53848.83 | 54016 |
| 341.1997 | 53785 | 53821.3 | 53879.45 | 53893.53 | 53865.2 | 53869 | 53916.15 | 53837.38 | 53885.6 | 53912.56 | 53866.74 | 53872.78 | 53857.33 | 53841 |
| 341.3351 | 53825.33 | 53860.35 | 53878.3 | 53874.42 | 53930.65 | 53886.6 | 53899.25 | 53833.15 | 53889.2 | 53929.72 | 53883.21 | 53888.67 | 53892.17 | 53826 |
| 341.4704 | 53840 | 53852.15 | 53875.95 | 53850.42 | 53912.4 | 53880.35 | 53872.2 | 53846.63 | 53886.1 | 53900.28 | 53880.37 | 53896.72 | 53954.33 | 53788 |
| 341.6058 | 53872.83 | 53841.1 | 53877.4 | 53858.95 | 53857.6 | 53862.95 | 53887.25 | 53855.83 | 53922.7 | 53903.5 | 53864.32 | 53927.22 | 53887.67 | 53799 |
| 341.7411 | 53922.67 | 53892.55 | 53900.45 | 53889.16 | 53905.25 | 53883.95 | 53910.05 | 53857.55 | 53859.6 | 53902.83 | 53875.53 | 53907.56 | 53871.83 | 53811 |
| 341.8765 | 53766.83 | 53883.95 | 53886.7 | 53853.21 | 53864.8 | 53887.5 | 53900.2 | 53842.9 | 53836.9 | 53896.5 | 53845.89 | 53863.83 | 53886.83 | 53835 |
| 342.0119 | 53767.67 | 53851.75 | 53876.15 | 53858.47 | 53851.4 | 53913.1 | 53867.8 | 53855.45 | 53871.15 | 53900.11 | 53885.68 | 53871.5 | 53909.33 | 53810 |
| 342.1473 | 53801.17 | 53856.25 | 53890.75 | 53888.05 | 53899.85 | 53889.15 | 53910.1 | 53854.43 | 53875.95 | 53932 | 53896.53 | 53851.56 | 53866.17 | 53887 |
| 342.2827 | 53811.5 | 53863.75 | 53877.8 | 53920.26 | 53886.15 | 53857.5 | 53877.45 | 53866 | 53859.25 | 53917.44 | 53910.58 | 53868.17 | 53874.17 | 54002 |
| 342.4181 | 53767 | 53856.75 | 53906.1 | 53884.37 | 53852.75 | 53877.05 | 53877.7 | 53856 | 53872.5 | 53901 | 53883.53 | 53882.61 | 53943 | 54125 |
| 342.5535 | 53802.83 | 53893.4 | 53909.25 | 53852.95 | 53864.05 | 53873.8 | 53899.9 | 53832.98 | 53883.75 | 53928.17 | 53874.68 | 53898.17 | 53872.17 | 53877 |
| 342.6889 | 53803.33 | 53843.6 | 53904.35 | 53854.79 | 53911.95 | 53888.05 | 53900.4 | 53838.1 | 53865.35 | 53916.56 | 53891.21 | 53892.22 | 53852.17 | 53818 |
| 342.8244 | 53741.83 | 53852.15 | 53916.8 | 53818.95 | 53874.4 | 53901.1 | 53884.85 | 53845.25 | 53851.2 | 53910.72 | 53868.84 | 53879.44 | 53879.17 | 53922 |
| 342.9598 | 53737.33 | 53841.2 | 53901.6 | 53842.37 | 53882.9 | 53888.75 | 53891.3 | 53847.73 | 53844.5 | 53874.06 | 53869.05 | 53887.94 | 53863.67 | 53991 |
| 343.0953 | 53792.67 | 53811.1 | 53909.8 | 53826.05 | 53865.4 | 53851 | 53893.6 | 53815.55 | 53855.7 | 53898.17 | 53862.11 | 53841.94 | 53889 | 54054 |
| 343.2308 | 53776.83 | 53831.35 | 53895.9 | 53833.53 | 53869.4 | 53880.8 | 53888.15 | 53817 | 53870.3 | 53926.72 | 53866.21 | 53858.78 | 53933 | 53845 |
| 343.3662 | 53769.67 | 53859.3 | 53869.85 | 53831.21 | 53868.75 | 53866.5 | 53887 | 53826.33 | 53874.15 | 53925.61 | 53862.84 | 53894.28 | 53881.5 | 53814 |
| 343.5017 | 53761.5 | 53852.2 | 53870.2 | 53860.11 | 53869.5 | 53848.45 | 53874.15 | 53820.33 | 53872.95 | 53915.72 | 53860.26 | 53889.06 | 53875 | 53827 |
| 343.6372 | 53736.83 | 53821.45 | 53850.75 | 53873.68 | 53860.4 | 53863.05 | 53877.05 | 53822.13 | 53875.4 | 53909.83 | 53903.74 | 53897.44 | 53897.17 | 53787 |
| 343.7727 | 53742 | 53809.4 | 53871.1 | 53820 | 53889.3 | 53864.6 | 53852.25 | 53827.85 | 53882.2 | 53874.72 | 53893.32 | 53916.94 | 53936 | 53801 |
| 343.9083 | 53738 | 53844.15 | 53872.85 | 53835.47 | 53883.9 | 53876.45 | 53866.4 | 53836.13 | 53831.3 | 53889.72 | 53889.26 | 53864.11 | 53849.83 | 53806 |
| 344.0438 | 53738 | 53835.25 | 53850.7 | 53853 | 53888.1 | 53860.5 | 53891.4 | 53839 | 53850.65 | 53870.89 | 53892.84 | 53867.44 | 53919 | 53840 |
| 344.1793 | 53763.83 | 53824.6 | 53880.65 | 53845.11 | 53893.3 | 53875.4 | 53905.45 | 53829.83 | 53866.25 | 53895.22 | 53873.37 | 53849.17 | 53860.67 | 53896 |
| 344.3149 | 53821 | 53804.7 | 53865.6 | 53869.05 | 53877.15 | 53872.55 | 53894.95 | 53837 | 53874.1 | 53940.39 | 53933.89 | 53844.17 | 53862 | 53929 |
| 344.4504 | 53746.17 | 53795.4 | 53852.3 | 53864.84 | 53876.6 | 53869.45 | 53866.6 | 53831.48 | 53851.8 | 53909.39 | 53908.58 | 53857.83 | 53847.83 | 53964 |
| 344.586 | 53733.33 | 53799.2 | 53876.45 | 53852.11 | 53851.75 | 53890.8 | 53935.6 | 53841.83 | 53896.15 | 53942.5 | 53902.53 | 53878.28 | 53837 | 53789 |
| 344.7216 | 53760.17 | 53807.35 | 53856.25 | 53849.16 | 53863.5 | 53915.55 | 53953.15 | 53845.98 | 53881.65 | 53900.44 | 53939.05 | 53933.22 | 53846 | 53789 |
| 344.8572 | 53794 | 53814.6 | 53861.8 | 53876.84 | 53852.3 | 53896.2 | 53915.15 | 53872.98 | 53906.15 | 53941.28 | 53905.32 | 53915.61 | 53928.83 | 53798 |
| 344.9928 | 53778.17 | 53845.55 | 53861.2 | 53893.21 | 53908.8 | 53884.15 | 53904.15 | 53867.63 | 53915.95 | 53873.56 | 53907.42 | 53901.56 | 53902.67 | 53802 |
| 345.1284 | 53746.17 | 53920.9 | 53846.45 | 53867.42 | 53875.75 | 53857.65 | 53879.3 | 53834.5 | 53903.95 | 53867.78 | 53893.26 | 53872.72 | 53874.83 | 53985 |
| 345.264 | 53759.67 | 53844.75 | 53855.55 | 53853.16 | 53875.55 | 53873.55 | 53883.35 | 53811.38 | 53891.2 | 53902.94 | 53907.53 | 53854.56 | 53876.83 | 53824 |
| 345.3996 | 53797.67 | 53818.75 | 53875.25 | 53851.74 | 53915.3 | 53873.05 | 53920.15 | 53850.7 | 53898.25 | 53937.56 | 53854.63 | 53869.94 | 53911 | 53791 |
| 345.5353 | 53786.5 | 53841.45 | 53880.8 | 53824.37 | 53876.15 | 53863.6 | 53904.1 | 53875.6 | 53859.05 | 53891.11 | 53884.84 | 53914.44 | 53918.17 | 53788 |
| 345.6709 | 53823.5 | 53870.95 | 53835.15 | 53822.89 | 53834.85 | 53873.7 | 53864.9 | 53861.83 | 53847.45 | 53889.28 | 53877 | 53897.94 | 53898.83 | 53803 |
| 345.8066 | 53813.83 | 53849.3 | 53844.8 | 53804.37 | 53863.1 | 53888.7 | 53921 | 53871.18 | 53878.45 | 53896.06 | 53893.47 | 53857.11 | 53949.83 | 53790 |
| 345.9422 | 53869.33 | 53856.1 | 53875.6 | 53820 | 53874.35 | 53868.75 | 53893.3 | 53841.83 | 53840.6 | 53873.89 | 53893.32 | 53863 | 53888 | 53791 |
| 346.0779 | 53825 | 53827.25 | 53884.7 | 53820.95 | 53911.35 | 53901.6 | 53907.25 | 53847.45 | 53876.45 | 53880.83 | 53867.21 | 53893.83 | 53844 | 53771 |
| 346.2136 | 53785 | 53830.1 | 53867.75 | 53914.63 | 53870.5 | 53927.35 | 53899.8 | 53841.78 | 53903.15 | 53867.5 | 53859.53 | 53866.28 | 53857.5 | 53778 |
| 346.3492 | 53812.67 | 53832.65 | 53858.3 | 53846.05 | 53894.6 | 53910.45 | 53920.45 | 53833.63 | 53845.1 | 53886.11 | 53855.53 | 53861.61 | 53853.83 | 53763 |
| 346.485 | 53797.17 | 53814.85 | 53845.15 | 53834.11 | 53891 | 53897.1 | 53923.6 | 53865.9 | 53855.85 | 53879.72 | 53852.11 | 53897.78 | 53899.83 | 53783 |
| 346.6207 | 53763 | 53818.65 | 53842.7 | 53873.74 | 53885.75 | 53915.85 | 53889.9 | 53852.9 | 53889.3 | 53906.11 | 53894.84 | 53895.06 | 53846.17 | 53779 |
| 346.7564 | 53787.67 | 53802.9 | 53855.5 | 53843.63 | 53846.6 | 53885.25 | 53873.65 | 53804.7 | 53899.75 | 53893.5 | 53922.26 | 53842.17 | 53851.17 | 53768 |
| 346.8921 | 53749.67 | 53804.05 | 53900.55 | 53848.37 | 53879.95 | 53874.95 | 53938.65 | 53820.33 | 53861.6 | 53911.39 | 53907.79 | 53835.78 | 53881 | 53803 |
| 347.0278 | 53788.17 | 53778.6 | 53883.95 | 53828.26 | 53844.6 | 53856.15 | 53879.8 | 53840.9 | 53839.1 | 53865.28 | 53878.37 | 53840.56 | 53864.83 | 53927 |
| 347.1636 | 53847.33 | 53786.2 | 53850.55 | 53857.05 | 53863.3 | 53838.6 | 53889.5 | 53853.25 | 53828.45 | 53876.78 | 53891.21 | 53889.39 | 53861 | 53820 |
| 347.2994 | 53850.67 | 53814.8 | 53844.95 | 53846.32 | 53913 | 53850.6 | 53878.85 | 53820.45 | 53833 | 53892.17 | 53881.63 | 53861.22 | 53877.67 | 53886 |
| 347.4351 | 53808.67 | 53797.75 | 53874.6 | 53818.42 | 53856.95 | 53868.9 | 53895.55 | 53801.55 | 53885.55 | 53854.56 | 53906.42 | 53838.39 | 53830.33 | 53845 |
| 347.5709 | 53811.67 | 53819.1 | 53858.55 | 53846.26 | 53880.05 | 53885.1 | 53932.7 | 53843.73 | 53929.2 | 53900.28 | 53919.37 | 53858.78 | 53869.33 | 53846 |
| 347.7066 | 53836 | 53809.7 | 53859.4 | 53852.89 | 53882.2 | 53875.2 | 53887.15 | 53820.03 | 53880.45 | 53891.72 | 53888.11 | 53867 | 53905.67 | 53782 |
| 347.8424 | 53810.17 | 53810.2 | 53881.65 | 53838.79 | 53913.6 | 53871.1 | 53891.5 | 53847.15 | 53852.25 | 53898.72 | 53917.58 | 53857.78 | 53842.17 | 53784 |
| 347.9782 | 53788.67 | 53830.1 | 53862.75 | 53869.16 | 53852.6 | 53877.35 | 53903.25 | 53833.28 | 53867.65 | 53918.89 | 53873 | 53866.72 | 53846.83 | 53877 |
| 348.114 | 53758.67 | 53794.95 | 53891.75 | 53843.68 | 53862.5 | 53880.25 | 53851.55 | 53817.13 | 53855.4 | 53921.44 | 53858.58 | 53884.28 | 53847.33 | 53841 |
| 348.2499 | 53765 | 53832.7 | 53858.3 | 53818.79 | 53915.1 | 53884.4 | 53875.25 | 53826.3 | 53861.85 | 53899.78 | 53868.53 | 53900.89 | 53875.83 | 53784 |
| 348.3857 | 53849.67 | 53800.7 | 53865.05 | 53844 | 53879.5 | 53933.65 | 53922.05 | 53819.18 | 53844.8 | 53930.67 | 53898.63 | 53907.28 | 53878.83 | 53801 |
| 348.5215 | 53771.17 | 53794.55 | 53908.8 | 53818.16 | 53883.5 | 53934.05 | 53888.4 | 53837.05 | 53874.75 | 53935.28 | 53865 | 53857.5 | 53903.5 | 53764 |
| 348.6574 | 53832.83 | 53814.45 | 53874.65 | 53830.32 | 53890.6 | 53903.7 | 53890.75 | 53852.45 | 53870.5 | 53907.5 | 53874.11 | 53882.22 | 53944.33 | 53762 |
| 348.7932 | 53765.33 | 53847.9 | 53871.75 | 53802.68 | 53870.55 | 53851.3 | 53869.45 | 53815.9 | 53853 | 53910.56 | 53880 | 53857.67 | 53957.5 | 53782 |
| 348.9291 | 53824.83 | 53789.4 | 53835.35 | 53812.79 | 53849.15 | 53867.05 | 53856.95 | 53836.6 | 53833.35 | 53916.89 | 53877.16 | 53886.67 | 53859.5 | 53794 |
| 349.0649 | 53770.83 | 53791.8 | 53827.45 | 53845.16 | 53904 | 53821.9 | 53855.85 | 53831.78 | 53826.25 | 53919.28 | 53867 | 53864.67 | 53850.67 | 54076 |
| 349.2008 | 53739.83 | 53787.9 | 53825.25 | 53823.05 | 53827 | 53827.35 | 53867.7 | 53820.83 | 53867 | 53845 | 53873.53 | 53875.72 | 53901.33 | 53837 |
| 349.3367 | 53730.67 | 53833.1 | 53843.3 | 53873.05 | 53814.65 | 53905.8 | 53864.8 | 53827.95 | 53925.65 | 53870.44 | 53909.95 | 53897.44 | 53919.67 | 53769 |
| 349.4726 | 53759.67 | 53828 | 53835.8 | 53857.84 | 53839.3 | 53871.95 | 53869.75 | 53822.23 | 53903.05 | 53872.11 | 53917.63 | 53877 | 53924.5 | 53757 |
| 349.6085 | 53798 | 53796.85 | 53823.7 | 53868.16 | 53886.55 | 53926.4 | 53898.4 | 53814.88 | 53854.5 | 53866.56 | 53860.05 | 53949.72 | 53858 | 53758 |
| 349.7444 | 53754.67 | 53812.45 | 53826 | 53846.42 | 53892.7 | 53874.1 | 53913.05 | 53862.43 | 53822.2 | 53874.56 | 53842.16 | 53941.39 | 53908.17 | 53758 |
| 349.8803 | 53759.67 | 53812.65 | 53836 | 53872.47 | 53852.7 | 53840.5 | 53894.15 | 53885.03 | 53824.75 | 53928.22 | 53859.84 | 53883.83 | 53848.83 | 53767 |
| 350.0163 | 53761.5 | 53810.6 | 53846.35 | 53842.89 | 53852.05 | 53860.8 | 53900.4 | 53840.78 | 53826.1 | 53920.33 | 53859.32 | 53922.06 | 53857.17 | 53758 |
| 350.1522 | 53725.5 | 53835.65 | 53872.85 | 53864.68 | 53878.3 | 53867.1 | 53886.7 | 53837.18 | 53846.85 | 53886.67 | 53877.47 | 53884.5 | 53900.5 | 53759 |
| 350.2882 | 53767.67 | 53830.75 | 53891.8 | 53856.11 | 53855.25 | 53854.65 | 53921.95 | 53842.93 | 53828.9 | 53891.33 | 53846.53 | 53893.72 | 53853.17 | 53771 |
| 350.4241 | 53729.17 | 53810.75 | 53869.05 | 53833.84 | 53841.75 | 53837.6 | 53887.85 | 53841.1 | 53838.95 | 53915.61 | 53851.68 | 53852.22 | 53845.83 | 53767 |
| 350.5601 | 53776 | 53858.35 | 53884.15 | 53814.74 | 53837.45 | 53872.5 | 53866.3 | 53830.05 | 53853.25 | 53892.17 | 53855.21 | 53851.44 | 53855.17 | 53761 |
| 350.696 | 53816.17 | 53824.5 | 53846 | 53831.42 | 53852.05 | 53875.9 | 53890.25 | 53813.83 | 53834.05 | 53886.83 | 53879.16 | 53851.61 | 53845.67 | 53760 |
| 350.832 | 53755.83 | 53783.35 | 53824.05 | 53847.05 | 53838.15 | 53933.95 | 53891.05 | 53811.3 | 53868.7 | 53888.5 | 53832 | 53880.5 | 53842.33 | 53747 |
| 350.968 | 53780.83 | 53807.9 | 53877.9 | 53869.84 | 53864.15 | 53897 | 53920 | 53801.83 | 53834 | 53868.5 | 53850.47 | 53856.61 | 53844.17 | 53770 |
| 351.104 | 53890.17 | 53807.6 | 53860.9 | 53865.84 | 53873.3 | 53939.7 | 53885.9 | 53803.83 | 53846.15 | 53848.5 | 53847.58 | 53846.17 | 53854.33 | 53772 |
| 351.24 | 53791 | 53827.35 | 53920.65 | 53874.11 | 53867.05 | 53944.2 | 53919.55 | 53835.08 | 53893.15 | 53902.67 | 53892 | 53880.61 | 53900.83 | 53853 |
| 351.376 | 53762.67 | 53818.25 | 53868.2 | 53853.26 | 53855.4 | 53903.45 | 53868.35 | 53806.93 | 53858.45 | 53913.72 | 53935.05 | 53915.89 | 53826.5 | 53942 |
| 351.5121 | 53749.33 | 53825.95 | 53863.65 | 53841.79 | 53822.95 | 53902.05 | 53837.9 | 53820.6 | 53859.05 | 53884.72 | 53890.11 | 53904.28 | 53873 | 53888 |
| 351.6481 | 53779.33 | 53807.55 | 53900.6 | 53821.79 | 53835.85 | 53926.3 | 53873.6 | 53829.53 | 53853.05 | 53886.06 | 53936.58 | 53917.28 | 53870 | 53794 |
| 351.7841 | 53832 | 53772.2 | 53865.35 | 53818.79 | 53842.25 | 53907.1 | 53897.9 | 53815.35 | 53841.65 | 53881.89 | 53890.68 | 53880.67 | 53845.67 | 53816 |
| 351.9202 | 53833.17 | 53794.3 | 53859.35 | 53846.84 | 53874.45 | 53951.95 | 53907.65 | 53813.45 | 53831.45 | 53877.06 | 53910.42 | 53878.83 | 53828.67 | 53946 |
| 352.0562 | 53773.17 | 53808.75 | 53858.5 | 53814.68 | 53872.5 | 53926.45 | 53911.65 | 53810.28 | 53833.05 | 53874.78 | 53877.84 | 53877.78 | 53819.33 | 53881 |
| 352.1923 | 53827.33 | 53791.65 | 53889.65 | 53848.11 | 53859.7 | 53965.1 | 53935.2 | 53810.45 | 53839.75 | 53897 | 53894.42 | 53870.89 | 53847.67 | 53810 |
| 352.3283 | 53744 | 53870.05 | 53906.95 | 53900.74 | 53893.75 | 53893.7 | 53913.55 | 53853.73 | 53849.25 | 53897.61 | 53910 | 53851.06 | 53847.33 | 53809 |
| 352.4644 | 53778.5 | 53812.2 | 53847.3 | 53878.47 | 53882.5 | 53877.45 | 53884.25 | 53826.13 | 53841.9 | 53925.22 | 53911.47 | 53847.56 | 53884 | 53972 |
| 352.6005 | 53773.17 | 53827.4 | 53840.7 | 53825.58 | 53914.75 | 53881.3 | 53858.75 | 53843.98 | 53854.9 | 53936.78 | 53891.21 | 53874.11 | 53922.83 | 53910 |
| 352.7366 | 53778.67 | 53787.35 | 53850.2 | 53832.63 | 53894.3 | 53856.15 | 53879.95 | 53837.03 | 53864.05 | 53913.83 | 53887.74 | 53865.22 | 53897.83 | 53765 |
| 352.8727 | 53772.67 | 53797.8 | 53836.25 | 53883.05 | 53879.5 | 53843.6 | 53902.55 | 53843.75 | 53868.75 | 53887.72 | 53880.11 | 53830.5 | 53840.83 | 53770 |
| 353.0088 | 53752.17 | 53806.45 | 53867.3 | 53838.05 | 53873 | 53863.65 | 53903.95 | 53871.68 | 53829.95 | 53858.78 | 53883.84 | 53831.22 | 53840.67 | 53768 |
| 353.1449 | 53798.17 | 53831.65 | 53920.65 | 53841.53 | 53880.5 | 53906.1 | 53901.1 | 53852.18 | 53842.6 | 53865.22 | 53910 | 53847.5 | 53883.83 | 53849 |
| 353.2811 | 53764.67 | 53811.6 | 53900.65 | 53864.42 | 53887.4 | 53902.45 | 53913.85 | 53832.43 | 53811.1 | 53887.28 | 53915.47 | 53839.72 | 53881.33 | 53775 |
| 353.4172 | 53769 | 53809.3 | 53865 | 53866.63 | 53875.55 | 53878.5 | 53892.25 | 53811.7 | 53815.9 | 53860.5 | 53912.16 | 53866.89 | 53898 | 53778 |
| 353.5533 | 53736.67 | 53792.5 | 53871.8 | 53871.26 | 53903.25 | 53884.05 | 53887.6 | 53852.8 | 53817.1 | 53903.83 | 53880.37 | 53855.44 | 53850.83 | 53776 |
| 353.6895 | 53843.83 | 53812.15 | 53859.5 | 53851.16 | 53871.45 | 53913.5 | 53871 | 53854.7 | 53835.95 | 53853.67 | 53909.95 | 53843.89 | 53822.83 | 53855 |
| 353.8257 | 53787.17 | 53831.1 | 53853.05 | 53844.32 | 53891.95 | 53884.8 | 53950.15 | 53848.85 | 53818.6 | 53848 | 53954.63 | 53881.67 | 53822.83 | 53857 |
| 353.9618 | 53765.67 | 53829.6 | 53858.8 | 53827.95 | 53878 | 53867.75 | 53953.3 | 53853.18 | 53823.95 | 53883.72 | 53936.68 | 53951.61 | 53840.17 | 53775 |
| 354.098 | 53746.17 | 53809.65 | 53905.45 | 53886.84 | 53899.15 | 53861.15 | 53862.4 | 53834.53 | 53852.7 | 53871.94 | 53952.79 | 53911.89 | 53831.67 | 53756 |
| 354.2342 | 53757 | 53820.3 | 53959.8 | 53864.68 | 53864 | 53871.85 | 53869.55 | 53850.15 | 53839.3 | 53895.11 | 53896.79 | 53889 | 53855.83 | 53754 |
| 354.3704 | 53779.67 | 53804.2 | 53906.85 | 53828.89 | 53844.8 | 53924.45 | 53914.95 | 53820.6 | 53880.6 | 53878.33 | 53928.84 | 53880.11 | 53862.17 | 53789 |
| 354.5066 | 53848.67 | 53852.25 | 53930.95 | 53924 | 53876.25 | 53922.8 | 53896.35 | 53843.73 | 53909.95 | 53929.22 | 53893.68 | 53920.39 | 53858.5 | 53801 |
| 354.6428 | 53765 | 53836.45 | 53988.35 | 53910.84 | 53898.5 | 53930.15 | 53857.95 | 53843.55 | 53838.7 | 53891.72 | 53946.79 | 53873.06 | 53922.5 | 53767 |
| 354.779 | 53768.67 | 53841.55 | 53929.8 | 53860.16 | 53914.3 | 53922.8 | 53879.05 | 53820.3 | 53847.9 | 53879.11 | 53908.63 | 53908.72 | 53974.67 | 53759 |
| 354.9152 | 53806.33 | 53825.1 | 53899.15 | 53824.26 | 53927.85 | 53869.2 | 53865.25 | 53820.25 | 53838.55 | 53838.89 | 53919.16 | 53903.44 | 53956.33 | 53772 |
| 355.0514 | 53823.17 | 53831.8 | 53921.65 | 53835.53 | 53911.05 | 53867.6 | 53857.5 | 53828.3 | 53849.85 | 53854.89 | 53911.89 | 53912.78 | 53905 | 53791 |
| 355.1876 | 53818.5 | 53854.8 | 53880.8 | 53812.37 | 53875.35 | 53906.8 | 53887.7 | 53836.6 | 53836.9 | 53853.44 | 53938.11 | 53909.33 | 53920.33 | 53872 |
| 355.3239 | 53757.5 | 53835.65 | 53920.4 | 53845.95 | 53833.25 | 53885.8 | 53899.55 | 53825.3 | 53887.1 | 53901.56 | 53920.05 | 53955.5 | 53871.5 | 53819 |
| 355.4601 | 53720.33 | 53847.7 | 53966.7 | 53849.95 | 53854.35 | 53852.9 | 53906.4 | 53823.23 | 53872.85 | 53886.22 | 53900.32 | 53938.89 | 54027 | 53924 |
| 355.5964 | 53746 | 53833.45 | 53935.55 | 53858.32 | 53852.65 | 53888.25 | 53927.3 | 53844.95 | 53885.25 | 53873.5 | 53905.68 | 53881.5 | 53956 | 54390 |
| 355.7326 | 53782.67 | 53844.7 | 53914.25 | 53853.95 | 53867.4 | 53874.15 | 53910.2 | 53841.38 | 53827.45 | 53880.56 | 53930.89 | 53857.17 | 53882.5 | 53874 |
| 355.8689 | 53768.5 | 53822.7 | 53902.1 | 53847.05 | 53920.25 | 53891.85 | 53870.4 | 53847.8 | 53824.9 | 53909 | 53925.68 | 53882.22 | 53888.33 | 53936 |
| 356.0052 | 53816.67 | 53845.5 | 53915.4 | 53900.95 | 53881.75 | 53901.35 | 53893.75 | 53834.73 | 53853.45 | 53876.06 | 53893 | 53877.33 | 53935.67 | 53941 |
| 356.1415 | 53813.17 | 53843.5 | 53902.8 | 53880.74 | 53892.8 | 53868.05 | 53949.55 | 53866.73 | 53819.9 | 53884.44 | 53847.47 | 53883.28 | 53970.83 | 53883 |
| 356.2778 | 53739.5 | 53794.9 | 53891.55 | 53872.63 | 53896.65 | 53870.1 | 53952.25 | 53863.9 | 53819.25 | 53899.22 | 53910.84 | 53891.28 | 53920.33 | 53776 |
| 356.4141 | 53795.17 | 53797.9 | 53871.4 | 53854.95 | 53852.35 | 53899.9 | 53935.35 | 53871.8 | 53839.15 | 53882.5 | 53958.95 | 53928.39 | 53861.33 | 53792 |
| 356.5504 | 53899.5 | 53867.7 | 53888.55 | 53850.63 | 53881.85 | 53971.25 | 53915.9 | 53882.63 | 53848.75 | 53869.22 | 53893.26 | 53906.83 | 53911.67 | 53816 |
| 356.6867 | 53819.67 | 53907.05 | 53911.65 | 53897.05 | 53904.85 | 53907.55 | 53884.5 | 53895.8 | 53858.1 | 53862.44 | 53873.21 | 53912.5 | 53844.67 | 53769 |
| 356.823 | 53852.67 | 53923.35 | 53904.35 | 53844.84 | 53912.35 | 53912.8 | 53867.3 | 53845.93 | 53858.6 | 53859.83 | 53938.16 | 53902.22 | 53867.17 | 53765 |
| 356.9594 | 53831.67 | 53863.85 | 53880.65 | 53865.63 | 53904.8 | 53920.75 | 53898.5 | 53832.15 | 53899.05 | 53859.78 | 53969.89 | 53877.06 | 53882.67 | 53790 |
| 357.0957 | 53792.33 | 53869.5 | 53924.5 | 53855.84 | 53913.05 | 53892.9 | 53888.05 | 53823.95 | 53887.4 | 53857.39 | 53929.32 | 53929.89 | 53865.33 | 53880 |
| 357.232 | 53880.83 | 53875.65 | 53916.1 | 53846.63 | 53924.85 | 53873.3 | 53871.55 | 53826.9 | 53851.85 | 53869.72 | 53889.89 | 53915.11 | 53840.5 | 53826 |
| 357.3684 | 53854.33 | 53861 | 53892 | 53857 | 53947.95 | 53873.85 | 53893.15 | 53852.48 | 53878.1 | 53882.39 | 53900.53 | 53896.06 | 53861.67 | 53768 |
| 357.5047 | 53874 | 53843.8 | 53912.5 | 53896.84 | 53920.7 | 53877.25 | 53916.15 | 53851.58 | 53831.4 | 53875.39 | 53929.21 | 53845.28 | 53844.67 | 53795 |
| 357.6411 | 53844.33 | 53852.35 | 53955.3 | 53919.26 | 53939.1 | 53910 | 53945.15 | 53840.65 | 53851 | 53873.44 | 53877.68 | 53836.11 | 53862.17 | 53775 |
| 357.7775 | 53803.33 | 53870.25 | 53945.05 | 53886.42 | 53922.4 | 53899.2 | 53918.3 | 53852.25 | 53891.95 | 53860.94 | 53872.42 | 53912 | 53871.17 | 53809 |
| 357.9139 | 53796 | 53877.65 | 53944.2 | 53904.95 | 53899.65 | 53871 | 53964.15 | 53898.5 | 53924.75 | 53870.39 | 53915.53 | 53881.22 | 53936.67 | 53831 |
| 358.0502 | 53844.67 | 53884.05 | 53972.45 | 53919.32 | 53908.55 | 53894.75 | 53919.85 | 53914.05 | 53891.95 | 53886.83 | 53921.32 | 53868.22 | 53881.17 | 53842 |
| 358.1866 | 53753.17 | 53832.25 | 53982.3 | 53920.26 | 53885.55 | 53887.7 | 53880.85 | 53917.4 | 53880.4 | 53886.28 | 53866.16 | 53864.83 | 53876 | 53757 |
| 358.323 | 53817.17 | 53858.05 | 53963 | 53924.47 | 53928.65 | 53908 | 54005.2 | 53890.98 | 53899.05 | 53845.83 | 53879.16 | 53871.78 | 53951.17 | 53759 |
| 358.4594 | 53877.67 | 53905 | 53973.35 | 53945.58 | 53936.5 | 53872.15 | 53943.55 | 53892.73 | 53926.9 | 53893.83 | 53897.26 | 53859.72 | 53909.17 | 53739 |
| 358.5958 | 53805.17 | 53922.25 | 53996.5 | 53941.58 | 53882 | 53916.4 | 53979.3 | 53871.13 | 53908.15 | 53940.33 | 53930.05 | 53858.5 | 53866.5 | 53736 |
| 358.7322 | 53777.83 | 53908.5 | 54009.35 | 53883.89 | 53931.2 | 53920.5 | 53968.25 | 53859.58 | 53855.15 | 53926.06 | 53910.37 | 53854.44 | 53898.17 | 53759 |
| 358.8687 | 53837.17 | 53914.95 | 53963.55 | 53863.95 | 53922.25 | 53892.3 | 53916.85 | 53842.43 | 53891.35 | 53917 | 53881.42 | 53853.17 | 53852.67 | 53770 |
| 359.0051 | 53861.67 | 53906.1 | 53996.65 | 53895.37 | 53921.05 | 53900.9 | 53979.55 | 53869.33 | 53942.6 | 53890.56 | 53870.84 | 53843.11 | 53880.67 | 53962 |
| 359.1415 | 53781.33 | 53882.2 | 53956.9 | 53945.68 | 53905.5 | 53922.95 | 54053.6 | 53868.13 | 53973.45 | 53872.22 | 53909.21 | 53851.22 | 53847.83 | 53890 |
| 359.278 | 53885.67 | 53883.95 | 53956.45 | 53930.21 | 53895.05 | 53889.9 | 53971.2 | 53821.93 | 53932.25 | 53926.44 | 53889.32 | 53879.78 | 53860.67 | 53843 |
| 359.4144 | 53869.17 | 53955.75 | 54000.45 | 53979.16 | 53952.6 | 53907.7 | 53998.65 | 53857.88 | 53913.3 | 53972.56 | 53910 | 53871.17 | 53942.17 | 53861 |
| 359.5509 | 53846.83 | 53933.6 | 53945.05 | 53928.84 | 54026.05 | 53931.6 | 53942.8 | 53821.3 | 53932.3 | 53957.83 | 53917.37 | 53869.94 | 53909.67 | 53820 |
| 359.6874 | 53887.5 | 53887.65 | 53911.9 | 54018.53 | 53991.35 | 53910.85 | 53957.9 | 53850.63 | 53928 | 53917.33 | 53946.26 | 53863.33 | 53939.17 | 54025 |
| 359.8238 | 53845 | 53896.2 | 53960.9 | 53946.37 | 53951.75 | 53932.45 | 53982.2 | 53884.83 | 53941.2 | 53938 | 53920.47 | 53874.44 | 53926 | 53895 |
| 359.9603 | 53810.17 | 53901.95 | 53925.15 | 53952.74 | 54036.5 | 53912.6 | 53992.9 | 53868.48 | 53930.95 | 53993.17 | 53915.26 | 53890.56 | 53943.33 | 53798 |
| 360.0968 | 53819 | 53958.45 | 53932.5 | 53953.68 | 53937.95 | 53905.3 | 54011.1 | 53895.83 | 53892.1 | 53957.22 | 53919.68 | 53898.67 | 53835.33 | 53866 |
| 360.2333 | 53961.5 | 53987.8 | 53978.15 | 53976.84 | 54016.75 | 53930.6 | 54018.05 | 53875.68 | 53877.55 | 53889.22 | 53890.74 | 53908 | 53830.17 | 53878 |
| 360.3698 | 53888.17 | 53961.85 | 53977.9 | 54003.16 | 54017.1 | 53988.85 | 53962.85 | 53896.28 | 53882.35 | 53869.78 | 53998.58 | 53889.83 | 53854.17 | 53781 |
| 360.5063 | 53844.5 | 53994.95 | 54083.55 | 53951.47 | 54051.5 | 54051.8 | 53956.35 | 53902.48 | 53926.45 | 53891.06 | 53934.26 | 53886.39 | 53957.17 | 53784 |
| 360.6428 | 53790.83 | 53990.35 | 54065.6 | 53949.79 | 54012.55 | 54031.55 | 53990.95 | 53876.53 | 53894.9 | 53909.83 | 53911.89 | 53868.11 | 53926.17 | 53745 |
| 360.7793 | 53840.33 | 53940.95 | 54029.9 | 53983.11 | 54005.2 | 53934.35 | 54014.45 | 53852.53 | 53940.35 | 53985.17 | 53899.84 | 53918.44 | 53867.33 | 53747 |
| 360.9159 | 53828.83 | 53900.5 | 54076.6 | 53952.68 | 53941.85 | 53967.6 | 53952.25 | 53904.48 | 54008.75 | 54010.17 | 53914.21 | 53877.78 | 53912.83 | 53763 |
| 361.0524 | 53811.17 | 53888.2 | 54091.2 | 53922.53 | 53907.45 | 53910.35 | 53960.75 | 53888 | 53997.65 | 53958.22 | 53966.21 | 53870.83 | 53871.17 | 53833 |
| 361.1889 | 53792.17 | 53939.95 | 54006.5 | 53993.53 | 53941.75 | 53956.6 | 54017.65 | 53954.13 | 53956.45 | 53913.5 | 53907.42 | 53888.44 | 53890 | 53867 |
| 361.3255 | 53809.83 | 53947.5 | 54022.85 | 53951.89 | 53956.2 | 53965.1 | 54002.7 | 53898 | 53976.6 | 53948.28 | 53971.21 | 53884.22 | 53823.67 | 53915 |
| 361.462 | 53890.17 | 53940.55 | 53986.75 | 53968 | 53985.85 | 53935.5 | 53996.6 | 53897.68 | 53992.4 | 53890.17 | 53986.21 | 53918.06 | 53832.17 | 53824 |
| 361.5986 | 53908.17 | 53954.95 | 54079.45 | 53976.37 | 53939.1 | 53945.95 | 54109.7 | 53911.6 | 53982.8 | 53954.44 | 53963.16 | 53939.33 | 53847.17 | 53911 |
| 361.7351 | 53962.67 | 53936.95 | 53990.7 | 53928.74 | 53955.05 | 53954.55 | 54058.8 | 53945.05 | 54017.25 | 53999.72 | 53941.68 | 53937.06 | 53874.33 | 53732 |
| 361.8717 | 54003.17 | 53962.6 | 53995.35 | 53975.84 | 53971.1 | 53978.2 | 54060.8 | 53909.78 | 54000.05 | 53993.17 | 53919.63 | 53922.61 | 53879.5 | 53742 |
| 362.0083 | 53956.17 | 53918.7 | 54015.7 | 53969.11 | 54075.35 | 53973.6 | 54026.45 | 53870.3 | 53909.5 | 54013.11 | 53885.84 | 53932.56 | 53840.33 | 53753 |
| 362.1448 | 53966.5 | 53907.9 | 54078.2 | 54050.53 | 54078.5 | 54031.1 | 53979.65 | 53876.13 | 53925.4 | 53957.39 | 53914.58 | 53883.83 | 53851.33 | 53764 |
| 362.2814 | 53888.17 | 53899.95 | 54011.4 | 54057.21 | 54024.15 | 54003 | 54028.55 | 53897.8 | 54011.85 | 53930.94 | 53929.32 | 53877.67 | 53880.17 | 53804 |
| 362.418 | 53811 | 53938.25 | 54055.25 | 54042.79 | 54058.1 | 54000.75 | 54066 | 53927.33 | 54020.05 | 53978.94 | 53985.68 | 53939.06 | 53945.17 | 53774 |
| 362.5546 | 53804.17 | 53938.3 | 53998.8 | 54101.84 | 53918.3 | 53958.7 | 53986.1 | 53885.28 | 53970.1 | 53992.89 | 53955.16 | 53861.44 | 53994.83 | 53740 |
| 362.6912 | 53951.17 | 53969.1 | 54014.55 | 54066.89 | 53939.3 | 53963.95 | 54066.4 | 53878.98 | 53998.65 | 53980.94 | 53923.63 | 53872.89 | 54034.5 | 53759 |
| 362.8279 | 53963.5 | 53937.4 | 54048.1 | 54077.11 | 54055 | 54032.05 | 54181.3 | 53916.95 | 54001.6 | 53991.72 | 53921.16 | 53918.56 | 53882.83 | 53841 |
| 362.9645 | 53892.33 | 53975 | 54131.65 | 54108.74 | 54104.95 | 54115.65 | 54077.85 | 53929.68 | 53990.4 | 53992.83 | 53960.84 | 53898.06 | 54000.33 | 53937 |
| 363.1011 | 53940.33 | 54042.35 | 54184.1 | 54164.42 | 54032.8 | 54079 | 54032.85 | 53965.4 | 53988.25 | 53996.56 | 53985.26 | 53923.61 | 53954 | 53750 |
| 363.2377 | 53929.83 | 53958 | 54269.25 | 54050.89 | 54059.1 | 54078.6 | 54041.05 | 53916.98 | 54026.45 | 54030.17 | 53994.32 | 53978.56 | 53866.67 | 53755 |
| 363.3743 | 53849.5 | 53999 | 54229.75 | 54045.74 | 54090.65 | 54085.1 | 54084.9 | 53950.58 | 53993.25 | 53990.44 | 53967.53 | 53970.28 | 53831.67 | 53858 |
| 363.511 | 53854.67 | 54023.7 | 54172 | 54050.16 | 54155.9 | 53988.25 | 54128.75 | 53950.33 | 53999.85 | 54054.78 | 53933.21 | 53964.44 | 53872.17 | 53834 |
| 363.6476 | 53900.83 | 54121.65 | 54207.75 | 54057.47 | 54147.75 | 54067 | 54113.7 | 53973.4 | 54010.05 | 54043.56 | 53995.68 | 53943.94 | 53917.33 | 53846 |
| 363.7843 | 53972.83 | 54106.05 | 54205.55 | 54161.32 | 54074.65 | 54188.65 | 54092.05 | 54033.8 | 54055.2 | 53984.06 | 54030.11 | 53943.56 | 53872.67 | 53723 |
| 363.9209 | 53934.33 | 54068.45 | 54275.65 | 54115.79 | 54097.5 | 54178 | 54260.7 | 53984.9 | 54079.85 | 54030.72 | 54012.53 | 53950.67 | 53897.5 | 53733 |
| 364.0576 | 54167.5 | 54102.75 | 54200.55 | 54078.58 | 54111.95 | 54140.15 | 54286.1 | 53921.38 | 54072.65 | 53970.06 | 53930.05 | 53962.17 | 53853.67 | 53754 |
| 364.1943 | 54023.33 | 54114.25 | 54195.1 | 54063.84 | 54117.45 | 54085.8 | 54197.6 | 53983.65 | 53995.75 | 53998.28 | 53942.68 | 53965.33 | 53842.5 | 53796 |
| 364.3309 | 54216 | 54124.6 | 54186.05 | 54171.37 | 54082.7 | 54057.4 | 54132.85 | 53926.83 | 54000.15 | 53950.67 | 53944.89 | 54062 | 53816.17 | 53843 |
| 364.4676 | 54271.83 | 54120.75 | 54298.65 | 54187.68 | 54010.55 | 54064.45 | 54060.75 | 53929.38 | 53985.4 | 53940.11 | 53913.74 | 54062.39 | 53841.33 | 53889 |
| 364.6043 | 54160.17 | 54144.2 | 54199.75 | 54178.32 | 54078.7 | 54167.9 | 54174.3 | 53979.43 | 54088.05 | 53983.06 | 53958.95 | 54001.94 | 53937.5 | 53851 |
| 364.741 | 54140.33 | 54069.95 | 54083.85 | 54161.47 | 54019.7 | 54154.6 | 54223.75 | 53972.18 | 54088.65 | 54066.78 | 54007.63 | 53968.67 | 53853.83 | 53743 |
| 364.8777 | 54041.83 | 54117.65 | 54128.3 | 54135.37 | 54062 | 54055.55 | 54096.9 | 53942.65 | 54107.4 | 54002.56 | 53957.74 | 53977.33 | 53874 | 53736 |
| 365.0144 | 53978.33 | 54130.6 | 54129.3 | 54197 | 54119.2 | 54137.6 | 54086.9 | 54014.48 | 54082.3 | 54044.89 | 53958.16 | 53997.89 | 53934 | 53751 |
| 365.1511 | 54041.17 | 54118.9 | 54075.6 | 54083.32 | 54061.7 | 54089.45 | 54167 | 53993.6 | 54047.6 | 54022.56 | 54005.47 | 54028.89 | 53892 | 53752 |
| 365.2878 | 53897.5 | 54123 | 54136.85 | 54070.16 | 54047.25 | 54068.5 | 54187.2 | 53950.75 | 54130.8 | 53990.11 | 53974.21 | 53973.61 | 53936 | 53758 |
| 365.4245 | 53923 | 54042.8 | 54104.1 | 54092.63 | 54075.15 | 54017.55 | 54110.15 | 53954.45 | 54124.15 | 53944.28 | 53908.37 | 53971.28 | 53909.33 | 53858 |
| 365.5613 | 53889.67 | 54043.45 | 54132.05 | 54045.37 | 54103.2 | 54075.6 | 54059.3 | 53972.38 | 54100.65 | 53978.83 | 53975.53 | 53958.33 | 53856.17 | 53854 |
| 365.698 | 53857.17 | 54035.35 | 54196.95 | 54080.21 | 54152.2 | 54011.2 | 54146.2 | 53941.75 | 54069.8 | 53999.94 | 53962.53 | 54040 | 53939.17 | 53812 |
| 365.8347 | 53843.67 | 54122 | 54101.5 | 54100.37 | 54065.7 | 54025.6 | 54154.8 | 53931.45 | 54047.45 | 53972 | 53936 | 53980.56 | 53868.67 | 53771 |
| 365.9715 | 53885.83 | 54136.3 | 54182.25 | 54123.68 | 54049.05 | 54113.8 | 54122.7 | 53918.48 | 54115.4 | 54026.61 | 53989.21 | 53917.56 | 53869.33 | 53956 |
| 366.1082 | 53894.33 | 54134 | 54205.4 | 54141.32 | 54055.65 | 54038.1 | 54210 | 53963.83 | 54035.65 | 54016.28 | 53967.89 | 53926.22 | 53824.67 | 53773 |
| 366.245 | 53923.83 | 54149.25 | 54174.35 | 54210.26 | 54087.9 | 54084.2 | 54158.15 | 53967.05 | 54043.4 | 54026.06 | 53890.05 | 53975.89 | 53881 | 53834 |
| 366.3817 | 54103.83 | 54178.6 | 54127.7 | 54165.32 | 54046.35 | 54190.35 | 54218.05 | 53951.68 | 54080.55 | 53958.11 | 53961.26 | 54069.39 | 53906.33 | 53982 |
| 366.5185 | 54036.67 | 54104.7 | 54143.9 | 54110.53 | 54098.45 | 54087.7 | 54208.4 | 53976.05 | 54118.8 | 53949.11 | 53941.16 | 54010.44 | 53909.17 | 53778 |
| 366.6552 | 53994.17 | 54071.9 | 54158.7 | 54255.58 | 54136.25 | 54051 | 54158.55 | 54034.1 | 54043.55 | 54027.83 | 53985.84 | 54017.67 | 53998.5 | 53804 |
| 366.792 | 53921.67 | 54077.35 | 54150.15 | 54174.95 | 54084.85 | 54110.1 | 54172.35 | 54045.58 | 54093.25 | 53977.78 | 54012.37 | 54023.78 | 54042.33 | 54156 |
| 366.9288 | 53917.33 | 54020.75 | 54116.3 | 54143.11 | 54212.6 | 54187.45 | 54148.9 | 54034.6 | 54076.5 | 53943.61 | 54049.47 | 53926.78 | 54130.67 | 53854 |
| 367.0656 | 54185.33 | 54137.45 | 54273.35 | 54116.79 | 54157.65 | 54182.95 | 54252.75 | 54006.23 | 54153.25 | 53976.67 | 54054.58 | 53935.39 | 53949 | 53832 |
| 367.2024 | 54056.67 | 54164.75 | 54339 | 54224.53 | 54145.55 | 54130.95 | 54247.1 | 54031.18 | 54161.55 | 54050.94 | 53988.42 | 53945.17 | 53885.33 | 53995 |
| 367.3392 | 53999.83 | 54073.6 | 54270.95 | 54276.79 | 54154.2 | 54228.45 | 54182.75 | 53987.95 | 54150 | 54025.11 | 54058.26 | 54003 | 53867.67 | 54184 |
| 367.476 | 53991.83 | 54148.65 | 54296.55 | 54208.11 | 54119.85 | 54172.45 | 54251.1 | 53987.65 | 54115.8 | 53998.67 | 54046.53 | 54036.17 | 53917.67 | 53789 |
| 367.6128 | 53847.17 | 54112.5 | 54260.3 | 54220.68 | 54190.15 | 54207.75 | 54234.55 | 53992.43 | 54128.2 | 53982.39 | 54112.47 | 53982.44 | 53878.17 | 53756 |
| 367.7496 | 54038.33 | 54122.65 | 54251.35 | 54235.74 | 54190.15 | 54246.4 | 54138.4 | 53972.13 | 54126.6 | 54012.11 | 54032.79 | 53994.56 | 53908.83 | 53778 |
| 367.8864 | 54136.83 | 54226.7 | 54228.55 | 54281.53 | 54210.45 | 54141.65 | 54193.35 | 54027.95 | 54084.95 | 54030.56 | 53987.37 | 53976.33 | 53841 | 53952 |
| 368.0232 | 54010 | 54193.05 | 54353.85 | 54307.79 | 54178.05 | 54098.05 | 54248.6 | 54019.55 | 54052.25 | 54009.44 | 53997.53 | 54029.44 | 53946.5 | 53796 |
| 368.16 | 54046.33 | 54231.5 | 54340.95 | 54367.53 | 54198.9 | 54187.45 | 54256.5 | 54081.5 | 54083.95 | 54046.83 | 54001.26 | 54014.06 | 53942.17 | 53786 |
| 368.2969 | 53982.67 | 54320.75 | 54367.35 | 54386.63 | 54313.75 | 54183.15 | 54250.8 | 54094.25 | 54145 | 54046.61 | 54055 | 54074.89 | 54000.17 | 53952 |
| 368.4337 | 54266.83 | 54186.3 | 54324.85 | 54427.68 | 54236.35 | 54201.5 | 54281.65 | 54052.18 | 54182.75 | 54080.94 | 54008.89 | 53983.89 | 53997.33 | 54109 |
| 368.5705 | 54022.33 | 54350.4 | 54388.55 | 54293 | 54289.3 | 54244.6 | 54387.4 | 54044.4 | 54176.55 | 54016.78 | 54018.95 | 54027.89 | 53970.33 | 53800 |
| 368.7074 | 53972.83 | 54308.8 | 54497.7 | 54425.74 | 54368.4 | 54256.35 | 54354.95 | 54057.35 | 54190 | 54053.61 | 54052.79 | 54048.39 | 53854.83 | 53793 |
| 368.8442 | 54056.33 | 54233.2 | 54348.45 | 54345.63 | 54274.95 | 54247.7 | 54462.4 | 54079.53 | 54264.55 | 54148.39 | 54098.47 | 54024.83 | 53839.83 | 53835 |
| 368.9811 | 54117.67 | 54296.9 | 54284.8 | 54387 | 54362.75 | 54227.9 | 54408.25 | 54114.73 | 54337.1 | 54091.83 | 54125.68 | 54137.17 | 53882.83 | 53978 |
| 369.1179 | 54292.33 | 54378.35 | 54446 | 54372.95 | 54378.25 | 54298.6 | 54370.55 | 54138.2 | 54252.35 | 54162.5 | 54051.53 | 54103.39 | 53905.33 | 54043 |
| 369.2548 | 54064.5 | 54461.2 | 54495.7 | 54351.95 | 54426.2 | 54373.55 | 54520.65 | 54158 | 54349.2 | 54160.61 | 54084.53 | 54067.5 | 53900.83 | 53893 |
| 369.3917 | 54125.67 | 54408.2 | 54369.2 | 54383.32 | 54382.45 | 54451.3 | 54447.15 | 54092.35 | 54430.7 | 54061.78 | 54149.05 | 54056.28 | 54035.5 | 53944 |
| 369.5285 | 54118.5 | 54402.15 | 54534.4 | 54471.95 | 54359.3 | 54416.2 | 54418.55 | 54074.45 | 54318.1 | 54222.94 | 54088.42 | 54084.89 | 53942.33 | 53815 |
| 369.6654 | 54097.33 | 54501.55 | 54678.25 | 54474 | 54429.45 | 54410.55 | 54544.15 | 54140.85 | 54319.7 | 54445.44 | 54115.11 | 54022.83 | 53860.67 | 53826 |
| 369.8023 | 53996.5 | 54572.15 | 54540.6 | 54429.26 | 54487.35 | 54415.3 | 54491.15 | 54051.85 | 54227.4 | 54181.22 | 54055.32 | 54137.17 | 53846.5 | 54081 |
| 369.9392 | 54107.17 | 54449.8 | 54550.15 | 54421.42 | 54493.5 | 54334.4 | 54588.35 | 54115.45 | 54321.35 | 54167.39 | 54116.16 | 54195.44 | 53867.33 | 55302 |
| 370.0761 | 54102.33 | 54505.95 | 54536.8 | 54505.37 | 54481.6 | 54302.05 | 54459.15 | 54176.73 | 54349.45 | 54190.61 | 54144 | 54145.06 | 53854.67 | 54163 |
| 370.213 | 53984.5 | 54437.6 | 54626.95 | 54432.74 | 54517.15 | 54370.8 | 54387.55 | 54189.78 | 54413.35 | 54289.11 | 54134.16 | 54151.94 | 53888.83 | 54389 |
| 370.3499 | 54151.17 | 54522.65 | 54645.65 | 54356.21 | 54561.15 | 54484.25 | 54538.75 | 54101.03 | 54447.95 | 54192.39 | 54172.74 | 54159.06 | 53889.33 | 54036 |
| 370.4868 | 54235.83 | 54479.75 | 54612.4 | 54355.58 | 54450.3 | 54318.3 | 54583.3 | 54082.6 | 54390.05 | 54133.28 | 54050.68 | 54074.83 | 53907 | 53815 |
| 370.6237 | 54088.83 | 54476.7 | 54692.5 | 54368.21 | 54453.35 | 54293.6 | 54590.4 | 54073.8 | 54345.3 | 54192.22 | 54077.53 | 54111 | 53877.67 | 53827 |
| 370.7606 | 54134.17 | 54475.8 | 54490 | 54496.32 | 54403.6 | 54300 | 54566.8 | 54123.05 | 54323.9 | 54241.67 | 54112.11 | 54138.33 | 53985.83 | 53801 |
| 370.8975 | 54100 | 54399.65 | 54437.2 | 54308.79 | 54626.5 | 54343.7 | 54617.25 | 54139.78 | 54295.35 | 54280.61 | 54137.79 | 54091.56 | 53940.67 | 53969 |
| 371.0344 | 54074 | 54394.2 | 54536.6 | 54271.58 | 54518.85 | 54292.95 | 54615.3 | 54133 | 54334.25 | 54199.67 | 54115 | 54082.06 | 54010.33 | 54142 |
| 371.1714 | 54088.33 | 54336.85 | 54610.15 | 54361.16 | 54415.55 | 54341.85 | 54616.15 | 54246.68 | 54314.2 | 54224.83 | 54095.84 | 54114.44 | 54170.67 | 54023 |
| 371.3083 | 54274.83 | 54487.5 | 54586.85 | 54325.32 | 54493.3 | 54316.35 | 54464.15 | 54177.23 | 54342.5 | 54217.11 | 54032.37 | 54069.28 | 53923.67 | 53869 |
| 371.4452 | 54393.5 | 54414.3 | 54742.7 | 54405.11 | 54524.8 | 54310.35 | 54442.15 | 54147.95 | 54373.5 | 54154.11 | 54058.05 | 53974.17 | 53864.5 | 53989 |
| 371.5822 | 54243.17 | 54465.05 | 54611.25 | 54531.11 | 54497.3 | 54268.5 | 54428.7 | 54162.35 | 54485.4 | 54177.39 | 54146.11 | 54099.78 | 53933.17 | 53795 |
| 371.7191 | 54433.33 | 54332.3 | 54732.95 | 54593.21 | 54493.75 | 54323 | 54512.25 | 54241.78 | 54476.15 | 54327.56 | 54209.37 | 54149.28 | 53889.33 | 53787 |
| 371.8561 | 54354.33 | 54357.35 | 54633.3 | 54755.68 | 54409.9 | 54376.05 | 54533.6 | 54225.08 | 54628.05 | 54314.06 | 54235.53 | 54073.94 | 53959.17 | 53955 |
| 371.993 | 54247 | 54500.7 | 54541.85 | 54795.42 | 54609.85 | 54544.95 | 54707.4 | 54358.13 | 54654.85 | 54224.06 | 54222.58 | 54080.06 | 53984.67 | 53981 |
| 372.1299 | 54282.83 | 54433.5 | 54803.6 | 54975.53 | 54632.55 | 54579.15 | 54871.85 | 54461.23 | 54714.95 | 54295.94 | 54143.37 | 54070.89 | 54038.5 | 54393 |
| 372.2669 | 54259.33 | 54623.55 | 54870.3 | 54772.53 | 54659.3 | 54526.95 | 54873.5 | 54400.53 | 54651 | 54413.06 | 54107.11 | 54040.33 | 54051.5 | 54379 |
| 372.4039 | 54216.83 | 54481.15 | 54770.8 | 54649.11 | 54552.9 | 54582.3 | 54935.6 | 54245.78 | 54531.65 | 54369.22 | 54118.58 | 54148.22 | 53923.33 | 54403 |
| 372.5408 | 54180.5 | 54495.85 | 54781.95 | 54503.05 | 54688.2 | 54572.65 | 54837.55 | 54252.73 | 54553.3 | 54293.17 | 54157.74 | 54194.89 | 53978.83 | 53925 |
| 372.6778 | 54246.33 | 54513.9 | 54775.1 | 54508.63 | 54588.75 | 54553.05 | 54717.15 | 54188.3 | 54441.9 | 54278.83 | 54137.21 | 54138.83 | 54056.17 | 53815 |
| 372.8148 | 54337.83 | 54418.5 | 54615.85 | 54475.79 | 54449.65 | 54524.3 | 54706.75 | 54191.85 | 54342.35 | 54322.67 | 54117.32 | 54124.44 | 54048.17 | 53833 |
| 372.9518 | 54262 | 54449.3 | 54604.65 | 54620.68 | 54558.25 | 54325.4 | 54589.65 | 54211.9 | 54451 | 54342.44 | 54192.37 | 54132 | 53863.17 | 54289 |
| 373.0888 | 54291.83 | 54494.5 | 54619.35 | 54630 | 54708.4 | 54361.4 | 54578.3 | 54273.1 | 54607.45 | 54299.56 | 54345.53 | 54132.72 | 53946.17 | 54187 |
| 373.2257 | 54511.17 | 54469.4 | 54776.7 | 54724.32 | 54686.35 | 54460.25 | 54524.9 | 54193.15 | 54545.5 | 54202.83 | 54285.11 | 54063.83 | 53929.33 | 54035 |
| 373.3627 | 54287.83 | 54436.55 | 54746.55 | 55024.89 | 54668.6 | 54416.45 | 54700.9 | 54185.43 | 54529.7 | 54318.89 | 54205.05 | 54119.33 | 53972.67 | 54813 |
| 373.4997 | 54193.5 | 54542.9 | 54768.25 | 54795.42 | 54501.2 | 54500.25 | 54826.1 | 54299.35 | 54569 | 54295.89 | 54298.95 | 54205.56 | 54025 | 54226 |
| 373.6367 | 54311.67 | 54569.1 | 54806.35 | 54727 | 54585.9 | 54640.2 | 54833.4 | 54349.63 | 54540.7 | 54186.17 | 54386.47 | 54149.89 | 53918.33 | 53889 |
| 373.7737 | 54695.5 | 54841.45 | 54712.85 | 54724.74 | 54809.3 | 54748.25 | 54805.4 | 54377.88 | 54652.5 | 54185.28 | 54269.84 | 54060.78 | 53915.17 | 54073 |
| 373.9107 | 54444.83 | 54823 | 54678.65 | 54718.47 | 54716.65 | 54634.5 | 54949.5 | 54392.55 | 54545.6 | 54282.44 | 54273.95 | 54119.72 | 53872.83 | 54023 |
| 374.0477 | 54408.33 | 54558.4 | 54802.4 | 54643.74 | 54568.4 | 54647.55 | 55024 | 54256.75 | 54588.3 | 54265.39 | 54159 | 54024.72 | 53957.17 | 53999 |
| 374.1848 | 54407 | 54641.15 | 54974.4 | 54690.74 | 54577.8 | 54709.85 | 54864.7 | 54272.98 | 54518.95 | 54268.44 | 54235.95 | 54114.11 | 53890.5 | 54353 |
| 374.3218 | 54449.17 | 54555.15 | 54933.25 | 54827.84 | 54668.45 | 54555.15 | 54781.8 | 54268.03 | 54727.15 | 54256.67 | 54345.63 | 54147.33 | 53837.83 | 54077 |
| 374.4588 | 54332 | 54603.8 | 54825.7 | 55013.11 | 54664.1 | 54530.95 | 54891 | 54305.63 | 54737.15 | 54199.44 | 54304 | 54097.06 | 53844.17 | 53823 |
| 374.5958 | 54709.33 | 54741.3 | 54850.25 | 55034.16 | 54768.65 | 54702.9 | 54997.95 | 54343.03 | 54674.45 | 54199.56 | 54331.21 | 54075.11 | 53979.67 | 53845 |
| 374.7329 | 54698.5 | 54660.75 | 54781.2 | 55192.79 | 54782.25 | 54748.45 | 55057.7 | 54405.65 | 54681.7 | 54297.67 | 54261.84 | 54241.72 | 54019.17 | 53806 |
| 374.8699 | 54448.83 | 54893.4 | 54875.25 | 55219.42 | 54925.55 | 54783.7 | 54920.35 | 54490.65 | 54735.75 | 54296.78 | 54377.16 | 54152.28 | 53908.33 | 53968 |
| 375.0069 | 54469.33 | 54875.1 | 54825.95 | 54930.42 | 54846.85 | 54836.65 | 55096.2 | 54615.48 | 54697.75 | 54341.61 | 54226.11 | 54167.17 | 54048.17 | 54194 |
| 375.144 | 54365.67 | 54803.7 | 54945.5 | 54779.16 | 54988.25 | 54852.85 | 55210 | 54511.83 | 54617.25 | 54404.39 | 54229.58 | 54154.17 | 54134 | 54155 |
| 375.281 | 54298.33 | 54758 | 54978.55 | 54892.16 | 55000.95 | 54810.9 | 55033.45 | 54445.4 | 54470.4 | 54324.5 | 54263.47 | 54094.83 | 54003 | 53870 |
| 375.4181 | 54282.67 | 54753 | 55097.8 | 55116.58 | 54754.05 | 54830.55 | 55024.6 | 54413.35 | 54442.5 | 54262.5 | 54242.63 | 54243.33 | 53951.17 | 53952 |
| 375.5551 | 54311.67 | 54757.1 | 54854.05 | 54991.05 | 54794.75 | 54746.6 | 54989.15 | 54326.48 | 54563.5 | 54375.39 | 54265.42 | 54267.44 | 54070.67 | 54736 |
| 375.6922 | 54344.5 | 54706.85 | 55012.35 | 54832.89 | 54786.9 | 54633.5 | 54976.4 | 54295.38 | 54636.9 | 54321 | 54267.21 | 54361.39 | 53986.67 | 54019 |
| 375.8292 | 54443.83 | 54615.3 | 55038.45 | 54837.84 | 54852.4 | 54597.9 | 54990.1 | 54333.75 | 54666.2 | 54346.61 | 54187.21 | 54382.22 | 53929.83 | 54014 |
| 375.9663 | 54559.33 | 54800.75 | 55084.3 | 54813.32 | 54688.7 | 54700.25 | 54919.7 | 54288.6 | 54691 | 54304.39 | 54138.05 | 54388.67 | 54011.5 | 54022 |
| 376.1033 | 54170.5 | 54834.75 | 54940.7 | 54825.58 | 54877.1 | 54602.4 | 54854.15 | 54299.48 | 54566.75 | 54330 | 54190.58 | 54280.06 | 53980.67 | 53856 |
| 376.2404 | 54378.67 | 54818.5 | 54871.4 | 54855 | 54872.65 | 54631.05 | 54836.15 | 54263.08 | 54809.35 | 54433.78 | 54195.53 | 54196.06 | 53930.67 | 53805 |
| 376.3775 | 54416.33 | 54715.65 | 55075.55 | 54875.37 | 54882.1 | 54714.7 | 54915 | 54289.45 | 54638.15 | 54398.11 | 54229.68 | 54160.33 | 53944.33 | 53800 |
| 376.5146 | 54412.33 | 54671.65 | 54932.05 | 54754.68 | 54814.65 | 54684.6 | 54852.8 | 54334.9 | 54564.5 | 54392.78 | 54223.42 | 54182.78 | 53963.33 | 53845 |
| 376.6516 | 54416.33 | 54664.4 | 54889.35 | 54623.68 | 54791.1 | 54639.1 | 54793.8 | 54252.38 | 54580.3 | 54282.17 | 54248.37 | 54080.44 | 53961 | 53808 |
| 376.7887 | 54598.5 | 54614.35 | 54791.3 | 54622.68 | 54692.3 | 54629.5 | 54856.45 | 54312.18 | 54696.3 | 54250.39 | 54175.37 | 54178.44 | 54041.17 | 53742 |
| 376.9258 | 54713.5 | 54663.65 | 54780.65 | 54728.89 | 54855.95 | 54731.05 | 54758.4 | 54304.45 | 54754.1 | 54411.83 | 54211.63 | 54091.39 | 53961.5 | 53756 |
| 377.0629 | 54335.33 | 54740.35 | 54814.35 | 54498.74 | 54922.65 | 54644.5 | 54777.9 | 54248.48 | 54621.9 | 54438.72 | 54230.11 | 54118.06 | 53988.67 | 53795 |
| 377.2 | 54277 | 54728.75 | 54867.3 | 54557.68 | 54765.35 | 54736.3 | 54691.15 | 54194.6 | 54723.95 | 54332.11 | 54155.37 | 54154.5 | 53990 | 53924 |
| 377.3371 | 54449.5 | 54769.45 | 54845.75 | 54616.79 | 54711.8 | 54659.55 | 54661.45 | 54273.03 | 54700.8 | 54216.28 | 54148.26 | 54182.44 | 53970 | 53959 |
| 377.4742 | 54527 | 54770.35 | 54698.85 | 54670.26 | 54687.7 | 54563.9 | 54744.1 | 54233.93 | 54676.25 | 54180.89 | 54176.47 | 54280.78 | 53901.67 | 54258 |
| 377.6113 | 54288.33 | 54829.3 | 54655 | 54775.21 | 54714.75 | 54535.35 | 54586.9 | 54221.2 | 54784.1 | 54259.11 | 54216.26 | 54225.61 | 53975.17 | 53856 |
| 377.7484 | 54255 | 54843.9 | 54764.25 | 54681.26 | 54658.7 | 54585.1 | 54609.85 | 54281.35 | 54660.15 | 54300.33 | 54248.37 | 54244.89 | 53917.67 | 53957 |
| 377.8855 | 54276.17 | 54688.4 | 54808.7 | 54627.89 | 54516.7 | 54551.75 | 54819.3 | 54327.4 | 54625.95 | 54225.56 | 54144.05 | 54144.11 | 53862.67 | 54416 |
| 378.0226 | 54115.83 | 54686.2 | 54936.35 | 54644.26 | 54631.35 | 54581.85 | 54801.6 | 54282 | 54605.45 | 54243.83 | 54106.37 | 54155.56 | 53964 | 54142 |
| 378.1597 | 54184.5 | 54756.8 | 54957.7 | 54601.26 | 54747.3 | 54598.8 | 54790.2 | 54269.08 | 54554.05 | 54247.56 | 54170.84 | 54207.89 | 53934 | 54276 |
| 378.2968 | 54227.5 | 54936.95 | 54977.85 | 54714.58 | 54709.3 | 54571.6 | 54741.1 | 54272.93 | 54632.65 | 54240.72 | 54227.26 | 54257.11 | 54062.67 | 54308 |
| 378.4339 | 54203 | 54910.6 | 54766.9 | 54715.53 | 54704.15 | 54551.65 | 54665 | 54221.88 | 54761.45 | 54202 | 54242.63 | 54207.67 | 54086 | 54339 |
| 378.571 | 54203.33 | 54853.3 | 54743.8 | 54774.84 | 54697.75 | 54493.55 | 54757.75 | 54226.63 | 54596.55 | 54260.72 | 54286.26 | 54277.22 | 53918 | 54374 |
| 378.7082 | 54332.83 | 54881.45 | 54793.9 | 54732.84 | 54607.45 | 54579.55 | 54674.45 | 54232.83 | 54604.6 | 54327.83 | 54178.79 | 54144.72 | 53976.67 | 53841 |
| 378.8453 | 54591.5 | 54929.35 | 54742.7 | 54922.26 | 54819 | 54535.45 | 54847 | 54217.08 | 54525.4 | 54204.39 | 54156.42 | 54177.11 | 54072.5 | 53952 |
| 378.9825 | 54437.17 | 54872.55 | 54908.4 | 54792.53 | 54813.15 | 54627.8 | 54849.65 | 54180.3 | 54514.15 | 54304.39 | 54084 | 54158.5 | 53974.5 | 54601 |
| 379.1196 | 54452.83 | 54842.1 | 55041.85 | 54717.26 | 54683.55 | 54541.3 | 54839.65 | 54180.55 | 54748.7 | 54413.78 | 54124.63 | 54276 | 53924 | 54213 |
| 379.2567 | 54247.5 | 54861.4 | 54823.5 | 54774.84 | 54900.1 | 54451.25 | 54857.8 | 54244.33 | 54886.3 | 54437.22 | 54266.32 | 54203.28 | 53905.67 | 54023 |
| 379.3939 | 54399.17 | 54866.3 | 54755.1 | 54782.42 | 54896.15 | 54552.15 | 54705.85 | 54264.48 | 54587.2 | 54330.78 | 54205.05 | 54141 | 53918.5 | 53802 |
| 379.531 | 54304.33 | 54729.7 | 54893.75 | 54688.42 | 54781 | 54519.4 | 54642.85 | 54320.25 | 54633.35 | 54257.78 | 54192 | 54204.11 | 53970.17 | 53817 |
| 379.6681 | 54278.5 | 54815.2 | 54854.65 | 54590.21 | 54834.75 | 54584.65 | 54629.4 | 54229.75 | 54566.65 | 54284.83 | 54222.32 | 54139.28 | 53977 | 53826 |
| 379.8053 | 54487 | 54739.35 | 54912.9 | 54620.84 | 54761 | 54694.5 | 54699.15 | 54169.35 | 54692.1 | 54294 | 54197.11 | 54173.72 | 54087.83 | 54067 |
| 379.9424 | 54765.33 | 54697 | 54799.15 | 54673.68 | 54721.6 | 54587.1 | 54853.1 | 54226.38 | 54568.15 | 54354.94 | 54158.05 | 54169.11 | 54120 | 54011 |
| 380.0796 | 54720.5 | 54616.6 | 54886.95 | 54687.68 | 54719 | 54610.55 | 54853.25 | 54254.45 | 54728.6 | 54369.89 | 54108.74 | 54178.56 | 54121 | 54047 |
| 380.2167 | 54468.83 | 54718.75 | 54803.05 | 54808 | 54722.4 | 54693.05 | 54915.35 | 54321.68 | 54855.6 | 54249.11 | 54093.53 | 54251.33 | 54028.33 | 53856 |
| 380.3539 | 54272.5 | 54563.35 | 54779.85 | 54833.89 | 54638 | 54642.1 | 54871.2 | 54311.28 | 54811.45 | 54213.67 | 54123.42 | 54277.11 | 54272.33 | 54031 |
| 380.4911 | 54456.5 | 54557.5 | 54824.65 | 54767.16 | 54643.45 | 54598.9 | 54786.5 | 54215.9 | 54702.7 | 54260.5 | 54192.63 | 54210.78 | 54210.67 | 54134 |
| 380.6282 | 54434.83 | 54710.1 | 54908.6 | 54825.21 | 54731.05 | 54517.6 | 54791.7 | 54218.65 | 55132.9 | 54195.5 | 54232.42 | 54380.44 | 54094.5 | 53960 |
| 380.7654 | 54210 | 54707.55 | 54698.75 | 54629.05 | 54779.6 | 54511.1 | 54830.15 | 54199.33 | 55385.15 | 54310.78 | 54212.16 | 54385.06 | 54064.17 | 53838 |
| 380.9025 | 54262.83 | 54746.9 | 54709.35 | 54566.74 | 54670.75 | 54502.4 | 54759.3 | 54330.38 | 54862.1 | 54364.61 | 54140.21 | 54305.17 | 54060.5 | 53878 |
| 381.0397 | 54285.67 | 54652.45 | 54814.2 | 54587.37 | 54793.5 | 54604 | 54790.45 | 54236.95 | 54593.55 | 54280.78 | 54074 | 54221.11 | 54036.17 | 54244 |
| 381.1769 | 54256.83 | 54664.6 | 55016.75 | 54666.58 | 54620 | 54641.45 | 54785.3 | 54170.13 | 54625.05 | 54299.78 | 54193.42 | 54192.39 | 54133 | 54452 |
| 381.3141 | 54382 | 54602.85 | 54956.65 | 54718.74 | 54720.85 | 54592.45 | 54932.05 | 54168.83 | 54684.4 | 54284.11 | 54218 | 54123.11 | 54208.67 | 53983 |
| 381.4512 | 54363 | 54629.25 | 55068.75 | 54830.68 | 54674.25 | 54543.4 | 54777.15 | 54220.55 | 54680.6 | 54296.67 | 54279.42 | 54275.78 | 54170 | 53846 |
| 381.5884 | 54336.83 | 54702.95 | 55062.2 | 54817.42 | 54646.2 | 54560.65 | 54794.9 | 54231.63 | 54578.2 | 54303.11 | 54180 | 54220.78 | 54056 | 53868 |
| 381.7256 | 54110.33 | 54711.95 | 54958.1 | 54658.11 | 54758.25 | 54642.65 | 54741.5 | 54204.65 | 54598.9 | 54323.61 | 54209.89 | 54368.33 | 54082.67 | 53862 |
| 381.8628 | 54330.83 | 54715.15 | 55018.5 | 54652.68 | 54786.55 | 54663.85 | 54853.05 | 54204.18 | 54618.9 | 54272 | 54216.37 | 54483.67 | 53944.67 | 54215 |
| 382 | 54330.17 | 54825.45 | 54828.3 | 54815.05 | 54881.75 | 54647.4 | 55070.55 | 54267.33 | 54604.35 | 54251.17 | 54278.11 | 54365 | 53999.17 | 54121 |
| 382.1372 | 54216.33 | 54820.5 | 54974.4 | 54772.63 | 54713.85 | 54589.95 | 54985.75 | 54371.7 | 54592.95 | 54383.17 | 54113.84 | 54367.61 | 53968.17 | 54062 |
| 382.2743 | 54535.5 | 54773.35 | 54902.1 | 54947.84 | 54760.5 | 54783.9 | 55029.7 | 54437.13 | 54736.7 | 54302.11 | 54130.53 | 54254.28 | 54109.5 | 54916 |
| 382.4115 | 54653.83 | 54844.95 | 55030.15 | 55017.21 | 54832.1 | 54588 | 54928.25 | 54396.53 | 54582.5 | 54381 | 54116.95 | 54234.61 | 53944.33 | 54069 |
| 382.5487 | 54398.17 | 54885.65 | 54919 | 54951.37 | 54833.3 | 54728.4 | 55004.95 | 54444.6 | 54665.35 | 54338.94 | 54157.47 | 54305.56 | 54027.67 | 54351 |
| 382.6859 | 54436 | 54941.05 | 54843.8 | 55074.63 | 54712.75 | 54661.85 | 55278.45 | 54454 | 54951.1 | 54440.39 | 54263.42 | 54317.89 | 53978.83 | 54483 |
| 382.8231 | 54270.17 | 54914.45 | 54927.9 | 55062.42 | 54748.5 | 54626.85 | 55057.25 | 54445.65 | 54870.8 | 54461.89 | 54272.16 | 54349.89 | 53936.17 | 53962 |
| 382.9603 | 54617.5 | 54699.7 | 54855.75 | 54999.95 | 54900.35 | 54643.4 | 55047.6 | 54368.1 | 54746 | 54356.44 | 54317.26 | 54393.83 | 54011.17 | 53989 |
| 383.0975 | 54701.17 | 54673.8 | 54911.45 | 54986.79 | 54843.1 | 54594.85 | 54967.6 | 54342.55 | 54670.6 | 54307.78 | 54237.95 | 54277.17 | 53973 | 54181 |
| 383.2347 | 54487.5 | 54805.35 | 54965.45 | 55006.68 | 54901.1 | 54660.6 | 55057.05 | 54380.9 | 54496.1 | 54364.56 | 54281.53 | 54358 | 53983.83 | 53995 |
| 383.3719 | 54407.67 | 54893.55 | 55120 | 55005.16 | 54905.45 | 54674.6 | 55079.85 | 54415.75 | 54675.25 | 54555.67 | 54214.26 | 54313 | 54108.83 | 53932 |
| 383.5091 | 54393.83 | 54914.45 | 55003.45 | 54803.84 | 54903.9 | 54669.6 | 55018.95 | 54408.98 | 54819.9 | 54492.17 | 54144.58 | 54246.83 | 54030.33 | 54218 |
| 383.6464 | 54420 | 55020.25 | 55116.5 | 54776.74 | 54921.6 | 54804.2 | 55096.6 | 54424.48 | 54896.75 | 54526.5 | 54181.53 | 54272.72 | 54112.17 | 54229 |
| 383.7836 | 54639.83 | 54982.4 | 55093.7 | 54832.95 | 54973 | 54843.15 | 55189.8 | 54394.85 | 54946.1 | 54446.89 | 54155.16 | 54396.28 | 54011.5 | 54021 |
| 383.9208 | 54281 | 55030.4 | 55107 | 54872.79 | 54821.5 | 54756 | 55269.2 | 54333.08 | 54755.9 | 54425.06 | 54225.21 | 54298.11 | 53999.67 | 54398 |
| 384.058 | 54422.5 | 54930.1 | 55034.05 | 54997.58 | 54937.4 | 54779.4 | 55318 | 54362.63 | 54765.35 | 54542.11 | 54235.16 | 54286.72 | 53985.83 | 54508 |
| 384.1952 | 54594.5 | 54848.45 | 55093.9 | 54993.84 | 55032.6 | 54713.95 | 55141.05 | 54424.35 | 54849.5 | 54471.56 | 54322.26 | 54320.72 | 53994.5 | 53913 |
| 384.3324 | 54371.17 | 54835.2 | 54996.35 | 55041.21 | 55040.35 | 54741.95 | 55079.8 | 54421.8 | 54768.9 | 54321.67 | 54237.11 | 54435.44 | 53960.83 | 53979 |
| 384.4696 | 54402.67 | 54906.5 | 55144.6 | 55109.11 | 55162.75 | 54641.1 | 55064.65 | 54466.4 | 54817.05 | 54307.61 | 54209 | 54482.5 | 54109 | 53979 |
| 384.6069 | 54833 | 55020.35 | 55284.75 | 54876.47 | 55119.55 | 54777 | 55121.2 | 54341.4 | 54797.65 | 54312.5 | 54223 | 54322.39 | 53983.17 | 53883 |
| 384.7441 | 54464.17 | 55042.4 | 55085.95 | 54860.84 | 55079.65 | 54836.3 | 55056.65 | 54393.88 | 54886.4 | 54292.89 | 54276 | 54373.17 | 54108.5 | 54036 |
| 384.8813 | 54348.5 | 55006.85 | 55110.7 | 54961.63 | 54906.75 | 54926.2 | 55113 | 54328.6 | 54847.85 | 54293.22 | 54443.58 | 54488.89 | 54218.17 | 54324 |
| 385.0186 | 54342.83 | 55091.85 | 54974.5 | 54858.58 | 54937.1 | 54747.3 | 55001.4 | 54291.4 | 54934.25 | 54307.33 | 54284.79 | 54371.06 | 54162.17 | 53913 |
| 385.1558 | 54518.67 | 55088.95 | 54995.9 | 54877.84 | 54896.3 | 54821.3 | 54901.3 | 54384.35 | 54913.1 | 54474.67 | 54316.47 | 54428.56 | 54176.5 | 54173 |
| 385.293 | 54626.83 | 54884.15 | 54977.1 | 55077.05 | 54731.75 | 54697.05 | 55004.85 | 54284.15 | 54854.25 | 54391.94 | 54275.26 | 54325.72 | 54101.33 | 54373 |
| 385.4302 | 54372.33 | 54837.4 | 55073.55 | 54973.68 | 54760.2 | 54639.7 | 55031.85 | 54392.33 | 54733.2 | 54329.61 | 54290.53 | 54218.56 | 54277.83 | 54181 |
| 385.5675 | 54581.83 | 54875.95 | 55160.65 | 54740.42 | 54737.2 | 54743.15 | 54978.4 | 54386.98 | 54798.95 | 54370.78 | 54282.32 | 54249.06 | 54114 | 54453 |
| 385.7047 | 54540.67 | 54970.65 | 55122.2 | 54795.32 | 54943.2 | 54691.5 | 55014.9 | 54396.2 | 54705.35 | 54425.17 | 54502.58 | 54205.28 | 54175.67 | 54155 |
| 385.842 | 54668.67 | 54862.65 | 55206.5 | 54932.95 | 55152.15 | 54821.35 | 55128.05 | 54451.38 | 54778.45 | 54380.22 | 54360.26 | 54176.89 | 54072 | 54049 |
| 385.9792 | 54589.83 | 54865.4 | 55183.5 | 55091.84 | 54995.85 | 54725.45 | 55310.8 | 54518.55 | 54801.05 | 54436 | 54280.74 | 54222.94 | 54024.17 | 54081 |
| 386.1164 | 54562.67 | 55073.9 | 55137 | 55105.63 | 55104.55 | 54745.7 | 55627.2 | 54537.88 | 54830.35 | 54446.67 | 54306.47 | 54334.67 | 54061.5 | 54197 |
| 386.2537 | 54401.17 | 55009.3 | 54980.45 | 55074.58 | 55110.3 | 54800.95 | 55438.9 | 54502.85 | 54739.9 | 54420.22 | 54257.16 | 54326.61 | 53960.83 | 54305 |
| 386.3909 | 54518.83 | 55020.6 | 55006.9 | 55018.32 | 54874.2 | 54684.7 | 55092.1 | 54431.63 | 54886.85 | 54551.61 | 54243.05 | 54245.06 | 54004.17 | 54545 |
| 386.5282 | 54444 | 54959.6 | 55095 | 54983.16 | 54966.15 | 54749.65 | 55117.65 | 54385.13 | 54682.95 | 54514.78 | 54200.74 | 54289.72 | 53949.83 | 54071 |
| 386.6654 | 54198.17 | 54835.5 | 55200.6 | 54924.68 | 54928.75 | 54635.8 | 55078.4 | 54387 | 54688.65 | 54435.78 | 54181.16 | 54266.06 | 53983 | 53835 |
| 386.8026 | 54426.83 | 54924.3 | 55132.45 | 54891.11 | 54938.55 | 54682.8 | 54893.75 | 54378.5 | 54705.3 | 54485.67 | 54222.58 | 54203.44 | 54179.17 | 53975 |
| 386.9399 | 54420.67 | 54969.6 | 55024.5 | 54949.37 | 54965.05 | 54614.25 | 55055.7 | 54364.35 | 54728.6 | 54576.33 | 54168.68 | 54334.61 | 54093.17 | 54514 |
| 387.0772 | 54477.83 | 55017.55 | 55102.3 | 55063.05 | 55029.15 | 54688.2 | 54865.05 | 54366.13 | 54846.2 | 54467.22 | 54190.47 | 54181.28 | 53971.5 | 54085 |
| 387.2144 | 54463.83 | 54953.3 | 55092 | 55024.47 | 55073.7 | 54919.3 | 54766.45 | 54332.08 | 54777.9 | 54350.33 | 54191.05 | 54317.67 | 54064.83 | 53891 |
| 387.3517 | 54493.83 | 55030.45 | 54946.65 | 54896.37 | 54986.45 | 54889.25 | 54952.3 | 54378.05 | 54828.7 | 54487.11 | 54175.58 | 54263.56 | 54088.67 | 54059 |
| 387.4889 | 54475.67 | 55322.85 | 55109.2 | 54747.32 | 54885.65 | 54871.2 | 55137.6 | 54457.43 | 54925.1 | 54492.61 | 54267.11 | 54171.89 | 54100.5 | 54196 |
| 387.6262 | 54745 | 55442.5 | 55166.9 | 54889.32 | 54992.4 | 55069.9 | 55338.75 | 54455.55 | 55001.15 | 54484.11 | 54461.21 | 54261.94 | 54047.67 | 54246 |
| 387.7634 | 54369.5 | 55215.6 | 55221.55 | 54948.84 | 55145.05 | 54891.95 | 55094.95 | 54488.3 | 54799.1 | 54455.56 | 54429.95 | 54264.67 | 54111.5 | 54047 |
| 387.9007 | 54310.83 | 55168.35 | 55519.4 | 55106.05 | 54986.2 | 54913.55 | 55032.45 | 54513.03 | 54839.1 | 54499.44 | 54368.16 | 54293.33 | 54180.5 | 54115 |
| 388.0379 | 54609.17 | 54910.65 | 55355.2 | 55242.21 | 55080.2 | 54841.85 | 55056.95 | 54478.48 | 54941.15 | 54650.72 | 54219.32 | 54348.33 | 54122.17 | 54286 |
| 388.1752 | 54503.83 | 54877.85 | 55459.05 | 55251.11 | 54973.85 | 54967.4 | 55053 | 54432.95 | 54798.55 | 54664.33 | 54237.16 | 54336.39 | 54062.33 | 54252 |
| 388.3124 | 54601 | 55202.6 | 55478.6 | 55256 | 54916.55 | 54915.55 | 55064.85 | 54393.28 | 54930.4 | 54487.44 | 54225 | 54335 | 54044.83 | 54578 |
| 388.4497 | 54483 | 55147.2 | 55452.6 | 55212.68 | 55145.8 | 54956.05 | 55144.7 | 54413.48 | 55038.4 | 54404.72 | 54363 | 54499.06 | 54167.83 | 54132 |
| 388.587 | 54380.5 | 55154.75 | 55365.15 | 55229.68 | 55102.4 | 54951.15 | 55192.35 | 54571.13 | 54931.15 | 54574.33 | 54332.63 | 54403.89 | 53993.33 | 54135 |
| 388.7242 | 54632.17 | 55247.45 | 55342.55 | 55258.53 | 55195.4 | 54945.7 | 55186.6 | 54728.25 | 55004.8 | 54526.78 | 54482.89 | 54392.5 | 53940.83 | 54121 |
| 388.8615 | 54741 | 55238.15 | 55437.7 | 54992.21 | 55253.7 | 55000.45 | 55290.2 | 54831.48 | 55096.3 | 54537.22 | 54492.32 | 54394.78 | 54029.17 | 53968 |
| 388.9988 | 55041.33 | 55087.4 | 55512.5 | 54942.16 | 55200.3 | 54946.85 | 55160.35 | 54749.58 | 55035.55 | 54553.22 | 54257.79 | 54323.56 | 53945 | 53890 |
| 389.136 | 54988.17 | 55150 | 55380.25 | 55136.95 | 55199.2 | 54864.8 | 55258.2 | 54603.4 | 55160.5 | 54529.44 | 54445.32 | 54334.78 | 54059 | 54355 |
| 389.2733 | 54877.67 | 55264.75 | 55466.15 | 55077.05 | 55300.4 | 54928.6 | 55194.2 | 54525.83 | 54987.85 | 54430.83 | 54323.89 | 54316.17 | 54135.5 | 54653 |
| 389.4106 | 54788.83 | 55211.9 | 55552.4 | 55134.37 | 55168.45 | 54948.7 | 55054.45 | 54485.4 | 55001 | 54516.17 | 54279.32 | 54339.22 | 54033.17 | 54118 |
| 389.5478 | 54892.17 | 55178 | 55342.2 | 55226.74 | 54946.1 | 54811.5 | 55051.35 | 54449.38 | 55018.8 | 54468 | 54307.95 | 54346 | 53975.67 | 54404 |
| 389.6851 | 54400.33 | 55228.3 | 55287.3 | 55114.26 | 55034.45 | 54869.15 | 55145.75 | 54418.95 | 55026.5 | 54519.67 | 54356.68 | 54387.61 | 53894.5 | 54641 |
| 389.8224 | 54591.17 | 55068.35 | 55540.65 | 55154.37 | 55134 | 55172.65 | 55252.9 | 54482.93 | 54947.7 | 54423.61 | 54276.89 | 54310.56 | 53863.33 | 54387 |
| 389.9596 | 54426.67 | 55119.4 | 55289.1 | 55293.42 | 55042.2 | 54969.45 | 55087.6 | 54460.73 | 54948.35 | 54424.61 | 54323.47 | 54321.78 | 54057 | 54599 |
| 390.0969 | 54913 | 55068.85 | 55282.25 | 55378.68 | 54795.15 | 54951.4 | 55122.2 | 54471.05 | 54893.6 | 54437.83 | 54405.74 | 54276.17 | 53941.33 | 54356 |
| 390.2342 | 54525 | 55114.25 | 55279.2 | 54805.37 | 54962.1 | 54802.6 | 55263.35 | 54459.15 | 54818.3 | 54396.67 | 54207.11 | 54150.39 | 54016.5 | 54809 |
| 390.3715 | 54499.17 | 55149.45 | 55368.45 | 55088.53 | 55189.2 | 54759.7 | 55331.4 | 54382.65 | 54823.05 | 54414 | 54121.32 | 54159.89 | 54096.67 | 54735 |
| 390.5087 | 54585.5 | 55087.65 | 55208.6 | 54926.42 | 54949.7 | 54879.05 | 55186.6 | 54469.18 | 54796.75 | 54489.39 | 54239.42 | 54227.33 | 53985.5 | 54639 |
| 390.646 | 54595.67 | 54960.1 | 55186 | 54934.42 | 54872.15 | 54823.4 | 55013.35 | 54476.25 | 54798.45 | 54542.5 | 54248.16 | 54344.72 | 53960.67 | 53887 |
| 390.7833 | 54550.5 | 55118.25 | 55224.9 | 55184.89 | 54960.6 | 54865.8 | 55044.6 | 54448.45 | 54693.6 | 54473.83 | 54262.84 | 54278.56 | 53961 | 53912 |
| 390.9205 | 54459.33 | 54919.15 | 55415.85 | 55105.05 | 54968.45 | 54878.25 | 55162.5 | 54377.88 | 54743.2 | 54395.67 | 54234.42 | 54280.39 | 53937.83 | 54062 |
| 391.0578 | 54728.5 | 54980.7 | 55381.8 | 55080.74 | 55027.65 | 54896.5 | 55342.8 | 54474.53 | 54851.25 | 54541.28 | 54220.74 | 54398.17 | 54014.17 | 53870 |
| 391.1951 | 55049.83 | 55078.95 | 55331.8 | 55031.79 | 55239.7 | 54906.75 | 55001.2 | 54527.43 | 54937.8 | 54635.5 | 54468.26 | 54397.28 | 54093.33 | 53820 |
| 391.3324 | 54768.5 | 55334.2 | 55270.75 | 55333.32 | 55268.7 | 54958.9 | 55128.7 | 54462.5 | 54874.1 | 54632.33 | 54521.53 | 54292.61 | 54004.17 | 53890 |
| 391.4696 | 54690 | 55251.8 | 55351.9 | 55485.89 | 55181.9 | 55005.7 | 55240.7 | 54489.48 | 55099.75 | 54753.78 | 54413.21 | 54226.28 | 54026.5 | 53892 |
| 391.6069 | 54763.33 | 55146.3 | 55389.05 | 55327.95 | 55171.15 | 54931.65 | 55274.7 | 54518.6 | 55080.5 | 54762.89 | 54429.53 | 54338.22 | 54018 | 53865 |
| 391.7442 | 54963.5 | 55254.75 | 55530.6 | 55413.32 | 55151.95 | 54952.15 | 55379.3 | 54523.83 | 55094.4 | 54587.78 | 54363.58 | 54355.61 | 53943.5 | 53934 |
| 391.8815 | 54876.83 | 55447 | 55422.9 | 55507.74 | 55321.45 | 55038.25 | 55398.95 | 54462.08 | 55187.55 | 54539 | 54348.16 | 54370.56 | 54025.67 | 54942 |
| 392.0187 | 54713.67 | 55474.55 | 55575.15 | 55514.37 | 55247.3 | 55189.95 | 55373.75 | 54641.7 | 55106.1 | 54622.67 | 54504.95 | 54428.44 | 54039.67 | 54575 |
| 392.156 | 54793.33 | 55307.3 | 55842.7 | 55356.74 | 55390.45 | 55202.7 | 55613.05 | 54702.65 | 55264.05 | 54608.44 | 54582 | 54431 | 54034.33 | 54448 |
| 392.2933 | 55052.17 | 55431.05 | 55800.5 | 55402.68 | 55624.85 | 55291.05 | 55577.65 | 54698.4 | 55133.5 | 54758.44 | 54551.21 | 54490.33 | 54152 | 54371 |
| 392.4306 | 55090.33 | 55564.45 | 55941.85 | 55653.47 | 55572.95 | 55412.2 | 55763.85 | 54712.3 | 55210.1 | 54648.22 | 54693.95 | 54540.61 | 54080 | 54188 |
| 392.5678 | 55125.67 | 55563.7 | 56091.65 | 55576 | 55598.75 | 55294.75 | 55805.05 | 54809.73 | 55474.6 | 54762.44 | 54603.37 | 54544.94 | 54108.5 | 54710 |
| 392.7051 | 55261.5 | 55601.25 | 56161.1 | 56073.47 | 55789.85 | 55289.45 | 55734.55 | 54906.88 | 55546.3 | 54880.78 | 54611.26 | 54525.11 | 54084 | 54356 |
| 392.8424 | 55130.17 | 55844.95 | 55946.4 | 56286.68 | 55888.5 | 55487.15 | 55991.75 | 55023.3 | 55661.5 | 54880.5 | 54820.58 | 54508.11 | 54021.5 | 54185 |
| 392.9797 | 55958 | 56000.3 | 56434.75 | 56987.47 | 56015 | 55655.7 | 56168 | 55136.03 | 56379.85 | 54878.39 | 54744.37 | 54669.17 | 54093.17 | 54101 |
| 393.117 | 56474.67 | 56501.35 | 57154.15 | 58369.63 | 56338.6 | 56002.8 | 56700.5 | 55439.05 | 57125.45 | 54948.89 | 54994.53 | 54695.89 | 54141.67 | 54268 |
| 393.2542 | 57252.67 | 57187.55 | 58449.1 | 59152.63 | 56882.85 | 56931.65 | 57501.2 | 55683.68 | 58205.8 | 55444.06 | 55316.42 | 54900.5 | 54260.33 | 54551 |
| 393.3915 | 57198.67 | 58293.5 | 59517.25 | 58619.89 | 57595.15 | 57813.7 | 58144.5 | 55714.85 | 58068.35 | 55834.28 | 55362.42 | 55048.06 | 54244.67 | 54686 |
| 393.5288 | 56227.17 | 59552.1 | 60195.65 | 57681.32 | 58509.75 | 58102.55 | 59401.1 | 55568.65 | 57476.45 | 55854.44 | 55205.32 | 55256.11 | 54183.17 | 54494 |
| 393.6661 | 55745.17 | 59097.6 | 59123.8 | 56809.53 | 58535.2 | 57688.35 | 59215.2 | 55383.1 | 57081.25 | 55602.89 | 55000.74 | 55222.44 | 54119.17 | 54106 |
| 393.8033 | 55514.5 | 58055.5 | 58147.3 | 56437.32 | 57557.85 | 57003.4 | 58355.55 | 55138.18 | 56311.25 | 55465.39 | 54701.16 | 54938.28 | 54070.33 | 54234 |
| 393.9406 | 55012.5 | 57118.45 | 57105.55 | 56123.79 | 56894.55 | 56525.65 | 57194.7 | 55026.43 | 55932.8 | 55374.22 | 54736 | 54728.83 | 54144.83 | 54442 |
| 394.0779 | 55275.83 | 56512.65 | 56633.25 | 55839.58 | 56342.55 | 56315.2 | 56401.4 | 54809.1 | 55618.35 | 55116.28 | 54607.32 | 54671.83 | 54133.17 | 54158 |
| 394.2152 | 55234.67 | 56382.95 | 56582.6 | 55707.26 | 56050.65 | 55764.5 | 56274.3 | 54754.48 | 55475.55 | 55009.28 | 54677.89 | 54502.83 | 53997.33 | 54474 |
| 394.3525 | 55087 | 56249.9 | 56503.95 | 55851.42 | 55833.45 | 55522.9 | 56274.65 | 54864.93 | 55449.35 | 54867.94 | 54774.95 | 54498.67 | 54129.5 | 54064 |
| 394.4898 | 55062.83 | 56150.7 | 56386.55 | 55765.32 | 55958.35 | 55636.7 | 56235.35 | 54841.35 | 55496.55 | 54756.56 | 54701.11 | 54351.33 | 54199.83 | 54073 |
| 394.627 | 55057.5 | 56023.15 | 56530.15 | 55862.84 | 56055.75 | 55540.8 | 56073.1 | 54848.85 | 55553.7 | 54936.72 | 54736.26 | 54378.11 | 54012.83 | 54276 |
| 394.7643 | 55321.83 | 55974.7 | 56387.9 | 55937.32 | 56164.2 | 55412.9 | 55911.2 | 54772.23 | 55424.25 | 54810.17 | 54621.58 | 54573.72 | 54029.17 | 54487 |
| 394.9016 | 55051.67 | 56015.8 | 56275.4 | 55857.53 | 55960.6 | 55252.15 | 55748.3 | 54803.33 | 55550.75 | 54847.61 | 54563.84 | 54550.44 | 54289.17 | 54117 |
| 395.0389 | 54869.5 | 56058.85 | 56269.5 | 55732.58 | 55844.8 | 55266.4 | 56052.95 | 54851.03 | 55624.95 | 54871.28 | 54659 | 54623.83 | 54075.33 | 53928 |
| 395.1761 | 54991.83 | 55927.8 | 56264.95 | 55709.11 | 55845.05 | 55414 | 56082.1 | 54844.8 | 55376.9 | 54892.61 | 54460.11 | 54513.89 | 54071.67 | 53826 |
| 395.3134 | 55102.67 | 55948.25 | 56412.6 | 55885.79 | 56027.5 | 55610.25 | 56098.95 | 54701.15 | 55467.4 | 54888.94 | 54510.89 | 54547.61 | 54075.33 | 53952 |
| 395.4507 | 55202.33 | 55947.05 | 56109.7 | 55798.58 | 55838.9 | 55535.3 | 55974.3 | 54764.85 | 55497.2 | 54687.89 | 54561.95 | 54412.11 | 54095.83 | 54105 |
| 395.588 | 55428.5 | 55984.7 | 56189.6 | 55735.37 | 55853 | 55446.45 | 56025.55 | 54786.33 | 55605.05 | 54722.94 | 54612.89 | 54462.89 | 54093.17 | 54185 |
| 395.7252 | 55137.33 | 56021.55 | 55963.7 | 55817.58 | 55740.65 | 55301.5 | 56110.95 | 54798 | 55605.3 | 54740.39 | 54466.21 | 54552.67 | 53941 | 54020 |
| 395.8625 | 55097.5 | 56099.15 | 56024.3 | 55914.37 | 55827.55 | 55226.75 | 55998.65 | 54668.05 | 55607.1 | 54747.83 | 54605.74 | 54767.22 | 54012.83 | 54196 |
| 395.9998 | 55382 | 56031.4 | 56242.65 | 55891.32 | 55754.45 | 55381.8 | 56034.8 | 54643.75 | 55878.1 | 54794.72 | 54698.84 | 54668.39 | 53988.83 | 54214 |
| 396.1371 | 55449.17 | 56257.45 | 56290.2 | 56189 | 55901.9 | 55293.55 | 55967.15 | 54704.48 | 55815 | 54776.83 | 54721.05 | 54611.11 | 54047.5 | 54093 |
| 396.2743 | 55479.17 | 56339.45 | 56252.7 | 56456.68 | 55737.2 | 55290.3 | 55900.95 | 54804.88 | 55872.7 | 54937.5 | 54599 | 54587.5 | 54056.33 | 54197 |
| 396.4116 | 55220.83 | 56436.25 | 56430.1 | 56535.53 | 55836.85 | 55343.2 | 55978.4 | 54877.35 | 56100.5 | 55172.39 | 54580.63 | 54797.5 | 54173.67 | 54156 |
| 396.5489 | 55673.67 | 56251.75 | 56683.1 | 57078.11 | 56120.9 | 55690.45 | 56221.25 | 55000.28 | 56711.9 | 55349.61 | 54898.16 | 54902.11 | 54092.17 | 54497 |
| 396.6861 | 56175 | 56808.4 | 57111.2 | 57530.95 | 56533.05 | 56063.85 | 56715.1 | 55150.58 | 57124.85 | 55156.78 | 54816.84 | 55103.11 | 54042.17 | 54972 |
| 396.8234 | 56300 | 57178.25 | 57954.15 | 57805.68 | 57042.65 | 56617.7 | 57224 | 55346.73 | 57381.1 | 55417.17 | 54914.37 | 54919 | 54146.5 | 54249 |
| 396.9607 | 56080.33 | 57741.15 | 58687.15 | 57482.79 | 57617.5 | 57009.7 | 57669.2 | 55397.03 | 57018.4 | 55341.22 | 54985.95 | 54907 | 54184.5 | 54433 |
| 397.0979 | 55842.33 | 57867.15 | 58649.85 | 56824.37 | 57591.35 | 57228.7 | 57840.2 | 55230.78 | 56535.7 | 55418.33 | 54812.63 | 54948.06 | 54340.33 | 54099 |
| 397.2352 | 55593.67 | 57480.4 | 57778.9 | 56315.74 | 57265.05 | 56556.15 | 57682.1 | 55140.13 | 56058.9 | 55403.89 | 54808.47 | 54892.89 | 54172.33 | 53921 |
| 397.3725 | 55212.67 | 57034.6 | 56945.3 | 55943.53 | 56712.25 | 55867.1 | 57167.8 | 54932.55 | 55682 | 55073.94 | 54740.47 | 54739.22 | 54088.5 | 53848 |
| 397.5097 | 54983 | 56494.4 | 56416.75 | 55722.21 | 56327.8 | 55674.55 | 56398.65 | 54780.93 | 55553.95 | 54849.11 | 54619.42 | 54682.33 | 54236.67 | 53784 |
| 397.647 | 55138.17 | 56336.2 | 56451.25 | 55604 | 56257.3 | 55683.15 | 56205.6 | 54774.8 | 55463.75 | 55006.56 | 54534 | 54563.67 | 54406.33 | 53760 |
| 397.7843 | 55244 | 56079.4 | 56069.8 | 55860.21 | 56104.65 | 55443.8 | 55859.7 | 54769.85 | 55406.75 | 54841.11 | 54492.37 | 54557.67 | 54190.67 | 53817 |
| 397.9215 | 55154.67 | 56402.25 | 56198.15 | 55658.79 | 55856.9 | 55271.2 | 55952 | 54679.58 | 55337.45 | 54723.5 | 54588.32 | 54408.61 | 54107.17 | 53918 |
| 398.0588 | 54826.33 | 56000.65 | 56135.9 | 55863.63 | 55717.45 | 55327.15 | 55702.4 | 54637.1 | 55440.55 | 54753.67 | 54596.53 | 54391.67 | 54011.17 | 53850 |
| 398.1961 | 54753.33 | 55657.5 | 55818.45 | 55797.26 | 55496.5 | 55429.25 | 55749.65 | 54699.23 | 55308 | 54777.67 | 54406.95 | 54534.56 | 54120.33 | 53845 |
| 398.3333 | 54561.67 | 55750.3 | 55958.35 | 55522.95 | 55645.3 | 55269.7 | 55694.05 | 54545.28 | 55253.85 | 54830.39 | 54384.89 | 54384.5 | 54200.17 | 53809 |
| 398.4706 | 54885.5 | 55663.4 | 55878.85 | 55408.11 | 55796.5 | 55242.3 | 55882.55 | 54539.1 | 55241.2 | 54844.61 | 54482.47 | 54320.17 | 54118.17 | 53944 |
| 398.6079 | 54624.5 | 55661 | 55801.1 | 55500.79 | 55867.85 | 55197.15 | 55946.3 | 54478.25 | 55152.55 | 54777.39 | 54466.79 | 54515.17 | 54171.17 | 53952 |
| 398.7451 | 55005.33 | 55775.1 | 56039.8 | 55505.95 | 55694.8 | 55324.85 | 55880.05 | 54493.08 | 55212.8 | 54846.56 | 54395.42 | 54610.78 | 54214 | 53945 |
| 398.8824 | 54806.17 | 55705.9 | 55877.6 | 55465.37 | 55668.9 | 55280.6 | 55741.7 | 54560.13 | 55254.75 | 54752.94 | 54523.16 | 54585.72 | 54216.17 | 54019 |
| 399.0196 | 54790 | 55659.75 | 55884.15 | 55586.79 | 55456.65 | 55292.65 | 55584.9 | 54577.98 | 55059.65 | 54614.56 | 54573 | 54581.67 | 54265.17 | 54088 |
| 399.1569 | 54712.83 | 55637.25 | 55825.15 | 55420.32 | 55423.95 | 55155.1 | 55518.2 | 54582.63 | 55161.15 | 54540.61 | 54401.21 | 54564.11 | 54222.5 | 54147 |
| 399.2941 | 54889.67 | 55611.15 | 55714.7 | 55467.16 | 55489.3 | 55102.75 | 55562.65 | 54520.98 | 55217.3 | 54588.22 | 54391.21 | 54489.56 | 54185.33 | 54494 |
| 399.4314 | 54796.17 | 55761.8 | 55808.8 | 55537.58 | 55610.1 | 55095 | 55475.95 | 54529.55 | 55163.1 | 54703.17 | 54364 | 54497.94 | 53974.67 | 54604 |
| 399.5686 | 54669.83 | 55686.6 | 55984.7 | 55424.11 | 55536 | 55017.95 | 55589.5 | 54581.85 | 55220.55 | 54691.78 | 54293.68 | 54554.11 | 54082.33 | 54218 |
| 399.7059 | 54648.5 | 55616.4 | 55861.8 | 55722.37 | 55383 | 55218.85 | 55601 | 54594.6 | 55331.6 | 54656.78 | 54375.53 | 54548.39 | 54252.17 | 54262 |
| 399.8431 | 55081.17 | 55509.35 | 55851.3 | 55618.32 | 55570.2 | 55181 | 55631.9 | 54652.43 | 55368.75 | 54761.28 | 54359.58 | 54457.28 | 54438.83 | 55165 |
| 399.9804 | 54716.83 | 55880.5 | 55911.4 | 55501.37 | 55557.65 | 55011.25 | 55601.65 | 54684.48 | 55228.35 | 54743.06 | 54546.84 | 54514.72 | 54094.17 | 54636 |
| 400.1176 | 54786.33 | 55981.35 | 55948.9 | 55621.89 | 55833.75 | 55177.15 | 55527.85 | 54631.13 | 55195.85 | 54695.83 | 54442.37 | 54440.56 | 54081 | 54815 |
| 400.2549 | 55104.33 | 55857 | 56072.3 | 55717.42 | 55657 | 55188.4 | 55535.25 | 54550.93 | 55214.45 | 54664.56 | 54492.42 | 54402.44 | 54126.33 | 55246 |
| 400.3921 | 55016.5 | 55709.3 | 56083.45 | 55685.26 | 55757.85 | 55060.55 | 55440.9 | 54621 | 55176.05 | 54744.5 | 54530.68 | 54595.61 | 54054.67 | 55029 |
| 400.5294 | 55045.33 | 55532.25 | 55971.1 | 55491.37 | 55735.3 | 55267.4 | 55600.4 | 54691.95 | 55339.9 | 54733.72 | 54531.47 | 54561.11 | 54172.5 | 54780 |
| 400.6666 | 55051.33 | 55836.3 | 55811.35 | 55526.32 | 55714.25 | 55442.95 | 55573.25 | 54555.88 | 55239.2 | 54670.28 | 54575.68 | 54418 | 54222 | 54317 |
| 400.8038 | 54764.17 | 56075.1 | 55960.55 | 55583.37 | 55490.4 | 55197.15 | 55668.45 | 54598.05 | 55429.5 | 54679.11 | 54534.95 | 54458.56 | 54219.17 | 54147 |
| 400.9411 | 54638.5 | 55922.75 | 56102.25 | 55726.89 | 55718.5 | 55349.45 | 55672.25 | 54615.05 | 55455.4 | 54656.11 | 54656.21 | 54492.44 | 54053.33 | 54114 |
| 401.0783 | 54720.5 | 55907.3 | 56002.6 | 55725.53 | 56132.05 | 55414.3 | 55755.6 | 54722.35 | 55487.65 | 54707.78 | 54557.84 | 54513.72 | 54212.5 | 54121 |
| 401.2156 | 54992.17 | 55713.05 | 56109.3 | 55638.11 | 55902.35 | 55405.75 | 55854.9 | 54719.2 | 55336.95 | 54813.06 | 54489.89 | 54523.17 | 54238.5 | 54321 |
| 401.3528 | 55210.5 | 55813.9 | 55933.1 | 55831.58 | 55517.4 | 55291.55 | 55716.5 | 54600.73 | 55436.7 | 54778.39 | 54505.26 | 54502.67 | 54386.67 | 54620 |
| 401.49 | 55410.83 | 55864.9 | 55967.65 | 55837.47 | 55496.1 | 55410.75 | 55814.4 | 54606.33 | 55435.7 | 54672 | 54550.47 | 54541.17 | 54219.17 | 54748 |
| 401.6272 | 55264.67 | 55762.2 | 55932.4 | 55657.84 | 55713.55 | 55323.4 | 55785.35 | 54623.4 | 55570.05 | 54642.61 | 54487.63 | 54549.94 | 54159 | 54258 |
| 401.7645 | 54827.83 | 55677.6 | 56087.1 | 55740.11 | 55655.2 | 55498.1 | 55722.9 | 54784.38 | 55472.6 | 54813.83 | 54516.89 | 54673.39 | 54295.83 | 54244 |
| 401.9017 | 54562 | 55569.9 | 55891.2 | 55585.16 | 55550.15 | 55490.3 | 55615.55 | 54824.98 | 55554.05 | 54818.94 | 54496.42 | 54531.89 | 54144.33 | 53920 |
| 402.0389 | 54610 | 55853.75 | 55959.35 | 55690.16 | 55505.15 | 55460.2 | 55866.05 | 54766.2 | 55424.5 | 54813.61 | 54514.95 | 54625.5 | 54182 | 54350 |
| 402.1762 | 54718.17 | 55836.55 | 55899.5 | 55609.11 | 55687.65 | 55472.35 | 55746.4 | 54622.23 | 55420.8 | 54785.11 | 54525.42 | 54658.83 | 54077.83 | 54517 |
| 402.3134 | 55118.67 | 55823.15 | 56260.45 | 55659.16 | 55760.15 | 55273.4 | 55882.5 | 54705.58 | 55524.6 | 54816.61 | 54532.63 | 54569 | 54067.67 | 54205 |
| 402.4506 | 55168.17 | 56021.5 | 56298.55 | 55545.16 | 55946.85 | 55415 | 55866.65 | 54635.95 | 55630.8 | 54855.33 | 54509.68 | 54566.72 | 54133.17 | 54220 |
| 402.5878 | 55316.67 | 56018.55 | 56235.6 | 55742.37 | 55864.25 | 55521.35 | 55913.25 | 54672.98 | 55647.6 | 54863.78 | 54700.58 | 54506.83 | 54159.83 | 54771 |
| 402.725 | 55207.17 | 56007.5 | 56095.8 | 55983 | 55955.15 | 55423.25 | 56048.3 | 54800.65 | 55600.35 | 54981.39 | 54726.58 | 54617.61 | 54151.67 | 54988 |
| 402.8622 | 55342.83 | 56117.85 | 56674.55 | 56288.26 | 56047.5 | 55585.4 | 56282.8 | 54934.5 | 55738.25 | 54908.28 | 54830.42 | 54615.89 | 53941.83 | 54624 |
| 402.9995 | 55528.5 | 56466.3 | 56920.95 | 56690 | 56328.5 | 55933.2 | 56223.05 | 55157.15 | 56093.45 | 55283.06 | 54965.16 | 54892.33 | 54106 | 54746 |
| 403.1367 | 55558.17 | 56534.35 | 56793.15 | 56826.16 | 56751.05 | 55994.55 | 56556.1 | 55147 | 56152.6 | 55241.22 | 55140.37 | 54919.67 | 54293.67 | 55103 |
| 403.2739 | 55695.33 | 56815.35 | 56954.75 | 56727.68 | 56887.8 | 56172.1 | 57030.9 | 55371.03 | 56241.7 | 55511.89 | 55049.79 | 54912.67 | 54259.83 | 55214 |
| 403.4111 | 55620.17 | 57244.15 | 57487.3 | 56365.21 | 57029.5 | 56349.15 | 57171.8 | 55326.23 | 56168.15 | 55260.17 | 55120 | 54937.61 | 54194 | 54594 |
| 403.5483 | 55524.83 | 56806.3 | 57446 | 56259.42 | 56996.2 | 56877.95 | 56935.9 | 55209.95 | 56197.5 | 55223.28 | 55017.63 | 54916.56 | 54241.83 | 54298 |
| 403.6855 | 55052.33 | 56740.55 | 56967.8 | 56227.42 | 56735.85 | 56380.7 | 56823.05 | 55108.9 | 55849.35 | 55199.39 | 54878.84 | 54738.39 | 54220.83 | 54461 |
| 403.8227 | 55203.67 | 56448.8 | 56419.15 | 56004.42 | 56369.5 | 56031.95 | 56511.2 | 54827.9 | 55529.8 | 54918.94 | 54743.68 | 54694.61 | 54451.33 | 54086 |
| 403.9599 | 55260.83 | 56286 | 56593.85 | 55948.26 | 56035.7 | 55876.05 | 56086.05 | 54763.9 | 55621.7 | 55031.72 | 54615.74 | 54682.5 | 54058.5 | 54076 |
| 404.0971 | 54848.33 | 56181.65 | 56082.65 | 55629.63 | 56032.25 | 55439.35 | 55857.45 | 54639.18 | 55464.15 | 54923.5 | 54518.16 | 54673.72 | 54193.83 | 53923 |
| 404.2343 | 55093.33 | 56102 | 55864.95 | 55581.11 | 55788.1 | 55311.15 | 55819 | 54659.25 | 55415.75 | 54875.39 | 54437.42 | 54647.56 | 54104.33 | 54631 |
| 404.3715 | 54893.5 | 55797.4 | 55819.45 | 55536.26 | 55801.2 | 55323.35 | 55825.95 | 54639.85 | 55392.15 | 54693.06 | 54462.47 | 54511.33 | 54205.5 | 54575 |
| 404.5087 | 54797.83 | 55594.45 | 56014.15 | 55703.47 | 55799.45 | 55268.45 | 55667.55 | 54717.15 | 55496.75 | 54727 | 54437.84 | 54589.61 | 54031.67 | 53949 |
| 404.6458 | 55168.67 | 55675.55 | 55969.1 | 55480.63 | 55711.1 | 55450.2 | 55737.9 | 54793.25 | 55311.35 | 54667.11 | 54370.79 | 54630.56 | 54026 | 54528 |
| 404.7831 | 55088.83 | 55809.8 | 55892.35 | 55436.21 | 55732.95 | 55550.5 | 55851.65 | 54722.43 | 55265.6 | 54725.06 | 54357.95 | 54494.22 | 54211.5 | 54462 |
| 404.9202 | 55111.83 | 55741.05 | 56116.85 | 55476.63 | 55683.6 | 55419 | 55544.7 | 54715.63 | 55056.2 | 54677.83 | 54523.47 | 54540.67 | 54164.83 | 53993 |
| 405.0574 | 55221.33 | 55688.3 | 55986.55 | 55585.58 | 55670.25 | 55312.85 | 55771.65 | 54657.35 | 55113.5 | 54558.72 | 54486.79 | 54654.5 | 54038.5 | 54088 |
| 405.1946 | 55437.17 | 55722.75 | 55759.3 | 55288.68 | 55593.7 | 55268.7 | 55792.75 | 54677.8 | 55112 | 54616.78 | 54587.42 | 54475.94 | 54264.5 | 55416 |
| 405.3318 | 55469 | 55596.1 | 55746.95 | 55423.32 | 55578.65 | 55104.15 | 55774 | 54628.63 | 55106.1 | 54597.94 | 54574.32 | 54522.72 | 54070.33 | 54143 |
| 405.4689 | 55338 | 55663.85 | 55911.55 | 55423.79 | 55507.75 | 55165.75 | 55564.15 | 54664.73 | 55296.85 | 54662.56 | 54581 | 54630.89 | 54047 | 54058 |
| 405.6061 | 55164 | 55904.65 | 55903.1 | 55642.53 | 55782.55 | 55097.1 | 55691.6 | 54653.25 | 55210.45 | 54684.06 | 54543.74 | 54529.61 | 54175.17 | 54163 |
| 405.7433 | 54955 | 55626.3 | 55931.7 | 55663.95 | 55727.1 | 55186.45 | 55802.6 | 54668.28 | 55221.35 | 54660.72 | 54620.74 | 54541.83 | 54073.17 | 54324 |
| 405.8804 | 55169.67 | 55600.65 | 56099.05 | 55499.32 | 55733.55 | 55224.3 | 55610.55 | 54685.43 | 55358.2 | 54700.61 | 54501.63 | 54565.72 | 54227.5 | 54615 |
| 406.0176 | 54985 | 55805.4 | 55785.3 | 55551.95 | 55769.75 | 55235.95 | 55627.9 | 54628.1 | 55427.75 | 54824.94 | 54439 | 54602.89 | 54157 | 53965 |
| 406.1548 | 55082.17 | 55774.05 | 55988.55 | 55651.47 | 55722.25 | 55224.8 | 55810.75 | 54767.58 | 55401.65 | 54659.39 | 54460.26 | 54573.22 | 53962.83 | 54559 |
| 406.2919 | 55182.5 | 55741.2 | 56289.7 | 55742 | 55642.7 | 55225.7 | 55833.55 | 54802.28 | 55482.35 | 54738.5 | 54431.53 | 54491.33 | 54072.67 | 54305 |
| 406.4291 | 54944.17 | 55674 | 55972.45 | 55641.58 | 55725 | 55299.5 | 55657.85 | 54675.05 | 55539.65 | 54752.94 | 54441.26 | 54623.39 | 54126 | 53935 |
| 406.5663 | 54934.67 | 55640.3 | 55894.35 | 55708.47 | 55736.3 | 55237.35 | 55735.5 | 54614.33 | 55502.7 | 54723.5 | 54574.68 | 54571.83 | 54199.17 | 54278 |
| 406.7034 | 55308.33 | 55630.05 | 56073.8 | 55800.95 | 55555.75 | 55055.7 | 55762.6 | 54671.33 | 55318.5 | 54745.56 | 54581.26 | 54508.28 | 54107 | 54451 |
| 406.8406 | 54992.67 | 55915 | 55799 | 55563.21 | 55565.3 | 54932.15 | 55650.25 | 54681.83 | 55120.35 | 54694.94 | 54418.26 | 54504.61 | 54056 | 54277 |
| 406.9777 | 55043.5 | 55727.95 | 55846.85 | 55473.32 | 55386.35 | 55109.9 | 55713.8 | 54581.98 | 55120.65 | 54665.5 | 54470.26 | 54451.67 | 54038.17 | 54253 |
| 407.1148 | 55064.5 | 55568.45 | 55930.7 | 55512.58 | 55479 | 55150 | 55577.2 | 54585.8 | 55201.75 | 54529.11 | 54551.32 | 54419.61 | 54034.83 | 54648 |
| 407.252 | 54877.67 | 55721.8 | 55781.1 | 55612.84 | 55543 | 55141.35 | 55533.45 | 54653.03 | 55259.3 | 54662.39 | 54686.53 | 54586.28 | 54139.17 | 54200 |
| 407.3891 | 54993.5 | 55752.3 | 55791.9 | 55705.53 | 55556.6 | 55130.9 | 55584.15 | 54581.05 | 55313.7 | 54656.28 | 54470.84 | 54610.22 | 54296.17 | 54224 |
| 407.5263 | 55083.67 | 55781.9 | 55911.65 | 55849.63 | 55607.05 | 55157.05 | 55576.3 | 54528.4 | 55236.2 | 54623.06 | 54453.16 | 54495 | 53977 | 54539 |
| 407.6634 | 54845.17 | 55744.45 | 55932.65 | 55766.26 | 55769.9 | 55223.85 | 55679.05 | 54704.83 | 55317.25 | 54597.17 | 54484.95 | 54336.72 | 54015.5 | 54147 |
| 407.8005 | 54887 | 55725.25 | 56023.85 | 55612.05 | 55656 | 55255.45 | 55691.55 | 54615.18 | 55174.15 | 54733.17 | 54494.21 | 54418.33 | 54130 | 54049 |
| 407.9377 | 54918.83 | 55786.3 | 55910.85 | 55349.11 | 55536.85 | 55178.55 | 55746.6 | 54683.23 | 55098.25 | 54727.56 | 54531.21 | 54613 | 54147.17 | 54183 |
| 408.0748 | 54762.83 | 55893.1 | 55872.75 | 55506.58 | 55747.85 | 55064.45 | 55656.85 | 54585.65 | 55034.7 | 54727.28 | 54422 | 54498.78 | 54137 | 54263 |
| 408.2119 | 54693.5 | 55734.25 | 55866.75 | 55440.05 | 55570.7 | 55190.4 | 55481 | 54503.63 | 55094.15 | 54751.56 | 54508.95 | 54355.61 | 54013 | 54683 |
| 408.349 | 54929.5 | 55723.75 | 55937 | 55400.68 | 55554.45 | 55264.65 | 55588.45 | 54557.5 | 55172.6 | 54516.83 | 54367.26 | 54475.22 | 53986.83 | 54296 |
| 408.4862 | 54720.83 | 55740.65 | 55888.85 | 55479.26 | 55820.95 | 55292.95 | 55581.65 | 54565.73 | 55204.4 | 54575.28 | 54358.32 | 54595.61 | 54081.5 | 54474 |
| 408.6233 | 54757.33 | 55795.85 | 55749.45 | 55465.21 | 55747.2 | 55313.45 | 55719.45 | 54584.3 | 55296.75 | 54655.28 | 54411.37 | 54556.61 | 54031.17 | 54518 |
| 408.7604 | 54878.5 | 55802.25 | 55718.55 | 55649.47 | 55569.25 | 55072.2 | 55583.25 | 54704.25 | 55356.2 | 54648.39 | 54511.95 | 54451.89 | 54007 | 55495 |
| 408.8975 | 54769.17 | 55917.45 | 55796.9 | 55545.16 | 55573.65 | 55181.05 | 55648.2 | 54593.73 | 55119.65 | 54638.33 | 54521.68 | 54400.5 | 54178.5 | 55316 |
| 409.0346 | 54836.5 | 55708.2 | 55841.5 | 55632.05 | 55656.4 | 55160.85 | 55598.15 | 54595.13 | 55176.3 | 54619.39 | 54511.05 | 54421 | 53929.83 | 54699 |
| 409.1717 | 54505.83 | 55608.25 | 55741.35 | 55846.11 | 55348.35 | 55039.6 | 55581.7 | 54540.08 | 55218.75 | 54707.94 | 54564.68 | 54351.06 | 53879.67 | 54217 |
| 409.3088 | 54483.67 | 55699.2 | 55818.15 | 55708.58 | 55632.95 | 55050.4 | 55657.6 | 54527.98 | 55556.2 | 54697.39 | 54624.47 | 54396.5 | 53981.33 | 54126 |
| 409.4459 | 54457.5 | 55742.15 | 55872.05 | 55596.11 | 55639.95 | 55068.65 | 55374.45 | 54666.6 | 55452 | 54613.83 | 54470.79 | 54342.06 | 54036.5 | 54127 |
| 409.583 | 54643.33 | 55710.15 | 55713.5 | 55584.58 | 55564.85 | 54986.45 | 55385.35 | 54727.48 | 55381.55 | 54794.11 | 54551.05 | 54489.06 | 54093 | 53997 |
| 409.7201 | 54630.33 | 55623.45 | 55835.1 | 55685.42 | 55582.15 | 55031.65 | 55704.55 | 54607.43 | 55281.85 | 54880.72 | 54612.63 | 54412.33 | 54132 | 53955 |
| 409.8572 | 54711.67 | 55681.6 | 55991.65 | 55529.21 | 55659.2 | 55071.2 | 55492.85 | 54651.58 | 55265.45 | 54745.56 | 54505.16 | 54447.61 | 54181.17 | 54346 |
| 409.9942 | 54964.83 | 55615.9 | 56065.6 | 55658.47 | 55813.95 | 55003.25 | 55404.25 | 54673.8 | 55374.85 | 54764 | 54392.84 | 54388.44 | 54438.83 | 54462 |
| 410.1313 | 55339.33 | 55469.8 | 56095.95 | 55537.74 | 55586.55 | 55050.8 | 55502.15 | 54659.68 | 55465.15 | 54829.39 | 54527.74 | 54478.61 | 54259 | 53953 |
| 410.2684 | 55213.83 | 55516.3 | 56010.75 | 55320.95 | 55463.85 | 55186.1 | 55459.9 | 54492.78 | 55383.95 | 54873.17 | 54514.32 | 54516.44 | 54172.5 | 53874 |
| 410.4055 | 54893.17 | 55359.7 | 55819.2 | 55379.89 | 55299.2 | 55070.15 | 55532.75 | 54521.78 | 55172.05 | 54705.61 | 54415.95 | 54585.44 | 54136.17 | 53998 |
| 410.5425 | 54750 | 55652.85 | 55947.45 | 55187.89 | 55420.55 | 55289.1 | 55434.35 | 54545.48 | 55268.6 | 54628.22 | 54379.89 | 54516.94 | 54250.33 | 54451 |
| 410.6796 | 54999.83 | 55552.45 | 55884.8 | 55157.53 | 55501.3 | 55385.3 | 55387.55 | 54586.4 | 55398.15 | 54552.83 | 54360.05 | 54558.33 | 54232.67 | 54592 |
| 410.8167 | 54900 | 55608.95 | 56092.5 | 55299.89 | 55504.9 | 55175.75 | 55386.9 | 54731.03 | 55246.15 | 54764.06 | 54380.32 | 54561.94 | 54222.83 | 54184 |
| 410.9537 | 54995.67 | 55692.15 | 55775.2 | 55289.26 | 55418.9 | 55370 | 55306.3 | 54574.85 | 55255.7 | 54642.22 | 54422.21 | 54583.44 | 54103.17 | 54726 |
| 411.0908 | 55035.83 | 55366.4 | 55686.7 | 55226.68 | 55385.4 | 55391.35 | 55390.05 | 54635.58 | 55074.45 | 54581.5 | 54500.63 | 54535.28 | 54220.67 | 54393 |
| 411.2278 | 55134.5 | 55505.85 | 55637.25 | 55522.74 | 55351.4 | 55361.75 | 55480.95 | 54594.95 | 55302.15 | 54698.78 | 54397.58 | 54425.5 | 54399 | 54010 |
| 411.3649 | 55439.83 | 55552.25 | 55785 | 55562.95 | 55501.15 | 55178.55 | 55651.55 | 54613.55 | 55370.55 | 54689.22 | 54466.11 | 54466.72 | 54349.17 | 54327 |
| 411.5019 | 55297.17 | 55487.45 | 55750.7 | 55486.26 | 55438.35 | 55196 | 55565.65 | 54572.05 | 55273.3 | 54689.44 | 54462.68 | 54656.83 | 54090.67 | 55206 |
| 411.639 | 54993.5 | 55593.75 | 55654.75 | 55719.11 | 55493.15 | 55163.9 | 55541.25 | 54557.58 | 55121.95 | 54773.56 | 54430.42 | 54507 | 54106.67 | 54815 |
| 411.776 | 54862.83 | 55550.1 | 55809.25 | 55471.89 | 55463.3 | 55399.05 | 55468 | 54564 | 55141.2 | 54694.78 | 54432 | 54518.89 | 54077 | 54195 |
| 411.913 | 54855.5 | 55584 | 55851.3 | 55415.53 | 55431.3 | 55204.3 | 55564.9 | 54609.43 | 55193 | 54640.94 | 54512.68 | 54586.06 | 54186 | 54186 |
| 412.0501 | 55126.33 | 55561.05 | 55801.8 | 55747.16 | 55546.6 | 54990.6 | 55531.1 | 54570.53 | 55400.05 | 54846.5 | 54484.21 | 54553.78 | 54150.67 | 54266 |
| 412.1871 | 55041.17 | 55765.1 | 55961.05 | 55549.53 | 55650.75 | 55151.4 | 55489.5 | 54665.35 | 55317.6 | 54837.5 | 54498.32 | 54484.72 | 54190.17 | 54139 |
| 412.3241 | 55293.33 | 55752.35 | 56021.8 | 55517 | 55479.45 | 55350.3 | 55489.3 | 54701.75 | 55451.25 | 54807.83 | 54548.68 | 54567.11 | 54226.67 | 54403 |
| 412.4611 | 55448.67 | 55732.6 | 55828.65 | 55403.21 | 55463.85 | 55272.75 | 55460.8 | 54700.48 | 55283.95 | 54816.72 | 54562.21 | 54452.61 | 54153.83 | 53935 |
| 412.5981 | 55424.83 | 55686.25 | 56063.05 | 55871.42 | 55606.7 | 55152.95 | 55798.3 | 54673.38 | 55496.45 | 54845.78 | 54528 | 54490 | 54067.83 | 53789 |
| 412.7352 | 55283.83 | 55767.7 | 56069.3 | 55808.21 | 55602.65 | 55321 | 55955.9 | 54728.35 | 55543.5 | 54822.61 | 54395.21 | 54504.06 | 53937.83 | 54020 |
| 412.8722 | 55050.5 | 55832.75 | 56246.1 | 55776.26 | 55771.2 | 55403.05 | 55954.4 | 54683.48 | 55413.1 | 54754.94 | 54524.05 | 54456.33 | 54089.67 | 54076 |
| 413.0092 | 55078.5 | 55997.95 | 56229.35 | 55749.11 | 55562.65 | 55444.4 | 55977 | 54695.45 | 55324.45 | 54755.28 | 54586.16 | 54551.33 | 53965.33 | 53994 |
| 413.1462 | 55413.83 | 56039.9 | 56141.25 | 55518 | 55834.85 | 55282.25 | 55933.3 | 54702.85 | 55423.65 | 54883.28 | 54575.63 | 54547.56 | 54015.5 | 53939 |
| 413.2832 | 55288.5 | 56007.65 | 56115.75 | 55717.68 | 55683.15 | 55559.05 | 55915.85 | 54764.2 | 55462 | 54788.39 | 54595.37 | 54566.33 | 54080 | 54495 |
| 413.4202 | 54979.5 | 55972.85 | 56237.55 | 55802.42 | 55654.05 | 55520.1 | 55777.85 | 54671.85 | 55464.25 | 54838.67 | 54527.63 | 54627.28 | 54173 | 54461 |
| 413.5572 | 54979.17 | 55784.85 | 56152.15 | 55760.16 | 55805.35 | 55294.25 | 55745.45 | 54639.85 | 55386.8 | 54779.22 | 54611.79 | 54736.61 | 54210.83 | 54185 |
| 413.6941 | 54798.83 | 55922 | 56146.2 | 55838.11 | 55794.15 | 55257.55 | 55914.45 | 54749.73 | 55513.2 | 54660.5 | 54530.11 | 54739.72 | 54010.83 | 54201 |
| 413.8311 | 55168.5 | 56018.85 | 56171 | 55665.26 | 55525.5 | 55318.2 | 55741.35 | 54684.9 | 55370.45 | 54797.33 | 54472.79 | 54884.22 | 54283.67 | 54501 |
| 413.9681 | 55240 | 55912 | 55852.35 | 55409.89 | 55479.75 | 55297.7 | 55618.3 | 54784.6 | 55476.95 | 54816.89 | 54530.21 | 54532.67 | 54279.67 | 54064 |
| 414.1051 | 55303.83 | 56029.55 | 56004.45 | 55671.58 | 55518.3 | 55270.15 | 55758 | 54763.93 | 55500.45 | 54873.28 | 54470.32 | 54445 | 54047.67 | 54110 |
| 414.242 | 55167.5 | 56003.95 | 56157.4 | 55735.79 | 55969.5 | 55500.05 | 55841.3 | 54588.15 | 55580.05 | 54713.17 | 54476.37 | 54517 | 54053 | 54124 |
| 414.379 | 54842.83 | 55886.65 | 55992.4 | 55765.47 | 55984.6 | 55390.65 | 55823.85 | 54604.9 | 55422.8 | 54896.22 | 54484.05 | 54541.11 | 54049 | 54717 |
| 414.516 | 54950.83 | 56088.55 | 56189.05 | 55675.42 | 55682.3 | 55315.15 | 55896 | 54666.5 | 55362.65 | 54812.83 | 54545.42 | 54510.06 | 54144.5 | 54087 |
| 414.6529 | 54954.83 | 56072.85 | 56070.55 | 55714.05 | 55687.2 | 55355.2 | 55658.15 | 54597.93 | 55214.15 | 54783.28 | 54550.63 | 54547.17 | 53981.17 | 54390 |
| 414.7899 | 54596.67 | 55963.25 | 55918.75 | 55387.63 | 55882.85 | 55429.4 | 55534.5 | 54616.18 | 55358.8 | 54767.28 | 54478.79 | 54679.17 | 54095.83 | 54480 |
| 414.9268 | 54868.67 | 55683.7 | 56067 | 55554.74 | 55617.8 | 55457.15 | 55773.4 | 54574.03 | 55439.15 | 54779.33 | 54598.32 | 54651 | 54102 | 54169 |
| 415.0638 | 54689 | 55679.6 | 55907.85 | 55709.74 | 55555.7 | 55392.75 | 55687.7 | 54545.18 | 55396.3 | 54771 | 54633.53 | 54587.28 | 53984.33 | 54038 |
| 415.2007 | 54994.17 | 55629.2 | 55825.8 | 55558.26 | 55650.5 | 55423.75 | 55628.65 | 54635.38 | 55350.05 | 54685.5 | 54583.84 | 54448.17 | 54073.33 | 53921 |
| 415.3377 | 54692.67 | 55509.1 | 55985.05 | 55658.74 | 55566.55 | 55272.2 | 55516.1 | 54712.53 | 55535.3 | 54731.56 | 54457.26 | 54570.33 | 53913.83 | 53957 |
| 415.4746 | 54975.17 | 55574.9 | 55719.8 | 55490.58 | 55361.25 | 55297.4 | 55603.1 | 54605.95 | 55487.9 | 54702.06 | 54500.89 | 54693.89 | 53967.67 | 54116 |
| 415.6115 | 54999.33 | 55575 | 55604.25 | 55382.21 | 55460.65 | 55425.2 | 55694.9 | 54595.28 | 55472.3 | 54787.06 | 54608.21 | 54602.44 | 53981.5 | 54084 |
| 415.7484 | 54935.5 | 55424.15 | 55589.5 | 55407.79 | 55594.6 | 55249.95 | 55669.25 | 54625.88 | 55346.85 | 54676.89 | 54782 | 54661.33 | 54047.33 | 54037 |
| 415.8854 | 54817.5 | 55549.6 | 55652.1 | 55406.84 | 55489.1 | 55278.2 | 55699.7 | 54703.5 | 55218.65 | 54634.56 | 54547.16 | 54754.28 | 54035.5 | 54154 |
| 416.0223 | 54914.83 | 55551.7 | 55759.6 | 55308.53 | 55619.35 | 55182.9 | 55576.7 | 54633.6 | 55346.5 | 54766.94 | 54607.58 | 54863.67 | 54225.83 | 54282 |
| 416.1592 | 55032.33 | 55476.25 | 55845.15 | 55350.21 | 55602.8 | 55118.45 | 55611.5 | 54647.13 | 55313.15 | 54982.22 | 54696.58 | 54753.61 | 54096.67 | 53897 |
| 416.2961 | 55350.5 | 55447.55 | 55771.5 | 55238.68 | 55687.3 | 55047.85 | 55371.95 | 54686.7 | 55130.45 | 54880.22 | 54757.58 | 54622.22 | 53993.17 | 54211 |
| 416.433 | 55411 | 55617.9 | 55614.25 | 55431.79 | 55659.05 | 55162.5 | 55527.75 | 54638.58 | 55151.1 | 54696.17 | 54767.11 | 54672.72 | 53968.5 | 54348 |
| 416.5699 | 54916.83 | 55501.3 | 55825.95 | 55543.47 | 55604.3 | 55163.3 | 55404.1 | 54651.68 | 55344.35 | 54646.17 | 54741.84 | 54597 | 53985.17 | 54310 |
| 416.7068 | 54972.83 | 55534.55 | 55682.35 | 55431.74 | 55513.15 | 55175.75 | 55352.8 | 54686.1 | 55360.25 | 54803.06 | 54719.42 | 54538 | 54108.17 | 54649 |
| 416.8437 | 54955.83 | 55774.25 | 55784.55 | 55253.21 | 55488.9 | 54967.2 | 55671.65 | 54680.2 | 55396.35 | 54777.83 | 54697.58 | 54607.28 | 54330.33 | 54187 |
| 416.9805 | 55159.5 | 55803.15 | 55950.45 | 55522.79 | 55545.45 | 55286.45 | 55604.1 | 54752.63 | 55348.7 | 54739.72 | 54632.95 | 54723.17 | 54250.33 | 53959 |
| 417.1174 | 55143.83 | 56028.95 | 56120.55 | 55531.32 | 55469 | 55346.15 | 55645.5 | 54966.65 | 55340.15 | 55022.72 | 54643.63 | 54649.17 | 54193.5 | 54334 |
| 417.2543 | 55110.33 | 55883.25 | 56091.6 | 55644.47 | 55573.35 | 55459.85 | 55710.2 | 55075.35 | 55309.95 | 54882.28 | 54681.26 | 54661.28 | 54258 | 54272 |
| 417.3911 | 55070.67 | 55493.2 | 55984.5 | 55577.58 | 55589.15 | 55502.2 | 55784.4 | 54816.03 | 55465.7 | 54777.56 | 54541.68 | 54551.89 | 54252.67 | 54793 |
| 417.528 | 54993.33 | 55588.15 | 55810.75 | 55780.95 | 55560.05 | 55434.55 | 55659.85 | 54854.13 | 55555.6 | 54928.78 | 54757.74 | 54541.11 | 54408.17 | 54822 |
| 417.6649 | 55107.33 | 55810.8 | 55862.3 | 55710.84 | 55807.6 | 55556.75 | 55577.2 | 54741.98 | 55646.6 | 54974 | 54867.16 | 54753.5 | 54470 | 54545 |
| 417.8017 | 54954.67 | 55759.25 | 56028.3 | 55657.74 | 55785.3 | 55555.4 | 55662.45 | 54817.53 | 55495.65 | 55112.33 | 54849.63 | 54760.72 | 54619.67 | 54593 |
| 417.9386 | 55130.67 | 55748.75 | 55890 | 55709.37 | 55718.6 | 55443.4 | 55821.55 | 54905.63 | 55591.5 | 55050.56 | 54886.63 | 54751.78 | 54472.67 | 54453 |
| 418.0754 | 55595.67 | 55944.45 | 56061.05 | 55752.68 | 55745.9 | 55539.85 | 55929.2 | 54986.58 | 55713.55 | 54992.67 | 54778.16 | 54846.28 | 54428 | 54325 |
| 418.2123 | 55532.17 | 56083.3 | 56163.65 | 56037.89 | 55804.05 | 55437.55 | 55821 | 54914.73 | 55615.45 | 54996.89 | 54741.11 | 54821.94 | 54568.33 | 54421 |
| 418.3491 | 55542.67 | 55983.9 | 56181.3 | 56006.37 | 56063.45 | 55738.05 | 55789.55 | 54842.88 | 55467.8 | 55056.72 | 54780.84 | 54952.5 | 54303 | 54208 |
| 418.4859 | 55564.67 | 55936.55 | 56156.2 | 56177.16 | 55950.6 | 55824 | 55979.35 | 54935.1 | 55625 | 55132.89 | 54849.26 | 54905.83 | 54366.33 | 54328 |
| 418.6228 | 55398.33 | 56043.85 | 56366.05 | 56080.42 | 55822.85 | 55858.25 | 56025 | 54951.6 | 55629.65 | 55212.06 | 55008.26 | 54888 | 54313.5 | 54846 |
| 418.7596 | 55504.17 | 56072 | 56278.85 | 55976 | 55793 | 55843.8 | 56005.85 | 54868.68 | 55715.85 | 54959.89 | 54973.79 | 55120.33 | 54396.17 | 54461 |
| 418.8964 | 55385.67 | 55887.15 | 56046.2 | 55972.37 | 55875.85 | 55625 | 56121.55 | 54905.8 | 55970.15 | 55045.39 | 54895.21 | 55045.67 | 54150.67 | 54486 |
| 419.0332 | 55460.17 | 55957.5 | 56415.9 | 56156.89 | 55769.1 | 55620.45 | 56051.15 | 55068.78 | 55903.85 | 55217.5 | 55061.32 | 55201.22 | 54297.17 | 54766 |
| 419.17 | 55849.5 | 56165.5 | 56576.85 | 56043.89 | 55886.2 | 55682.95 | 55968.35 | 55068.23 | 55695.05 | 55254.44 | 55123.53 | 55158.33 | 54466.83 | 54567 |
| 419.3068 | 55561 | 56241.3 | 56436.1 | 56248.37 | 56110.05 | 55717.5 | 56162.35 | 55091.65 | 55893.55 | 55358.67 | 54910.37 | 55168.11 | 54491.5 | 54305 |
| 419.4436 | 55755.67 | 56298.25 | 56415.75 | 56434.79 | 56078.15 | 55986.15 | 56075.05 | 55151.13 | 55843.6 | 55287.06 | 55024.47 | 55254.61 | 54532.83 | 54778 |
| 419.5804 | 55596.5 | 56555.45 | 56214.9 | 56242.05 | 56120.85 | 55655 | 56210.4 | 55017.55 | 55919.8 | 55341.78 | 55098.79 | 55117.44 | 54395.33 | 54571 |
| 419.7172 | 55497.33 | 56522.65 | 56269.65 | 56261.11 | 55973.6 | 55790.65 | 56201.5 | 55072.23 | 55973.3 | 55398.17 | 55180.84 | 55132.39 | 54572.33 | 54693 |
| 419.854 | 55872.5 | 56543.2 | 56373.35 | 56262.89 | 56131.05 | 55752.15 | 56244 | 55135 | 56229.9 | 55452.33 | 55183.47 | 55210.28 | 54588.67 | 54702 |
| 419.9908 | 55249.67 | 56550.65 | 56325.5 | 56329.58 | 56304 | 55936.2 | 56275.95 | 55193 | 55965.45 | 55397.5 | 55293.74 | 55144.72 | 54769.67 | 54731 |
| 420.1275 | 55599.83 | 56567.4 | 56394.95 | 56505.84 | 56383.4 | 55943.45 | 56282.85 | 55142.08 | 55793.5 | 55288.83 | 55181.74 | 55187.78 | 55063.33 | 54546 |
| 420.2643 | 55585.67 | 56430.9 | 56392.45 | 56348.05 | 56468.9 | 56115.5 | 56277.5 | 55153.68 | 55856.75 | 55379.06 | 55208.16 | 54986.78 | 54725.83 | 54444 |
| 420.401 | 55891.83 | 56640.55 | 56583.95 | 56280.32 | 56388.6 | 55878.45 | 56293.2 | 55229.65 | 56159.15 | 55357 | 55291.05 | 55145 | 54650.33 | 54495 |
| 420.5378 | 55754.5 | 56616.9 | 56577.5 | 56301.42 | 56366.6 | 55840.75 | 56535.6 | 55350.08 | 56315.35 | 55577.17 | 55283.32 | 54988.33 | 54725.33 | 54466 |
| 420.6745 | 55683 | 56406 | 56793.75 | 56364.05 | 56562.1 | 55796.2 | 56404.2 | 55289.45 | 56010.75 | 55640.89 | 54999.89 | 55200.78 | 54814 | 54540 |
| 420.8113 | 56052.5 | 56633.35 | 56490.4 | 56391.42 | 56568.9 | 55766.6 | 56634.55 | 55225.7 | 56102.35 | 55531.44 | 55083.47 | 55346.94 | 54877.67 | 55062 |
| 420.948 | 55769.83 | 56573.4 | 56421.25 | 56444.47 | 56526.1 | 55911.7 | 56651.6 | 55299.3 | 56270.25 | 55267.33 | 55294.32 | 55194.44 | 54849 | 56108 |
| 421.0848 | 55659.17 | 56789 | 56724.2 | 56678.26 | 56506.55 | 56210.65 | 56596.25 | 55290.3 | 56314.9 | 55317.5 | 55378.32 | 55255.78 | 54862.5 | 55643 |
| 421.2215 | 55602.83 | 56629.6 | 56616.2 | 56804.26 | 56607.45 | 56272.8 | 56649.1 | 55401 | 56407.7 | 55513.44 | 55371.53 | 55247.11 | 54628.33 | 55118 |
| 421.3582 | 55710.17 | 56941.45 | 57002.65 | 56958.42 | 56456.55 | 56340.05 | 56741.35 | 55577.03 | 56498.95 | 55658.61 | 55487.21 | 55324 | 54622.5 | 55319 |
| 421.4949 | 56080 | 56893.3 | 57127.7 | 56934.32 | 56747.35 | 56410.75 | 56707.15 | 55594.68 | 56635.75 | 55735.67 | 55468.95 | 55274.22 | 54553.83 | 55380 |
| 421.6316 | 55924.67 | 57063.6 | 57256.15 | 57018.63 | 56740 | 56317.7 | 56776.6 | 55685.15 | 56510.25 | 55872.39 | 55483.68 | 55404.39 | 54804.33 | 55364 |
| 421.7683 | 56068.5 | 57110.45 | 57731.85 | 57131.05 | 56926.3 | 56509.25 | 57321.7 | 55856.35 | 56688.55 | 55904.89 | 55575.47 | 55451.83 | 54945.83 | 55700 |
| 421.905 | 56252 | 57386.2 | 57809.1 | 57374.68 | 57364.05 | 56756.3 | 57588.95 | 55955.13 | 56952.6 | 56001.5 | 55563.37 | 55681.33 | 54770.67 | 55622 |
| 422.0417 | 56765.33 | 57359.75 | 58247.95 | 58028.32 | 57609.2 | 57091.3 | 57633.2 | 56075.63 | 57393.2 | 56337.06 | 55867.74 | 55739.94 | 54915.17 | 55213 |
| 422.1784 | 57361 | 57858.1 | 58496.5 | 58874.37 | 58031.5 | 57470.9 | 57962.8 | 56371.05 | 58517.7 | 56652.17 | 56562.79 | 55898.61 | 55132.5 | 55775 |
| 422.3151 | 59495.5 | 58455.7 | 59532 | 61898 | 58581.25 | 58090 | 58359.3 | 57055.8 | 60376.75 | 57291.11 | 57188.16 | 56471.17 | 55347.33 | 56774 |
| 422.4518 | 62225 | 59995 | 61567.4 | 66580.89 | 59968.45 | 59179 | 59938.65 | 58614.1 | 63622.35 | 58455.22 | 58663.42 | 57367.94 | 55588.5 | 58720 |
| 422.5884 | 65656.33 | 63435.95 | 66425.25 | 71107.21 | 62986.65 | 62152.45 | 63169.65 | 60902.78 | 67687.95 | 60336.94 | 60563.95 | 58598.28 | 56290.5 | 59897 |
| 422.7251 | 66810.17 | 67355.1 | 72399.75 | 71814.32 | 67844.95 | 66423.55 | 67839.25 | 62603.55 | 69267.9 | 62247.83 | 61129.68 | 60224.06 | 57148.33 | 58402 |
| 422.8618 | 65693.67 | 71901.45 | 74938.95 | 68635.74 | 71835.9 | 70239.85 | 72252.4 | 62952.9 | 67859.45 | 62967 | 60284.32 | 60781.72 | 57121.83 | 59384 |
| 422.9984 | 62113 | 72986.7 | 74054.65 | 64480.32 | 72313.05 | 69526.55 | 73620.5 | 61445.1 | 65529.75 | 62482.28 | 59070.63 | 59833.06 | 56379 | 58351 |
| 423.1351 | 59815.17 | 70250 | 69370.1 | 61475.68 | 69308.6 | 65767.15 | 70044.65 | 59465.03 | 62116.5 | 60621.72 | 57718.74 | 58422.22 | 56202.33 | 57038 |
| 423.2717 | 58404.33 | 65843.4 | 64877.2 | 59726.26 | 65350.2 | 62307.15 | 65824.8 | 57939.2 | 59907.4 | 58872.94 | 57106.58 | 57441.17 | 56027.17 | 55476 |
| 423.4084 | 57093.67 | 63003.4 | 61704 | 58633.79 | 61602.95 | 59834.65 | 62109.3 | 57235.68 | 58697.4 | 57717.5 | 56409.21 | 56730 | 55831.5 | 55659 |
| 423.545 | 56959.5 | 60322.1 | 59862.95 | 58251.37 | 59878.3 | 58420.8 | 60187.35 | 56667.6 | 58039.85 | 56957.11 | 56184.05 | 56440.11 | 55516.17 | 55124 |
| 423.6816 | 56563.67 | 59234.25 | 59059.5 | 57980.21 | 58636.7 | 57772.7 | 58765.05 | 56317.48 | 57515.4 | 56622.33 | 56038.53 | 56382.22 | 55343.17 | 55100 |
| 423.8182 | 56301.33 | 58648.55 | 58879.8 | 57531.53 | 58137.4 | 57623.05 | 58393.15 | 55986.23 | 57319.8 | 56479.22 | 56070.26 | 56111.39 | 55180 | 56005 |
| 423.9548 | 56374.83 | 58004.95 | 58169.8 | 57309.11 | 57787.75 | 57272.05 | 57931.35 | 56029.23 | 57251.65 | 56254.67 | 56024.11 | 55900.56 | 55456.83 | 55342 |
| 424.0914 | 56298 | 57986.6 | 58312.5 | 57301.79 | 57709.3 | 56865.6 | 57569.95 | 56028.13 | 56979.45 | 56179.56 | 56062.47 | 55991.94 | 55687 | 55317 |
| 424.2281 | 56661.17 | 57874.5 | 58312.9 | 57391.68 | 57504.1 | 56750.05 | 57446.7 | 56012.3 | 56933.4 | 56045.5 | 56033.68 | 55964.44 | 55363.17 | 55556 |
| 424.3647 | 56943.67 | 57565.45 | 57911.05 | 57191.47 | 57654.3 | 56769.05 | 57412.9 | 56013.88 | 56835.7 | 56017.28 | 56194.63 | 55831.06 | 55305.5 | 55751 |
| 424.5012 | 56613.33 | 57549.1 | 57761.8 | 57075.53 | 57347.45 | 57005.25 | 57144.85 | 55999.35 | 56920.55 | 56318.67 | 56040.42 | 55656 | 55209.33 | 55342 |
| 424.6378 | 56620 | 57779.8 | 57998.8 | 57121.05 | 57327.85 | 56771.1 | 57228.9 | 55811.8 | 57054.8 | 56275.33 | 55842.32 | 55876.89 | 55368.33 | 55043 |
| 424.7744 | 56415.5 | 57438.3 | 57492.65 | 57176 | 57300 | 56907.35 | 57331.85 | 55720.63 | 56742.8 | 56251.44 | 55769.58 | 55897.44 | 55396.83 | 55333 |
| 424.911 | 56129.33 | 57611.25 | 57730.65 | 57017.37 | 57532.5 | 57033.65 | 57296.1 | 55900.55 | 56715.9 | 56109.33 | 55864.84 | 55875.94 | 55469.33 | 56216 |
| 425.0475 | 56724.33 | 57686.25 | 57703.2 | 56930.53 | 57359.55 | 56854.6 | 57518.25 | 55845.05 | 56687.25 | 56103.67 | 55900.42 | 55557.22 | 55461.5 | 55788 |
| 425.1841 | 56556.17 | 57651.4 | 57639.8 | 56984.95 | 57319.75 | 56746.75 | 57206.95 | 55977.05 | 56663.45 | 55981.28 | 55941.58 | 55587.56 | 55386.67 | 55689 |
| 425.3207 | 56122.67 | 57392.05 | 57995.5 | 57156.84 | 57252.05 | 56805 | 57201.4 | 55992.25 | 56607.5 | 56021.33 | 55643.84 | 55671.22 | 55407.17 | 54870 |
| 425.4572 | 56174.83 | 57061.6 | 57939.85 | 56928.89 | 57113.5 | 56723.45 | 57181.8 | 55949.83 | 56570.4 | 56029.28 | 55670.37 | 55777.78 | 55239 | 55149 |
| 425.5937 | 56462.67 | 57127.25 | 57574.75 | 56926.84 | 57013.05 | 56796.15 | 57014.3 | 55947.25 | 56760.95 | 56042.06 | 55850.26 | 55874.44 | 55290.17 | 55312 |
| 425.7303 | 56461.33 | 57439.3 | 57504 | 57048.74 | 57286.15 | 56610.6 | 56948 | 55848.63 | 56446.55 | 55923.39 | 55956.95 | 55787 | 55516.67 | 55290 |
| 425.8668 | 56641.17 | 57247.75 | 57407.85 | 57115.42 | 57155.4 | 56574.5 | 56962.25 | 55861.93 | 56598.9 | 56211.67 | 56113.32 | 55755.39 | 55425.67 | 56112 |
| 426.0033 | 56773.33 | 57153.6 | 57219 | 56884.53 | 57065.3 | 56637.15 | 56964.5 | 55901.35 | 56670.05 | 56062.33 | 56107.68 | 56086.89 | 55332.33 | 55689 |
| 426.1398 | 56533 | 57183.7 | 57434.6 | 56828.89 | 56986.8 | 56618.2 | 56943.75 | 55872.6 | 56777.75 | 55885.89 | 55963.53 | 55861.5 | 55503.17 | 55409 |
| 426.2763 | 56891.17 | 57257.3 | 57399.75 | 56973.74 | 56904.15 | 56829.05 | 57201.25 | 55977.48 | 56788.1 | 56317.39 | 55892.11 | 56013.33 | 55277.17 | 55385 |
| 426.4128 | 56777.83 | 57466.1 | 57036.5 | 57063.84 | 57037.35 | 56700.55 | 56888.05 | 56005.6 | 56749.35 | 56186.33 | 55984.74 | 55878.61 | 55374.17 | 55554 |
| 426.5493 | 56604.83 | 57519.3 | 57416.5 | 56925.42 | 57008.55 | 56630.3 | 57036.5 | 55887.93 | 56708.2 | 55936.67 | 55906.37 | 55814.61 | 55274 | 55423 |
| 426.6858 | 56414.83 | 57303.45 | 57542.6 | 56863.16 | 56832.25 | 56918.4 | 57275.45 | 55947.2 | 56693.35 | 56078.06 | 55818.26 | 55830.61 | 55323.33 | 55915 |
| 426.8223 | 56249.33 | 57111.8 | 57503.55 | 57130.58 | 56975.1 | 56835.7 | 57439.5 | 55909.13 | 56816.55 | 56038.28 | 55847.26 | 55998.33 | 55265.83 | 55606 |
| 426.9588 | 56433 | 57082.5 | 57266 | 57239.16 | 56973.1 | 56704.5 | 57403.95 | 55882.08 | 56738.3 | 56068 | 55805.16 | 56011.06 | 55804.83 | 55825 |
| 427.0952 | 56321 | 57172.85 | 57393.2 | 57136.37 | 57025.45 | 56716.35 | 57424.7 | 55858.33 | 56854.1 | 56181.28 | 55851.47 | 55879.94 | 55733.5 | 55362 |
| 427.2317 | 56219.33 | 56913.95 | 57795.6 | 57145.32 | 56961.1 | 56637.15 | 57355.15 | 55911.75 | 56849.85 | 56325.33 | 55894.53 | 55971.17 | 55716.83 | 56278 |
| 427.3681 | 56451.33 | 57054.4 | 57489.5 | 56912.63 | 57044.2 | 56741.3 | 57455.1 | 55901.6 | 56644.75 | 56037.72 | 55949.95 | 55956.5 | 55365.17 | 55429 |
| 427.5046 | 56560.17 | 57243.9 | 57294.15 | 56851.63 | 57214.15 | 56723.9 | 57180.3 | 55898.48 | 56573.65 | 56090.5 | 55796.58 | 55632.06 | 55427 | 55207 |
| 427.6411 | 56431 | 57025.3 | 57259.15 | 57089.26 | 57177.95 | 56505.35 | 57199.55 | 55981.23 | 56606.5 | 56169.39 | 55850.05 | 55840.5 | 55484.33 | 55689 |
| 427.7775 | 56290.83 | 56966.9 | 57096.8 | 57171.16 | 57088.65 | 56730.75 | 57202.25 | 55972.13 | 56766.25 | 56009.17 | 55744.42 | 55879.17 | 55452.17 | 55615 |
| 427.9139 | 56940.33 | 57072.65 | 57191.45 | 57121.16 | 57059.8 | 56515.5 | 57261.6 | 55863.35 | 56766.05 | 56108.94 | 55690.42 | 55611.56 | 55766.67 | 55230 |
| 428.0503 | 56804.5 | 57017.4 | 57129.7 | 57051.21 | 57015.85 | 56473.4 | 57056.6 | 55788.9 | 56692.3 | 55938.39 | 55692.32 | 55551.33 | 55621.5 | 55602 |
| 428.1867 | 56517.5 | 57078.5 | 57124.7 | 57096.42 | 56956.9 | 56423.4 | 57277.6 | 55913.75 | 56567.35 | 56122.17 | 55876.89 | 55751.94 | 55588.67 | 56530 |
| 428.3232 | 56657.83 | 57203.8 | 57089 | 57210.21 | 57117.95 | 56373.05 | 57219.1 | 55769.48 | 56403.25 | 55965.56 | 55869.68 | 55914.56 | 55553.83 | 56543 |
| 428.4595 | 56506.83 | 57011.95 | 57077.75 | 57436.42 | 56850.45 | 56313.5 | 57036.05 | 55865.13 | 56257.1 | 56194.17 | 55772.74 | 55894.11 | 55455.33 | 55535 |
| 428.596 | 56427.33 | 57148.95 | 57174.1 | 57261.84 | 56751.4 | 56782.95 | 57070.1 | 55839.85 | 56293.4 | 56235.67 | 55724.63 | 56081.17 | 55300.33 | 54889 |
| 428.7323 | 56491.67 | 56994.7 | 57129.2 | 57158.26 | 56772.6 | 56634.85 | 57020.6 | 55895.13 | 56563.9 | 56655.17 | 55799.37 | 56212.72 | 55694.83 | 55429 |
| 428.8687 | 56432.67 | 57050.55 | 57335.15 | 56935.79 | 56769.95 | 56565.6 | 57063.05 | 55870.63 | 56655.55 | 56150.89 | 55741.74 | 55862.44 | 55650.33 | 55591 |
| 429.0051 | 55960.67 | 56965.25 | 57305.9 | 57021.26 | 56856.4 | 56681 | 57374.95 | 55905.38 | 56472.6 | 56262.11 | 55791.26 | 56069.44 | 55444.67 | 55024 |
| 429.1415 | 56212 | 56909.7 | 57208 | 56892.63 | 56657.15 | 56735 | 57369.75 | 55851.43 | 56526 | 56335.11 | 55948.26 | 55987.06 | 55697.5 | 55469 |
| 429.2778 | 56033.33 | 56879 | 57387.25 | 56946.37 | 56729.8 | 56654.1 | 56838.85 | 55975.38 | 56602.6 | 56164.78 | 55767.68 | 55829.94 | 55696.33 | 54964 |
| 429.4142 | 56169.67 | 57193.75 | 57162.7 | 56969.21 | 56948.65 | 56685.1 | 57018.35 | 55864.53 | 56629.05 | 56159.06 | 55896.32 | 55830.06 | 55575.5 | 55596 |
| 429.5505 | 56201.67 | 57201.1 | 57302.15 | 56982.95 | 56703.95 | 56891.45 | 57019.35 | 55950.6 | 56568.65 | 55965.67 | 56082.11 | 55817.39 | 55542.83 | 54619 |
| 429.6869 | 56050.5 | 57096.35 | 56999.75 | 56989.11 | 56743.7 | 57021.3 | 57250.6 | 55973.4 | 56526.25 | 56064.33 | 56032.89 | 55910 | 55563.67 | 54721 |
| 429.8232 | 55937.5 | 56996.1 | 57147.45 | 56906.11 | 57406 | 56780.75 | 57250.7 | 55903.63 | 56571.45 | 56291.17 | 56027.84 | 56165.83 | 55537.33 | 55152 |
| 429.9595 | 56395.5 | 57179.05 | 57216.75 | 57329.42 | 57242.55 | 56688.65 | 57241.4 | 56012.85 | 56714.35 | 56210.39 | 56084.16 | 56007.56 | 55470.83 | 55438 |
| 430.0958 | 56543.33 | 56999.05 | 57310.3 | 57197.79 | 56925 | 56855.6 | 57065.7 | 56045.2 | 56810.4 | 56203.17 | 55994.47 | 55694.22 | 55312.17 | 55429 |
| 430.2321 | 56042.17 | 57142.3 | 57374.55 | 57144 | 57102.65 | 56781.85 | 57002.05 | 56091.05 | 56756.75 | 56044.06 | 56032.26 | 55873.44 | 55588.83 | 55525 |
| 430.3684 | 56422 | 57449.3 | 57456.1 | 57399.42 | 57351.05 | 56968.4 | 57334.7 | 55896.05 | 56552.05 | 56207.89 | 55899.74 | 55880.11 | 55556.83 | 55376 |
| 430.5047 | 56953.67 | 57439.45 | 57516.1 | 57251.05 | 57268.6 | 57051.05 | 57464.05 | 55990.63 | 56681.8 | 56242.33 | 55961.53 | 55702.83 | 55278.83 | 54934 |
| 430.641 | 56661.83 | 57486.6 | 57590.3 | 57295.21 | 57123.6 | 56977.2 | 57175.65 | 56041.85 | 56562.75 | 56269.11 | 56015.74 | 55913 | 54960.83 | 54933 |
| 430.7773 | 56733.67 | 57530.85 | 57557.7 | 57355.21 | 57378.25 | 56830.1 | 57419.55 | 56007.4 | 56600.65 | 56081.22 | 56203.26 | 56013.44 | 55172.67 | 55108 |
| 430.9136 | 57013 | 57340.2 | 57742.55 | 57777.74 | 57509.2 | 56978.25 | 57255.95 | 56065.03 | 56824.95 | 55966.28 | 56186.74 | 56007.06 | 55435.17 | 55369 |
| 431.0498 | 56635.67 | 57517.4 | 57591.1 | 57553.37 | 57611.8 | 57084.15 | 57635.3 | 56199.13 | 56841.7 | 56445.33 | 56006.53 | 56179.89 | 55358.17 | 55778 |
| 431.1861 | 56456.17 | 57488.7 | 57651.3 | 57416.26 | 57414.35 | 57021.85 | 57653.95 | 56107.6 | 56883.6 | 56350.5 | 56186.95 | 56271.78 | 55508.67 | 56672 |
| 431.3224 | 56178.83 | 57447.4 | 57589.1 | 57427.47 | 57488.55 | 57096.3 | 57764.25 | 56061.4 | 56967.55 | 56327.56 | 56057.37 | 56207.78 | 55023.83 | 56655 |
| 431.4586 | 56653.67 | 57574.6 | 57779 | 57143.84 | 57736.1 | 57027.95 | 57407 | 56058.18 | 56923.5 | 56509.39 | 55974.21 | 55786.11 | 55091.5 | 56511 |
| 431.5948 | 56834.5 | 57695.1 | 57887.4 | 57084.79 | 57483 | 57044.9 | 57454.6 | 56080.85 | 56918.95 | 56373 | 56034.74 | 55753.94 | 55529.67 | 55553 |
| 431.7311 | 56838 | 57663.5 | 57782.05 | 56936.05 | 57287.7 | 57052.3 | 57041.3 | 56058.43 | 56982.5 | 56350.17 | 55789.95 | 55965.56 | 55504.5 | 56296 |
| 431.8673 | 56737.33 | 57456.8 | 57772.9 | 57317.79 | 57335.35 | 57008.3 | 57158.55 | 55953.73 | 56805.65 | 56412.44 | 55962.84 | 55796.33 | 55354.67 | 55864 |
| 432.0035 | 56757 | 57555.4 | 57534.25 | 57052.32 | 57054.95 | 57062.85 | 57425.1 | 56015.48 | 56770.9 | 56249.5 | 55849.95 | 55902.22 | 55264.17 | 55817 |
| 432.1397 | 56751 | 57329.25 | 57565.5 | 57121.11 | 57456.5 | 57033.75 | 57384.15 | 56057.13 | 56777.75 | 56318.67 | 55863.37 | 56152.33 | 55203.17 | 55432 |
| 432.2759 | 56801.17 | 57613.6 | 57452.65 | 57419.32 | 57605.85 | 56824.3 | 57543.4 | 56052.25 | 56783.95 | 56361.39 | 56045 | 56057.56 | 55347.83 | 55915 |
| 432.4121 | 56955.17 | 57622.9 | 57338.5 | 57237.42 | 57338.45 | 56748.35 | 57494.25 | 56039.18 | 56685.45 | 56231.44 | 56373.11 | 56025 | 55191.67 | 57051 |
| 432.5483 | 57294.5 | 57716.4 | 57515.25 | 57276.42 | 57170.35 | 56508.45 | 57632.75 | 56024.93 | 56615.1 | 56269.67 | 56024.05 | 56116.56 | 55637.33 | 56164 |
| 432.6845 | 56729.67 | 57664.85 | 57503.65 | 57301.53 | 57181.05 | 56799.75 | 57549.35 | 56202.48 | 57092.55 | 56280.5 | 55986.21 | 55906.33 | 55653.33 | 55311 |
| 432.8206 | 56821.17 | 57566.9 | 57740.85 | 57162.21 | 57368.95 | 56916.3 | 57242.5 | 56199.43 | 56855 | 56281.22 | 55770.89 | 56059.22 | 55634.17 | 56137 |
| 432.9568 | 56682.5 | 57562.35 | 57482.45 | 56971.63 | 57561.45 | 56885.3 | 57336.3 | 56188.38 | 56894.4 | 56199.11 | 55815.05 | 56132.28 | 55870 | 55271 |
| 433.0929 | 56545.83 | 57381.25 | 57630.2 | 57106.32 | 57350 | 56804.75 | 57563.5 | 56251.23 | 56885.9 | 56267.33 | 55854.68 | 56004.22 | 55607.5 | 54707 |
| 433.2291 | 56471.83 | 57200.6 | 57332.5 | 57190.89 | 57341.65 | 56768.1 | 57428.85 | 56104.6 | 56915.55 | 56167.89 | 55997.26 | 56057.5 | 55461 | 54911 |
| 433.3652 | 56389.33 | 57346 | 57244 | 57111.53 | 57225.75 | 56588.1 | 56971.35 | 56111.7 | 56636.8 | 56262.83 | 55979.47 | 55865.78 | 55507 | 56161 |
| 433.5013 | 56512.5 | 57268.45 | 57431 | 56953.32 | 57204.3 | 56883.35 | 57225.15 | 56092.55 | 56808.6 | 56244.94 | 55976.89 | 55805.22 | 55542 | 55526 |
| 433.6375 | 56728 | 57553.4 | 57454.95 | 57181.37 | 57021.5 | 57008.2 | 57257.65 | 56059.35 | 56617.8 | 56350.72 | 55801.63 | 55933.61 | 55334.5 | 55544 |
| 433.7736 | 56589.5 | 57425.95 | 57250.5 | 57270.89 | 57103 | 56978.8 | 57508.55 | 56085.93 | 56715.25 | 56285.67 | 55782.16 | 56286.17 | 55627 | 55287 |
| 433.9097 | 56876.33 | 57482.4 | 57391.6 | 57314.95 | 57136.35 | 56828.35 | 57380.15 | 56139.9 | 56851.05 | 56312 | 55883.84 | 56180.28 | 55222 | 56080 |
| 434.0458 | 56744.17 | 57589.2 | 57488.3 | 57203.32 | 57241.75 | 56709.95 | 57137.6 | 56077.25 | 56908.45 | 56062.28 | 55780.95 | 56090.56 | 55092.83 | 56249 |
| 434.1819 | 57071.5 | 57633.85 | 57538.25 | 57262.42 | 57208.8 | 56812.55 | 57460.95 | 55908.3 | 56739.15 | 56153.28 | 55770 | 55858.5 | 55454 | 55183 |
| 434.318 | 56800.83 | 57485.8 | 57623.5 | 57213.74 | 57344.6 | 56748.95 | 57117.65 | 56054.9 | 56758.25 | 56114.72 | 55678.95 | 55922.5 | 55554.17 | 55301 |
| 434.454 | 56320.67 | 57530.8 | 57478.2 | 56995.42 | 57121.95 | 56505.65 | 57077.45 | 56160.95 | 56737.45 | 56129.83 | 55952.89 | 55881.11 | 55675.67 | 54899 |
| 434.5901 | 56243.17 | 57473.95 | 57147.75 | 56969.95 | 57276.8 | 56454 | 57076.1 | 56335.03 | 56702.95 | 56275.11 | 56070.42 | 56314.06 | 55567.83 | 54889 |
| 434.7261 | 56321.67 | 57250.3 | 57227.55 | 56993.37 | 57254.2 | 56482.3 | 57198.05 | 56291.68 | 56949.35 | 56201.78 | 55980 | 55910.39 | 55635.67 | 55736 |
| 434.8622 | 56590.83 | 57364.1 | 57425.6 | 57001.05 | 57240.1 | 56589 | 57483 | 56099.35 | 56779.45 | 56276.44 | 55912.16 | 55910.17 | 55656 | 56713 |
| 434.9982 | 56679.83 | 57438.3 | 57297.35 | 57211.42 | 57043.2 | 56636.45 | 57461.1 | 56071.98 | 56464.5 | 56432.61 | 56199.53 | 55912.83 | 55684.17 | 55315 |
| 435.1343 | 56474.33 | 57293.3 | 57549.95 | 57237.16 | 57065.75 | 56705.1 | 57393.55 | 56080.6 | 56449.55 | 56299.44 | 55977.74 | 56022.94 | 55709.83 | 54751 |
| 435.2703 | 57018 | 57073.1 | 57333.15 | 57353.95 | 56978.3 | 56686.3 | 57346.95 | 56077.28 | 56614.45 | 56277.17 | 56047.26 | 55993.67 | 55636.67 | 54952 |
| 435.4063 | 56768 | 57332.75 | 57531.2 | 57132.89 | 57035.75 | 56665.65 | 57386.35 | 56012.43 | 56768.9 | 56287.17 | 55764.53 | 56018.78 | 55630.67 | 54797 |
| 435.5423 | 56914 | 57366.2 | 57789.1 | 56882.42 | 57151.45 | 56822.8 | 57363 | 56061.35 | 57009.85 | 55958.61 | 55905.21 | 56098.56 | 55542.17 | 55386 |
| 435.6783 | 57058.83 | 57269.8 | 57567.6 | 56980.42 | 57198.75 | 56680.35 | 57299.25 | 56078.13 | 56916.45 | 56069.22 | 55826.21 | 55849.72 | 55496.67 | 55673 |
| 435.8143 | 56827.83 | 57499.8 | 57464 | 57138.37 | 57304 | 56686.9 | 57341.8 | 56152.2 | 56643.1 | 55942.17 | 55969.42 | 55973.67 | 55681.33 | 55749 |
| 435.9503 | 56754.67 | 57480.35 | 57317.7 | 57073.21 | 57364.65 | 56845.7 | 57432.15 | 56012.28 | 56777.55 | 56172 | 55828.58 | 56158.67 | 55325.83 | 54798 |
| 436.0863 | 56698.67 | 57555.25 | 57184.8 | 57198.63 | 57099.95 | 56983.55 | 57403.65 | 55974.4 | 56893.2 | 56134.67 | 56022.74 | 55906.06 | 55229.5 | 54658 |
| 436.2222 | 56536 | 57450.35 | 57269.3 | 57461.89 | 56908.9 | 56801.1 | 57415.55 | 56054.68 | 56735.7 | 56113.61 | 56079.16 | 55888.78 | 55170.5 | 55069 |
| 436.3582 | 56811.67 | 57428.75 | 57289.65 | 57096.21 | 56964.15 | 56739.65 | 57127.15 | 56112.35 | 56681.55 | 56130.28 | 55867.95 | 55901.22 | 55234.5 | 55572 |
| 436.4941 | 56648.17 | 57295.4 | 57105.2 | 57231.16 | 57134.5 | 56882.8 | 57018.25 | 56139.78 | 56765.55 | 56240.22 | 55775.32 | 55876.72 | 55296 | 55334 |
| 436.6301 | 57105.67 | 57150.25 | 57387 | 57129.63 | 56886.05 | 56855.85 | 57161.9 | 55963.4 | 56673.1 | 56406.33 | 55866.79 | 55939.61 | 55260.17 | 56514 |
| 436.766 | 56800.5 | 57187.45 | 57338.8 | 57067.32 | 56934.2 | 56682.45 | 57294.2 | 55989.13 | 56666.05 | 56270.33 | 55881.89 | 56139.89 | 55202.33 | 56483 |
| 436.902 | 56671.33 | 57074.2 | 57214.5 | 56944.79 | 56976.35 | 56535.75 | 57416.45 | 55932.45 | 56589.25 | 56247.78 | 56052.58 | 56077.94 | 55245 | 56365 |
| 437.0379 | 56586.67 | 57313.8 | 57186.1 | 56914.16 | 56885 | 56750.15 | 57089.2 | 56024.03 | 56786.55 | 56089 | 55963.32 | 55929.44 | 55327.83 | 56382 |
| 437.1738 | 56582.67 | 57253.55 | 57408.45 | 57101.84 | 56942.65 | 56867.65 | 57023.1 | 56115.93 | 56762.85 | 55875.89 | 55976.53 | 55886.5 | 55573.5 | 56640 |
| 437.3097 | 57000 | 57312 | 57483.5 | 57038.89 | 56969.2 | 56823 | 57275.15 | 56041.4 | 56786.95 | 55995.06 | 55900 | 56047.06 | 55619.67 | 55749 |
| 437.4456 | 56946 | 57299.2 | 57442.15 | 57015.53 | 57009.3 | 56747.95 | 57437.5 | 55932.23 | 56934.5 | 56142.39 | 55969 | 56003.39 | 55497.67 | 56726 |
| 437.5815 | 56598 | 57254.5 | 57529 | 57000.11 | 56882.05 | 56844.6 | 57336.15 | 55890.83 | 56495.9 | 56078.83 | 56029.89 | 56176.28 | 55905.67 | 56046 |
| 437.7173 | 56392 | 57220.75 | 57437.85 | 56848.42 | 56998.75 | 56725.45 | 57280.4 | 55945.55 | 56623.85 | 56198.33 | 56106.68 | 56245.39 | 55380.33 | 55708 |
| 437.8532 | 56387.17 | 57314.65 | 57215.8 | 56836.05 | 57249.15 | 56920.6 | 57201.4 | 55882.73 | 56857.4 | 56188.78 | 56190.05 | 56123.89 | 55343.17 | 55356 |
| 437.989 | 56295.67 | 57286.35 | 57391.2 | 56856.26 | 57074.75 | 56873.15 | 57057.9 | 56095.38 | 56742.85 | 56156 | 55952 | 56033.67 | 55255.67 | 56232 |
| 438.1249 | 56344.17 | 57495.1 | 57271.25 | 56746.47 | 56715.5 | 56664.5 | 57263.6 | 55922.7 | 56871.25 | 56440.67 | 56040.53 | 56004.33 | 55352.83 | 55357 |
| 438.2607 | 56699.17 | 57308.7 | 57500.1 | 57092.95 | 56691.35 | 56563.95 | 57190.8 | 56042.7 | 57193.1 | 56459.06 | 55907.16 | 55886.67 | 55602.67 | 55660 |
| 438.3966 | 56671.67 | 57248.6 | 57444.45 | 56974.53 | 56956.4 | 56968.55 | 57303.85 | 56057.03 | 56770.65 | 56333.28 | 55736.47 | 56004.94 | 56014 | 56226 |
| 438.5324 | 56481.33 | 57302.95 | 57384.75 | 57348.47 | 57109.7 | 57142.9 | 57274.1 | 56128.1 | 56988.1 | 56360.56 | 55776.89 | 56003.72 | 55731 | 55262 |
| 438.6682 | 56584 | 57397.2 | 57520.35 | 57264 | 57083.85 | 56947 | 57349.05 | 56152.05 | 56885.7 | 56276.06 | 55883.16 | 56204.78 | 55990.17 | 55738 |
| 438.804 | 56398.17 | 57436.8 | 57423.6 | 57312.42 | 57239 | 57068.95 | 57238.25 | 56088.65 | 56657.65 | 56164.28 | 55982.89 | 56015.94 | 55688.17 | 55534 |
| 438.9398 | 56407.17 | 57391 | 57142.1 | 56909.11 | 57279.5 | 56629.6 | 57074.4 | 55977.93 | 56597.05 | 56179.78 | 56079.53 | 55913.44 | 55501 | 55110 |
| 439.0756 | 56320.17 | 57034.65 | 57323.2 | 57010.47 | 57115.85 | 56611.4 | 57082.55 | 55953.2 | 56518.25 | 56102.78 | 56070.84 | 55765.83 | 55575.5 | 55637 |
| 439.2113 | 56305.33 | 57209.6 | 57331.65 | 56953.32 | 57020.95 | 56652 | 57126.1 | 55892.5 | 56738 | 55916.28 | 56006.05 | 55998.39 | 55738.17 | 56215 |
| 439.3471 | 56574.5 | 57185.1 | 57549.55 | 56988.58 | 57089.45 | 56650.2 | 56871.35 | 56034.9 | 56915.5 | 56165.06 | 56060 | 55834.89 | 55402.5 | 56693 |
| 439.4829 | 56371.67 | 57139.8 | 57209.85 | 56986.11 | 56954.65 | 56426.3 | 56801.2 | 56032.98 | 56670.95 | 56213.83 | 55872.47 | 55881.33 | 55340.5 | 55567 |
| 439.6186 | 56297.17 | 56983.75 | 57205 | 56879.47 | 56994.85 | 56487.65 | 56985.2 | 55955.3 | 56801.75 | 56308.78 | 55936.63 | 55952.5 | 55244 | 55792 |
| 439.7544 | 56128.67 | 56989.05 | 57334.05 | 56946.21 | 56801.05 | 56353.8 | 56980.7 | 55977.03 | 56723.2 | 56271.44 | 55855.47 | 56006.11 | 55255.67 | 56874 |
| 439.8901 | 56405.83 | 57172.95 | 57168.25 | 56838.11 | 56916.5 | 56483.75 | 57082.75 | 56003.53 | 56906.35 | 56004.5 | 55789 | 55894.94 | 55123 | 56399 |
| 440.0258 | 56595.17 | 57149.35 | 57134.55 | 56999.11 | 57019.6 | 56260.05 | 57033.15 | 55888.08 | 56676.35 | 56009.33 | 55788.21 | 56052.28 | 55308.33 | 56112 |
| 440.1615 | 56454.67 | 57277.05 | 57423.4 | 57070.63 | 56997.05 | 56215.5 | 57156.05 | 55896.88 | 56499.45 | 56039.67 | 55823.47 | 55935.22 | 55379 | 55429 |
| 440.2972 | 56281.5 | 57139.5 | 57267.25 | 57102.95 | 56808.5 | 56393.5 | 57051.4 | 55907.88 | 56522.85 | 56034.5 | 55923.32 | 56026.83 | 55223.5 | 55319 |
| 440.4329 | 55903 | 57196.95 | 57510.65 | 57144.21 | 56856.6 | 56560.65 | 57105.25 | 56047.6 | 56741.05 | 56151.83 | 56011.32 | 56062.72 | 55329.17 | 56192 |
| 440.5686 | 56264 | 57069.9 | 57363.1 | 57190.42 | 56890.95 | 56667.55 | 57042.85 | 55998.95 | 56504.5 | 56402.17 | 55985.79 | 55873.17 | 55379.17 | 56056 |
| 440.7043 | 56634.17 | 57009.8 | 57413.2 | 56950.68 | 57210.85 | 56384.2 | 57141.7 | 55967.35 | 56654.4 | 56282.11 | 55787.37 | 55812.83 | 55351.83 | 56249 |
| 440.8399 | 56798.83 | 57182.25 | 57142.9 | 56957.26 | 57108.55 | 56624.05 | 56974.4 | 55963.1 | 56773.55 | 56118.78 | 55807.84 | 55738.17 | 55507.5 | 56690 |
| 440.9756 | 56587.33 | 57174.3 | 57206.5 | 57045.21 | 57013.85 | 56630 | 57121.2 | 55967.6 | 56543.5 | 55960 | 55832.26 | 55714.39 | 55201.83 | 58049 |
| 441.1112 | 56638.17 | 57100.5 | 57273.3 | 56851.16 | 56926.15 | 56754.05 | 56745.75 | 55995.03 | 56729.75 | 56158.28 | 55891.47 | 55847.11 | 54930.5 | 57725 |
| 441.2469 | 56061.5 | 57068.55 | 57195.6 | 56804.32 | 56927 | 56816.2 | 56720.9 | 55984.85 | 56472.9 | 56272.39 | 56074.05 | 55852.89 | 55292.67 | 57677 |
| 441.3825 | 56043.33 | 57491.85 | 57176.95 | 56928.05 | 56968.65 | 56729.65 | 56850.8 | 55908.6 | 56488.75 | 56366.83 | 56094.11 | 55882.56 | 55357.33 | 57110 |
| 441.5181 | 55984.83 | 57161.65 | 57088.35 | 56833.79 | 56932.65 | 56556.6 | 56876 | 55934.2 | 56706.35 | 56308.67 | 55941.26 | 55897 | 55309.17 | 56682 |
| 441.6538 | 56226.67 | 57082.3 | 57021.9 | 56748.53 | 56987.1 | 56576.5 | 57101.3 | 55996.93 | 56683.3 | 56114.56 | 55856.79 | 55955.33 | 55292.17 | 55269 |
| 441.7893 | 56013.83 | 57122.1 | 56978 | 56777.05 | 56905.75 | 56649.15 | 57137.6 | 55929.75 | 56703.7 | 56178.78 | 55784.79 | 55938.11 | 55203.83 | 55067 |
| 441.9249 | 56159.83 | 57266.5 | 57409 | 56697.68 | 56788.3 | 56646.05 | 57059.15 | 55936 | 56567.55 | 56226.33 | 55782.58 | 55818.67 | 55360.33 | 55912 |
| 442.0605 | 56092.83 | 57362.75 | 57142.65 | 56611.11 | 56879.7 | 56506.15 | 57362.55 | 55926.33 | 56615.7 | 56124.94 | 55698.32 | 55895.11 | 55199.83 | 55596 |
| 442.1961 | 56521.5 | 57297.8 | 57077.25 | 56484.74 | 56928.55 | 56407.75 | 57080.1 | 55936.05 | 56562.95 | 56350.56 | 55716.53 | 55695.39 | 55293.83 | 55579 |
| 442.3317 | 56632.67 | 57249.5 | 57034.5 | 56869.32 | 56937.95 | 56561.3 | 56931.25 | 55954.73 | 56542.55 | 56204.44 | 55975.79 | 55798.33 | 55252.67 | 56196 |
| 442.4672 | 56215.67 | 56971.9 | 57093.3 | 56799.53 | 57072.95 | 56609.85 | 56919.65 | 55924.05 | 56340.6 | 55966.22 | 55986.16 | 55713.83 | 55305.67 | 56366 |
| 442.6028 | 56510 | 57188.6 | 57159.75 | 56876.32 | 57138.3 | 56555.9 | 56808.9 | 55894.8 | 56481.45 | 55961.22 | 55871.47 | 55843.94 | 55173 | 55098 |
| 442.7383 | 56193 | 57166.95 | 57372.3 | 56913.84 | 56810 | 56539.45 | 56811.1 | 55977.75 | 56493.35 | 56013.5 | 55889.16 | 55763.11 | 55336.33 | 55546 |
| 442.8738 | 56472.5 | 57187.2 | 57280.95 | 56863.26 | 56886.55 | 56774.7 | 56862.5 | 55851.7 | 56406.55 | 55870.83 | 55709.42 | 55734.5 | 54968.33 | 55930 |
| 443.0093 | 56477.33 | 57041.9 | 57161.6 | 56984.53 | 56966.5 | 56550.4 | 56989.55 | 55881.28 | 56650.1 | 56173 | 55880.05 | 55773.06 | 55144.33 | 55624 |
| 443.1448 | 56288.83 | 57005.25 | 57278 | 56883.42 | 57038.25 | 56408.65 | 56957.4 | 56017.63 | 56713.95 | 56370 | 56035.11 | 55877.33 | 55152.5 | 55538 |
| 443.2803 | 56054.33 | 57033.3 | 56995.3 | 56664.74 | 57015.9 | 56381.95 | 57013.8 | 56064.35 | 56583.3 | 56182.61 | 56088.42 | 55825.67 | 55431.5 | 54847 |
| 443.4158 | 56195.33 | 57203.15 | 57096.55 | 56706.79 | 56967.65 | 56554.45 | 56964.85 | 55982.7 | 56612.85 | 56270.61 | 55967.84 | 55901.5 | 55366 | 55395 |
| 443.5513 | 56152 | 57004.95 | 57259.3 | 56865.32 | 56912.25 | 56688.85 | 56756.45 | 55951.48 | 56517.75 | 56110.5 | 55927.47 | 56035.06 | 55410.67 | 55915 |
| 443.6867 | 56303.33 | 57152.2 | 57270.85 | 56795.37 | 57016.8 | 56603.7 | 57077.9 | 55967.95 | 56616.1 | 56065.5 | 56133.95 | 55961.22 | 55193 | 55184 |
| 443.8222 | 56581.5 | 57027.1 | 57055.45 | 56536.37 | 57041.55 | 56498.15 | 57164.2 | 55890.98 | 56387.5 | 56176.22 | 55985.58 | 55796.11 | 55484.17 | 54833 |
| 443.9576 | 56174.83 | 56968.35 | 57071.85 | 56651.16 | 57101.8 | 56518.2 | 57143.55 | 55919.7 | 56265.55 | 56077.28 | 55838.47 | 55863.39 | 55612.83 | 54786 |
| 444.0931 | 56377 | 56738.2 | 57079.9 | 56665.32 | 57192.8 | 56519.65 | 56961.95 | 55931.85 | 56508.25 | 56064.56 | 55905.11 | 56070.22 | 55494.33 | 54882 |
| 444.2285 | 56001.5 | 56773.55 | 57044.1 | 56718.26 | 56830.55 | 56575.7 | 57133.45 | 55927.15 | 56254.6 | 55980.39 | 55863.11 | 56041 | 55369.83 | 54859 |
| 444.3639 | 55961 | 57050.75 | 57314 | 56540.68 | 56847.75 | 56475.95 | 57307.65 | 55862.63 | 56224.15 | 55909.5 | 55757.11 | 55791.78 | 55261 | 55031 |
| 444.4993 | 56053.33 | 57083.8 | 57356.9 | 56838.95 | 56757.2 | 56523.75 | 57099.45 | 55825.95 | 56352.6 | 56000.5 | 55666.42 | 55599.44 | 55232.5 | 55160 |
| 444.6347 | 56269.67 | 57076.75 | 57088.7 | 56806.58 | 56802.25 | 56567.5 | 56735.35 | 55755.2 | 56453.9 | 56396.17 | 55802.47 | 55974.67 | 55276.5 | 55139 |
| 444.7701 | 55986.33 | 57105.05 | 57249.25 | 56814 | 56814.35 | 56529.2 | 56610.35 | 55974.88 | 56630.6 | 56283.67 | 55872.16 | 56054.28 | 55125.33 | 55058 |
| 444.9055 | 56357 | 56908.95 | 57166.9 | 56653.37 | 56814.4 | 56623.9 | 56775.15 | 55934.18 | 56466.2 | 56437.61 | 55932.32 | 55961.56 | 55161.5 | 56203 |
| 445.0409 | 56170 | 56575.6 | 57219.1 | 56666.95 | 56972.6 | 56773.65 | 56975.85 | 55998.45 | 56514.65 | 56233.72 | 55858.26 | 55737.39 | 55460 | 55620 |
| 445.1762 | 56393.5 | 56833.05 | 57086.3 | 56880.84 | 56823.6 | 56731.8 | 57028.15 | 55913.33 | 56541.75 | 56168.56 | 55626.95 | 55906.39 | 55412.5 | 55859 |
| 445.3116 | 56473.5 | 57001 | 57318.7 | 56896 | 56822.5 | 56630.45 | 56840.6 | 55908.98 | 56749.9 | 56210.78 | 55821.95 | 55796.5 | 55560.83 | 55906 |
| 445.4469 | 56702 | 56971.55 | 57269.3 | 56908 | 56992.6 | 56875.5 | 56885.5 | 55973 | 56586.3 | 56076.5 | 55742.37 | 55646.67 | 55341 | 55894 |
| 445.5822 | 56666.5 | 57016.3 | 57315.25 | 57108.05 | 56858.15 | 56915.95 | 56848.35 | 55888.28 | 56483.9 | 56129.83 | 55648.53 | 55719.72 | 55408 | 54838 |
| 445.7175 | 56195 | 56966.65 | 57140.55 | 56616.42 | 57093.55 | 56881.8 | 57025.6 | 55900.18 | 56743.3 | 56002.17 | 55802.79 | 55705.78 | 55221.83 | 55432 |
| 445.8528 | 56494 | 56872.7 | 57340.8 | 56726.21 | 57041 | 56783.6 | 56884.8 | 55906.6 | 56821.95 | 55948.94 | 55784.05 | 55828.61 | 55253.33 | 56172 |
| 445.9881 | 56148.67 | 56824.15 | 57247 | 56379.79 | 56957.5 | 56669.6 | 56918.2 | 55855.93 | 56686.4 | 56228.17 | 55999.42 | 55837.28 | 55567.67 | 55090 |
| 446.1234 | 56231.33 | 56823.65 | 57309.05 | 56806.16 | 56893.05 | 56583.4 | 56920.5 | 55843.45 | 56587 | 56019.44 | 55881.84 | 56035.17 | 55517.67 | 56085 |
| 446.2587 | 56596.5 | 56996.5 | 57352.1 | 56887.37 | 57167.15 | 56378.2 | 56947 | 55819.43 | 56520.05 | 56058.06 | 55849.37 | 55961.22 | 56033.83 | 55550 |
| 446.394 | 57213.67 | 57064.55 | 57298 | 56838.84 | 57094.35 | 56532.75 | 56753.3 | 55901.28 | 56516.25 | 56090.17 | 55841.84 | 55785.94 | 55555.83 | 55283 |
| 446.5292 | 56365.5 | 56997.2 | 57360.05 | 56981.21 | 56847.65 | 56652.85 | 56853.6 | 55879.13 | 56377.1 | 56037.22 | 55927.32 | 55821.39 | 55514.33 | 54910 |
| 446.6644 | 55867 | 56879.35 | 57166.9 | 56924.42 | 57000.5 | 56592.3 | 56951.05 | 55740.88 | 56340.85 | 55848.44 | 55920.68 | 56019.94 | 55644.5 | 55422 |
| 446.7997 | 56200.83 | 56997.5 | 57093.5 | 56829.42 | 56719.85 | 56292.1 | 56736.1 | 55761.33 | 56251.3 | 55975.28 | 55795.21 | 55763.11 | 55450.83 | 55391 |
| 446.9349 | 56224.5 | 56871.95 | 57208.4 | 56879 | 56924 | 56471.6 | 56774.85 | 55828.1 | 56287.15 | 56072.22 | 55693.16 | 55862.67 | 55429.67 | 54922 |
| 447.0701 | 56571.67 | 56985.45 | 57197.5 | 56799.84 | 56699.55 | 56677 | 56788.25 | 55806.08 | 56382.9 | 56095.56 | 55623.58 | 55944.78 | 55329.17 | 54892 |
| 447.2053 | 56437.17 | 56965.7 | 57046.35 | 56644.84 | 56756.35 | 56575.9 | 56643.15 | 55766.35 | 56268.7 | 56140.78 | 55713.84 | 55986.5 | 55389 | 55755 |
| 447.3405 | 56273.5 | 56740.1 | 56883.85 | 56753.16 | 56872.55 | 56433 | 56639.3 | 55821.8 | 56256.7 | 55949.17 | 55843.32 | 55719.11 | 55721 | 56209 |
| 447.4757 | 56690 | 56876.95 | 56929.4 | 56709.05 | 56956.65 | 56511.55 | 56545.7 | 55848.65 | 56449.05 | 55931.78 | 55983.79 | 56023.17 | 55796.5 | 55874 |
| 447.6108 | 56732.67 | 57021.9 | 56927.1 | 56666.63 | 56864 | 56512.8 | 56802.4 | 55840.58 | 56496.7 | 56115.67 | 55812.68 | 56032.94 | 55635.83 | 55259 |
| 447.746 | 56607.5 | 56819.95 | 56869.95 | 56933.11 | 56961.25 | 56357.55 | 56750.3 | 55772.88 | 56323.15 | 56115.83 | 55729.26 | 55987.72 | 55198.33 | 55338 |
| 447.8811 | 56043.17 | 56805.15 | 56943.9 | 56541.89 | 56892.8 | 56378.8 | 56762.65 | 55825.85 | 56280.45 | 56019.83 | 55848.79 | 55902.56 | 55279.33 | 55237 |
| 448.0163 | 56249.33 | 56762.9 | 57100.2 | 56733.05 | 56672.65 | 56507.7 | 56874.1 | 55713.08 | 56429.8 | 55912.56 | 55858.11 | 55901.61 | 55478 | 55104 |
| 448.1514 | 56345.17 | 56837.15 | 57121.3 | 56615.42 | 56779.4 | 56615.9 | 56781.5 | 55640.2 | 56541.85 | 55785.89 | 55650.63 | 55927.56 | 55728.83 | 54918 |
| 448.2865 | 56387.17 | 56945.65 | 57326.15 | 56785.21 | 56803.8 | 56624.7 | 57136.95 | 55815.23 | 56373.35 | 55719.83 | 55672.89 | 56080.17 | 55775.33 | 55212 |
| 448.4216 | 56171.17 | 56890.75 | 57225.15 | 56631.53 | 56525.05 | 56347.1 | 57192 | 56040.93 | 56352.65 | 55765.28 | 55810.26 | 56128 | 55247.67 | 55227 |
| 448.5567 | 56219.17 | 56811.6 | 56733.7 | 56586.95 | 56608.65 | 56375.4 | 56994.1 | 55946.58 | 56272.95 | 55947.28 | 55620.21 | 56052.28 | 55273.83 | 55581 |
| 448.6918 | 56843.83 | 56728.7 | 56805.1 | 56602.42 | 56772.7 | 56294.45 | 56892.95 | 55803.35 | 56408.7 | 55917.72 | 55913.32 | 55959.06 | 55360.83 | 55138 |
| 448.8269 | 57095.83 | 56967.55 | 56737.3 | 56723 | 56690.7 | 56202.3 | 56739.1 | 55695.63 | 56418.5 | 55896.44 | 55730 | 55958.72 | 55283.33 | 55226 |
| 448.9619 | 56614.67 | 56862.25 | 56933.6 | 56716.79 | 56664.55 | 56316.25 | 56931.2 | 55795.4 | 56438.1 | 56077.83 | 55736.05 | 56106.61 | 55487.67 | 55125 |
| 449.097 | 56465.83 | 56905.75 | 56901.35 | 56756.11 | 56682.4 | 56322.7 | 56750 | 55708.95 | 56510.9 | 56037.61 | 55594.95 | 56058.61 | 55656.17 | 55021 |
| 449.232 | 56342 | 57137.65 | 57115.55 | 56928.89 | 56578.75 | 56391.75 | 56752.75 | 55692.9 | 56349.95 | 56092.72 | 55682.47 | 55965.89 | 55354 | 55615 |
| 449.367 | 56253.67 | 57071 | 57048.55 | 56672 | 56858.75 | 56448.15 | 56769.25 | 55666.55 | 56433.5 | 56079.78 | 55801.42 | 55880.33 | 55440.83 | 55814 |
| 449.502 | 56073.33 | 56875 | 57018.2 | 56664.42 | 56866.7 | 56333.9 | 56793.65 | 55691.58 | 56482.2 | 55994.72 | 55739.58 | 55927.67 | 55435.83 | 55944 |
| 449.6371 | 55810.33 | 56817.45 | 56979.7 | 56480.47 | 57052.2 | 56462.9 | 56847.45 | 55889.9 | 56298.65 | 55983.06 | 55806.84 | 56032.61 | 55519.5 | 55627 |
| 449.7721 | 55659 | 56950.15 | 56734.5 | 56670.68 | 56824.4 | 56637.4 | 56717.85 | 55824.23 | 56339.25 | 56090.17 | 55873.05 | 55909.72 | 55169.33 | 55104 |
| 449.907 | 56020.5 | 56797.1 | 56722.4 | 56952.11 | 56887.55 | 56722.35 | 56826.05 | 55720.15 | 56271.4 | 56196.78 | 55672.53 | 55790.94 | 55317.67 | 55706 |
| 450.042 | 56127.83 | 57029.6 | 56931.65 | 56796.63 | 56768 | 56728.1 | 56965.45 | 55820.33 | 56373.45 | 56100.89 | 55762.68 | 55627.56 | 55571.67 | 55463 |
| 450.177 | 56030.67 | 57115.45 | 56711.15 | 56580.32 | 56825.75 | 56743 | 56855.6 | 55750.73 | 56558.55 | 56126.67 | 55767.16 | 55671.67 | 55651.5 | 55426 |
| 450.3119 | 55811.67 | 56926.95 | 56638.6 | 56652.11 | 56896.05 | 56614.1 | 56742.4 | 55670.13 | 56541.7 | 56252.67 | 55875.58 | 55748.67 | 55697.17 | 56203 |
| 450.4469 | 56096.17 | 56948.3 | 56585.45 | 56727.21 | 56933.9 | 56372.4 | 56728.15 | 55753.65 | 56456.55 | 56142.06 | 55802.63 | 55885.06 | 55570 | 56097 |
| 450.5818 | 56349.17 | 56987.35 | 56871.4 | 56643.05 | 56888.25 | 56339.55 | 56909.75 | 55765.1 | 56452.8 | 56114.06 | 55798.37 | 55978.11 | 55769.33 | 56133 |
| 450.7167 | 55963.83 | 56750.9 | 56851.15 | 56563.95 | 56817.45 | 56355.05 | 56739.65 | 55681.68 | 56501.1 | 55824.67 | 55804.32 | 55841.72 | 55756.83 | 55931 |
| 450.8516 | 55888 | 56657.1 | 57064.9 | 56634.95 | 56724.2 | 56414 | 56548.35 | 55657.28 | 56316.3 | 55812 | 55728.53 | 55828.44 | 55827.33 | 56031 |
| 450.9865 | 56101.5 | 56903.35 | 57086.85 | 56879.58 | 56726.85 | 56394.8 | 56807.75 | 55701.25 | 56449.2 | 55867.78 | 55686 | 55864.89 | 55729.33 | 55316 |
| 451.1214 | 56119.33 | 57093.35 | 56975.9 | 56872.26 | 56959.25 | 56388.75 | 57081.75 | 55871.4 | 56441.05 | 56071.56 | 55770.53 | 55674.17 | 55747.67 | 55515 |
| 451.2563 | 56266.33 | 57198.15 | 56936.85 | 56768.79 | 57105.05 | 56392.2 | 56986.95 | 55948.35 | 56458.2 | 56026.44 | 55692.42 | 55812 | 55735 | 56041 |
| 451.3911 | 56097.5 | 56692.5 | 57225.65 | 56788.21 | 56875.35 | 56463.7 | 56763.15 | 55813.7 | 56242.15 | 56010.72 | 55855.26 | 55970.11 | 55310.83 | 55372 |
| 451.526 | 56422.5 | 56526 | 57295.85 | 56633.11 | 56871.95 | 56315.45 | 56758.15 | 55757.75 | 56119.4 | 56059.11 | 55777.58 | 55787.56 | 55310.67 | 55145 |
| 451.6608 | 55975.83 | 56728.7 | 57005.2 | 56646.95 | 56796.5 | 56553.05 | 56834.15 | 55643.15 | 56147.8 | 56086.17 | 55739.05 | 55745.56 | 55654.33 | 55312 |
| 451.7956 | 56049.83 | 56770.05 | 57052.4 | 56667.89 | 56982.9 | 56404.4 | 56718.8 | 55807.8 | 56269.55 | 56269.61 | 55679 | 55961.89 | 55570.5 | 54992 |
| 451.9305 | 55743.83 | 56761.15 | 56932.25 | 56518.32 | 56860.8 | 56523.3 | 56872.75 | 55755.85 | 56264.5 | 56160.22 | 55607.32 | 55696.78 | 55159.33 | 55145 |
| 452.0653 | 55762.17 | 56893.6 | 56810.6 | 56531.32 | 56753.65 | 56583.05 | 56799.8 | 55764.73 | 56198.35 | 56027.22 | 55698.47 | 55721.94 | 55341.67 | 55873 |
| 452.2 | 56096.83 | 56880.9 | 56885.4 | 56526.79 | 56683.55 | 56704.85 | 56791.85 | 55762.95 | 56183.75 | 55923.44 | 55665.95 | 55835.39 | 55247.5 | 55505 |
| 452.3348 | 55626.5 | 56686.3 | 56966.95 | 56609.89 | 56937.1 | 56697.75 | 56703.05 | 55712.25 | 56221.9 | 55867.28 | 55647.26 | 55777.83 | 55485.67 | 55748 |
| 452.4696 | 55978 | 56920.75 | 56824.95 | 56665.79 | 56886.3 | 56654.9 | 56666.15 | 55802.05 | 56297.75 | 55849.17 | 55787.26 | 55772.89 | 55580.83 | 55617 |
| 452.6043 | 56104.17 | 57077.9 | 56758.15 | 56543.11 | 56876.1 | 56561 | 56519.35 | 55810.65 | 56322.25 | 55864.33 | 55680.37 | 56017.33 | 56036.33 | 55831 |
| 452.7391 | 55957.17 | 56889.35 | 56955.3 | 56638.95 | 56970.55 | 56329.65 | 56528.8 | 55738.78 | 56419 | 56032.39 | 55820.79 | 55846.33 | 55760.33 | 55957 |
| 452.8738 | 56014.5 | 56912.1 | 57083.85 | 56567.79 | 56940.65 | 56253.35 | 56765.65 | 55838.75 | 56452.05 | 56006.61 | 55867.21 | 55880.06 | 55510.67 | 56438 |
| 453.0085 | 55948.17 | 56823.55 | 56821.75 | 56645.42 | 56628.85 | 56531.35 | 56772.95 | 55757.13 | 56324.85 | 55990.33 | 55952.11 | 55827.94 | 55634 | 56220 |
| 453.1433 | 55850.67 | 56779.95 | 56979.5 | 56686.58 | 56573.05 | 56590.3 | 56526.85 | 55885.83 | 56317.7 | 55938.78 | 55875.16 | 55987.83 | 55446.5 | 56244 |
| 453.278 | 56006.33 | 56492.2 | 57152.3 | 56809.32 | 56709.55 | 56486 | 56577.15 | 55947.73 | 56390.65 | 55962.11 | 56027.89 | 55922.33 | 55330 | 55290 |
| 453.4126 | 56150 | 56528.3 | 57220.4 | 56588.05 | 56763.3 | 56555.9 | 56850.7 | 55873.35 | 56190.25 | 55999.72 | 56022.47 | 55889.06 | 55376.67 | 54777 |
| 453.5473 | 56050.67 | 56803.75 | 56985.9 | 56527.74 | 56647.65 | 56629.95 | 56715.95 | 55871.9 | 56381.3 | 56093.56 | 55830.79 | 55761.89 | 55357 | 55221 |
| 453.682 | 56125.33 | 56886.45 | 56985.05 | 56520.63 | 56656 | 56507.6 | 56813.1 | 55809.05 | 56454 | 56280.78 | 55866.74 | 56003.28 | 55351 | 55092 |
| 453.8166 | 56071.17 | 56685.15 | 56978.8 | 56695.89 | 56639.7 | 56485.1 | 56708.65 | 55658.7 | 56273.45 | 56207.56 | 55945.68 | 56210.56 | 55409.33 | 54850 |
| 453.9513 | 56137.83 | 56825.95 | 56868.1 | 56751.89 | 56625.6 | 56404.9 | 56763.9 | 55740.25 | 56646.2 | 56114 | 55787.16 | 55783.83 | 55382 | 55667 |
| 454.0859 | 56166.83 | 56790.45 | 56951 | 56968.42 | 56578.15 | 56542 | 56896.35 | 55850.85 | 56454.15 | 56109.17 | 55923.58 | 55803 | 55106.33 | 55914 |
| 454.2205 | 56144.67 | 56856.65 | 57063.8 | 56837.84 | 56745.8 | 56582.3 | 56794.2 | 55918.4 | 56375.4 | 56152.44 | 55751.32 | 55754.28 | 55314 | 55881 |
| 454.3551 | 56333.5 | 57161.45 | 57037.65 | 56838.21 | 56766.4 | 56515.55 | 56705.25 | 55752.3 | 56302.4 | 56109.78 | 55838.79 | 55738.56 | 55232.83 | 55248 |
| 454.4897 | 56415.67 | 57118.9 | 57154.3 | 56760.68 | 56798.85 | 56291.75 | 56842.4 | 55744.83 | 56336.35 | 56114.06 | 55833.05 | 55853.44 | 55187.33 | 55277 |
| 454.6243 | 56201.17 | 57006.4 | 56848.15 | 56610.16 | 57119 | 56578.4 | 56863.65 | 55691.85 | 56396.2 | 56017.33 | 55736.63 | 56024.94 | 55083.17 | 55329 |
| 454.7588 | 56037.17 | 57568.1 | 56989.5 | 56650.47 | 56922.55 | 56237.35 | 56812 | 55778.23 | 56325.9 | 55953.83 | 55736.79 | 56015.11 | 55133.17 | 55412 |
| 454.8934 | 56159.67 | 57354.6 | 57041.85 | 56504.42 | 56876.2 | 56291.7 | 56809.1 | 55708.93 | 56510.95 | 55899.78 | 55830.79 | 56032.78 | 55313.67 | 54945 |
| 455.0279 | 55956.33 | 57193.35 | 57325.4 | 56630.26 | 56927.95 | 56198.6 | 56850.2 | 55853.4 | 56475.05 | 56010.78 | 56040.84 | 55843.56 | 55355.17 | 54960 |
| 455.1625 | 56255.33 | 57177.25 | 57099.8 | 56704.21 | 56760.75 | 56395.55 | 56816.65 | 55894.13 | 56405.1 | 55886.11 | 55975.47 | 55836.33 | 55486 | 54891 |
| 455.297 | 56079.17 | 56968.9 | 56779.6 | 56804.26 | 56874.7 | 56387.15 | 57043.05 | 55972.68 | 56405.8 | 55968.44 | 56043.63 | 56018.11 | 55549.17 | 55555 |
| 455.4315 | 56037.17 | 57066.05 | 57001.8 | 56809.74 | 56879.55 | 56535.85 | 56939.4 | 55861.3 | 56456.15 | 55992.44 | 55973.26 | 55865.39 | 55798.17 | 56130 |
| 455.566 | 56099.17 | 57152.7 | 56807.65 | 56838.79 | 56984.5 | 56625.2 | 57140.9 | 55903.73 | 56327.1 | 56158.33 | 55748.95 | 55848 | 55527.17 | 55559 |
| 455.7005 | 56253.17 | 57041.75 | 56896.6 | 56957.11 | 56931.4 | 56553.45 | 56977.25 | 55834.73 | 56605.45 | 56282.67 | 55740.89 | 55869.33 | 55470 | 55756 |
| 455.8349 | 56478.17 | 57052.15 | 56819.4 | 56666.21 | 56809.1 | 56660.6 | 57114.05 | 55811.55 | 56662.4 | 56127.28 | 55768.68 | 55999.56 | 55714.5 | 56013 |
| 455.9694 | 55990.5 | 57137.25 | 56862.6 | 56599.42 | 56818.75 | 56454.8 | 57049.6 | 55923.63 | 56451.9 | 56104.67 | 55913.68 | 55937.67 | 55527.33 | 56131 |
| 456.1038 | 56350.5 | 56921.7 | 57032.8 | 56533.11 | 56914.15 | 56447.3 | 56912.7 | 55911.25 | 56451 | 56010.39 | 55852.05 | 55848.78 | 55932.33 | 55786 |
| 456.2383 | 56332 | 56826.3 | 57090.8 | 56503.74 | 56876.3 | 56468.9 | 56867.9 | 55806.93 | 56277.35 | 56129.83 | 55947.42 | 55871.83 | 55839.17 | 56498 |
| 456.3727 | 56196.5 | 56766.1 | 57188.75 | 56667.37 | 56837.35 | 56298.95 | 56699.4 | 55905.95 | 56398.7 | 56128.94 | 55863.21 | 55954.94 | 55703.5 | 55808 |
| 456.5071 | 56213.5 | 56941.45 | 57163.4 | 56823.68 | 56917.4 | 56485.6 | 56910.4 | 55908.83 | 56307.55 | 56075.61 | 55919.95 | 55886.5 | 55745.33 | 55493 |
| 456.6415 | 56228.83 | 56884.65 | 57007.7 | 56782.21 | 56742.9 | 56573.35 | 56869.5 | 55945.8 | 56521.3 | 56382.94 | 55945.42 | 55849.39 | 55453.5 | 55102 |
| 456.7759 | 56404.33 | 57029 | 56906.2 | 56866.21 | 56536.45 | 56339.95 | 56860.1 | 55861.53 | 56389.15 | 56109.17 | 56137.32 | 55867.78 | 55736.5 | 55222 |
| 456.9103 | 56655.67 | 57215.05 | 56906.05 | 56752.11 | 56697.55 | 56291.45 | 56825.95 | 55822.78 | 56236.8 | 56140.94 | 56048 | 55847.44 | 55482.5 | 55663 |
| 457.0446 | 56151 | 56998.05 | 56970.75 | 56683.53 | 56726.25 | 56409.8 | 56772.85 | 55865.63 | 56159.15 | 56117.33 | 55869.16 | 55869.17 | 55636.83 | 55217 |
| 457.1789 | 55884.67 | 56882.2 | 57097.1 | 56633.68 | 56590.55 | 56330.35 | 56743.6 | 55962.43 | 56209.65 | 56066.11 | 55807.47 | 56079.89 | 55905.17 | 55869 |
| 457.3133 | 56241.67 | 56827.8 | 57147.25 | 56601.84 | 56546 | 56339.2 | 56815.65 | 55911.25 | 56380.5 | 56118.83 | 55879.47 | 56041.94 | 55844.5 | 55506 |
| 457.4476 | 56337.33 | 57110.2 | 57021.5 | 56657.63 | 56557.75 | 56462.05 | 57084.85 | 55865.75 | 56404.85 | 56136.72 | 55825.95 | 55779.06 | 55879.17 | 55500 |
| 457.5819 | 56412.17 | 56995.75 | 56919.95 | 56909.11 | 56541.95 | 56289.7 | 56758.05 | 55889.08 | 56441.85 | 56036.78 | 55811.21 | 55935.5 | 55724 | 56165 |
| 457.7162 | 56381.83 | 57026.6 | 57041.7 | 56696.95 | 56707.85 | 56298.1 | 56673.45 | 55887.93 | 56750.05 | 55978.56 | 55907 | 56013.94 | 55737.67 | 55385 |
| 457.8505 | 56329.17 | 57056 | 56990.45 | 56482.26 | 56866.5 | 56372.25 | 56779.05 | 55741.05 | 56460.15 | 56016.61 | 56002.95 | 55891.44 | 55861.83 | 55973 |
| 457.9847 | 56582.17 | 56977.55 | 56951.85 | 56673.63 | 57190.1 | 56471 | 56606.75 | 55749.53 | 56272.9 | 56143.78 | 55874.79 | 55759.78 | 55639.5 | 55342 |
| 458.119 | 56317.67 | 56897.1 | 57043.15 | 56785.68 | 57140.4 | 56408.8 | 56580.95 | 55891.98 | 56252.85 | 56212.28 | 55915.47 | 56064.11 | 55640.33 | 55784 |
| 458.2532 | 56386.5 | 56868.75 | 56964.95 | 56685.37 | 56791.1 | 56565.1 | 56697.55 | 55935.33 | 56551.75 | 56211 | 55697.63 | 56013.17 | 55322.5 | 55801 |
| 458.3875 | 56374.83 | 56812.2 | 57148.2 | 56734.11 | 56999.5 | 56853.85 | 56622.35 | 55816.35 | 56612.3 | 55944.22 | 55780.74 | 56024.39 | 55302 | 56302 |
| 458.5217 | 56150.67 | 56883.15 | 57296.2 | 56706.26 | 57064.3 | 56719.05 | 56859.65 | 55687.9 | 56528.75 | 56052.5 | 55742.89 | 56004.61 | 55646.33 | 55518 |
| 458.6559 | 56128.17 | 56962.2 | 57239.45 | 56614.74 | 56591.45 | 56719.1 | 57076.45 | 55834.33 | 56524.9 | 56134.39 | 55788.47 | 55993.06 | 55636.5 | 55374 |
| 458.7901 | 56366.33 | 57067 | 57373.55 | 56586.05 | 56610.1 | 56623.65 | 56733.85 | 55832.4 | 56560.15 | 56164.67 | 55959.68 | 55883 | 55573.33 | 56189 |
| 458.9243 | 56217.33 | 57038.3 | 57186.65 | 56717.05 | 56811.95 | 56531.1 | 56802.25 | 55847.18 | 56616.35 | 56174.06 | 55886.89 | 55924.17 | 55624.33 | 55874 |
| 459.0584 | 56422.5 | 56970.3 | 57232.8 | 56447.37 | 56835 | 56554.15 | 56620.15 | 55844.88 | 56693.85 | 56150.67 | 55742.32 | 55924.44 | 55400 | 55703 |
| 459.1926 | 56397.33 | 56929.25 | 57079.15 | 56602.58 | 56791.5 | 56450.05 | 56685.9 | 55810.6 | 56467.15 | 56118.89 | 55847.89 | 55890.61 | 55562.67 | 55335 |
| 459.3268 | 56331.17 | 56914.85 | 57211 | 56584.89 | 56564.7 | 56576.9 | 56722.6 | 55809.2 | 56533.55 | 56103.06 | 55739.16 | 55918.11 | 55633.67 | 56085 |
| 459.4609 | 56226.17 | 57110.75 | 56957.3 | 56690.21 | 56906.55 | 56277.25 | 56570.1 | 55797.48 | 56513.95 | 56196.89 | 55656 | 55909.06 | 55513.67 | 56221 |
| 459.595 | 56540.17 | 56891.15 | 57111.25 | 56570.11 | 56715.55 | 56464.4 | 56376.35 | 55786.23 | 56458.4 | 56197.72 | 55671.95 | 56004.67 | 55587.67 | 56923 |
| 459.7291 | 56601.5 | 56940 | 57078.3 | 56642.53 | 56935.8 | 56343.35 | 56449.65 | 55796.18 | 56158.85 | 56048.61 | 55758 | 55963.67 | 55466.33 | 56072 |
| 459.8632 | 56352.17 | 56735.55 | 57038.5 | 56386.42 | 56959.65 | 56391.5 | 56615.9 | 55827.65 | 56317.65 | 56048.33 | 55691.47 | 55844 | 55419.17 | 55817 |
| 459.9973 | 56139.17 | 56983.1 | 56926.45 | 56543.32 | 56854.85 | 56478.4 | 56659.3 | 55739.73 | 56287.5 | 56255.06 | 55783.95 | 55996.67 | 55714 | 55343 |
| 460.1313 | 56205.5 | 56787.1 | 56956.65 | 56682.58 | 56648.25 | 56490.25 | 56763.05 | 55815.23 | 56371.15 | 55987.61 | 55888.42 | 55804.61 | 55740.83 | 55637 |
| 460.2654 | 56264.33 | 57061.65 | 57064.25 | 56623.95 | 56870.9 | 56488.95 | 56663.2 | 55692.75 | 56443.9 | 56089.56 | 56065.21 | 55965.28 | 55785.17 | 55883 |
| 460.3994 | 56529.67 | 56971.6 | 57126.6 | 56421.11 | 56808.35 | 56546.35 | 56566.7 | 55728.5 | 56412.45 | 56042.89 | 55927.79 | 55968.28 | 55412.17 | 55200 |
| 460.5334 | 56504.17 | 56950 | 57045.45 | 56680.53 | 56899 | 56540.05 | 56623.55 | 55756.03 | 56383.85 | 55958.56 | 56030.37 | 56089.5 | 55686.5 | 55987 |
| 460.6674 | 56107.17 | 57169.65 | 57115.9 | 56768.95 | 56877.55 | 56619.7 | 56792.7 | 55770.6 | 56387.55 | 55936.11 | 56062.63 | 55961.06 | 55461.33 | 56628 |
| 460.8014 | 56485.33 | 57160.45 | 56939.4 | 56707.16 | 56843.8 | 56527.6 | 56853.8 | 55796.48 | 56337.8 | 56114.28 | 55859.58 | 55952.83 | 55571.5 | 55790 |
| 460.9354 | 56191 | 57089.3 | 56910.15 | 56526.21 | 56778.75 | 56414.95 | 56668.8 | 55782.78 | 56452.4 | 56112.28 | 55826.47 | 55953.11 | 55717.83 | 55866 |
| 461.0694 | 56001.83 | 57013.15 | 56864.55 | 56491.26 | 56833.45 | 56428.35 | 56510.6 | 55782.38 | 56413.6 | 56015.5 | 56050.21 | 55978.72 | 55468 | 56357 |
| 461.2033 | 56183.83 | 57048.7 | 56967.6 | 56836.68 | 56904.4 | 56576.9 | 56713.2 | 55760.55 | 56490.6 | 55926.22 | 55855.95 | 55873.83 | 55496.83 | 55719 |
| 461.3373 | 56221 | 57014.45 | 56894.35 | 56685.84 | 56527.9 | 56514.95 | 56720.8 | 55883.83 | 56450.55 | 55930 | 55832.16 | 55847.44 | 55456.33 | 55472 |
| 461.4712 | 56539.33 | 56964.8 | 56793.75 | 56555.74 | 56500.75 | 56377.75 | 56688.25 | 55841.9 | 56390.95 | 56104.39 | 55862.11 | 55936.5 | 55617.5 | 55272 |
| 461.6051 | 56463.17 | 56948.65 | 56985 | 56426 | 56636.3 | 56241.05 | 56766.35 | 55826.18 | 56413.55 | 56053.44 | 55905.47 | 55751.44 | 55494.5 | 55501 |
| 461.739 | 56585.17 | 56824.2 | 56928 | 56281.11 | 56629.55 | 56185.7 | 56707.75 | 55841.98 | 56407.2 | 56032.44 | 55798.68 | 55580.61 | 55495.17 | 55250 |
| 461.8729 | 56359 | 56642.55 | 56972.9 | 56524.53 | 56648.2 | 56232.3 | 56608.3 | 55777.25 | 56738.7 | 56082.11 | 55779 | 55675.78 | 55624.17 | 55771 |
| 462.0068 | 56343.5 | 56628.55 | 56888.8 | 56538.37 | 56579.4 | 56181.95 | 56611.9 | 55838.23 | 56542.3 | 55967.39 | 55556.79 | 55837.94 | 55705.5 | 55739 |
| 462.1407 | 56131.83 | 56933.95 | 56929.5 | 56389.79 | 56517.6 | 56381.95 | 56657.85 | 55785.9 | 56442.05 | 55881.78 | 55672.53 | 55788.61 | 55437.83 | 55334 |
| 462.2745 | 56274.83 | 56671.85 | 57062.8 | 56511.21 | 56463.25 | 56242.9 | 56497.05 | 55744.68 | 56373.9 | 55768.61 | 55772 | 55832.56 | 55210.17 | 55554 |
| 462.4084 | 56288.5 | 56840 | 56980.05 | 56429.89 | 56633.3 | 56328.75 | 56518.15 | 55690.33 | 56463.45 | 55780.06 | 55858.68 | 56116.5 | 55275.83 | 55539 |
| 462.5422 | 56211.17 | 56901 | 57128.8 | 56361.11 | 56646.55 | 56288.25 | 56624.15 | 55667.75 | 56216.7 | 55852.72 | 55903.68 | 56148.83 | 55422.33 | 55590 |
| 462.676 | 56090.5 | 56835.4 | 57018.35 | 56435.68 | 56785.15 | 56273.75 | 56555.4 | 55606.9 | 56222.95 | 55860.28 | 55821.95 | 56000.17 | 55661.67 | 55104 |
| 462.8098 | 55858.5 | 56751.65 | 56837.05 | 56621.58 | 56797.75 | 56299.2 | 56703.6 | 55672.38 | 56281.55 | 55911.61 | 55619.16 | 55763.72 | 55717.83 | 56116 |
| 462.9435 | 55789.5 | 56642.65 | 56782.75 | 56603.74 | 56684.1 | 56281.05 | 56563.55 | 55695.7 | 56193.55 | 55841.11 | 55651 | 55642.22 | 55505 | 56177 |
| 463.0773 | 56065.17 | 56596.05 | 56708 | 56734.89 | 56736.5 | 56317.75 | 56767 | 55705.98 | 56026.1 | 55817.33 | 55828.16 | 55793.06 | 55347.5 | 55444 |
| 463.2111 | 55701.67 | 56829.55 | 56669.45 | 56641.58 | 56629.25 | 56467.05 | 56664.35 | 55624.63 | 56119.6 | 55938.5 | 55602.42 | 55862.61 | 55392.33 | 55251 |
| 463.3448 | 55964.17 | 56663.15 | 56658.8 | 56508.47 | 56603.4 | 56392.45 | 56559.15 | 55605.85 | 56160.4 | 55978.28 | 55707.53 | 55684.94 | 55138.17 | 55614 |
| 463.4786 | 56122.5 | 56791 | 56641.9 | 56427.21 | 56466.1 | 56152.8 | 56559.55 | 55644.88 | 56181.6 | 56049.83 | 55813.79 | 55685.78 | 55266.83 | 55299 |
| 463.6122 | 56210.17 | 56854.5 | 57054.3 | 56268.89 | 56484.2 | 56195.2 | 56361.05 | 55787.4 | 56254.9 | 55872.44 | 55745.47 | 55561.39 | 55359 | 55449 |
| 463.7459 | 56107.33 | 56675.45 | 56773.35 | 56340.84 | 56556.25 | 56312.6 | 56270.75 | 55679.88 | 56109 | 55863.61 | 55788.63 | 55748.28 | 55321.17 | 56482 |
| 463.8796 | 56320.17 | 56737.3 | 56570.95 | 56385.79 | 56453.85 | 56259.1 | 56251.6 | 55645.25 | 55919.35 | 55995.56 | 55602.89 | 55716.72 | 55155.5 | 55752 |
| 464.0133 | 56066.83 | 56509.45 | 56502.4 | 56364.74 | 56420.35 | 56068.05 | 56368.35 | 55511.48 | 55953.4 | 55851.78 | 55316.95 | 55661.5 | 55185.67 | 55318 |
| 464.147 | 56122.33 | 56366.85 | 56519.35 | 56244.89 | 56370.25 | 55893.95 | 56214 | 55579.65 | 55908.9 | 55709.22 | 55602.53 | 55602.94 | 55265.33 | 56010 |
| 464.2806 | 55806.83 | 56260.6 | 56445.65 | 56091.32 | 56228 | 55697.35 | 56358.2 | 55493.73 | 55976.6 | 55546.5 | 55452 | 55438.89 | 55204.17 | 54808 |
| 464.4143 | 55788.33 | 56131.9 | 56459.55 | 56189.37 | 56122.6 | 55554 | 56119.1 | 55431.68 | 55790.75 | 55517.67 | 55339.79 | 55338.56 | 55278.67 | 54920 |
| 464.5479 | 55689.5 | 56020.1 | 56436.7 | 56239.63 | 56093.55 | 55763.55 | 56101.9 | 55362.95 | 55795.9 | 55595.89 | 55232.16 | 55361 | 55014.17 | 54836 |
| 464.6815 | 55735 | 56241.75 | 56213.05 | 55960.26 | 56154.5 | 55825.2 | 55997.75 | 55205.4 | 55702.6 | 55554.44 | 55168.89 | 55289.89 | 55064.33 | 55076 |
| 464.815 | 55745 | 56220.05 | 56052.45 | 55799.53 | 56140.65 | 55824.7 | 55867.6 | 55145.53 | 55737.3 | 55290.22 | 55011 | 55264.61 | 54915.5 | 55382 |
| 464.9486 | 55530.67 | 55900.5 | 55875.55 | 55709.79 | 56236.85 | 55733.35 | 55724.9 | 55181.9 | 55524.3 | 55337.39 | 55101.68 | 55144.61 | 54660.17 | 55495 |
| 465.0822 | 55243.33 | 55790.2 | 55721.7 | 55633.11 | 55923.05 | 55498.8 | 55798.5 | 54983.63 | 55376.9 | 55170.83 | 54991.47 | 54996.89 | 54515.33 | 54919 |
| 465.2157 | 54961.83 | 55636.95 | 55612.85 | 55663.47 | 55816.7 | 55280.4 | 55777.15 | 54908.83 | 55498.8 | 55039.5 | 54953.95 | 54921.5 | 54619.5 | 54722 |
| 465.3492 | 54938.33 | 55690 | 55553.5 | 55445.37 | 55665.35 | 55203.9 | 55626.15 | 54798.83 | 55472.65 | 54823.33 | 55004 | 54837 | 54568.5 | 54262 |
| 465.4828 | 54889 | 55437.5 | 55543.85 | 55206 | 55321.55 | 55335.8 | 55522.95 | 54734.55 | 55275.1 | 54819.22 | 54919.58 | 54727.78 | 54613.67 | 54291 |
| 465.6163 | 54808.67 | 55298.25 | 55380.95 | 54994.95 | 55233.8 | 55135.85 | 55305.15 | 54578.7 | 54928 | 54720.61 | 54475.26 | 54546.56 | 54398.83 | 54466 |
| 465.7498 | 54644.33 | 55123.4 | 55159.85 | 54918.47 | 55043.55 | 55006.45 | 55007.1 | 54430.08 | 54796.7 | 54480.89 | 54436.37 | 54522.44 | 54370 | 54394 |
| 465.8832 | 54382.33 | 54866.8 | 54817.2 | 54695.95 | 54735.15 | 54658.5 | 54736.55 | 54219.13 | 54605.35 | 54319.67 | 54248 | 54206.22 | 54247.33 | 53861 |
| 466.0167 | 54192 | 54391.85 | 54481.05 | 54373.21 | 54451.85 | 54233.55 | 54337.55 | 54066.88 | 54290.95 | 54053.44 | 54062.63 | 54017.22 | 54022.17 | 53715 |
| 466.1501 | 53761.83 | 54028.25 | 54106.65 | 53999.63 | 54051.65 | 53955 | 54005.6 | 53835.33 | 54021.4 | 53854.28 | 53832.58 | 53819.72 | 53872 | 53656 |
| 466.2836 | 53638.17 | 53769.9 | 53820.5 | 53781 | 53782.9 | 53759.3 | 53809.9 | 53675.6 | 53771.4 | 53714.56 | 53702 | 53702.5 | 53692 | 53635 |
| 466.417 | 53586.17 | 53656.4 | 53712.6 | 53689 | 53707.6 | 53697.3 | 53715.6 | 53629.08 | 53692.7 | 53668.83 | 53669.11 | 53658.94 | 53656.33 | 53589 |
| 466.5504 | 53572.33 | 53625.45 | 53674.5 | 53654.16 | 53673.85 | 53661 | 53681 | 53611.15 | 53666.1 | 53656.67 | 53648.21 | 53639.11 | 53646.67 | 53586 |
| 466.6838 | 53559.33 | 53597.15 | 53657.5 | 53639.11 | 53653.3 | 53647.6 | 53664.05 | 53597.85 | 53648.55 | 53643.89 | 53635.63 | 53626.89 | 53636 | 53581 |
| 466.8171 | 53547.83 | 53588.6 | 53648.85 | 53627.42 | 53644.55 | 53639.3 | 53654.25 | 53592.7 | 53640 | 53635.22 | 53633.21 | 53620.78 | 53633.83 | 53555 |
| 466.9505 | 53543.67 | 53575.6 | 53630.4 | 53616.63 | 53632.8 | 53626.3 | 53639.5 | 53582.83 | 53632.2 | 53627.78 | 53623.26 | 53609.94 | 53623 | 53561 |
| 467.0838 | 53526.33 | 53558.2 | 53620.45 | 53602.37 | 53618.25 | 53617.5 | 53629.5 | 53580.78 | 53626.1 | 53620.83 | 53620.21 | 53604.39 | 53620.5 | 53560 |
| 467.2172 | 53529.17 | 53556.55 | 53612.85 | 53602.42 | 53611.85 | 53609.7 | 53626.8 | 53569.78 | 53617.3 | 53617.11 | 53612 | 53601.44 | 53611 | 53562 |
| 467.3505 | 53517.5 | 53548.9 | 53602.55 | 53590.16 | 53604.45 | 53605.7 | 53616.7 | 53565.08 | 53604.85 | 53610 | 53607.89 | 53597.83 | 53608.67 | 53552 |
| 467.4838 | 53510 | 53537.75 | 53597.4 | 53588.32 | 53593.7 | 53600.25 | 53609.45 | 53561.63 | 53600.95 | 53607.28 | 53604.05 | 53592 | 53605.83 | 53548 |
| 467.6171 | 53514.5 | 53536.1 | 53590.3 | 53580.11 | 53595.15 | 53592.35 | 53601.8 | 53556.38 | 53597.95 | 53600.61 | 53599.05 | 53589.67 | 53595.83 | 53551 |
| 467.7503 | 53512.33 | 53531.1 | 53586.75 | 53580.63 | 53592.5 | 53590.8 | 53605.4 | 53556.68 | 53591.8 | 53597.5 | 53597.05 | 53585.72 | 53605.5 | 53565 |
| 467.8836 | 53498.5 | 53525.75 | 53583.35 | 53579.32 | 53588.05 | 53587.6 | 53594.6 | 53552.85 | 53590.05 | 53595.39 | 53592 | 53584.33 | 53601.83 | 53539 |
| 468.0169 | 53495.67 | 53528.35 | 53584.25 | 53571.74 | 53581.65 | 53585.9 | 53596.8 | 53551.2 | 53590.8 | 53595.56 | 53594.42 | 53585.06 | 53601.33 | 53543 |
| 468.1501 | 53504.67 | 53519.05 | 53576.9 | 53568.79 | 53578.4 | 53579.2 | 53587.25 | 53545.63 | 53581.45 | 53592.61 | 53589.89 | 53574.39 | 53593.83 | 53530 |
| 468.2833 | 53490.83 | 53516.55 | 53572.05 | 53567.16 | 53578.55 | 53580.85 | 53589.2 | 53545.13 | 53579.35 | 53590.78 | 53586.53 | 53572.67 | 53594.33 | 53544 |
| 468.4165 | 53498 | 53516 | 53575.25 | 53570.53 | 53576 | 53583.85 | 53588.95 | 53551 | 53583.2 | 53586.28 | 53589.84 | 53577.72 | 53597.83 | 53528 |
| 468.5497 | 53497.83 | 53513.95 | 53573.2 | 53567.95 | 53574.5 | 53576.55 | 53583 | 53547.1 | 53577.65 | 53589.44 | 53588 | 53578.33 | 53587.33 | 53545 |
| 468.6828 | 53496.17 | 53516.9 | 53573.7 | 53566.16 | 53578.3 | 53580.65 | 53588.5 | 53547.58 | 53579.65 | 53591.72 | 53592.32 | 53575.78 | 53597.83 | 53537 |
| 468.816 | 53492.33 | 53510.15 | 53575.1 | 53563.79 | 53570.75 | 53575.35 | 53583.1 | 53548.48 | 53573.4 | 53586.94 | 53589.37 | 53577.78 | 53595.17 | 53525 |
| 468.9491 | 53499 | 53513.95 | 53567.1 | 53560.68 | 53572.9 | 53572.55 | 53583.05 | 53546.23 | 53577.6 | 53587.17 | 53585 | 53572.78 | 53588.83 | 53543 |
| 469.0822 | 53491.83 | 53507.15 | 53566.1 | 53562.89 | 53569.1 | 53575.8 | 53583.05 | 53545.6 | 53579.5 | 53585.61 | 53586.84 | 53572.33 | 53588.67 | 53528 |
| 469.2153 | 53486.17 | 53503.5 | 53562.4 | 53557.58 | 53567.05 | 53566.6 | 53578.3 | 53544.7 | 53574.15 | 53582.44 | 53583.58 | 53572.44 | 53579.67 | 53540 |
| 469.3485 | 53487.33 | 53508.85 | 53567.25 | 53562.16 | 53566.3 | 53577.65 | 53578.55 | 53542.9 | 53576.75 | 53583.94 | 53589.63 | 53573.06 | 53591.67 | 53543 |
| 469.4815 | 53480.33 | 53495.8 | 53560.1 | 53552.05 | 53561 | 53568.8 | 53573.15 | 53538.8 | 53570.4 | 53580.28 | 53576.95 | 53570.39 | 53583.67 | 53505 |

**Spectral Range 2: 568 – 704 nm
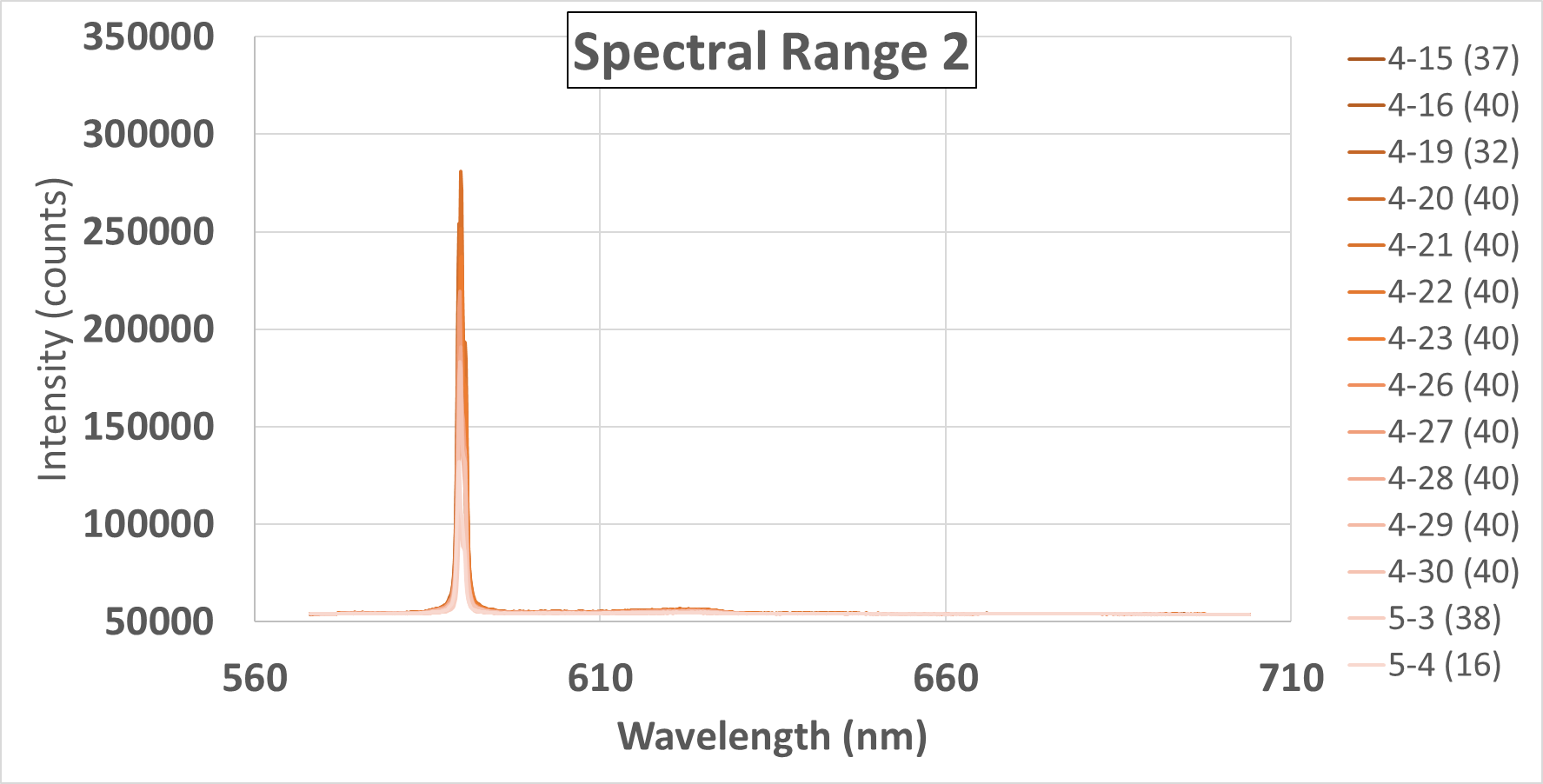
**

**Figure S2:** Range 2 - Data collected between 4-15 and 5-4 showing sodium (Na) emission lines.

**Table S2:** Range 2 - Column labels indicate the collection date and number of useable spectra included in the average.

| wL(nm) | 4-15 (37) | 4-16 (40) | 4-19 (32) | 4-20 (40) | 4-21 (40) | 4-22 (40) | 4-23 (40) | 4-26 (40) | 4-27 (40) | 4-28 (40) | 4-29 (40) | 4-30 (40) | 5-3 (38) | 5-4 (16) |
| --- | --- | --- | --- | --- | --- | --- | --- | --- | --- | --- | --- | --- | --- | --- |
| 568.06 | 53845.65 | 53896.18 | 53853.78 | 53902.13 | 53927.93 | 53874.65 | 53911.83 | 53863.93 | 53876.93 | 53919.1 | 53896.75 | 53915.53 | 53941.16 | 53920.5 |
| 568.1948 | 53845.7 | 53881 | 53915.16 | 53887.45 | 53870.98 | 53900.1 | 53914.78 | 53888.23 | 53833.08 | 53961.73 | 53909.65 | 53890.18 | 53892.61 | 53918.81 |
| 568.3296 | 53853.65 | 53885.08 | 53942.59 | 53886.28 | 53873.5 | 53874.3 | 53899.73 | 53971.98 | 53898.8 | 53871.15 | 53919.65 | 53902.75 | 53895.29 | 53900.5 |
| 568.4644 | 53856.54 | 53886.18 | 53925.28 | 53861.75 | 53878.05 | 53869.93 | 53868.35 | 53950.7 | 53897.6 | 53864.65 | 53902.73 | 53916.83 | 53893.79 | 53868.63 |
| 568.5992 | 53852.41 | 53886.85 | 53881.09 | 53848.35 | 53889.3 | 53868.73 | 53857.4 | 53894.53 | 53872.58 | 53861.4 | 53903.3 | 53913.18 | 53886.24 | 53881.13 |
| 568.734 | 53847.86 | 53884.23 | 53850.59 | 53858.73 | 53890.35 | 53864.75 | 53857.5 | 53854.85 | 53866.03 | 53850.53 | 53901.25 | 53910.1 | 53879.58 | 53887.19 |
| 568.8687 | 53840.97 | 53880.7 | 53831.53 | 53865.43 | 53895.03 | 53866.53 | 53859.2 | 53842.75 | 53865.75 | 53846.13 | 53900.35 | 53901.85 | 53879.39 | 53895.44 |
| 569.0035 | 53832.08 | 53874.85 | 53822.69 | 53866.28 | 53887.8 | 53862.23 | 53852.3 | 53831 | 53865.18 | 53847.4 | 53892.55 | 53897.6 | 53870.97 | 53897.06 |
| 569.1383 | 53828.89 | 53871.15 | 53820.44 | 53877.68 | 53889.65 | 53865.05 | 53854.33 | 53830.95 | 53863.08 | 53847.63 | 53895.33 | 53892.98 | 53871.39 | 53901.56 |
| 569.2731 | 53821.76 | 53864.93 | 53813.63 | 53874.3 | 53884.98 | 53862.73 | 53848.83 | 53823.45 | 53857.73 | 53848.63 | 53890.1 | 53885.88 | 53862.53 | 53901.06 |
| 569.4078 | 53822.62 | 53869.18 | 53814.41 | 53872.93 | 53887.43 | 53858.78 | 53848.88 | 53820.55 | 53861.38 | 53849.6 | 53886.78 | 53887.15 | 53863.5 | 53908.75 |
| 569.5426 | 53816.27 | 53859.7 | 53810.06 | 53874.53 | 53880.95 | 53855.85 | 53841.75 | 53819.83 | 53855.8 | 53846.5 | 53885.63 | 53878.55 | 53859.79 | 53897.31 |
| 569.6774 | 53814.24 | 53859.5 | 53810.5 | 53869.9 | 53883.1 | 53860.18 | 53844.68 | 53816.68 | 53856.55 | 53845.1 | 53883.05 | 53876.95 | 53854.32 | 53900.94 |
| 569.8121 | 53812.92 | 53855.73 | 53806.63 | 53872.38 | 53881.48 | 53854.33 | 53839.53 | 53815.58 | 53853.75 | 53844.18 | 53880.6 | 53874.4 | 53855.39 | 53899.75 |
| 569.9469 | 53817.16 | 53857.15 | 53813.16 | 53875.05 | 53884.15 | 53858.58 | 53842.8 | 53815.68 | 53856.8 | 53844.65 | 53883.85 | 53879.68 | 53856.74 | 53900.81 |
| 570.0816 | 53822.97 | 53861.8 | 53813.16 | 53881.65 | 53890.3 | 53867.68 | 53851.83 | 53824.3 | 53863.05 | 53850.53 | 53887.68 | 53882.95 | 53860.47 | 53907.69 |
| 570.2164 | 53811 | 53851.58 | 53801.03 | 53870.43 | 53880.25 | 53847.2 | 53837.6 | 53807.1 | 53847.68 | 53838.48 | 53877.5 | 53869.98 | 53844 | 53894.81 |
| 570.3511 | 53810.14 | 53851.1 | 53800.63 | 53873.43 | 53878.55 | 53851.6 | 53837.5 | 53811.93 | 53849.88 | 53835.83 | 53873.18 | 53872.03 | 53845.58 | 53894.69 |
| 570.4859 | 53808.22 | 53851.53 | 53803.34 | 53866.53 | 53873.68 | 53846.5 | 53834.03 | 53806.4 | 53848.23 | 53835.13 | 53872.1 | 53869.5 | 53847.34 | 53890.25 |
| 570.6206 | 53809.68 | 53854.7 | 53806.13 | 53871.15 | 53880.18 | 53855.13 | 53838.7 | 53811.78 | 53850.13 | 53839.63 | 53873.5 | 53872.95 | 53847.47 | 53890.25 |
| 570.7554 | 53809.81 | 53851.1 | 53802.09 | 53872.38 | 53878.2 | 53850.35 | 53837.18 | 53809.75 | 53851.93 | 53837.55 | 53873.03 | 53868.7 | 53846.37 | 53890.06 |
| 570.8901 | 53812.46 | 53857.5 | 53806.09 | 53869.53 | 53882.38 | 53854.93 | 53837.5 | 53810.05 | 53852.25 | 53838.4 | 53870.4 | 53873.78 | 53841.5 | 53888.94 |
| 571.0248 | 53814.27 | 53855.78 | 53806.19 | 53875.8 | 53887.03 | 53856.5 | 53839.9 | 53813.2 | 53854.23 | 53838.08 | 53877.73 | 53867.08 | 53844.39 | 53889.25 |
| 571.1596 | 53819.32 | 53861.5 | 53812.78 | 53878.98 | 53887.45 | 53856.25 | 53843.23 | 53814.65 | 53855.83 | 53840.83 | 53875.18 | 53872.8 | 53843.08 | 53890.38 |
| 571.2943 | 53817.51 | 53862.13 | 53809.03 | 53875.45 | 53890.5 | 53859.9 | 53847.2 | 53811.13 | 53855.18 | 53836.55 | 53871.83 | 53868.38 | 53839.18 | 53884.63 |
| 571.4291 | 53824.14 | 53870.43 | 53818.59 | 53884.4 | 53896.93 | 53866.93 | 53857.13 | 53818.13 | 53861.55 | 53845.1 | 53880.25 | 53875.13 | 53844 | 53891.56 |
| 571.5638 | 53832.41 | 53877.33 | 53823.03 | 53892.15 | 53906.43 | 53873.8 | 53859.8 | 53822.68 | 53870.3 | 53849.53 | 53884.1 | 53877.35 | 53844.34 | 53891.75 |
| 571.6985 | 53843.05 | 53890.55 | 53836.34 | 53904.95 | 53920.48 | 53884.68 | 53871.7 | 53832.08 | 53879.03 | 53856.38 | 53886.98 | 53883.5 | 53844.76 | 53902 |
| 571.8332 | 53904.03 | 53961.13 | 53902.06 | 53973.85 | 54003.93 | 53951.68 | 53939.1 | 53877.65 | 53936.33 | 53894.83 | 53923.93 | 53910.4 | 53867.21 | 53918 |
| 571.968 | 54112 | 54184.53 | 54097.22 | 54203.48 | 54236.5 | 54158.68 | 54189.85 | 54022.63 | 54133.98 | 54001.95 | 54016.53 | 54010.18 | 53915.24 | 54000.56 |
| 572.1027 | 54220 | 54307.1 | 54270.13 | 54388.83 | 54442.25 | 54265.05 | 54331.45 | 54166.05 | 54214.5 | 54076.55 | 54099.8 | 54094.88 | 53953.58 | 54011.81 |
| 572.2374 | 54362.95 | 54385.38 | 54255.28 | 54483.63 | 54582.73 | 54435.45 | 54386.58 | 54247.23 | 54283.9 | 54221.18 | 54227.2 | 54111.75 | 53975.79 | 54029.38 |
| 572.3721 | 54377.81 | 54446.18 | 54291.53 | 54443.28 | 54681.15 | 54449.6 | 54398.5 | 54252.73 | 54306.08 | 54270.33 | 54186.18 | 54114.85 | 53956.39 | 54064.44 |
| 572.5068 | 54449.49 | 54423.9 | 54340.53 | 54490.98 | 54679.8 | 54476.48 | 54469.38 | 54251.08 | 54429.33 | 54266.68 | 54217.68 | 54160.8 | 53987.08 | 54196 |
| 572.6415 | 54416.7 | 54487.23 | 54352.19 | 54508.93 | 54664.53 | 54553.55 | 54459.05 | 54200.3 | 54504.35 | 54254.3 | 54204.35 | 54193.23 | 53980.29 | 54129.75 |
| 572.7762 | 54412.59 | 54527.08 | 54424.31 | 54478.4 | 54685.65 | 54562.9 | 54391.08 | 54256.65 | 54439.55 | 54270.6 | 54203.78 | 54229.25 | 53974.71 | 54135.19 |
| 572.9109 | 54438.54 | 54559.3 | 54409.88 | 54490 | 54651.88 | 54452.58 | 54481.83 | 54213.08 | 54407.5 | 54271.95 | 54305.35 | 54281.93 | 54009 | 54157.13 |
| 573.0456 | 54414.19 | 54551.05 | 54393.19 | 54529.93 | 54634.73 | 54507.98 | 54428.13 | 54265.6 | 54438.7 | 54284.63 | 54268.78 | 54278.43 | 54075.84 | 54218.13 |
| 573.1803 | 54387.32 | 54578.73 | 54436.66 | 54565.1 | 54581.25 | 54607.85 | 54511.93 | 54263.7 | 54412.15 | 54270.1 | 54215.85 | 54228.78 | 54091.29 | 54106.56 |
| 573.3149 | 54421.97 | 54537.7 | 54399.94 | 54570.58 | 54603.2 | 54569.93 | 54532.23 | 54292.7 | 54430.3 | 54311.65 | 54260.8 | 54217.85 | 54021.37 | 54077.56 |
| 573.4497 | 54360.68 | 54512.6 | 54367.97 | 54609.6 | 54551.3 | 54581.3 | 54449.28 | 54324 | 54475.55 | 54314.05 | 54201.45 | 54225.85 | 54007.05 | 54137.31 |
| 573.5844 | 54434.84 | 54558.35 | 54430.41 | 54562.55 | 54570.58 | 54555.33 | 54568.25 | 54313.75 | 54459.18 | 54344.88 | 54258.83 | 54217.2 | 54024.26 | 54082.06 |
| 573.719 | 54473.41 | 54604.25 | 54428.28 | 54542.13 | 54540.85 | 54472.55 | 54566.85 | 54294.93 | 54368.05 | 54269.53 | 54309.85 | 54188.4 | 54034.39 | 54100.19 |
| 573.8537 | 54366.41 | 54655.33 | 54431.94 | 54716 | 54600.2 | 54503.05 | 54492.38 | 54294.23 | 54410.65 | 54223.58 | 54235.63 | 54197.7 | 54024.03 | 54097.5 |
| 573.9883 | 54417.7 | 54619.38 | 54463 | 54686.4 | 54679.83 | 54529.7 | 54494 | 54267.6 | 54462.9 | 54274.6 | 54251.5 | 54303.65 | 54050.84 | 54106.75 |
| 574.1231 | 54423.95 | 54615.93 | 54381.88 | 54578.83 | 54607.98 | 54590.03 | 54566.88 | 54307.75 | 54446.73 | 54309.13 | 54293.38 | 54268.75 | 54104.92 | 54085.81 |
| 574.2577 | 54473.95 | 54575.43 | 54275.72 | 54541.28 | 54584.95 | 54520.9 | 54500.5 | 54336.2 | 54383.18 | 54329.7 | 54255.1 | 54287.55 | 54067.34 | 54106.5 |
| 574.3924 | 54491.59 | 54572.6 | 54369.13 | 54607.68 | 54670.78 | 54480.95 | 54490.25 | 54356.48 | 54375.88 | 54231.6 | 54251.88 | 54266.1 | 54054.55 | 54148.94 |
| 574.527 | 54537.59 | 54563.6 | 54439.03 | 54557.65 | 54761.5 | 54491.68 | 54509 | 54352.95 | 54417.95 | 54289.35 | 54290.98 | 54245.5 | 54058.24 | 54097.94 |
| 574.6617 | 54419.92 | 54644.23 | 54366.25 | 54483.7 | 54742.53 | 54523.13 | 54466.4 | 54331.85 | 54422.2 | 54296.68 | 54326 | 54259.95 | 54025.74 | 54089.69 |
| 574.7964 | 54432.14 | 54569.85 | 54343.63 | 54523.38 | 54662.1 | 54495.05 | 54486.55 | 54345.13 | 54420.85 | 54287.35 | 54261.73 | 54286.7 | 53995.92 | 54155.38 |
| 574.931 | 54417.59 | 54557.6 | 54453.56 | 54522.28 | 54635.95 | 54553.43 | 54466.9 | 54352.68 | 54336.25 | 54215.1 | 54292.53 | 54294.95 | 53994.92 | 54129.5 |
| 575.0657 | 54331.11 | 54593.75 | 54420.53 | 54561.83 | 54565.6 | 54483.23 | 54450.88 | 54324.8 | 54393.88 | 54240.48 | 54260.28 | 54237.35 | 54031.63 | 54153.69 |
| 575.2003 | 54374.95 | 54548.1 | 54372.84 | 54508.1 | 54617.25 | 54538.1 | 54425.15 | 54319.68 | 54407.63 | 54230.23 | 54290.88 | 54214.25 | 54046.55 | 54144.88 |
| 575.335 | 54385.76 | 54562.43 | 54417.06 | 54595.55 | 54539.28 | 54462.43 | 54487.78 | 54289.25 | 54402.6 | 54303.38 | 54256.03 | 54250.35 | 54075.87 | 54125.75 |
| 575.4696 | 54392.68 | 54561.55 | 54307.03 | 54524.55 | 54597.5 | 54423.33 | 54542.83 | 54302.2 | 54480.28 | 54271.95 | 54222.75 | 54298.23 | 54036.32 | 54174.88 |
| 575.6043 | 54408.68 | 54584.6 | 54415.19 | 54496.53 | 54770.95 | 54420.9 | 54521.83 | 54328.95 | 54522 | 54255.5 | 54257.33 | 54285.63 | 54052.24 | 54112.38 |
| 575.7389 | 54459.08 | 54551.78 | 54481.06 | 54415.05 | 54723.23 | 54402.1 | 54458.43 | 54270.55 | 54395.4 | 54255.15 | 54210.6 | 54321.3 | 54018.03 | 54059.69 |
| 575.8735 | 54423.08 | 54570.23 | 54422.72 | 54525.63 | 54624.08 | 54466.58 | 54517.95 | 54257.03 | 54388.73 | 54232.15 | 54151.85 | 54269.48 | 54021.05 | 54109.5 |
| 576.0082 | 54500.49 | 54471.78 | 54458.66 | 54510.7 | 54569 | 54478.88 | 54476.68 | 54218.85 | 54454.88 | 54237.28 | 54195 | 54265.45 | 54002.55 | 54124.25 |
| 576.1428 | 54419.97 | 54521.45 | 54455.28 | 54470.1 | 54570.75 | 54526.7 | 54383.55 | 54244.68 | 54397.95 | 54266.8 | 54177.8 | 54284.3 | 54001.37 | 54085.25 |
| 576.2774 | 54340.14 | 54474.75 | 54464.63 | 54539.58 | 54673.58 | 54455.2 | 54435.1 | 54250.68 | 54329 | 54254.75 | 54249.35 | 54286.98 | 54003.18 | 54057.75 |
| 576.4121 | 54311.49 | 54552.43 | 54463.94 | 54549.03 | 54543.1 | 54538.85 | 54499.9 | 54258.45 | 54342.63 | 54223.95 | 54271.93 | 54256.65 | 53981.97 | 54093.88 |
| 576.5466 | 54386.08 | 54565.43 | 54491.72 | 54522.58 | 54553.35 | 54472.9 | 54407.8 | 54216.75 | 54343.7 | 54279.95 | 54241.03 | 54169.28 | 54042.45 | 54141.94 |
| 576.6813 | 54329.38 | 54528.88 | 54440.69 | 54526.2 | 54530.85 | 54444.1 | 54490.25 | 54295.7 | 54386.65 | 54315.3 | 54208.05 | 54223.33 | 54016.79 | 54152.13 |
| 576.8159 | 54439.97 | 54556.23 | 54378.53 | 54515.13 | 54522.98 | 54491.95 | 54481.18 | 54300.95 | 54385.38 | 54309.9 | 54251.05 | 54247.1 | 54016.74 | 54191.38 |
| 576.9505 | 54359.41 | 54534.28 | 54352.88 | 54476.38 | 54512.25 | 54487.23 | 54428.8 | 54297.53 | 54425.13 | 54226.05 | 54210.9 | 54284.93 | 54050.42 | 54124.06 |
| 577.0851 | 54444.27 | 54488.18 | 54331.41 | 54444.78 | 54561.25 | 54477.75 | 54440.28 | 54311.48 | 54404.13 | 54209.73 | 54263 | 54300.95 | 54038.76 | 54082.19 |
| 577.2197 | 54500.65 | 54451.78 | 54358.69 | 54467.55 | 54599.25 | 54477 | 54453.13 | 54343.7 | 54404.03 | 54164.53 | 54263.35 | 54299.98 | 54038.87 | 54102.06 |
| 577.3543 | 54448.86 | 54484.75 | 54396.47 | 54471.23 | 54553.58 | 54495.65 | 54500.73 | 54242.08 | 54407.4 | 54237.23 | 54305.05 | 54312.65 | 54039.13 | 54238.56 |
| 577.4889 | 54381.38 | 54441.83 | 54338.28 | 54434.78 | 54506.98 | 54524.85 | 54458.85 | 54296.5 | 54327.38 | 54276.75 | 54346.13 | 54236.5 | 54058.18 | 54160.13 |
| 577.6235 | 54349.03 | 54420.28 | 54337.19 | 54491.43 | 54582.75 | 54516.58 | 54422.2 | 54235.03 | 54365.78 | 54262.05 | 54261.63 | 54213.28 | 54032.92 | 54139.06 |
| 577.7581 | 54399.38 | 54473.08 | 54392.25 | 54577.4 | 54616 | 54518.1 | 54403.13 | 54269.08 | 54385.53 | 54269.25 | 54221.85 | 54238.18 | 54035 | 54122.5 |
| 577.8927 | 54388.11 | 54535.55 | 54450.53 | 54511.15 | 54589.7 | 54405.2 | 54397.5 | 54192.38 | 54410.15 | 54247.93 | 54183.1 | 54246.73 | 54010.11 | 54133.88 |
| 578.0273 | 54367.22 | 54581.68 | 54441.22 | 54490.55 | 54627.33 | 54404.45 | 54438.23 | 54203 | 54455.25 | 54289.73 | 54209.48 | 54297.93 | 53989.16 | 54107.06 |
| 578.1619 | 54436.05 | 54587.65 | 54322.44 | 54572.85 | 54696.85 | 54495.33 | 54441.55 | 54261.05 | 54390.65 | 54351.38 | 54258.93 | 54231.95 | 53996.89 | 54090.38 |
| 578.2965 | 54455.24 | 54594.98 | 54321.63 | 54563.63 | 54741.98 | 54555.38 | 54482.78 | 54199.53 | 54386.18 | 54272.53 | 54280.53 | 54259.4 | 53993.5 | 54078.88 |
| 578.431 | 54433.05 | 54467.58 | 54320.34 | 54479.03 | 54562.05 | 54554.7 | 54474.15 | 54192.23 | 54346.98 | 54240.45 | 54283.43 | 54265.28 | 54011.24 | 54082.31 |
| 578.5656 | 54376.32 | 54557.08 | 54334.75 | 54471.58 | 54562.85 | 54507.15 | 54443.83 | 54228.6 | 54361.18 | 54268.68 | 54224.95 | 54236.65 | 54038.89 | 54249.13 |
| 578.7002 | 54366.97 | 54473.85 | 54323.53 | 54490.75 | 54584.65 | 54464.45 | 54393.43 | 54235.48 | 54337.65 | 54267.85 | 54239.9 | 54210.78 | 54032.61 | 54207.13 |
| 578.8348 | 54321.41 | 54514.1 | 54344.22 | 54452.8 | 54544.73 | 54476.7 | 54463 | 54259.45 | 54379.95 | 54244.1 | 54274.95 | 54256.43 | 53997.03 | 54158.81 |
| 578.9693 | 54339.57 | 54461.68 | 54256.06 | 54492.65 | 54538.83 | 54495.93 | 54491.35 | 54243.6 | 54432.03 | 54264.08 | 54273.93 | 54290.93 | 53998.45 | 54106.13 |
| 579.1039 | 54400.78 | 54509.18 | 54283.03 | 54478.13 | 54610 | 54556.8 | 54487.48 | 54218.4 | 54383.23 | 54301.08 | 54306.53 | 54255.7 | 54049.87 | 54188.94 |
| 579.2385 | 54412.51 | 54547.05 | 54289.94 | 54511.55 | 54572 | 54587.2 | 54416.18 | 54205.55 | 54440.18 | 54273.28 | 54305.53 | 54279.3 | 54037 | 54129 |
| 579.373 | 54415.11 | 54480.1 | 54351.47 | 54573.75 | 54619.73 | 54514.85 | 54433.18 | 54245.2 | 54492.15 | 54229.85 | 54253.35 | 54322.55 | 54018.03 | 54139.25 |
| 579.5076 | 54336.92 | 54486.4 | 54363.44 | 54580.6 | 54586.5 | 54538.55 | 54482.75 | 54302.1 | 54510.83 | 54283.95 | 54247.8 | 54292.33 | 54009.47 | 54148.19 |
| 579.6421 | 54322 | 54563.8 | 54363.97 | 54496.73 | 54520.38 | 54470.2 | 54543.03 | 54214.88 | 54428.4 | 54329.3 | 54331.1 | 54273 | 54031.82 | 54156.69 |
| 579.7767 | 54394.92 | 54561.95 | 54406.06 | 54552.8 | 54608.53 | 54435.85 | 54497.93 | 54263.18 | 54447.9 | 54306.5 | 54315.08 | 54281.9 | 54036.42 | 54148 |
| 579.9112 | 54444.11 | 54595.85 | 54481.22 | 54608.23 | 54710.35 | 54433.83 | 54548.58 | 54292.98 | 54416.48 | 54289.53 | 54281.58 | 54247.38 | 54042.34 | 54154.06 |
| 580.0458 | 54395.84 | 54597.53 | 54502.5 | 54499.38 | 54655.03 | 54498 | 54633.8 | 54277.13 | 54404.73 | 54242.28 | 54328.55 | 54331.95 | 53986.29 | 54109.5 |
| 580.1803 | 54378.46 | 54640.23 | 54482.34 | 54548.88 | 54618.03 | 54566.25 | 54604.98 | 54302.18 | 54355.95 | 54237.55 | 54279.68 | 54310.88 | 53990.71 | 54171.88 |
| 580.3148 | 54473.95 | 54607.48 | 54444.81 | 54551.85 | 54683.35 | 54547.03 | 54563.28 | 54271.35 | 54420.6 | 54279.68 | 54309.03 | 54300.18 | 54044.74 | 54161.5 |
| 580.4493 | 54488.81 | 54570.98 | 54440.84 | 54542.4 | 54669.28 | 54551.03 | 54473.13 | 54306.08 | 54415.8 | 54331.85 | 54408.08 | 54367.25 | 54079.79 | 54182.19 |
| 580.5839 | 54518.08 | 54623.25 | 54489.75 | 54562.23 | 54586.6 | 54608.88 | 54432.13 | 54289.45 | 54440.63 | 54356.3 | 54305.93 | 54284 | 54103.42 | 54168.75 |
| 580.7184 | 54459.05 | 54617.23 | 54390.5 | 54502.53 | 54615.68 | 54585.7 | 54501.93 | 54292.53 | 54370.38 | 54360.85 | 54278.38 | 54304.23 | 54077.26 | 54278.5 |
| 580.853 | 54360.65 | 54630.55 | 54468.25 | 54514.33 | 54647.13 | 54562.53 | 54571.88 | 54366.38 | 54392.1 | 54381.25 | 54344.85 | 54264.3 | 54075.45 | 54244.13 |
| 580.9875 | 54430.78 | 54664.93 | 54504.69 | 54490.9 | 54625.58 | 54599.15 | 54580.95 | 54304.93 | 54509.18 | 54258.85 | 54346.9 | 54309.53 | 54075.37 | 54212.94 |
| 581.122 | 54424.81 | 54634.58 | 54442.53 | 54556.3 | 54651.53 | 54628.23 | 54559.33 | 54274.8 | 54531.58 | 54211.53 | 54315.43 | 54285.13 | 54103.95 | 54191.5 |
| 581.2565 | 54476.14 | 54629.93 | 54399.06 | 54610.13 | 54659.23 | 54607.75 | 54556.53 | 54319.3 | 54455.03 | 54236.9 | 54385.43 | 54327.35 | 54100.42 | 54116.81 |
| 581.391 | 54459.03 | 54539.1 | 54455.81 | 54612.85 | 54676.33 | 54571.08 | 54498.25 | 54329.25 | 54447.55 | 54293.25 | 54378.1 | 54349.8 | 54080.71 | 54191.63 |
| 581.5255 | 54514.51 | 54559.4 | 54493.81 | 54599.48 | 54713.75 | 54577.63 | 54540.5 | 54307.95 | 54440.43 | 54303.15 | 54315.45 | 54309.18 | 54101.87 | 54145.5 |
| 581.66 | 54533 | 54598.43 | 54587.63 | 54664.23 | 54633.68 | 54684.38 | 54664.58 | 54339.68 | 54438.18 | 54325.4 | 54322.73 | 54361.1 | 54060.76 | 54199.81 |
| 581.7946 | 54446.11 | 54582.5 | 54542.97 | 54683.48 | 54608.68 | 54616.45 | 54605.1 | 54362.28 | 54407.48 | 54287.2 | 54338.1 | 54324.25 | 54060.89 | 54148.13 |
| 581.929 | 54528.11 | 54550.23 | 54532.25 | 54714.48 | 54690.03 | 54690.25 | 54572.33 | 54383.5 | 54404.73 | 54330.15 | 54406.98 | 54301.4 | 54028.47 | 54253.88 |
| 582.0635 | 54557.38 | 54543.35 | 54516.69 | 54589.7 | 54778.25 | 54585.65 | 54525.93 | 54298.33 | 54462.83 | 54297.05 | 54404.68 | 54284.2 | 54064.42 | 54281.38 |
| 582.1981 | 54625.78 | 54571.18 | 54505.28 | 54677.45 | 54698.7 | 54573.68 | 54597.28 | 54334.15 | 54489.53 | 54363 | 54440.33 | 54392.8 | 54084.76 | 54183.25 |
| 582.3325 | 54648.41 | 54603.65 | 54484 | 54728.13 | 54665.83 | 54683.9 | 54615.43 | 54349.7 | 54507.2 | 54442.25 | 54337.43 | 54370.88 | 54118.66 | 54167.44 |
| 582.467 | 54539.03 | 54667.78 | 54534.94 | 54640.8 | 54846.8 | 54639.13 | 54664.03 | 54382.6 | 54523.18 | 54446.1 | 54343.53 | 54348.65 | 54056.45 | 54227.69 |
| 582.6015 | 54605.05 | 54697.2 | 54493.75 | 54662.6 | 54921.25 | 54619.98 | 54731.1 | 54399.33 | 54594.88 | 54408.53 | 54367.48 | 54364.03 | 54075.66 | 54261.44 |
| 582.736 | 54587.3 | 54642.83 | 54521.66 | 54756.48 | 54889.85 | 54574.83 | 54719.63 | 54472.58 | 54649.58 | 54362.58 | 54431.78 | 54367.23 | 54065.95 | 54202.63 |
| 582.8705 | 54608 | 54662.25 | 54580.28 | 54725.53 | 54935.7 | 54545.43 | 54686.95 | 54493.85 | 54629 | 54335.68 | 54453.75 | 54343.73 | 54061.84 | 54196.13 |
| 583.0049 | 54628.95 | 54653.88 | 54602.34 | 54781.85 | 54781.85 | 54635.15 | 54646.18 | 54512.23 | 54556.75 | 54400.38 | 54441.55 | 54348.28 | 54054.66 | 54220.25 |
| 583.1394 | 54649.08 | 54748.8 | 54426.25 | 54814.73 | 54756 | 54715.83 | 54662.35 | 54516.03 | 54612.3 | 54490.78 | 54534.53 | 54311.4 | 54110.05 | 54304 |
| 583.2739 | 54633.14 | 54749.5 | 54491.63 | 54804.7 | 54816.93 | 54776.13 | 54714.98 | 54476 | 54635.4 | 54448.13 | 54516.55 | 54439.1 | 54112.58 | 54263.88 |
| 583.4084 | 54638.97 | 54807.45 | 54511.06 | 54787.58 | 54900.05 | 54728.48 | 54735.98 | 54466.58 | 54650.38 | 54439.35 | 54486.05 | 54428.68 | 54078.05 | 54251.13 |
| 583.5429 | 54650.32 | 54726.68 | 54638.56 | 54826.8 | 54933.78 | 54745.08 | 54781 | 54510 | 54578.93 | 54520 | 54482.78 | 54413.8 | 54054.71 | 54296 |
| 583.6773 | 54635.7 | 54754.75 | 54658.16 | 54890.85 | 54809.55 | 54758.03 | 54748.85 | 54430.2 | 54616.55 | 54498.25 | 54438.18 | 54455.6 | 54071.55 | 54234.75 |
| 583.8118 | 54764.92 | 54899.35 | 54701.06 | 54886.2 | 54885.63 | 54794.28 | 54807.25 | 54459.38 | 54788.93 | 54488.43 | 54474.1 | 54491.95 | 54090.34 | 54208.63 |
| 583.9462 | 54760.76 | 54818.95 | 54627.47 | 54836.65 | 54979.03 | 54809.4 | 54799.43 | 54473.33 | 54690.05 | 54486.75 | 54515.73 | 54561.85 | 54105.21 | 54316.88 |
| 584.0806 | 54764.22 | 54894.9 | 54710 | 54978.68 | 55085 | 54764.05 | 54843.4 | 54492.65 | 54693 | 54551.98 | 54511.33 | 54563.55 | 54144.11 | 54324.25 |
| 584.2151 | 54810.62 | 54907.7 | 54842.19 | 54931.85 | 55100.53 | 54831.33 | 54853.03 | 54583.38 | 54723.98 | 54518.25 | 54552.3 | 54648.93 | 54126.42 | 54285.88 |
| 584.3496 | 54913.05 | 54928.63 | 54835.72 | 54964.58 | 55099.73 | 54950.48 | 54837.93 | 54659.68 | 54776.25 | 54571.5 | 54565.2 | 54529.78 | 54165.63 | 54423.94 |
| 584.484 | 55036.38 | 55060.63 | 54865.81 | 55177.33 | 55162.7 | 54990.9 | 54842.9 | 54658.78 | 54910.45 | 54646.28 | 54573.98 | 54600.95 | 54165.26 | 54466.88 |
| 584.6184 | 55104.38 | 55081.85 | 54892.19 | 55272.75 | 55134.55 | 55060.03 | 54891.35 | 54711.18 | 54975.53 | 54717.95 | 54668.83 | 54697.65 | 54212.58 | 54417.5 |
| 584.7529 | 55174.62 | 55105.4 | 54962.78 | 55325.3 | 55228.5 | 55095.13 | 54993.28 | 54775.18 | 55008.03 | 54749.28 | 54752.35 | 54725.23 | 54206.58 | 54403.56 |
| 584.8873 | 55170.38 | 55104.05 | 55008.72 | 55409.1 | 55257.75 | 55162 | 55049.2 | 54858.85 | 55208.05 | 54756.03 | 54790.18 | 54701.7 | 54186.47 | 54450 |
| 585.0217 | 55378.41 | 55193.5 | 55124.09 | 55429.78 | 55466.48 | 55308.88 | 55190.38 | 54894.63 | 55166.78 | 54863.9 | 54883.08 | 54747.25 | 54213.71 | 54494 |
| 585.1561 | 55438.41 | 55290.1 | 55190.31 | 55601.38 | 55669.93 | 55354.73 | 55379.65 | 54971.9 | 55313.83 | 54970.4 | 55064.35 | 54906.45 | 54280.21 | 54553.44 |
| 585.2906 | 55559.73 | 55363.93 | 55263.63 | 55693.05 | 55735.75 | 55508.9 | 55446.98 | 55059.35 | 55381.03 | 55109.75 | 55027.38 | 54959.65 | 54320.13 | 54565.88 |
| 585.425 | 55638.7 | 55523.1 | 55417.19 | 55858.25 | 55802.95 | 55772.18 | 55575.38 | 55083.73 | 55456.53 | 55223.2 | 55120.53 | 54982.18 | 54303.03 | 54661.44 |
| 585.5594 | 55852.78 | 55781.68 | 55634.59 | 56051.05 | 55930.5 | 55764.6 | 55622.6 | 55172.08 | 55593.23 | 55228.8 | 55203.35 | 55112.55 | 54355.87 | 54759.19 |
| 585.6939 | 56036.95 | 55767.53 | 55574.91 | 56189.53 | 56064.4 | 55722.35 | 55699.23 | 55369.73 | 55609.8 | 55317.98 | 55310.13 | 55280.98 | 54440.89 | 54854.63 |
| 585.8283 | 56073.76 | 55956.65 | 55753.81 | 56165.18 | 56194.83 | 55959.95 | 55807 | 55401.78 | 55874.88 | 55335.58 | 55299.55 | 55312.73 | 54501.39 | 54833.38 |
| 585.9627 | 56100.76 | 56079.85 | 55893.5 | 56329.58 | 56202.1 | 56030.4 | 55891.08 | 55383.73 | 56090.05 | 55373.08 | 55380.03 | 55325.45 | 54534.47 | 54878.94 |
| 586.0971 | 56289.62 | 56127.08 | 56038.5 | 56395.18 | 56455.23 | 56197.03 | 56045.58 | 55517.43 | 56080.3 | 55507.78 | 55513.55 | 55371.05 | 54559.5 | 54962.5 |
| 586.2315 | 56266.27 | 56239.93 | 56084.53 | 56562.45 | 56602.08 | 56268.45 | 56194.65 | 55693.33 | 56104.95 | 55602.8 | 55613.3 | 55393.28 | 54589.63 | 55035.13 |
| 586.3658 | 56295.16 | 56335.78 | 56191.16 | 56609.13 | 56629.65 | 56397.5 | 56216.75 | 55759.8 | 56061.18 | 55750.98 | 55702.48 | 55554.18 | 54638.79 | 55082.94 |
| 586.5002 | 56533.84 | 56491.3 | 56322.38 | 56773.93 | 56783.23 | 56468.3 | 56260.18 | 55822.43 | 56339.43 | 55787.8 | 55726.53 | 55653.65 | 54652.45 | 55004.63 |
| 586.6346 | 56641.86 | 56586.45 | 56496.09 | 56900.93 | 56954.45 | 56520.05 | 56416.15 | 55910.73 | 56571.55 | 55842.33 | 55859.03 | 55661.3 | 54673.26 | 55131.5 |
| 586.769 | 56804.95 | 56695.03 | 56500.63 | 57090.78 | 57138.88 | 56757.5 | 56586.85 | 55944.08 | 56633.8 | 55944.58 | 55969.78 | 55823.5 | 54696.26 | 55139.31 |
| 586.9034 | 57010.35 | 56954.23 | 56751.91 | 57290.2 | 57177.25 | 56966.18 | 56685.88 | 56088.7 | 56692.18 | 56099.15 | 56031.05 | 55940.08 | 54790.61 | 55167.44 |
| 587.0378 | 57104.57 | 57078.83 | 56934.59 | 57460.65 | 57454.03 | 57041.48 | 56909.45 | 56258 | 56822.33 | 56143.88 | 56164.68 | 56063.85 | 54828.84 | 55383.5 |
| 587.1722 | 57378.76 | 57316.18 | 57184.41 | 57607.05 | 57568.58 | 57274.95 | 57045.23 | 56252.3 | 56995.7 | 56265.58 | 56333.58 | 56174.85 | 54897.16 | 55455.25 |
| 587.3066 | 57683.51 | 57556.15 | 57469.69 | 57914.83 | 57968.28 | 57554.68 | 57232.28 | 56490.65 | 57169.4 | 56388.83 | 56508.05 | 56330.35 | 54904.55 | 55583.56 |
| 587.4409 | 57967.43 | 57715.48 | 57695.19 | 58294.08 | 58331.2 | 57719.13 | 57594.35 | 56709 | 57423.33 | 56633.53 | 56618.4 | 56446.78 | 54988.03 | 55668.5 |
| 587.5753 | 58286.62 | 58011.45 | 57921.88 | 58672 | 58482.88 | 57949.58 | 57820.85 | 57121.15 | 57782.58 | 56824.83 | 56814.05 | 56690.18 | 55074.37 | 55812.56 |
| 587.7097 | 58680.49 | 58238.83 | 58328.78 | 59031.8 | 58758.6 | 58190.5 | 57978.08 | 57203.1 | 58109.55 | 57124 | 57026.55 | 56950.33 | 55117.39 | 55862.75 |
| 587.8441 | 59268.54 | 58568.03 | 58438.94 | 59653.3 | 59205.2 | 58807.75 | 58453.53 | 57572.63 | 58604.55 | 57318.15 | 57517 | 57256.98 | 55315.71 | 56160.56 |
| 587.9784 | 59755.24 | 59102.88 | 58826 | 60264.23 | 59662.7 | 59112.05 | 58852.23 | 58015.48 | 59111.58 | 57701.23 | 57908.25 | 57635.85 | 55470.37 | 56492.25 |
| 588.1127 | 60611.65 | 59619.1 | 59526 | 61194.93 | 60255.75 | 59532.05 | 59410.45 | 58356.68 | 59667.23 | 58353.35 | 58374.2 | 58082.08 | 55677.89 | 57114.69 |
| 588.2471 | 61820.89 | 60354.25 | 60196.28 | 62708.93 | 61202.9 | 60348.7 | 60075.73 | 59053.98 | 60669.7 | 58824.48 | 59078.18 | 58680.38 | 55858.32 | 57464.13 |
| 588.3815 | 63530.78 | 61208.13 | 61116.97 | 64265.7 | 62283.53 | 61271.63 | 60877.05 | 59965.25 | 62094.6 | 59799.78 | 60075.98 | 59377.75 | 56202.63 | 58334.88 |
| 588.5158 | 65746.84 | 62370.73 | 62619.41 | 66560.03 | 63619.5 | 62525.58 | 61815.63 | 61189.1 | 63875.1 | 60853.93 | 61522.58 | 60411.68 | 56707.66 | 59252.75 |
| 588.6502 | 68627.49 | 64197.28 | 64594.31 | 69905.85 | 65384.05 | 64655.53 | 63552.75 | 62899.63 | 66523.6 | 62414.25 | 63316 | 62192.93 | 57281.08 | 61097.31 |
| 588.7845 | 73138.59 | 66659.98 | 67050.59 | 74992.9 | 68098.38 | 67415.53 | 65481.95 | 65231.78 | 70045.23 | 64339.88 | 65976.45 | 64058.45 | 58254.63 | 63350 |
| 588.9188 | 80901 | 70097.68 | 71301.38 | 83731.1 | 71810.25 | 71144.88 | 68967.73 | 68635.2 | 75650.48 | 67438.5 | 69944.75 | 67132.1 | 59738.76 | 67932.13 |
| 589.0532 | 96407.97 | 74844.58 | 77700 | 99535.33 | 77690.9 | 76972.8 | 73959.43 | 74841.83 | 86039.8 | 72259 | 77460.33 | 71808.3 | 61650.24 | 76180.88 |
| 589.1875 | 123705.8 | 84081.33 | 89372.03 | 129466.3 | 87689.1 | 87000.85 | 82001.93 | 86159.03 | 104829.5 | 80827.85 | 91598.88 | 80337.38 | 65620.58 | 90644.06 |
| 589.3218 | 168232.2 | 99587.05 | 110697.8 | 176460.5 | 104880.2 | 106339.6 | 96924.78 | 106660.4 | 138608.2 | 96423.9 | 115602.5 | 96894.4 | 73188.29 | 108994 |
| 589.4562 | 214997.3 | 127920.1 | 146346.5 | 227021.8 | 135030.5 | 139592.1 | 122329.1 | 138624.8 | 179289.7 | 123670.8 | 148763.7 | 124190 | 84767 | 126699.3 |
| 589.5905 | 241391.5 | 169593.7 | 188218.8 | 254190.3 | 184942.6 | 184157.3 | 160887.7 | 169734 | 211263 | 156857.9 | 174908.9 | 156896.8 | 97965.55 | 131886.9 |
| 589.7248 | 227230.3 | 219682.2 | 225599.1 | 239675 | 243859.8 | 229425.1 | 206164.4 | 194015.1 | 219368.3 | 185269.8 | 181367 | 183353.2 | 107440.7 | 121519.1 |
| 589.8591 | 188132 | 254390.2 | 234936.9 | 197355.6 | 280910.3 | 251566.3 | 236546.1 | 189102.1 | 191440.4 | 190901.6 | 160261.8 | 179997.8 | 104818.2 | 107869.9 |
| 589.9934 | 168616.8 | 248358.3 | 205935.2 | 179113 | 271597.8 | 232978.5 | 237238.3 | 165146 | 166302.2 | 170367.3 | 141328.5 | 157059.4 | 96001.95 | 103866.9 |
| 590.1278 | 168097.1 | 206743.9 | 173352.3 | 177292.4 | 220109.1 | 191992.2 | 198874.8 | 145313.1 | 156990.1 | 146867.2 | 134325.8 | 138165.2 | 88973.03 | 101621.9 |
| 590.262 | 159621.4 | 181390.3 | 165731.7 | 167410.9 | 194103.5 | 175822.3 | 172820.4 | 142395.4 | 153691.3 | 140423.9 | 130507.8 | 135137.8 | 87889.92 | 94701.75 |
| 590.3964 | 128533.1 | 178623.8 | 162495.3 | 134834.3 | 193082.7 | 174655.8 | 170040.4 | 136210.8 | 135480.2 | 137525.3 | 117243 | 132113.1 | 86145.66 | 82388.69 |
| 590.5306 | 97646.16 | 170084.1 | 143200.5 | 101722.9 | 184324.7 | 161362.6 | 163645.2 | 116471 | 106695.3 | 123050.6 | 96760.13 | 114600.8 | 78579.11 | 71005.81 |
| 590.6649 | 80418.68 | 140130.5 | 110837.5 | 83235.3 | 149224 | 127392.4 | 136519.4 | 92047.9 | 85930.75 | 98838.93 | 80303.38 | 91171.53 | 68930.29 | 65111.5 |
| 590.7993 | 72705.16 | 106577.1 | 88000.69 | 74993 | 111039.5 | 97305.15 | 104604.8 | 77696.13 | 75529.75 | 81906.6 | 71905.85 | 76940.98 | 62918.87 | 62247.75 |
| 590.9335 | 68818.11 | 86266.63 | 76556.31 | 70213.93 | 88178.35 | 82081.7 | 84703.65 | 69859.68 | 69982.98 | 72556.7 | 66700.18 | 69648.43 | 60160.97 | 60426.38 |
| 591.0678 | 65728.16 | 76442.2 | 71067.72 | 66805.9 | 77513.08 | 73702.98 | 75216.08 | 65681.35 | 66585.43 | 67924.18 | 63828.83 | 65944.93 | 58832.39 | 59097.81 |
| 591.2021 | 63662.35 | 71139.53 | 67447 | 64749.58 | 72297.35 | 69174.6 | 69895.93 | 63740.05 | 64162.58 | 64679.55 | 61923.58 | 63609.23 | 57727.55 | 58289 |
| 591.3364 | 61789.54 | 67688.25 | 64655.66 | 62887 | 68944.23 | 66037.63 | 66537.03 | 61826.73 | 62461.63 | 62593.85 | 60481.28 | 61758.75 | 56983.53 | 57351.88 |
| 591.4706 | 60602.84 | 65230.13 | 62822.19 | 61366.55 | 66200.68 | 63972.28 | 64071.9 | 60432.68 | 60996.35 | 60948.1 | 59474.68 | 60213.48 | 56678.63 | 56794 |
| 591.6049 | 59893.08 | 63084.88 | 61272.25 | 60460.05 | 64037.93 | 62439.4 | 62633.53 | 59457.7 | 59940.5 | 59842.3 | 58712.08 | 59109.2 | 56149.71 | 56469 |
| 591.7392 | 59300.95 | 61840.75 | 60303.06 | 59677.38 | 62388.78 | 61380.15 | 61181.05 | 58627.63 | 59199.45 | 59052.1 | 58095.78 | 58624.9 | 55801.18 | 56112.63 |
| 591.8735 | 58666.03 | 60686.68 | 59370.22 | 59190.23 | 61260.33 | 60364.2 | 60231.03 | 58075.8 | 58518.7 | 58299.73 | 57632.2 | 58075.43 | 55531.66 | 55957.56 |
| 592.0078 | 58092.95 | 60046.35 | 58971 | 58704.83 | 60402.03 | 59666.83 | 59511.03 | 57757.48 | 58144.6 | 57857.08 | 57170.23 | 57579.68 | 55311.42 | 55896.94 |
| 592.142 | 57783.16 | 59405.25 | 58410.66 | 58330.6 | 59719.98 | 59066.9 | 58892.58 | 57332.83 | 57910.38 | 57526.15 | 56984.55 | 57257.5 | 55125.03 | 55703.06 |
| 592.2763 | 57446.24 | 58780.48 | 58084.53 | 57959.2 | 59382.28 | 58505.35 | 58555.53 | 57000.98 | 57479.4 | 57186.2 | 56726.08 | 56934.13 | 55067.42 | 55569.69 |
| 592.4105 | 57160.57 | 58213.9 | 57776.06 | 57712.6 | 58916.78 | 58258.6 | 58201.33 | 56680.15 | 57104.83 | 56927.83 | 56581.85 | 56705.45 | 55022.39 | 55474.69 |
| 592.5447 | 56925.54 | 57902.3 | 57457.22 | 57458.68 | 58325.75 | 57852.13 | 57757.65 | 56499.23 | 56890.05 | 56662.5 | 56158.28 | 56505.33 | 55025.66 | 55346.25 |
| 592.679 | 56785.86 | 57783.98 | 57081 | 57106.1 | 58042.18 | 57508.78 | 57428.1 | 56326.9 | 56749.88 | 56519.43 | 56017.83 | 56346.83 | 54952.97 | 55125.81 |
| 592.8132 | 56635.84 | 57492.48 | 56953.88 | 56971.43 | 57794.23 | 57309.58 | 57350.63 | 56141.85 | 56575.15 | 56375.8 | 55937.23 | 56279.1 | 54794.11 | 54987.13 |
| 592.9475 | 56562.24 | 57335.95 | 56834 | 56844.83 | 57447.1 | 56947.18 | 57058.45 | 56003.78 | 56445.25 | 56219.88 | 55729.8 | 56106.55 | 54778.61 | 54982 |
| 593.0817 | 56434.65 | 57073.53 | 56577.22 | 56681.28 | 57304.35 | 56778.63 | 56795.55 | 55963.88 | 56293.38 | 56002.73 | 55603.55 | 55839.3 | 54669.21 | 54897.25 |
| 593.2159 | 56236.78 | 56870.38 | 56332.75 | 56575.05 | 57152.58 | 56728 | 56614.3 | 55861.38 | 56107.53 | 55890.85 | 55504.95 | 55699.35 | 54606.82 | 54781.13 |
| 593.3502 | 56117.62 | 56679.93 | 56249.97 | 56388.78 | 56997.93 | 56558.4 | 56493.83 | 55705.08 | 55983.05 | 55769.73 | 55526.48 | 55568.88 | 54549.97 | 54863.13 |
| 593.4844 | 56212.76 | 56622.38 | 56250.44 | 56382.9 | 56771.85 | 56539.48 | 56424.9 | 55672 | 55860.93 | 55698.65 | 55455.68 | 55557.6 | 54521.18 | 54754.19 |
| 593.6187 | 55989.38 | 56452.25 | 56077.09 | 56136.28 | 56713.48 | 56471.98 | 56395.48 | 55611.03 | 55721.58 | 55605.3 | 55423.58 | 55459.58 | 54511.58 | 54748.88 |
| 593.7529 | 55817.78 | 56250.8 | 56000.06 | 56105.13 | 56495.7 | 56182.25 | 56193 | 55540.08 | 55646.25 | 55566.78 | 55315.23 | 55367.15 | 54477.32 | 54757.19 |
| 593.8871 | 55688.84 | 56173.5 | 55910.34 | 55971.2 | 56422.95 | 56058.05 | 56104.25 | 55424.25 | 55689 | 55432.48 | 55251.78 | 55316.78 | 54435.53 | 54709.94 |
| 594.0213 | 55596.76 | 55993.63 | 55779.38 | 55874.45 | 56276.48 | 55928.5 | 55876.23 | 55336.95 | 55636.05 | 55351.88 | 55118.63 | 55213.5 | 54397.11 | 54583.56 |
| 594.1555 | 55570.08 | 55854.48 | 55657.38 | 55746.13 | 56169.35 | 55800.18 | 55798.23 | 55323.4 | 55521.08 | 55331.7 | 55094.13 | 55180.73 | 54369.55 | 54530.81 |
| 594.2897 | 55436.84 | 55837 | 55576.34 | 55630.03 | 56191.15 | 55814.43 | 55852 | 55235.05 | 55420.65 | 55249.43 | 55058.93 | 55186.28 | 54332.63 | 54498.94 |
| 594.4239 | 55385.14 | 55829.45 | 55494.5 | 55570.2 | 56126.3 | 55810.35 | 55650.13 | 55266.43 | 55349.6 | 55147.08 | 54997.23 | 54994.33 | 54309.92 | 54475.31 |
| 594.5581 | 55297.68 | 55623.58 | 55386.56 | 55459.8 | 56132.08 | 55564.63 | 55593 | 55188.93 | 55368.3 | 55052.35 | 54936.18 | 54943 | 54302 | 54422.38 |
| 594.6923 | 55195.08 | 55524.23 | 55336.13 | 55422.63 | 55953.05 | 55532.9 | 55454.7 | 55030.98 | 55238.73 | 55042.18 | 54859.68 | 54962.48 | 54300.18 | 54344.81 |
| 594.8265 | 55112.49 | 55585.85 | 55242.13 | 55433.73 | 55750.28 | 55483.55 | 55482.55 | 54979.98 | 55188.3 | 54965.65 | 54879.35 | 54896.8 | 54315.37 | 54368.31 |
| 594.9607 | 55054.49 | 55451.9 | 55229.66 | 55313.43 | 55686.95 | 55394.1 | 55426.08 | 54940.6 | 55199.8 | 55031.23 | 54842.28 | 54820.85 | 54318.95 | 54328.31 |
| 595.0949 | 55041.24 | 55354.15 | 55127.63 | 55263.4 | 55532.2 | 55209.6 | 55290.08 | 54968.55 | 54961.95 | 54901.6 | 54816.95 | 54792.73 | 54224.66 | 54304.06 |
| 595.2291 | 54983.41 | 55230.75 | 55131.88 | 55121.78 | 55439.38 | 55252.95 | 55195.48 | 54861.2 | 54990.13 | 54823.6 | 54711.38 | 54720.2 | 54237.61 | 54328.31 |
| 595.3632 | 55053.95 | 55106.75 | 55064.22 | 55070.1 | 55409.85 | 55189.2 | 55245.9 | 54776.08 | 54978.63 | 54765.48 | 54742.35 | 54634.38 | 54203.42 | 54249.5 |
| 595.4974 | 55008.62 | 55097.38 | 54971.22 | 55059.1 | 55369.53 | 55182.1 | 55175.08 | 54701.3 | 54931.25 | 54643.9 | 54621.95 | 54634.6 | 54205.16 | 54207.81 |
| 595.6316 | 54927.81 | 55103.73 | 54946.59 | 54968.28 | 55421.4 | 55201.6 | 55121.2 | 54666.75 | 54778.58 | 54661.25 | 54537.98 | 54565 | 54226.18 | 54191.63 |
| 595.7658 | 54952.65 | 55105.28 | 54932.03 | 55034.43 | 55232.2 | 55158.13 | 55024.9 | 54686.98 | 54770.55 | 54681.5 | 54467.03 | 54638.75 | 54188.53 | 54221.44 |
| 595.9 | 54873.19 | 55114.9 | 54837.19 | 55019.43 | 55286.1 | 55083.9 | 54966.6 | 54722.9 | 54781.28 | 54650.8 | 54481.48 | 54643.68 | 54185.26 | 54279.88 |
| 596.0341 | 54969.73 | 55074.03 | 54767.94 | 54947.63 | 55290.85 | 54975.95 | 55034.98 | 54761.1 | 54736.85 | 54693.73 | 54547.4 | 54557.95 | 54134.45 | 54310.88 |
| 596.1683 | 54896.14 | 54955.63 | 54889.81 | 54930.88 | 55215.63 | 54956.43 | 55016.93 | 54669.75 | 54756.48 | 54666.98 | 54534 | 54530.2 | 54124.84 | 54367.5 |
| 596.3024 | 54848.86 | 54894.48 | 54897.28 | 55002.25 | 55222.28 | 55023.23 | 54934.8 | 54638.45 | 54860.45 | 54618.93 | 54488.15 | 54581.28 | 54114.58 | 54309.88 |
| 596.4366 | 54864.76 | 54954.4 | 54855.56 | 55137.05 | 55195.68 | 54972.38 | 54946.75 | 54701.35 | 54848.38 | 54560.45 | 54579.23 | 54518.78 | 54200.79 | 54226.31 |
| 596.5707 | 54835.3 | 54967.1 | 54791.88 | 54983.38 | 55180.33 | 55026.85 | 54938.6 | 54619.68 | 54823.88 | 54578.8 | 54573.5 | 54607.15 | 54141.61 | 54268.31 |
| 596.7049 | 54880.19 | 54906.78 | 54766.94 | 54910 | 55121.1 | 54963.6 | 54996.65 | 54645.68 | 54826.73 | 54600.98 | 54466.5 | 54587.4 | 54149.47 | 54283.19 |
| 596.839 | 54863.62 | 54863.63 | 54814.53 | 54823.68 | 55108.18 | 55008.5 | 54958.28 | 54640.5 | 54844.8 | 54659.25 | 54534.43 | 54552.5 | 54129.13 | 54302.38 |
| 596.9731 | 54921.41 | 54855.8 | 54735.72 | 54861.4 | 55096.9 | 55004.1 | 54920.88 | 54551.9 | 54754.33 | 54619.35 | 54548.38 | 54611.18 | 54180.74 | 54299.56 |
| 597.1073 | 54904.84 | 54892.63 | 54759.81 | 54911.08 | 55079.68 | 54965.85 | 54942.73 | 54556.3 | 54730.28 | 54645.48 | 54610.1 | 54571.28 | 54191.45 | 54294.44 |
| 597.2414 | 54847.78 | 54966 | 54745.84 | 54985.83 | 55087.05 | 54931.2 | 55020.58 | 54608.38 | 54729.35 | 54643.88 | 54624.98 | 54468.5 | 54107.24 | 54160.81 |
| 597.3756 | 54819.38 | 54915.03 | 54731.97 | 54904.38 | 55175.58 | 54937.18 | 54934.48 | 54620.05 | 54694.93 | 54534.75 | 54551.45 | 54580.68 | 54066.45 | 54197.63 |
| 597.5097 | 54770.11 | 54894.98 | 54758.91 | 54939.98 | 55253.78 | 54901.58 | 54899.1 | 54571.05 | 54767.58 | 54537.6 | 54542.6 | 54603.78 | 54066.76 | 54222.88 |
| 597.6438 | 54696.49 | 54884.48 | 54784.41 | 54952.58 | 55128.38 | 54877.7 | 54794.35 | 54598.1 | 54815.1 | 54575.43 | 54560.88 | 54515.98 | 54052.74 | 54201 |
| 597.7779 | 54875.43 | 54968.23 | 54837.34 | 54977.83 | 55137.23 | 54948.75 | 54870.88 | 54575.4 | 54841.58 | 54488.98 | 54531.25 | 54602.98 | 54084.13 | 54191.38 |
| 597.9121 | 54815.16 | 55032.63 | 54764.94 | 54889.1 | 55119.03 | 55019.8 | 54930.83 | 54560.85 | 54757.15 | 54612.7 | 54588.53 | 54570.35 | 54133.26 | 54275.13 |
| 598.0461 | 54826.38 | 55064.6 | 54763.72 | 54815.28 | 55096.43 | 54967.73 | 54943.5 | 54555.3 | 54793.45 | 54574.85 | 54565.75 | 54526.55 | 54156.97 | 54228.75 |
| 598.1802 | 54852.49 | 54988.85 | 54751.28 | 54866.95 | 55080.13 | 55029.38 | 54903.7 | 54592.53 | 54760.63 | 54548.9 | 54491.5 | 54493.63 | 54141.24 | 54262.69 |
| 598.3143 | 54911.27 | 55032.73 | 54774.31 | 54928.13 | 55298.25 | 54981.43 | 54929.3 | 54652.15 | 54785.3 | 54580.15 | 54521.85 | 54428.8 | 54093.61 | 54302.81 |
| 598.4485 | 54848.27 | 54831.68 | 54906.84 | 54932.53 | 55348.68 | 54956.73 | 55029.48 | 54625.33 | 54886.68 | 54570.58 | 54433.83 | 54484.95 | 54112.66 | 54291.19 |
| 598.5826 | 54838.54 | 54836.3 | 54871.56 | 54892.03 | 55189.2 | 54895.45 | 54945.75 | 54655.48 | 54830.25 | 54570.15 | 54497.7 | 54448.1 | 54112.58 | 54360 |
| 598.7167 | 54848.49 | 54877.85 | 54880.81 | 54871.75 | 55098.18 | 54940.95 | 54939.2 | 54562.33 | 54790.58 | 54579.8 | 54426.73 | 54443.68 | 54121.63 | 54268.56 |
| 598.8508 | 54819.65 | 54976.8 | 54798.47 | 54935.7 | 55230.7 | 54911.68 | 54913.73 | 54601 | 54718.95 | 54535.18 | 54428.78 | 54505.3 | 54080.32 | 54237.38 |
| 598.9849 | 54769.95 | 55032.4 | 54810.59 | 54896.43 | 55093 | 55016.38 | 54982.6 | 54633.13 | 54807.53 | 54560.05 | 54525.73 | 54597.98 | 54080.45 | 54184.38 |
| 599.1189 | 54727.41 | 55012.75 | 54780.56 | 54944.95 | 55025 | 54994.78 | 54929.78 | 54575.05 | 54791.88 | 54451.3 | 54612.3 | 54534.88 | 54122.34 | 54278.06 |
| 599.253 | 54861.27 | 54894.9 | 54902.84 | 54964.33 | 55030.78 | 54986.85 | 54932.55 | 54635.18 | 54864.08 | 54540.7 | 54470.53 | 54510.4 | 54148.74 | 54230.94 |
| 599.3871 | 54899.54 | 54892.88 | 54791.5 | 54899.28 | 55094.63 | 55004.78 | 54872.7 | 54589.28 | 54792.93 | 54596.33 | 54485.73 | 54470.48 | 54074.21 | 54248.63 |
| 599.5212 | 54839.84 | 54882.48 | 54801.94 | 54897.88 | 55167.58 | 54854.65 | 54797.7 | 54570.9 | 54735.85 | 54714.78 | 54497.55 | 54407.43 | 54065.74 | 54193.94 |
| 599.6552 | 54763.41 | 54865.93 | 54649.63 | 54878.55 | 55201.95 | 54869.78 | 54882.1 | 54589.3 | 54672.95 | 54555.73 | 54516.6 | 54493.35 | 54137.18 | 54226.13 |
| 599.7893 | 54863.24 | 54891.85 | 54766.31 | 54867.65 | 55185.75 | 54991.78 | 54858.43 | 54535.28 | 54683.25 | 54552.38 | 54353.15 | 54489.93 | 54103.13 | 54216.31 |
| 599.9233 | 54779.11 | 54865.28 | 54735.53 | 54920.58 | 55087.8 | 55061.38 | 54888.18 | 54509.38 | 54806.63 | 54541.28 | 54400.8 | 54486.03 | 54088.71 | 54246.13 |
| 600.0574 | 54876.68 | 54885.35 | 54768.47 | 54928.55 | 55025.75 | 55024 | 54864.98 | 54548.45 | 54700.18 | 54525.33 | 54461.1 | 54565.78 | 54078.16 | 54275.38 |
| 600.1915 | 54858.86 | 54941.45 | 54752.47 | 54877.38 | 55161.5 | 54905.9 | 54807.33 | 54589.03 | 54764.38 | 54552 | 54477.43 | 54521.93 | 54042.26 | 54365.06 |
| 600.3256 | 54806.16 | 54919.43 | 54796.06 | 54886.73 | 55086.58 | 54956.35 | 54811.03 | 54608.08 | 54743.78 | 54567.18 | 54520.15 | 54487.45 | 54062.08 | 54266.75 |
| 600.4596 | 54847.08 | 54803.55 | 54699.53 | 54932.73 | 55148.18 | 55099.88 | 54866.23 | 54591.53 | 54692.38 | 54524.1 | 54459.95 | 54460.98 | 54048.42 | 54185.38 |
| 600.5936 | 54898.11 | 54828.75 | 54714.66 | 54832.75 | 55083.2 | 55068.93 | 54872.33 | 54653.58 | 54766.4 | 54465.73 | 54388.9 | 54421.53 | 54062.05 | 54346.06 |
| 600.7277 | 54742.59 | 54813.5 | 54753.53 | 54855.23 | 55088.63 | 54976.15 | 54881.78 | 54697.75 | 54753.4 | 54409.68 | 54442.45 | 54413.3 | 54088.5 | 54357.81 |
| 600.8617 | 54881.24 | 54837.35 | 54758.94 | 54934.2 | 55025.58 | 55049.05 | 54932.08 | 54606.33 | 54760.68 | 54446.25 | 54520.38 | 54437.53 | 54038.18 | 54285 |
| 600.9957 | 54721.73 | 54898.78 | 54727.84 | 55013.38 | 55058.6 | 55105.5 | 54954.5 | 54484.15 | 54806.03 | 54524.8 | 54641.55 | 54464.45 | 54069.66 | 54324.81 |
| 601.1298 | 54778.11 | 54892.5 | 54818.34 | 55016.88 | 55152.88 | 55016.8 | 54950.13 | 54550.35 | 54852.95 | 54593.83 | 54633.3 | 54572.13 | 54186.97 | 54346.5 |
| 601.2638 | 54872.54 | 54884.43 | 54840.78 | 55024.83 | 55233.25 | 55124.88 | 54863.1 | 54602.75 | 54833.08 | 54584.38 | 54583.05 | 54517.33 | 54113.24 | 54350.69 |
| 601.3978 | 54968.03 | 55038.08 | 54774.88 | 55006.85 | 55231.78 | 55058.53 | 54910.83 | 54571.98 | 54808.65 | 54561.7 | 54539.05 | 54489.18 | 54093.18 | 54295.13 |
| 601.5319 | 54861.76 | 54996.38 | 54737.38 | 54914.78 | 55232.45 | 54969.65 | 54907.03 | 54524.68 | 54823.6 | 54645.23 | 54472.75 | 54498.2 | 54089.71 | 54358.19 |
| 601.6659 | 54847 | 54937.98 | 54797.53 | 55047.3 | 55220.7 | 55033.45 | 54840.73 | 54592.55 | 54915.93 | 54558.03 | 54499.68 | 54508.93 | 54075.74 | 54334.81 |
| 601.7999 | 54868.7 | 54943.03 | 54937.59 | 54979.48 | 55131.83 | 54943.28 | 54825.38 | 54556.28 | 54850.53 | 54566.7 | 54467.98 | 54518.45 | 54079.58 | 54389.06 |
| 601.9339 | 54947.16 | 54872.53 | 54711 | 54893.95 | 55241.53 | 54993.05 | 54954.93 | 54521.78 | 54885.68 | 54609.13 | 54434.53 | 54498.23 | 54067.34 | 54334.94 |
| 602.0679 | 55061.81 | 54884 | 54714 | 54927.23 | 55241.13 | 54900.28 | 54888.48 | 54542.73 | 54852.9 | 54618.38 | 54455.4 | 54489.9 | 54097.11 | 54356.13 |
| 602.2019 | 54900.35 | 54958.3 | 54759.88 | 54880.1 | 55208.48 | 54942.93 | 54897.13 | 54573.7 | 54937.6 | 54663.83 | 54486.93 | 54490.18 | 54091.16 | 54329.75 |
| 602.3359 | 54848.54 | 55055.98 | 54806.72 | 54934.98 | 55190.4 | 54911.33 | 54921.95 | 54572.53 | 54806.03 | 54757.5 | 54537.25 | 54547.45 | 54029.53 | 54266.06 |
| 602.4699 | 54862.57 | 54925.63 | 54799.88 | 54948.25 | 55166.1 | 55063.95 | 54957.05 | 54577.88 | 54820.83 | 54622.43 | 54438.05 | 54520.98 | 54054.71 | 54266.69 |
| 602.6039 | 54967.86 | 54966.18 | 54878.31 | 55020.68 | 55039.65 | 55115.1 | 54888.35 | 54614.9 | 54858.95 | 54628 | 54531.83 | 54497.05 | 54040.95 | 54491.94 |
| 602.7379 | 54918.51 | 55016.5 | 54901.09 | 55023.08 | 55181.8 | 55074.53 | 54984.85 | 54562.85 | 54808.78 | 54632.7 | 54598.55 | 54464 | 54134.21 | 54286.63 |
| 602.8719 | 54881.62 | 54882.4 | 54854 | 55119.3 | 55203.1 | 54939.28 | 55000.7 | 54601.93 | 54793.9 | 54647.58 | 54518.15 | 54485.8 | 54146.87 | 54347.5 |
| 603.0059 | 54885.92 | 54839.43 | 54845.44 | 55026.3 | 55224.85 | 54944.15 | 54952.88 | 54652.25 | 54812.45 | 54566.93 | 54509.9 | 54477.9 | 54087.68 | 54372.44 |
| 603.1398 | 54975.43 | 55002.18 | 54849.41 | 55160.9 | 55251.68 | 55019.88 | 55055.9 | 54586.08 | 54857.53 | 54585.05 | 54526.13 | 54503.43 | 54205.68 | 54388.25 |
| 603.2738 | 55018.76 | 54998.1 | 54901.88 | 55154.53 | 55299.4 | 55037.95 | 54984.75 | 54537.6 | 54877.73 | 54608.43 | 54491.28 | 54502.55 | 54174.68 | 54370.5 |
| 603.4078 | 54993.05 | 54974.5 | 54972.41 | 55006.65 | 55223.9 | 55037.53 | 54980.08 | 54594.63 | 54856.8 | 54611.15 | 54516.48 | 54544.35 | 54169.84 | 54493.13 |
| 603.5418 | 54897.32 | 54953.25 | 54904.09 | 55020.08 | 55235.93 | 55120.93 | 54986.9 | 54623.78 | 54822.98 | 54494.18 | 54548.35 | 54610.28 | 54159.32 | 54440.31 |
| 603.6757 | 55140.81 | 54858.63 | 54901.91 | 55038.03 | 55248.73 | 55126.73 | 55016.85 | 54566.93 | 54823.1 | 54571.5 | 54609.3 | 54628.6 | 54135.61 | 54373.75 |
| 603.8097 | 54990.24 | 54873.53 | 54932.25 | 55024.88 | 55244.08 | 55019.93 | 54939.4 | 54572.68 | 54845.85 | 54609.75 | 54572.93 | 54543.93 | 54089.29 | 54317.25 |
| 603.9437 | 54904.27 | 54943.58 | 54861.38 | 54967.33 | 55295.35 | 55041.48 | 55017.08 | 54558.75 | 54828.8 | 54519.88 | 54611.8 | 54519.08 | 54127.29 | 54349.88 |
| 604.0776 | 54898.16 | 54939.38 | 54839.78 | 55037.38 | 55267.68 | 54982.13 | 54999.85 | 54538.65 | 54801.43 | 54598.45 | 54637.83 | 54454.85 | 54171.92 | 54395.88 |
| 604.2116 | 54904.97 | 54908.65 | 54809.41 | 55150 | 55235.18 | 54962.5 | 54984.33 | 54623.15 | 54902.6 | 54623.15 | 54612.1 | 54532.25 | 54098.5 | 54367.75 |
| 604.3455 | 54845.92 | 54936.58 | 54764.28 | 55072.68 | 55286.93 | 55133 | 55058.45 | 54596.15 | 54737.4 | 54599.8 | 54564.4 | 54513.1 | 54108.11 | 54257.75 |
| 604.4794 | 54873.43 | 54938.63 | 54778 | 54971.8 | 55191.8 | 55123.18 | 54945.68 | 54598.7 | 54774.9 | 54566.1 | 54594.35 | 54538.7 | 54101 | 54230.25 |
| 604.6134 | 54863.68 | 54936 | 54712.97 | 54881.95 | 55176.78 | 55051.48 | 54875.58 | 54535.38 | 54873.55 | 54514.83 | 54465.03 | 54488.75 | 54084.13 | 54250.94 |
| 604.7473 | 54936.97 | 55004.2 | 54758.63 | 54906.28 | 55226.5 | 54990.53 | 54920.2 | 54501.35 | 54903.35 | 54496.25 | 54468.9 | 54534.68 | 54110.92 | 54322.06 |
| 604.8813 | 54877.11 | 54982.93 | 54910.88 | 55024.48 | 55225.28 | 54960.7 | 54860.7 | 54613.25 | 54765.38 | 54500.5 | 54471.43 | 54592.93 | 54113.76 | 54430.19 |
| 605.0152 | 54960.08 | 55028.65 | 54800.91 | 55007.83 | 55186.5 | 54940.93 | 54865.23 | 54583.63 | 54745.53 | 54576.75 | 54535.05 | 54621.45 | 54100.13 | 54331.75 |
| 605.1491 | 54815.7 | 54911.58 | 54671.44 | 55009.08 | 55232.4 | 54945.63 | 54955.58 | 54614.15 | 54847.83 | 54518.75 | 54551.73 | 54604.35 | 54095.16 | 54279.44 |
| 605.283 | 54974.89 | 54952.4 | 54680.53 | 54947.18 | 55123.73 | 54906.2 | 54926.88 | 54627.2 | 54807.48 | 54496.55 | 54573.08 | 54504.23 | 54135.74 | 54321.88 |
| 605.417 | 54923.68 | 54929.43 | 54766.91 | 54988.08 | 55163.58 | 54996.13 | 54935.88 | 54532.33 | 54873.95 | 54592.18 | 54569.35 | 54536.95 | 54174.45 | 54301.69 |
| 605.5509 | 54991.38 | 54940.65 | 54879.66 | 54954.15 | 55062.5 | 54988.6 | 54815.93 | 54542.85 | 54834.78 | 54636.48 | 54526.38 | 54580.55 | 54118.26 | 54362.75 |
| 605.6848 | 54939 | 54844.75 | 54773.44 | 54957.4 | 55164.13 | 54944.63 | 54840.6 | 54539.23 | 54792.2 | 54614.2 | 54515.08 | 54536.43 | 54073.18 | 54311.31 |
| 605.8187 | 54909.7 | 54983.55 | 54788.41 | 54992.53 | 55171.03 | 55014.25 | 54931.45 | 54527.5 | 54772.7 | 54620.6 | 54515.6 | 54609.53 | 54076.61 | 54225.94 |
| 605.9526 | 54965.54 | 54960.7 | 54792.28 | 54941.4 | 55136.45 | 54995.83 | 54926 | 54617.28 | 54824.55 | 54616.2 | 54482.1 | 54513.9 | 54133.55 | 54176.31 |
| 606.0865 | 54991.14 | 54973.03 | 54919.72 | 55006.33 | 55174.08 | 54966.18 | 54963.75 | 54549.4 | 54822.35 | 54583.65 | 54515.08 | 54519.23 | 54164.05 | 54337.06 |
| 606.2204 | 54830.92 | 54962.2 | 54826.53 | 54991.33 | 55170.15 | 54977.25 | 54889.78 | 54554.45 | 54764.38 | 54555.65 | 54461.73 | 54534.9 | 54160.24 | 54351.75 |
| 606.3543 | 54871.7 | 54963.15 | 54797.63 | 55076.93 | 55099.53 | 54970.63 | 54941.5 | 54619.18 | 54752.45 | 54657.78 | 54473.13 | 54570.3 | 54089.89 | 54303.69 |
| 606.4882 | 54959.54 | 55017.98 | 54834.5 | 55068.98 | 55128.78 | 54990.25 | 54974.93 | 54655.08 | 54747.1 | 54666.6 | 54560.93 | 54480.23 | 54033.82 | 54195.06 |
| 606.6221 | 55049.49 | 55034.9 | 54883.28 | 55016.75 | 55180.1 | 55109.7 | 55056.03 | 54645.3 | 54804.4 | 54715.3 | 54445.28 | 54479.83 | 54091.68 | 54258.63 |
| 606.756 | 54979.51 | 55068.53 | 54902.72 | 54932.83 | 55298.03 | 55053.98 | 55008.75 | 54711.38 | 54925.03 | 54617.7 | 54510.95 | 54536.7 | 54143.45 | 54303.25 |
| 606.8898 | 54970.62 | 54984.05 | 54765.25 | 54932.58 | 55234.5 | 55083 | 54892.1 | 54537.48 | 54805.65 | 54475.45 | 54474.6 | 54537.65 | 54193.32 | 54401.56 |
| 607.0237 | 54986.59 | 54924.53 | 54799.72 | 54942.4 | 55208.73 | 55115.78 | 54894.83 | 54613.35 | 54708.5 | 54490.15 | 54505.28 | 54517.3 | 54115.42 | 54257.94 |
| 607.1576 | 54967.84 | 55005.73 | 54881.84 | 55030.58 | 55253.23 | 55092.48 | 54911.3 | 54611.78 | 54793.3 | 54586.95 | 54524.13 | 54494.58 | 54091.03 | 54236.88 |
| 607.2914 | 54927.49 | 54937.23 | 54810.78 | 54992.48 | 55219.23 | 55095.18 | 54946.73 | 54567.98 | 54784.13 | 54648.23 | 54536.65 | 54531.05 | 54064.16 | 54264.5 |
| 607.4253 | 54953.81 | 54868.25 | 54698.88 | 54950.75 | 55285.05 | 55055.4 | 55029.93 | 54509.25 | 54743.53 | 54549.1 | 54428.75 | 54538.55 | 54057.66 | 54190.13 |
| 607.5592 | 54879.86 | 54875.25 | 54779.44 | 54897.05 | 55212.5 | 55000.83 | 54969.43 | 54579 | 54793.9 | 54533.95 | 54379.7 | 54497.35 | 54087.87 | 54246.69 |
| 607.6931 | 54764.08 | 54916.35 | 54720.25 | 54891.03 | 55100.48 | 55080.25 | 54883.75 | 54586.93 | 54855.55 | 54479.35 | 54404.33 | 54430.1 | 54077.58 | 54341.31 |
| 607.8269 | 54833.57 | 54862.8 | 54630.19 | 54918.25 | 55049.73 | 54903.03 | 54885.05 | 54572.6 | 54819.15 | 54508.5 | 54425.75 | 54403.35 | 54029.05 | 54309.5 |
| 607.9608 | 54835.78 | 54929.05 | 54625.16 | 54943.3 | 55090.78 | 55033.88 | 54905.55 | 54629.63 | 54790.4 | 54550.58 | 54527 | 54525.25 | 54025.5 | 54251.25 |
| 608.0946 | 54910.97 | 54932 | 54778.38 | 54922.83 | 55065.3 | 54917.23 | 54953.38 | 54548 | 54721.88 | 54637.93 | 54563.08 | 54516.68 | 54034.76 | 54196.63 |
| 608.2285 | 54851 | 55037.18 | 54630.22 | 54928.15 | 55072.65 | 54857.8 | 54860.9 | 54519.48 | 54715.8 | 54598.05 | 54544.55 | 54504 | 54081.37 | 54205.38 |
| 608.3622 | 54822.68 | 55001.35 | 54715.75 | 54948.68 | 55160.38 | 54922.88 | 54846.6 | 54530.5 | 54721.28 | 54644.6 | 54490.38 | 54560.03 | 54135.03 | 54234.81 |
| 608.4961 | 54860.92 | 54933.48 | 54715.84 | 54965.25 | 55179.65 | 55052.53 | 54911.13 | 54525.48 | 54684.2 | 54606.4 | 54475.45 | 54474.63 | 54066.32 | 54303.56 |
| 608.6299 | 54878.73 | 54835 | 54760.69 | 55008.78 | 55082.3 | 54998.98 | 54995.45 | 54596.4 | 54634.95 | 54601.03 | 54470.33 | 54457.63 | 54033.16 | 54305.94 |
| 608.7637 | 54993.11 | 54863.73 | 54831.34 | 54941.23 | 55146.13 | 55056.73 | 54890.7 | 54631.85 | 54859.73 | 54631.3 | 54474.48 | 54395.93 | 54037.45 | 54197.44 |
| 608.8976 | 54922.14 | 54923.85 | 54898.03 | 54974.23 | 55236.78 | 55016.03 | 54878.33 | 54594.45 | 54858.63 | 54573.9 | 54462.88 | 54416.58 | 54065.34 | 54215.25 |
| 609.0314 | 54898.3 | 54983.35 | 54808 | 55045.48 | 55106.68 | 55064 | 54830.65 | 54611.53 | 54858.03 | 54520.13 | 54488.23 | 54422.63 | 54052.58 | 54135.5 |
| 609.1652 | 54847.78 | 54952.38 | 54794.19 | 55049.98 | 55149.03 | 55000.35 | 54847.13 | 54624.85 | 54889.63 | 54572.1 | 54479.9 | 54443.53 | 54050.39 | 54167.25 |
| 609.299 | 54897.95 | 54864.43 | 54784.38 | 55115.55 | 55192.78 | 55024.38 | 54798.93 | 54599.43 | 54827.65 | 54588.7 | 54412.98 | 54520.13 | 54114.39 | 54223.5 |
| 609.4329 | 54935.43 | 54935.03 | 54748.38 | 55102.88 | 55078.4 | 55090.55 | 54845.35 | 54643.35 | 54772.08 | 54608.08 | 54424.53 | 54497.58 | 54082.34 | 54180.63 |
| 609.5667 | 54884.19 | 54994.65 | 54795.84 | 55017.15 | 55119.2 | 55055.8 | 54896.28 | 54614.55 | 54765.83 | 54570.7 | 54472.43 | 54441.08 | 54037.5 | 54230.06 |
| 609.7004 | 54899.78 | 55093.98 | 54876.66 | 55004.98 | 55244.53 | 55028.05 | 54837.18 | 54624.05 | 54881.05 | 54612.95 | 54511.6 | 54563.15 | 54017.79 | 54151.31 |
| 609.8342 | 54846.81 | 54954.98 | 54880.47 | 54959.05 | 55146.9 | 54993.75 | 54917.18 | 54636 | 54837.95 | 54613.55 | 54570.28 | 54505.35 | 54041.95 | 54252 |
| 609.968 | 54854.86 | 54943.15 | 54818.28 | 54945.7 | 55251.9 | 55126.5 | 54924.73 | 54581.5 | 54808.63 | 54609.53 | 54508.35 | 54552.4 | 54056.84 | 54301.69 |
| 610.1019 | 54831.16 | 54979 | 54884 | 54906.23 | 55326.4 | 55148.73 | 55051.3 | 54704.15 | 54789.28 | 54571.1 | 54499.7 | 54566.8 | 54079.24 | 54245.31 |
| 610.2356 | 54888.11 | 55035.05 | 54788.56 | 54943.13 | 55262.75 | 55113.05 | 54976.73 | 54652.75 | 54821.45 | 54586.28 | 54420.43 | 54473.4 | 54103.95 | 54292.06 |
| 610.3694 | 54834.95 | 55059.6 | 54804.91 | 55005.05 | 55255.33 | 55061.43 | 54931.48 | 54498.2 | 54882.85 | 54635.38 | 54573.08 | 54538.28 | 54089.82 | 54327.44 |
| 610.5032 | 55056.73 | 54980.63 | 54917 | 55074.68 | 55293.18 | 55076.9 | 54909.63 | 54533.7 | 54907.23 | 54662.65 | 54650.2 | 54472.15 | 54109.84 | 54345 |
| 610.637 | 55109.65 | 55130.15 | 54798.97 | 55045.08 | 55197.45 | 55095.75 | 54946.28 | 54698.05 | 54912.5 | 54607.85 | 54548.48 | 54551.4 | 54023.18 | 54317.5 |
| 610.7708 | 54955.65 | 55076.3 | 54778.88 | 55023.88 | 55275 | 55177.58 | 54980.5 | 54643.73 | 54891.83 | 54571.25 | 54506.58 | 54594.25 | 54018.11 | 54356.69 |
| 610.9045 | 54879.68 | 55024.1 | 54782.19 | 55056.5 | 55290.65 | 55131.88 | 54936.28 | 54768.08 | 54966.35 | 54631.15 | 54500.75 | 54475.55 | 54053.76 | 54353.69 |
| 611.0383 | 54928.73 | 55124.33 | 54886.97 | 55093.55 | 55184.18 | 55077.7 | 54845.18 | 54738.78 | 54801.05 | 54567.75 | 54495.05 | 54555.1 | 54071.87 | 54307.06 |
| 611.1721 | 54923.27 | 55025.95 | 54835.69 | 55025.18 | 55187.18 | 55142.43 | 54863 | 54695.1 | 54901.48 | 54589.3 | 54559.93 | 54533.58 | 54096.76 | 54339.13 |
| 611.3058 | 54982.14 | 55076.35 | 54848.22 | 55045.43 | 55138.05 | 55086.48 | 54877.05 | 54635.23 | 54835.93 | 54639.6 | 54560.03 | 54581.3 | 54083.95 | 54341.63 |
| 611.4395 | 54895.24 | 55035.68 | 54905 | 55016.15 | 55115.58 | 54991.58 | 54982.18 | 54668.98 | 54857.88 | 54557 | 54513.7 | 54524.7 | 54119.79 | 54347.56 |
| 611.5733 | 54917.11 | 54963.4 | 54842.19 | 55088.6 | 55197.05 | 55116.4 | 55046.8 | 54671.75 | 54986.65 | 54610.73 | 54510.75 | 54556.98 | 54102.53 | 54254.81 |
| 611.707 | 54982.76 | 55085.73 | 54839.03 | 55103.43 | 55205.48 | 55189.83 | 55127.9 | 54779.8 | 55022 | 54608.7 | 54492.1 | 54510.4 | 54084.68 | 54439.25 |
| 611.8408 | 54928.92 | 55099 | 54893.72 | 55112.28 | 55220.05 | 55145.05 | 55097.55 | 54732.75 | 54992.3 | 54676.73 | 54575.18 | 54491.45 | 54145.63 | 54375 |
| 611.9745 | 55010.92 | 55050.75 | 54933.56 | 55222.03 | 55359.25 | 55134.85 | 55076.68 | 54783.88 | 54881.4 | 54634.15 | 54537.1 | 54564.5 | 54097.08 | 54303.75 |
| 612.1083 | 55033.19 | 55110.15 | 55000.84 | 55237.93 | 55420.63 | 55126.73 | 54994.3 | 54733.95 | 54941.55 | 54595.85 | 54543.18 | 54536.78 | 54128.11 | 54356.94 |
| 612.242 | 55120.92 | 55138.58 | 55007.5 | 55102.8 | 55395.05 | 55193.73 | 55128.88 | 54702.65 | 55027.28 | 54743.7 | 54566.55 | 54540.08 | 54100.53 | 54382.56 |
| 612.3757 | 55092.54 | 55150.4 | 54949.53 | 55170.2 | 55392.53 | 55197.38 | 55227.2 | 54688.18 | 55053 | 54620.1 | 54570.9 | 54625.23 | 54051.82 | 54411.75 |
| 612.5095 | 55055.3 | 55126.63 | 54972.5 | 55326.65 | 55456.1 | 55251.73 | 55243.18 | 54787.8 | 55006.95 | 54653.1 | 54697.78 | 54635.05 | 54111 | 54350.31 |
| 612.6431 | 55153.3 | 55098.45 | 55060.75 | 55338.78 | 55444.8 | 55162.53 | 55200.65 | 54833.23 | 54951.23 | 54638.4 | 54576.83 | 54658.6 | 54085.37 | 54344.06 |
| 612.7769 | 55134.38 | 55230.88 | 55152.09 | 55230.85 | 55429.2 | 55276.8 | 55166.38 | 54782.9 | 55036.1 | 54669.63 | 54477.38 | 54615.68 | 54073.47 | 54316.06 |
| 612.9106 | 55233.46 | 55358.53 | 55026 | 55326.28 | 55423 | 55319.85 | 55261.13 | 54765.93 | 55071.9 | 54690.93 | 54577.55 | 54686.43 | 54101.16 | 54466.13 |
| 613.0443 | 55282.95 | 55203.85 | 54932.53 | 55290.83 | 55361.8 | 55350.73 | 55234.53 | 54830.35 | 55098.8 | 54695.7 | 54647.05 | 54789.43 | 54140.39 | 54352.88 |
| 613.178 | 55216.3 | 55296.88 | 55086.09 | 55327.63 | 55403.33 | 55425.2 | 55225.45 | 54803.18 | 55000.08 | 54759.55 | 54688.65 | 54725.28 | 54160.13 | 54355.69 |
| 613.3117 | 55194.38 | 55296.08 | 55099.56 | 55395 | 55529.83 | 55446.33 | 55268.68 | 54771.18 | 55059.23 | 54785.63 | 54718.23 | 54787.28 | 54137.21 | 54449.63 |
| 613.4454 | 55247.03 | 55236.43 | 55056.56 | 55404.05 | 55638.55 | 55422.53 | 55322.9 | 54718.1 | 55182.2 | 54798.55 | 54645.88 | 54751.2 | 54158.63 | 54359.38 |
| 613.5791 | 55164.65 | 55183.6 | 55091.31 | 55389.75 | 55659.48 | 55414.23 | 55308.85 | 54766.73 | 55111.25 | 54876.08 | 54640.05 | 54711.63 | 54166.61 | 54499.38 |
| 613.7128 | 55229.11 | 55236.6 | 55149.38 | 55341.7 | 55506.13 | 55449.98 | 55349.8 | 54783.2 | 55072.23 | 54822.83 | 54589.48 | 54702 | 54205.55 | 54353.56 |
| 613.8464 | 55201.57 | 55175.28 | 55211.59 | 55273.28 | 55602.33 | 55521.45 | 55193.83 | 54745.48 | 55094.88 | 54788.23 | 54617.7 | 54664.93 | 54180.87 | 54307.94 |
| 613.9802 | 55127.49 | 55214.88 | 55131.53 | 55391.85 | 55693.75 | 55408.98 | 55224.28 | 54761.7 | 55163.6 | 54762.9 | 54639.33 | 54730.33 | 54160.16 | 54461.5 |
| 614.1138 | 55185.89 | 55300.88 | 55257.03 | 55302.63 | 55617.93 | 55368.95 | 55235.4 | 54840.85 | 55166.85 | 54817.23 | 54616.93 | 54749.05 | 54189.5 | 54346.63 |
| 614.2475 | 55149.7 | 55250.65 | 55214.59 | 55308.95 | 55583.83 | 55447.15 | 55289.4 | 54922.18 | 55151.95 | 54857.7 | 54604.6 | 54751.83 | 54224.42 | 54410.38 |
| 614.3812 | 55252.41 | 55228.95 | 55113 | 55201.6 | 55722.73 | 55431.63 | 55374.28 | 54922.88 | 55171.5 | 54815.88 | 54715.58 | 54744.23 | 54185.18 | 54382.44 |
| 614.5148 | 55225.7 | 55357.33 | 55116.13 | 55339.5 | 55658.3 | 55345.53 | 55376.35 | 54852.13 | 55256.53 | 54864.98 | 54681.45 | 54738.1 | 54124.18 | 54350.5 |
| 614.6485 | 55319.08 | 55335.65 | 55112.34 | 55491.48 | 55742.25 | 55266.93 | 55430.18 | 54899.43 | 55256.8 | 54891.23 | 54786.85 | 54823 | 54142.18 | 54323.69 |
| 614.7822 | 55205.59 | 55200.58 | 55237.59 | 55457.38 | 55886.73 | 55311.58 | 55390.68 | 54874.8 | 55318.43 | 54867.28 | 54755.33 | 54752.7 | 54189.37 | 54334.88 |
| 614.9158 | 55229.84 | 55207.35 | 55137.09 | 55504.43 | 55808.18 | 55458.73 | 55455.13 | 54931.25 | 55317.6 | 54950.5 | 54812.53 | 54714.03 | 54227.61 | 54351.63 |
| 615.0494 | 55283 | 55236.1 | 55069.66 | 55594.4 | 55675.05 | 55529.03 | 55410.8 | 54877.85 | 55295.5 | 54953.63 | 54721.43 | 54762.9 | 54204.37 | 54364.88 |
| 615.1831 | 55361.32 | 55352.08 | 55115.38 | 55523.4 | 55670.55 | 55587.48 | 55395.23 | 54942.43 | 55244.88 | 54896.35 | 54764.48 | 54729.75 | 54221.82 | 54444.19 |
| 615.3168 | 55343.19 | 55358.8 | 55268.13 | 55550.33 | 55802.13 | 55397.3 | 55418.53 | 54908.63 | 55234.33 | 54966.33 | 54854.98 | 54785.1 | 54241.74 | 54397.19 |
| 615.4504 | 55309.43 | 55345.33 | 55200.06 | 55360.08 | 55709.5 | 55365.6 | 55408.15 | 54948.23 | 55185.25 | 54920.25 | 54828.5 | 54771.93 | 54231.26 | 54439.56 |
| 615.5841 | 55473.46 | 55359.3 | 55050.06 | 55380.68 | 55781.75 | 55438.18 | 55414.08 | 54913.35 | 55230.9 | 54925.55 | 54842.2 | 54803.08 | 54125.37 | 54433.44 |
| 615.7177 | 55390.11 | 55347.33 | 55093.06 | 55334.43 | 55782.65 | 55586.63 | 55463.18 | 54953.75 | 55272.75 | 54890.83 | 54851.5 | 54896.58 | 54107.37 | 54371.19 |
| 615.8513 | 55415.51 | 55221.5 | 55132.66 | 55374.23 | 55916.65 | 55508.93 | 55402.78 | 55021.08 | 55141.3 | 54877.4 | 54954.35 | 54833.48 | 54193.92 | 54503.94 |
| 615.9849 | 55463.08 | 55306.28 | 55217.38 | 55471.48 | 55810.35 | 55558.83 | 55364.35 | 55007.6 | 55339.1 | 54953.38 | 54857.88 | 54818.7 | 54256.39 | 54474.69 |
| 616.1185 | 55425.24 | 55324.43 | 55146.28 | 55580.73 | 55863.83 | 55516.1 | 55376.45 | 54896.65 | 55225.83 | 54955.48 | 54892.05 | 54757.48 | 54211.68 | 54396.94 |
| 616.2521 | 55509.3 | 55455.58 | 55130.53 | 55616.15 | 55765.3 | 55486.33 | 55398.4 | 54919.35 | 55131.93 | 55027.7 | 54860.68 | 54792.53 | 54218.66 | 54387.19 |
| 616.3857 | 55408 | 55386.58 | 55291.25 | 55515.08 | 55707.23 | 55557.65 | 55519.93 | 55002.08 | 55248.25 | 55146.78 | 54869.13 | 54906.85 | 54190.95 | 54415.81 |
| 616.5194 | 55403 | 55409.48 | 55204.88 | 55536.05 | 55727.85 | 55557.58 | 55445.08 | 55034.73 | 55179.33 | 55063.73 | 54861.68 | 54904.55 | 54160.47 | 54410.31 |
| 616.653 | 55430.24 | 55351.58 | 55276.06 | 55569.13 | 55796.28 | 55621.73 | 55425.43 | 55029.98 | 55276.43 | 55044.28 | 54924.58 | 54832.75 | 54196.29 | 54435.31 |
| 616.7866 | 55551.05 | 55470.28 | 55293.56 | 55577.75 | 55821.33 | 55578.23 | 55377.08 | 54930.43 | 55350.33 | 55089.13 | 54918.15 | 54822.13 | 54191.13 | 54455.88 |
| 616.9202 | 55549 | 55426.3 | 55113.13 | 55667.33 | 55780.98 | 55679.45 | 55434.88 | 54882.35 | 55430.58 | 55027.05 | 54899.55 | 54832.93 | 54277.89 | 54397.19 |
| 617.0538 | 55502.73 | 55496.2 | 55334.41 | 55672.98 | 56037.08 | 55719.45 | 55588.4 | 55139.55 | 55462.28 | 55081.75 | 54915.9 | 54876.35 | 54360.79 | 54593.94 |
| 617.1874 | 55523.7 | 55452.98 | 55380.31 | 55677.78 | 56024.98 | 55690.4 | 55577.6 | 55060.1 | 55523.23 | 55076.48 | 54877.3 | 54887.43 | 54259.95 | 54591 |
| 617.3209 | 55634.86 | 55564.38 | 55410.44 | 55595.58 | 55991.68 | 55593.33 | 55621.23 | 54950.2 | 55364.5 | 55052.88 | 54890.6 | 54934.1 | 54246.16 | 54534.63 |
| 617.4545 | 55448.14 | 55367.95 | 55422.13 | 55548.7 | 56116.6 | 55820.7 | 55624.53 | 54998.83 | 55322.95 | 55042.5 | 54828.33 | 54998.33 | 54186.84 | 54643.63 |
| 617.5881 | 55505.16 | 55580.03 | 55349.47 | 55482.98 | 55976.68 | 55798.85 | 55697.3 | 55041.25 | 55301.28 | 55035.13 | 54811.03 | 54965.25 | 54144.16 | 54562.88 |
| 617.7217 | 55563.65 | 55683.78 | 55338.13 | 55506.35 | 55947.4 | 55655.68 | 55578.75 | 55176.95 | 55361.7 | 55174.65 | 54745.53 | 54996.43 | 54262.55 | 54553.06 |
| 617.8552 | 55468.86 | 55505.48 | 55353.75 | 55602.8 | 55982.05 | 55643.23 | 55592.28 | 55191.88 | 55326.45 | 55224.2 | 54813.28 | 55016.68 | 54237.21 | 54530.69 |
| 617.9888 | 55424.14 | 55445.13 | 55409.88 | 55561.35 | 55866.58 | 55552.45 | 55486.2 | 55189.33 | 55338.55 | 55173.6 | 54960.73 | 54999.43 | 54255.76 | 54497.5 |
| 618.1224 | 55474.57 | 55545.18 | 55388.22 | 55570.45 | 55938.53 | 55657.35 | 55756.78 | 55261.6 | 55257.63 | 55020.75 | 54924.88 | 55025.83 | 54316.82 | 54614 |
| 618.2559 | 55575.84 | 55583.15 | 55421.13 | 55717.3 | 56048.48 | 55696.65 | 55757.33 | 55205.15 | 55342 | 55199.45 | 54999.98 | 55047.75 | 54283.5 | 54576.19 |
| 618.3895 | 55584.76 | 55595.25 | 55495.63 | 55641.18 | 55967.28 | 55764.23 | 55629.13 | 55364.13 | 55290.28 | 55085.45 | 55038.85 | 54910.73 | 54210.5 | 54597.38 |
| 618.523 | 55587.76 | 55701.75 | 55414.22 | 55639.33 | 55971.35 | 55728.5 | 55727.63 | 55275.23 | 55588.08 | 55132.58 | 54896.58 | 54918.4 | 54265.5 | 54576.63 |
| 618.6566 | 55638.22 | 55733.68 | 55337.28 | 55798.6 | 56020.93 | 55719.18 | 55789.25 | 55340.63 | 55551.95 | 55109.78 | 54927.03 | 55040.3 | 54284.08 | 54664.63 |
| 618.7901 | 55610.35 | 55639.7 | 55365.34 | 55925.48 | 56073.18 | 55805 | 55776.75 | 55207.03 | 55370.25 | 55154.38 | 55037.9 | 55004.9 | 54274.84 | 54668 |
| 618.9237 | 55652.11 | 55661.18 | 55444.16 | 55847.3 | 55948.68 | 55929.88 | 55619.4 | 55319.35 | 55390.43 | 55251.38 | 54950.68 | 55020.58 | 54280.18 | 54592.06 |
| 619.0572 | 55687.97 | 55585.2 | 55461.44 | 55819.43 | 56124.7 | 55924.63 | 55590.03 | 55310.23 | 55474.8 | 55204.43 | 54959.38 | 54944.13 | 54329.34 | 54611.25 |
| 619.1907 | 55611.49 | 55752.88 | 55523.56 | 55672.45 | 56340.88 | 55821.75 | 55729.48 | 55323.53 | 55673.65 | 55226.98 | 55085.83 | 55037.95 | 54280.92 | 54758.63 |
| 619.3242 | 55663.22 | 55861.18 | 55557.47 | 55913.8 | 56391.48 | 55905.68 | 55680.35 | 55378.48 | 55656.78 | 55120.08 | 55096.15 | 55114.25 | 54347.42 | 54603.31 |
| 619.4578 | 55588.14 | 55785.13 | 55545.03 | 55936.25 | 56219.3 | 56030.9 | 55801.68 | 55362.53 | 55679.85 | 55133.1 | 55074.88 | 55185.4 | 54387.55 | 54602.5 |
| 619.5913 | 55658 | 55678.73 | 55661.72 | 55928.15 | 56146.4 | 56015.65 | 55850.43 | 55204.48 | 55632.55 | 55103.13 | 55072.63 | 55154.8 | 54232.47 | 54673.13 |
| 619.7248 | 55718.62 | 55759.23 | 55611.41 | 55864.85 | 56161.9 | 55946.23 | 55886.68 | 55350.13 | 55589.7 | 55180.75 | 55234.48 | 55282 | 54276.32 | 54827.5 |
| 619.8583 | 55698.89 | 55811.8 | 55604.5 | 55831.95 | 56007.6 | 56064.23 | 55842.9 | 55382.7 | 55567.35 | 55171.45 | 55108.03 | 55204.7 | 54381.53 | 54620.31 |
| 619.9918 | 55763.05 | 55792.65 | 55643.09 | 55830.18 | 56149.1 | 55875.68 | 55905.85 | 55184.7 | 55527.85 | 55207.9 | 55172.03 | 55017.93 | 54349.89 | 54553.69 |
| 620.1253 | 55632 | 55779.38 | 55591.25 | 55944.2 | 56194.9 | 55932.9 | 55886.18 | 55283.98 | 55685.6 | 55210.73 | 55272.9 | 55083.98 | 54320.89 | 54590.75 |
| 620.2588 | 55809.59 | 55676.93 | 55662.06 | 55916.9 | 56159.33 | 56020.93 | 55882.08 | 55319.03 | 55517.03 | 55136.43 | 55153.05 | 55123.88 | 54348.08 | 54579.63 |
| 620.3923 | 55894.54 | 55777.85 | 55587.34 | 55837.68 | 56226.4 | 56249.53 | 55975.95 | 55298.18 | 55506.73 | 55211.03 | 55159.98 | 55163.33 | 54365.97 | 54678.25 |
| 620.5258 | 55871.76 | 55786.8 | 55593.22 | 56054.23 | 56435.48 | 56222.38 | 55850.45 | 55282.53 | 55567.53 | 55269.63 | 55092.65 | 55001.1 | 54282.74 | 54799.69 |
| 620.6593 | 55917.19 | 55753.35 | 55580 | 56036.85 | 56278.3 | 56000.53 | 55816.1 | 55178.18 | 55622.98 | 55380.4 | 55208.73 | 54960.13 | 54372.76 | 54577.38 |
| 620.7928 | 55896.54 | 55942.53 | 55645.47 | 55998.05 | 56037.28 | 55838 | 55866.38 | 55328.55 | 55626.03 | 55364.08 | 55218.88 | 55036.58 | 54371.08 | 54693.56 |
| 620.9263 | 55833.62 | 55855.85 | 55624.09 | 56026.58 | 56123.65 | 55990.45 | 56022.15 | 55333.88 | 55724.93 | 55219.7 | 55115.83 | 55030.58 | 54375.79 | 54758.69 |
| 621.0598 | 55803.08 | 55776.9 | 55672.16 | 56015.4 | 56283.58 | 56074.45 | 55990.48 | 55438.15 | 55630.08 | 55220.18 | 55190.93 | 54975.85 | 54357.53 | 54766.06 |
| 621.1932 | 55923.95 | 55789.63 | 55696.84 | 56037.78 | 56259.8 | 56037.8 | 55879.6 | 55433.43 | 55563.98 | 55294.15 | 55210.75 | 55076.08 | 54337.55 | 54756.13 |
| 621.3267 | 55766 | 55689.15 | 55750.53 | 56038.23 | 56389.58 | 56160.55 | 55757.4 | 55498.75 | 55604.43 | 55407.03 | 55201.53 | 55100.58 | 54391.34 | 54637.69 |
| 621.4601 | 55723.38 | 55997.75 | 55767.13 | 55947.8 | 56604.65 | 56096 | 55849.38 | 55322.75 | 55568.73 | 55330.98 | 55183.55 | 55117.23 | 54374.16 | 54685.5 |
| 621.5936 | 55904.35 | 56117.98 | 55788.06 | 56074.43 | 56602.5 | 56062.75 | 55913.7 | 55334.65 | 55526.13 | 55277.38 | 55276.8 | 55114.35 | 54372.11 | 54690.88 |
| 621.7271 | 55759.57 | 56043.43 | 55691.84 | 56120.68 | 56413.5 | 56113.73 | 55873.35 | 55290.73 | 55731.33 | 55284.63 | 55279.9 | 55063.23 | 54421.63 | 54700.56 |
| 621.8605 | 55879.24 | 56044.38 | 55740.78 | 56233.03 | 56283.55 | 56095.83 | 55995.73 | 55321.88 | 55765.63 | 55291.03 | 55316.4 | 55026.48 | 54312.68 | 54718.06 |
| 621.994 | 55878.73 | 55966.4 | 55692.56 | 56123.93 | 56332.7 | 56065.23 | 56018.78 | 55429.65 | 55623.58 | 55260.08 | 55302.13 | 55175.33 | 54384.97 | 54697.31 |
| 622.1274 | 55896.3 | 55960.35 | 55603.69 | 56066.58 | 56464.53 | 56198.6 | 56052.2 | 55349.03 | 55680.38 | 55293.53 | 55230.98 | 55152.48 | 54379.08 | 54686.44 |
| 622.2608 | 55899.43 | 55957.1 | 55721.53 | 56066.73 | 56428.9 | 56194.15 | 55999.18 | 55388.15 | 55583.83 | 55314.43 | 55102.58 | 55070.5 | 54363.76 | 54574.88 |
| 622.3943 | 55833.97 | 55952.55 | 55725.47 | 55963.33 | 56266.83 | 56095.25 | 55821.68 | 55405.38 | 55635.75 | 55404.1 | 55054.73 | 55149.2 | 54377 | 54667.44 |
| 622.5277 | 55847.7 | 55934.63 | 55684.81 | 56085.13 | 56308.93 | 56035.78 | 55740.45 | 55375.33 | 55572.15 | 55374.73 | 55192.53 | 55277.88 | 54386.24 | 54699.25 |
| 622.6611 | 55917.32 | 55879.93 | 55664.41 | 56171.15 | 56501.33 | 56006.08 | 55904.58 | 55230.43 | 55670.53 | 55393.45 | 55220.73 | 55186.55 | 54369.42 | 54721.75 |
| 622.7946 | 55896.51 | 55892.05 | 55724.63 | 56087.8 | 56596.95 | 56175.65 | 55916.6 | 55275.83 | 55814.65 | 55341.08 | 55230.75 | 55155.23 | 54330.92 | 54705.88 |
| 622.928 | 55813.76 | 56000.38 | 55768.75 | 56129.93 | 56478.43 | 56260.33 | 55891.4 | 55358.68 | 55801.3 | 55370.45 | 55260.5 | 55106.68 | 54387.61 | 54744.44 |
| 623.0614 | 56021.84 | 55833.53 | 55833.53 | 56230.08 | 56476.98 | 56207.13 | 56105.43 | 55485 | 55837.3 | 55471.48 | 55186.85 | 55222.13 | 54392.18 | 54606.13 |
| 623.1948 | 55962.35 | 55914.15 | 55864.31 | 56284.65 | 56273.08 | 56170.58 | 55950.95 | 55484.98 | 55866.85 | 55157.43 | 55250.88 | 55196 | 54404.5 | 54777.31 |
| 623.3282 | 55913.22 | 55744.18 | 55766.22 | 56464.15 | 56415.38 | 56369.13 | 55925.6 | 55506.73 | 55757.28 | 55221.75 | 55160.88 | 55269.93 | 54444.89 | 54725.5 |
| 623.4616 | 55810.05 | 55835.7 | 55699.34 | 55888.73 | 56422.68 | 56358.43 | 55990.53 | 55524.85 | 55751.35 | 55406.8 | 55141.35 | 55112.83 | 54453.21 | 54821.63 |
| 623.595 | 55876.11 | 56068.58 | 55692.03 | 55807.93 | 56287.1 | 56105.18 | 56045.6 | 55492.8 | 55906.45 | 55390 | 55211.83 | 55042.95 | 54446.53 | 54604.19 |
| 623.7284 | 55910.41 | 55928.8 | 55705.56 | 56108.38 | 56137.05 | 56049.35 | 55945.15 | 55387.7 | 55719.33 | 55358.98 | 55205.88 | 55157.7 | 54360.61 | 54667 |
| 623.8618 | 55943.73 | 55930.18 | 55686.81 | 56052.65 | 56245.8 | 56129.75 | 55984.43 | 55456.68 | 55641.68 | 55395.6 | 55134.95 | 55303.35 | 54353.03 | 54718.69 |
| 623.9952 | 55966.73 | 55965.1 | 55682.63 | 55972.5 | 56149.63 | 55978.4 | 56193 | 55420.48 | 55787.28 | 55405.65 | 55235.08 | 55139.43 | 54345.84 | 54913.25 |
| 624.1285 | 55752.32 | 55917.88 | 55598.66 | 55993.78 | 56253.25 | 56021.73 | 55915.9 | 55353.9 | 55722.93 | 55476.3 | 55197.1 | 55141.7 | 54377.92 | 54723.63 |
| 624.2619 | 55800 | 55941.6 | 55557.63 | 55876.45 | 56293.58 | 56002.35 | 55841.18 | 55296.1 | 55820.15 | 55340.75 | 55213.93 | 55160.53 | 54335.71 | 54708.75 |
| 624.3953 | 55719.81 | 55953.93 | 55701.69 | 55940.88 | 56380.38 | 56065.15 | 55961.3 | 55366.23 | 55693.08 | 55322.73 | 55235.05 | 55158.95 | 54312.05 | 54864.75 |
| 624.5287 | 55805.68 | 55935.78 | 55838.03 | 55946.3 | 56321.48 | 56103.53 | 56059.88 | 55404.68 | 55587.43 | 55162.58 | 55255.33 | 55061.53 | 54325.58 | 54884.75 |
| 624.6621 | 55815.95 | 56026.1 | 55669.25 | 55883.53 | 56289.53 | 56123.75 | 55875.98 | 55418.98 | 55536.65 | 55169.65 | 55218.18 | 55110.28 | 54376.68 | 54753.69 |
| 624.7954 | 55660.92 | 55908.98 | 55620.56 | 55869.53 | 56316.33 | 56058.4 | 55871.43 | 55302.3 | 55624 | 55118.85 | 55146.13 | 55208.38 | 54355.21 | 54527.25 |
| 624.9288 | 55686.73 | 55885.58 | 55542.44 | 55789.65 | 56354.55 | 55954.53 | 55800.28 | 55291.55 | 55665.7 | 55150.98 | 55223.35 | 55161.65 | 54295.89 | 54607.06 |
| 625.0621 | 55732.57 | 55842.45 | 55529.34 | 55793.15 | 56329.65 | 55940.7 | 55853.68 | 55398.7 | 55593.73 | 55274.03 | 55085.08 | 55098.03 | 54383.55 | 54927.75 |
| 625.1954 | 55679.78 | 55643.75 | 55574.13 | 55830.13 | 56220.5 | 55811.5 | 55781.65 | 55365.9 | 55685.5 | 55254.88 | 55188.55 | 54985.03 | 54398.32 | 54907.13 |
| 625.3288 | 55708.35 | 55685.78 | 55455.34 | 55727.15 | 56259.8 | 55848.43 | 55787.23 | 55328.58 | 55591.1 | 55117.35 | 55002.25 | 55089.08 | 54363.55 | 54863.38 |
| 625.4622 | 55634.59 | 55782.9 | 55631.91 | 55673.25 | 56204.45 | 55923.1 | 55711 | 55252.38 | 55488.38 | 55181.18 | 54942.95 | 54991.15 | 54287.71 | 54721.5 |
| 625.5955 | 55814.46 | 55770.23 | 55601.03 | 55632.28 | 56231.23 | 55984.28 | 55738.63 | 55182.15 | 55589.68 | 55269.38 | 55047.93 | 55033.68 | 54337.66 | 54694.75 |
| 625.7288 | 55676.95 | 55819.55 | 55494.22 | 55703.25 | 56204.95 | 55916.35 | 55832.38 | 55123.53 | 55531.03 | 55176.78 | 55088.48 | 55093.45 | 54273.63 | 54760.56 |
| 625.8621 | 55533.78 | 55621.33 | 55439.31 | 55569.1 | 56144.85 | 55900.98 | 55737.75 | 55199.68 | 55510.83 | 55219.73 | 55022.78 | 55080.65 | 54219.03 | 54706.5 |
| 625.9955 | 55529.86 | 55518.45 | 55384.03 | 55619.58 | 56027.33 | 55698.4 | 55720.35 | 55223.4 | 55452.6 | 55294.58 | 55053.8 | 55068.33 | 54244.42 | 54583.19 |
| 626.1288 | 55567.14 | 55567.4 | 55343.59 | 55589.3 | 56083.75 | 55852 | 55866.18 | 55198.3 | 55481.88 | 55179.4 | 55068.08 | 55086 | 54273.16 | 54592.63 |
| 626.2622 | 55465.19 | 55719.6 | 55094.31 | 55440 | 55996.9 | 55658.75 | 55730.38 | 55169.55 | 55325.6 | 55064.85 | 55016.55 | 54998.8 | 54270.08 | 54486.13 |
| 626.3955 | 55470.16 | 55518.23 | 55181.56 | 55386.78 | 55834.45 | 55615.28 | 55717.65 | 55195.13 | 55338.58 | 55046.8 | 54948.03 | 54961.8 | 54322.24 | 54433.13 |
| 626.5288 | 55323.92 | 55645.9 | 55246.56 | 55370.53 | 55747.98 | 55687.15 | 55519.43 | 55129.5 | 55292.7 | 55009.3 | 54794.18 | 55047.35 | 54306.29 | 54471.31 |
| 626.6621 | 55274.43 | 55429.9 | 55235.16 | 55325.78 | 55807.35 | 55502.23 | 55458.63 | 55031.58 | 55293.43 | 54885.8 | 54875.65 | 55083.08 | 54264.76 | 54445.44 |
| 626.7954 | 55206.32 | 55500.53 | 55380.25 | 55404.95 | 55722.25 | 55445.15 | 55461.5 | 54936.45 | 55265.58 | 54856.03 | 54885.65 | 54960.98 | 54239.84 | 54424.06 |
| 626.9287 | 55195.95 | 55543.25 | 55205.25 | 55329.43 | 55725.5 | 55424.58 | 55313.53 | 54858 | 55116.38 | 54867.35 | 54791.68 | 54869.23 | 54180.63 | 54330.63 |
| 627.062 | 55060.92 | 55305.9 | 55072.06 | 55237.33 | 55527.7 | 55361.05 | 55305.35 | 54869.73 | 55108.13 | 54848.03 | 54715.43 | 54811.03 | 54204.42 | 54494.5 |
| 627.1953 | 54865.3 | 55273.05 | 54993.69 | 55215.1 | 55412.28 | 55232.08 | 55195.65 | 54851.25 | 55068.83 | 54852.73 | 54701.13 | 54689.38 | 54170.08 | 54458.56 |
| 627.3285 | 54919.92 | 55247.35 | 54892.72 | 55147.8 | 55376.1 | 55254.38 | 55175.18 | 54811.1 | 55018.68 | 54836.98 | 54608.48 | 54589.35 | 54172 | 54305.38 |
| 627.4618 | 55007.16 | 55105 | 54788.44 | 55061.43 | 55302.73 | 55229.55 | 55174.78 | 54750.25 | 54834.43 | 54767.08 | 54586.83 | 54681.65 | 54057.79 | 54352.38 |
| 627.595 | 54894.32 | 55139.43 | 54825.31 | 55057.75 | 55273.23 | 55131.9 | 55200.73 | 54695.75 | 55025.9 | 54760.5 | 54608.15 | 54669.48 | 54066.92 | 54373.75 |
| 627.7283 | 54900.24 | 54960.4 | 54912.41 | 54972.4 | 55262.15 | 55047.8 | 55042.58 | 54632.13 | 54964.15 | 54638.63 | 54622.1 | 54640.73 | 54090.61 | 54297.94 |
| 627.8616 | 54904.7 | 54922.7 | 54901.53 | 54972.98 | 55325.45 | 55097.28 | 55047.38 | 54613.78 | 54839.95 | 54531.1 | 54610.18 | 54480.48 | 54126.87 | 54266.88 |
| 627.9949 | 54808.97 | 54933.13 | 54792.63 | 54887.48 | 55172.28 | 55047 | 55015.58 | 54676.5 | 54788.45 | 54574.68 | 54559.4 | 54483.35 | 54094.39 | 54293.19 |
| 628.1281 | 54767.73 | 54895.83 | 54809.47 | 54885.43 | 55122.05 | 54967.15 | 54880.95 | 54694.48 | 54791.23 | 54543.78 | 54528.68 | 54455.5 | 54067.89 | 54260.13 |
| 628.2614 | 54771.27 | 55007.8 | 54719.91 | 54849.6 | 55016.98 | 54911.1 | 54938.25 | 54713.5 | 54760.3 | 54559.25 | 54423.18 | 54430.85 | 54057.13 | 54237.69 |
| 628.3947 | 54826.97 | 54878.23 | 54682.72 | 54878.65 | 55095.33 | 54791 | 54874.68 | 54687.68 | 54792.48 | 54504.58 | 54354.43 | 54504.6 | 54053.92 | 54182.19 |
| 628.5279 | 54745.24 | 54955.23 | 54678.72 | 54795.15 | 55022.13 | 54782.38 | 54781.88 | 54594.7 | 54741.58 | 54425.8 | 54415.13 | 54456.53 | 54027.05 | 54155.5 |
| 628.6611 | 54716.59 | 54831.48 | 54648.22 | 54788.33 | 54928.93 | 54810.68 | 54883.43 | 54540.18 | 54670.75 | 54435.98 | 54355.68 | 54489.35 | 53987.32 | 54137.63 |
| 628.7944 | 54674.84 | 54801.55 | 54598.19 | 54690.98 | 54976.08 | 54745.15 | 54807.75 | 54523.65 | 54633.1 | 54387.2 | 54318.98 | 54323.18 | 54024.21 | 54196.88 |
| 628.9276 | 54641.16 | 54781.6 | 54565.25 | 54733.78 | 54928.93 | 54737.28 | 54752.25 | 54452.33 | 54526.53 | 54478.8 | 54385.85 | 54350.33 | 53996.47 | 54165.38 |
| 629.0609 | 54584.14 | 54666.33 | 54516.28 | 54726.7 | 54932.73 | 54678.33 | 54711.85 | 54433.15 | 54604.63 | 54469.2 | 54333.45 | 54423.78 | 54018.45 | 54049.69 |
| 629.194 | 54602.97 | 54692.43 | 54595.13 | 54646.2 | 54793.4 | 54658.38 | 54651.45 | 54419.75 | 54488.98 | 54371.53 | 54291.28 | 54372.1 | 54018.34 | 54062.19 |
| 629.3273 | 54619.89 | 54571 | 54438.66 | 54555.93 | 54698.95 | 54734.95 | 54534.18 | 54410 | 54438.33 | 54284.35 | 54294.48 | 54304.6 | 53963.34 | 53977.25 |
| 629.4605 | 54568.92 | 54602.45 | 54375.44 | 54516.8 | 54672.55 | 54716.33 | 54525.43 | 54279.4 | 54468.83 | 54277.28 | 54362.1 | 54267.1 | 53957 | 54070.88 |
| 629.5937 | 54551.62 | 54630.68 | 54406.72 | 54447.58 | 54702.98 | 54556.33 | 54534.55 | 54267.58 | 54406.2 | 54248.45 | 54285.9 | 54297.38 | 53963.39 | 54090.5 |
| 629.7269 | 54594.22 | 54569.85 | 54347.19 | 54446.8 | 54669.85 | 54577.85 | 54513.5 | 54268.35 | 54381.3 | 54300.05 | 54284.78 | 54313.7 | 53938.79 | 54121.5 |
| 629.8601 | 54407.41 | 54513.23 | 54414.34 | 54397.9 | 54664.53 | 54512.8 | 54507.13 | 54204.68 | 54446.93 | 54338.63 | 54221.35 | 54282.1 | 53986.03 | 53944.06 |
| 629.9934 | 54439.73 | 54444.85 | 54288.88 | 54458.83 | 54665.03 | 54489.13 | 54571.05 | 54268.15 | 54387 | 54282 | 54174.85 | 54307.35 | 54033.42 | 53943.63 |
| 630.1265 | 54396.54 | 54401.78 | 54339.13 | 54396.88 | 54588.83 | 54446.75 | 54518.5 | 54240.98 | 54318.43 | 54198.13 | 54177.73 | 54242.68 | 53980.24 | 54076.81 |
| 630.2597 | 54422.16 | 54475.45 | 54337.22 | 54444.55 | 54576.23 | 54514.85 | 54548.1 | 54246.53 | 54338.93 | 54178.43 | 54150.65 | 54247.6 | 53972.58 | 54088 |
| 630.3929 | 54447.68 | 54518.03 | 54294.22 | 54374.33 | 54563.25 | 54506.5 | 54443.4 | 54255.9 | 54366.58 | 54160.38 | 54203.58 | 54207 | 54019.18 | 54015.81 |
| 630.5261 | 54399.08 | 54371.33 | 54300.94 | 54364.78 | 54540.28 | 54519.83 | 54452.75 | 54186.35 | 54376.1 | 54180.9 | 54259.78 | 54178.55 | 53948.5 | 54011.44 |
| 630.6592 | 54397.76 | 54457.4 | 54333.94 | 54415.98 | 54685.95 | 54404.93 | 54412.93 | 54176.63 | 54333.35 | 54222.78 | 54168.08 | 54221.33 | 53900.47 | 53981.44 |
| 630.7924 | 54436.24 | 54453.08 | 54339.38 | 54341.03 | 54605.93 | 54347.15 | 54471.33 | 54140.1 | 54307.9 | 54186.93 | 54160.65 | 54151.95 | 53930.03 | 54073.44 |
| 630.9256 | 54328.41 | 54376.58 | 54325.91 | 54310.88 | 54581.98 | 54353.2 | 54460.63 | 54171.2 | 54214.8 | 54143.2 | 54152.45 | 54127.98 | 53955.24 | 54061.25 |
| 631.0588 | 54275.7 | 54355.6 | 54335.97 | 54237.93 | 54496.35 | 54305.5 | 54404.38 | 54132.95 | 54228.98 | 54137.25 | 54163.75 | 54114.58 | 53913.47 | 54097.5 |
| 631.192 | 54268.43 | 54367.48 | 54281.28 | 54228.48 | 54501.33 | 54340.73 | 54313.18 | 54114.08 | 54259.73 | 54194.58 | 54136.98 | 54080.68 | 53947.63 | 53951.5 |
| 631.3251 | 54251.24 | 54291.93 | 54314.44 | 54294.28 | 54495.55 | 54368.43 | 54251.2 | 54151.48 | 54217.08 | 54148.2 | 54099.18 | 54115.15 | 53945.63 | 53998.44 |
| 631.4583 | 54253.73 | 54306.15 | 54279.31 | 54262.2 | 54462.73 | 54429.28 | 54261.8 | 54106.55 | 54192.45 | 54107.33 | 54120.45 | 54110.58 | 53948.05 | 53933.44 |
| 631.5914 | 54224.11 | 54289.15 | 54305.13 | 54337.3 | 54427.23 | 54366.53 | 54269.38 | 54164.85 | 54205.65 | 54109.08 | 54116.48 | 54126.93 | 53923.47 | 53956.5 |
| 631.7246 | 54204.35 | 54289.4 | 54252.88 | 54393.18 | 54455.15 | 54340.78 | 54313.95 | 54102.8 | 54225.45 | 54107.03 | 54059.65 | 54118.43 | 53916.37 | 53937.63 |
| 631.8577 | 54257.08 | 54273.98 | 54244.91 | 54324.08 | 54462.68 | 54355.63 | 54262.53 | 54081.8 | 54215.15 | 54116.25 | 54088.58 | 54101.18 | 53915.61 | 53963.06 |
| 631.9908 | 54196.51 | 54247.9 | 54192.31 | 54212 | 54392.2 | 54315.25 | 54269.28 | 54063.73 | 54173.95 | 54084.95 | 54041.23 | 54053.55 | 53909.63 | 53916.88 |
| 632.124 | 54269 | 54194.65 | 54155.59 | 54273.7 | 54353.08 | 54392.93 | 54252.88 | 54106.25 | 54195.63 | 54082.95 | 54092.03 | 54051.73 | 53896.82 | 53948.5 |
| 632.2571 | 54234.76 | 54215.08 | 54156.56 | 54234.23 | 54341.95 | 54361.8 | 54266.73 | 54039.83 | 54194.75 | 54078.03 | 54092.88 | 54056.38 | 53965.66 | 53977.06 |
| 632.3902 | 54262.38 | 54216.9 | 54172.56 | 54173.83 | 54360.95 | 54343.85 | 54185.35 | 54101.35 | 54203.95 | 54058.83 | 54066.7 | 54089.7 | 53927.11 | 53975.44 |
| 632.5233 | 54178.86 | 54220.65 | 54284.41 | 54181.83 | 54327.5 | 54313.93 | 54196.83 | 54121.13 | 54157.03 | 54051.35 | 54092.4 | 54091.28 | 53904.84 | 54054 |
| 632.6564 | 54105.32 | 54205.6 | 54310.91 | 54208.78 | 54336.48 | 54415.48 | 54223.75 | 54115.18 | 54238.43 | 54053.33 | 54113.35 | 54101.3 | 53890.82 | 54093.75 |
| 632.7896 | 54170.76 | 54227.9 | 54195.28 | 54232.1 | 54316.55 | 54395.98 | 54258.08 | 54112.15 | 54225.93 | 53956.25 | 54085.05 | 54077.05 | 53897.97 | 54041.38 |
| 632.9227 | 54190.51 | 54277.9 | 54237.31 | 54293.3 | 54356.85 | 54302.45 | 54322.75 | 54094.98 | 54115.2 | 53969.98 | 54097.28 | 54050.63 | 53897.45 | 54020.13 |
| 633.0558 | 54182.08 | 54217.85 | 54112.28 | 54291.25 | 54297.43 | 54252.73 | 54252.1 | 54077.03 | 54250.55 | 54010.78 | 54044.25 | 54094.4 | 53862.18 | 53950.94 |
| 633.1889 | 54143.95 | 54215.05 | 54144.5 | 54253.45 | 54289.15 | 54333.25 | 54222.18 | 54092.93 | 54159.33 | 54078.08 | 54036.6 | 54079.73 | 53907.39 | 53901.25 |
| 633.322 | 54153.22 | 54211.18 | 54181.13 | 54238.08 | 54333.65 | 54273.83 | 54228.28 | 54125.48 | 54153.43 | 54017.55 | 54021.03 | 54043.78 | 53880.55 | 53959.13 |
| 633.4551 | 54195.73 | 54203.73 | 54125.28 | 54241.93 | 54242.75 | 54301.48 | 54225.7 | 54073.03 | 54180.7 | 54031.95 | 54052.6 | 54063.53 | 53876.84 | 53988.75 |
| 633.5881 | 54194.81 | 54230.03 | 54123.13 | 54271.38 | 54192.73 | 54306.05 | 54273.93 | 54046.83 | 54170.6 | 54017.05 | 54052.38 | 54018.7 | 53907.34 | 54027.25 |
| 633.7213 | 54180 | 54252.03 | 54144.41 | 54282.2 | 54312.98 | 54282.15 | 54213.35 | 54003.98 | 54229 | 54102 | 54043.13 | 54052.43 | 53925.34 | 54047.81 |
| 633.8543 | 54171.22 | 54187.13 | 54204.31 | 54186.88 | 54330.25 | 54158.58 | 54202.88 | 53985 | 54201.25 | 54123.25 | 54043.65 | 54048.08 | 53888.26 | 54009.38 |
| 633.9874 | 54120.27 | 54128.85 | 54178.59 | 54210.18 | 54299.5 | 54201.78 | 54106.75 | 54041.83 | 54202.68 | 54050.83 | 54019.6 | 54107.65 | 53925.18 | 53926.94 |
| 634.1204 | 54184.3 | 54102.08 | 54099.69 | 54224.23 | 54337.2 | 54260.3 | 54147.55 | 54044.3 | 54195.63 | 54032.85 | 54050.78 | 54065.23 | 53920.45 | 53952.38 |
| 634.2535 | 54210.51 | 54114.43 | 54133.31 | 54197.8 | 54292.05 | 54310.53 | 54132.05 | 54116.98 | 54105.08 | 54026 | 54098.4 | 54038.25 | 53894.79 | 53980.31 |
| 634.3866 | 54179.86 | 54098.15 | 54136.75 | 54160.4 | 54363.55 | 54251.23 | 54138.7 | 54049.95 | 54158.68 | 54047.83 | 54119.68 | 54018.53 | 53906.11 | 54041.5 |
| 634.5197 | 54195.11 | 54148.38 | 54109.91 | 54155.95 | 54313.35 | 54193.23 | 54268.28 | 54008.58 | 54173.88 | 54010.65 | 54087.23 | 54066.15 | 53912 | 53963.44 |
| 634.6527 | 54159.32 | 54232.33 | 54067.41 | 54202.18 | 54234.15 | 54223.33 | 54207.5 | 53995.45 | 54112.25 | 53996.75 | 54062.3 | 54053.95 | 53924.39 | 54027.81 |
| 634.7857 | 54170.81 | 54178.53 | 54100.88 | 54205.93 | 54274.9 | 54184.43 | 54209.1 | 54005 | 54138.75 | 54003.98 | 54106.8 | 54042.1 | 53926.24 | 53994.25 |
| 634.9188 | 54177.24 | 54197.13 | 54070.53 | 54147.33 | 54311.48 | 54229.05 | 54169.13 | 54044.4 | 54105.3 | 53990.9 | 54032.68 | 54054.48 | 53883.79 | 53907.88 |
| 635.0518 | 54141.57 | 54229.63 | 54077.84 | 54122.4 | 54303.1 | 54208.53 | 54180.35 | 54030.4 | 54104.95 | 53996.13 | 53998.73 | 54123.95 | 53896.55 | 53925.31 |
| 635.1849 | 54124.97 | 54249.63 | 54122 | 54172.25 | 54249.68 | 54150.08 | 54132.63 | 54073.63 | 54135.68 | 53975.35 | 53992.45 | 54053.48 | 53878.26 | 53998.5 |
| 635.3179 | 54153.27 | 54226.28 | 54105.97 | 54223.18 | 54249 | 54159.48 | 54132.53 | 54111.73 | 54118.08 | 54015.25 | 54064.18 | 54022.45 | 53863.05 | 53951.75 |
| 635.4509 | 54149.51 | 54182.65 | 54058.03 | 54212.33 | 54384.1 | 54222.23 | 54161.9 | 54028.83 | 54110.93 | 54037.2 | 54069 | 53998.13 | 53841.29 | 53973.13 |
| 635.5839 | 54175 | 54195.55 | 54034.56 | 54293.63 | 54286.2 | 54191.7 | 54139.38 | 54059.05 | 54132.7 | 54068.78 | 54059.2 | 54057.45 | 53894.95 | 53953.25 |
| 635.7169 | 54139.16 | 54140.33 | 54083.94 | 54197.45 | 54254.18 | 54209.28 | 54155.78 | 54062.85 | 54120.43 | 54055.63 | 54054.05 | 54011.43 | 53861.79 | 54009 |
| 635.85 | 54090.95 | 54135.55 | 54147.38 | 54246.43 | 54323.75 | 54229.63 | 54126.78 | 54023.8 | 54112.6 | 54040.73 | 54026.23 | 54030.73 | 53864.47 | 54019.56 |
| 635.983 | 54131.76 | 54172.38 | 54128.75 | 54150.75 | 54275.43 | 54193.23 | 54165.1 | 54032.15 | 54146.4 | 54057.28 | 54060.55 | 54026.18 | 53846.89 | 54028.81 |
| 636.116 | 54135.43 | 54169.4 | 54090 | 54136.58 | 54308.7 | 54164.1 | 54181.2 | 54026.13 | 54211.5 | 54014.5 | 54091.15 | 54042.93 | 53878.74 | 54001.56 |
| 636.249 | 54081.05 | 54215.2 | 54098.72 | 54140.7 | 54296.45 | 54227.63 | 54123.18 | 54035.75 | 54179.05 | 54038.25 | 53976.53 | 54121.58 | 53894.92 | 53971.88 |
| 636.382 | 54141.62 | 54218.18 | 54156.72 | 54157.25 | 54420.35 | 54262.75 | 54193.73 | 54052.65 | 54224.93 | 54113.68 | 54018.05 | 54089.3 | 53917.24 | 54022.38 |
| 636.515 | 54190.76 | 54238.18 | 54238.69 | 54201.85 | 54443.25 | 54295.7 | 54125.88 | 54004.03 | 54159.5 | 54064.93 | 54069.6 | 54088.18 | 53880.16 | 54031.13 |
| 636.648 | 54163.59 | 54194.9 | 54127.63 | 54243.08 | 54316.63 | 54226.58 | 54148.9 | 54060.6 | 54149.75 | 54007.9 | 54041.35 | 54068 | 53877.89 | 53981.38 |
| 636.7809 | 54191.76 | 54228.88 | 54108.63 | 54169.65 | 54345.18 | 54178.2 | 54130.25 | 54036.3 | 54139.9 | 54109.98 | 54072.65 | 54057.83 | 53868.32 | 54014.13 |
| 636.9139 | 54201.03 | 54199.25 | 54118.31 | 54246.9 | 54367.58 | 54191.63 | 54148.73 | 53998.18 | 54095.15 | 54085.88 | 54063.38 | 54118.85 | 53891.92 | 53976 |
| 637.0469 | 54122.05 | 54267 | 54188.22 | 54232.58 | 54345.85 | 54230.58 | 54151.28 | 54039.33 | 54105.68 | 54056.1 | 54055.58 | 54096.43 | 53887.24 | 53977.69 |
| 637.1799 | 54144.86 | 54238.58 | 54063.56 | 54143.9 | 54315.43 | 54184.6 | 54203.78 | 54025.08 | 54113.15 | 54073.48 | 54039.7 | 54030.3 | 53864.47 | 53972.75 |
| 637.3128 | 54208.38 | 54196.45 | 54036.22 | 54133.88 | 54271.5 | 54203.3 | 54158.58 | 54001.25 | 54162.48 | 54020.78 | 54066.78 | 54044.23 | 53890.95 | 54000 |
| 637.4458 | 54242.73 | 54169.98 | 54078.41 | 54130.2 | 54200.98 | 54204.15 | 54205.5 | 54016.38 | 54177.75 | 54027.18 | 54080.8 | 54018.13 | 53907.08 | 53974.31 |
| 637.5787 | 54171.84 | 54221.05 | 54041.88 | 54218.43 | 54252.9 | 54206.3 | 54219.8 | 54014.15 | 54165.4 | 54006.75 | 54088.13 | 54069.83 | 53884.08 | 53924.06 |
| 637.7117 | 54263.89 | 54232.13 | 54068.5 | 54258.2 | 54359.38 | 54207.95 | 54203.48 | 54011.48 | 54139.15 | 54065.8 | 54064.93 | 54125.88 | 53875.71 | 54024.06 |
| 637.8446 | 54249.43 | 54481.6 | 54130.78 | 54272.43 | 54427.58 | 54165.25 | 54278.35 | 54018.6 | 54170.13 | 54012.75 | 54078.53 | 54067.25 | 53866.21 | 53919.31 |
| 637.9776 | 54325.62 | 54359.75 | 54175.53 | 54249.63 | 54411.53 | 54219.1 | 54292.18 | 54047.1 | 54150.33 | 54055.35 | 54002.23 | 54083.23 | 53879.97 | 53953.38 |
| 638.1105 | 54316.81 | 54242 | 54185.56 | 54283.2 | 54375 | 54245.15 | 54280.25 | 54056.35 | 54196.5 | 54069.33 | 54074.38 | 54119.6 | 53950.61 | 53973.75 |
| 638.2435 | 54244.65 | 54185.85 | 54175.41 | 54221.43 | 54399.25 | 54242.28 | 54253.28 | 54040.98 | 54185.7 | 54080.93 | 54103.95 | 54077.78 | 53896.87 | 53947 |
| 638.3764 | 54194.19 | 54194.68 | 54128.06 | 54174.05 | 54393.25 | 54245.18 | 54307.75 | 54073.45 | 54205.4 | 54035.95 | 54068.93 | 54042.73 | 53864.42 | 53995.94 |
| 638.5093 | 54289.14 | 54209.85 | 54064.31 | 54342.08 | 54277.65 | 54205.08 | 54208.25 | 54048.38 | 54125.68 | 54041.3 | 54099.48 | 54060.83 | 53928.61 | 53961.13 |
| 638.6422 | 54261.78 | 54236.73 | 54107.69 | 54263.08 | 54307.93 | 54198.83 | 54181 | 54078.65 | 54159.48 | 54058.68 | 54075.6 | 54053.88 | 53937.68 | 53927 |
| 638.7752 | 54187.11 | 54231.13 | 54083.06 | 54185.48 | 54347.48 | 54212.63 | 54225.4 | 54050.75 | 54147.75 | 54086.45 | 54055.18 | 54065.85 | 53908.55 | 53962.44 |
| 638.9081 | 54199.84 | 54204.25 | 54063.38 | 54234.1 | 54371.88 | 54261.33 | 54355.65 | 54099.9 | 54155.63 | 54045.38 | 54102.6 | 54098.33 | 53954 | 53973.81 |
| 639.041 | 54143.7 | 54260.35 | 54043.22 | 54280.63 | 54361.8 | 54238.2 | 54278.45 | 54102.25 | 54099.73 | 54044.75 | 54062.6 | 54063.6 | 53947.34 | 54030.13 |
| 639.1739 | 54187.41 | 54211.43 | 54080.28 | 54268.6 | 54307.98 | 54188.5 | 54288.93 | 54069.33 | 54168.08 | 54108.18 | 54086 | 54097.03 | 53948.11 | 53939.94 |
| 639.3068 | 54211.05 | 54278.1 | 54168.56 | 54201.18 | 54250.65 | 54226.83 | 54273.2 | 54007.08 | 54164.18 | 54120.38 | 54088.08 | 54044.4 | 53896.03 | 53912 |
| 639.4396 | 54157.54 | 54239.1 | 54099.88 | 54280.53 | 54350.2 | 54220.05 | 54206.23 | 54045.75 | 54097.1 | 54130.33 | 54018.35 | 54078.33 | 53918.26 | 53912.63 |
| 639.5726 | 54230.62 | 54230.2 | 54140.56 | 54233.5 | 54324.98 | 54148.2 | 54167.3 | 54085.93 | 54157.63 | 54076.58 | 53999.18 | 54046.03 | 53892.92 | 53943.38 |
| 639.7054 | 54222.41 | 54213.53 | 54176.47 | 54249.83 | 54357.73 | 54313 | 54178.2 | 54027.73 | 54191.85 | 54116.05 | 54019.98 | 54044.68 | 53892.16 | 53992.13 |
| 639.8383 | 54259 | 54207.05 | 54162.94 | 54252.55 | 54335.7 | 54350.28 | 54177.98 | 54080.68 | 54100.23 | 54059.88 | 54024.55 | 54033.93 | 53886.97 | 54003.5 |
| 639.9712 | 54240.92 | 54273.88 | 54196.09 | 54259.23 | 54420.93 | 54320.18 | 54181.78 | 54098.2 | 54079.63 | 54072.73 | 54039.78 | 54141.13 | 53972.13 | 53982 |
| 640.1041 | 54150 | 54244.45 | 54180.16 | 54252.7 | 54418.2 | 54329.9 | 54254.53 | 54101.5 | 54087.7 | 54073 | 54045.15 | 54140.75 | 53918.82 | 54031.81 |
| 640.2369 | 54143.84 | 54271.08 | 54091.28 | 54224.88 | 54329.23 | 54285.5 | 54253.05 | 54121.83 | 54152.98 | 54079.85 | 54039.98 | 54104.18 | 53908.37 | 54093.06 |
| 640.3698 | 54208 | 54262.5 | 54098.91 | 54208.8 | 54328.45 | 54219.28 | 54231.9 | 54101.9 | 54209.23 | 54130.43 | 54055.85 | 54067.8 | 53883.11 | 53969.56 |
| 640.5027 | 54207.62 | 54278.43 | 54092.13 | 54224.83 | 54301.13 | 54237.18 | 54244.65 | 54109.88 | 54201.8 | 54069.28 | 54041.53 | 54055.08 | 53877.97 | 53963 |
| 640.6355 | 54291.51 | 54354.48 | 54124.19 | 54230.7 | 54234.5 | 54266.38 | 54293.68 | 54106.05 | 54157.23 | 54104.6 | 54053.38 | 54029.35 | 53889.58 | 54020.88 |
| 640.7684 | 54260.46 | 54304.7 | 54226.81 | 54187.38 | 54232.88 | 54248.65 | 54188.75 | 54055.68 | 54171.78 | 54142.38 | 54032.73 | 54069.08 | 53947.16 | 54111.69 |
| 640.9012 | 54218.22 | 54241.68 | 54192.16 | 54253.8 | 54300.18 | 54248.63 | 54171.75 | 54105.98 | 54215.23 | 54053.13 | 54070.2 | 54101.5 | 53943.18 | 54023 |
| 641.0341 | 54168.89 | 54236.93 | 54184.16 | 54304.38 | 54359.68 | 54241.6 | 54262.35 | 54052.58 | 54216.55 | 54100.9 | 54124.5 | 54102.2 | 53914.74 | 53966.75 |
| 641.1669 | 54280.24 | 54297.83 | 54112.78 | 54252.48 | 54294.48 | 54221.83 | 54244.98 | 54083.25 | 54284 | 54063.43 | 54104.75 | 54096.18 | 53903.76 | 53984.94 |
| 641.2997 | 54207.78 | 54314.65 | 54167.38 | 54227.38 | 54324.18 | 54212.98 | 54238.7 | 54061.3 | 54200.4 | 54074.68 | 54165.58 | 54105.65 | 53906.97 | 53907.56 |
| 641.4326 | 54313.92 | 54243.45 | 54180.56 | 54212.08 | 54381.9 | 54241.58 | 54184.93 | 54090.3 | 54228.18 | 54037.23 | 54130.65 | 54098.93 | 53890.21 | 53942.25 |
| 641.5654 | 54296.81 | 54217.28 | 54211 | 54178 | 54386.2 | 54225.15 | 54189 | 54160.08 | 54335.63 | 54077.95 | 54084 | 54073.63 | 53958.05 | 53987.25 |
| 641.6982 | 54269.14 | 54211.33 | 54153.94 | 54230.9 | 54396.55 | 54261.75 | 54157.38 | 54154.73 | 54197.98 | 54045.88 | 54038.65 | 54067.83 | 53976.42 | 54010.06 |
| 641.831 | 54133.35 | 54253.13 | 54103.69 | 54230.65 | 54431.2 | 54255.08 | 54233.25 | 54150.23 | 54201.28 | 54077.35 | 54067.33 | 54130.35 | 53972.16 | 53962 |
| 641.9638 | 54133.65 | 54263.3 | 54112.91 | 54251.35 | 54450.75 | 54224.18 | 54242.9 | 54104.03 | 54266.53 | 54079.6 | 54115.08 | 54104.58 | 53994.95 | 53971.31 |
| 642.0966 | 54197.95 | 54281.5 | 54163.31 | 54206.05 | 54391.48 | 54217.8 | 54269.85 | 54070.25 | 54169.63 | 54083.9 | 54083.15 | 54056 | 53955.97 | 53957.06 |
| 642.2294 | 54164.38 | 54196.13 | 54182.22 | 54225.23 | 54356.38 | 54259.83 | 54274.1 | 54101 | 54222.65 | 54132.2 | 54080.6 | 54096.55 | 53896.34 | 53933.19 |
| 642.3622 | 54246 | 54273.9 | 54151.16 | 54309.18 | 54362.45 | 54217.1 | 54261.5 | 54044.63 | 54275.85 | 54105.15 | 54074.53 | 54095.65 | 53932.03 | 54018.69 |
| 642.495 | 54294.76 | 54269.4 | 54123.72 | 54308.78 | 54344.63 | 54241.08 | 54254.68 | 54082.55 | 54209.23 | 54059.38 | 54091.4 | 54146.13 | 53913.32 | 54047.31 |
| 642.6278 | 54281.59 | 54250.6 | 54153.97 | 54297.5 | 54317.15 | 54236.2 | 54191 | 54083.53 | 54188.23 | 54089.83 | 54098.78 | 54171.03 | 53911.5 | 53988.63 |
| 642.7606 | 54323.27 | 54216.63 | 54203.16 | 54325.6 | 54360.38 | 54231.7 | 54271.13 | 54106.75 | 54169.5 | 54087.53 | 54100.95 | 54128.35 | 53918.74 | 53985.19 |
| 642.8934 | 54295.11 | 54202.78 | 54220.47 | 54277.68 | 54280.43 | 54293.78 | 54250.95 | 54076.33 | 54166.2 | 54053.78 | 54098.78 | 54095.78 | 53891 | 54032.63 |
| 643.0261 | 54210.43 | 54219.83 | 54200.06 | 54296.1 | 54287.65 | 54233 | 54222.73 | 54057.43 | 54275.33 | 54100.88 | 54088.1 | 54116.78 | 53899.05 | 54000.44 |
| 643.1589 | 54231.03 | 54225.43 | 54203.44 | 54309.05 | 54379.45 | 54270.13 | 54303.83 | 54048.93 | 54268.75 | 54085.23 | 54132.55 | 54094.9 | 53904.32 | 53933.19 |
| 643.2917 | 54172.41 | 54166.73 | 54162.69 | 54364.78 | 54338.3 | 54303.2 | 54297.5 | 54104.45 | 54204.03 | 54077.85 | 54107.95 | 54080.83 | 53912.24 | 53984.44 |
| 643.4244 | 54177.7 | 54201.6 | 54255.59 | 54314.38 | 54319.1 | 54265.7 | 54243.03 | 54111.28 | 54156 | 54103.85 | 54131.73 | 54082.45 | 53908.68 | 53964.44 |
| 643.5572 | 54236.38 | 54220.98 | 54330.94 | 54328.33 | 54323.88 | 54248.83 | 54257.75 | 54095.9 | 54130.53 | 54086.85 | 54070.28 | 54096.45 | 53907.21 | 53945.13 |
| 643.6899 | 54196.84 | 54195.55 | 54206.91 | 54287.88 | 54310.1 | 54213.58 | 54298.6 | 54100.3 | 54060.78 | 54064.75 | 54086.2 | 54090.3 | 53946.32 | 54002.56 |
| 643.8227 | 54248.38 | 54199.83 | 54194.41 | 54300.35 | 54363.63 | 54298.48 | 54242.18 | 54083.55 | 54084.93 | 54110.18 | 54099.75 | 54140.83 | 53911.61 | 53952.44 |
| 643.9554 | 54230.43 | 54169.23 | 54254.34 | 54246.7 | 54358.65 | 54273.93 | 54330.25 | 54070.3 | 54116.03 | 54148.93 | 54088.78 | 54076.75 | 53926.95 | 53957.31 |
| 644.0882 | 54225.54 | 54232.13 | 54227.78 | 54255.15 | 54317.65 | 54284.4 | 54342.9 | 54085.6 | 54168.6 | 54111.75 | 54113.13 | 54104.13 | 53906.84 | 54046 |
| 644.2209 | 54221.59 | 54201.38 | 54259.03 | 54311.35 | 54342.93 | 54296.6 | 54288.78 | 54097.4 | 54180.93 | 54073.6 | 54141.43 | 54084.3 | 53960.63 | 53966 |
| 644.3536 | 54243.43 | 54294.18 | 54231.13 | 54372.83 | 54313.85 | 54220.5 | 54173.33 | 54013.75 | 54160.5 | 54071.03 | 54177.65 | 54086.25 | 53946.29 | 53939.31 |
| 644.4863 | 54179.95 | 54226.05 | 54186.63 | 54264.3 | 54333.18 | 54197.8 | 54240.78 | 54052.98 | 54116.55 | 54116.03 | 54116.13 | 54198.05 | 53919.18 | 53966.81 |
| 644.6191 | 54137.89 | 54233.65 | 54222.13 | 54274.25 | 54383.28 | 54182.33 | 54204.23 | 54076 | 54175.58 | 54072.58 | 54135.48 | 54187.58 | 53901.55 | 53999.19 |
| 644.7518 | 54173.65 | 54194.38 | 54194.47 | 54348.8 | 54368.55 | 54230 | 54283.28 | 54148 | 54185.85 | 54065.7 | 54083.35 | 54189.58 | 53907.47 | 54038.25 |
| 644.8845 | 54176.41 | 54241 | 54223.66 | 54306.98 | 54366.08 | 54231.3 | 54315.33 | 54107.03 | 54196.8 | 54037.48 | 54072.2 | 54117.83 | 53921.03 | 53962.63 |
| 645.0172 | 54158.62 | 54215.38 | 54146.72 | 54315.5 | 54382.08 | 54301 | 54221.9 | 54086.3 | 54155.08 | 54043.98 | 54066.35 | 54082.28 | 53900.26 | 53970.63 |
| 645.1499 | 54140.7 | 54234.98 | 54150.69 | 54247.08 | 54359.73 | 54304.88 | 54175.98 | 54115.48 | 54097.65 | 54080.4 | 54053.75 | 54088.9 | 53899.05 | 53952.5 |
| 645.2826 | 54190.11 | 54202.98 | 54177.69 | 54264.25 | 54363.2 | 54313.98 | 54210.33 | 54045.35 | 54148.13 | 54053.65 | 54105.88 | 54078.5 | 53866.03 | 53938.38 |
| 645.4153 | 54230.19 | 54224.9 | 54114.03 | 54240.85 | 54300.78 | 54303.6 | 54240.08 | 54085.78 | 54186.98 | 54077.03 | 54136.38 | 54117.5 | 53850.61 | 54087.44 |
| 645.548 | 54189.65 | 54316.13 | 54072.88 | 54275.18 | 54358.95 | 54251.93 | 54271.2 | 54108.95 | 54289.75 | 54077.43 | 54102.58 | 54113.03 | 53840.18 | 54056.56 |
| 645.6807 | 54216.35 | 54305.38 | 54070.41 | 54254.1 | 54297.63 | 54224.23 | 54228.53 | 54072.9 | 54186.13 | 54020.65 | 54088.7 | 54045.73 | 53839.84 | 54037.75 |
| 645.8134 | 54208.46 | 54282.03 | 54073.94 | 54178.9 | 54288 | 54177.1 | 54233.35 | 54121.88 | 54139.58 | 54040.2 | 54083.78 | 54084.3 | 53828.66 | 54139.56 |
| 645.946 | 54246.08 | 54228.73 | 54084.59 | 54157.63 | 54248.08 | 54245.2 | 54277 | 54133.48 | 54168.83 | 54095.18 | 54031.05 | 54080.75 | 53889.76 | 54052.31 |
| 646.0787 | 54256.27 | 54258.65 | 54100.28 | 54204.1 | 54286.95 | 54218.75 | 54318.88 | 54201.48 | 54182.18 | 54052.63 | 54032.95 | 54063.3 | 53887.08 | 54050.25 |
| 646.2114 | 54241.62 | 54328.53 | 54139.56 | 54157.38 | 54318.6 | 54208.15 | 54271.18 | 54110.45 | 54100.1 | 54124.85 | 54023.43 | 54072.05 | 53895.39 | 54018.44 |
| 646.344 | 54221.54 | 54213.3 | 54089.69 | 54155.75 | 54403.23 | 54214.55 | 54223.73 | 54071.7 | 54097.2 | 54070 | 54084.55 | 54126.53 | 53929.53 | 54097.75 |
| 646.4767 | 54249.65 | 54202.33 | 54122.13 | 54180.63 | 54304.73 | 54273 | 54211.7 | 54043.85 | 54113.3 | 54064.83 | 54040.95 | 54100.83 | 53913.68 | 54009.06 |
| 646.6093 | 54184.76 | 54200.08 | 54105.16 | 54212.08 | 54384.7 | 54223.13 | 54181.15 | 54055.23 | 54122.05 | 54019.45 | 54039.13 | 54085.63 | 53865.16 | 54010.5 |
| 646.742 | 54174.54 | 54192.28 | 54109 | 54255.93 | 54448.18 | 54198.88 | 54158.73 | 54090.93 | 54168.93 | 54045.78 | 54008.48 | 54045.78 | 53910.53 | 54027.38 |
| 646.8746 | 54149.97 | 54184 | 54128.41 | 54225.15 | 54394.4 | 54270.75 | 54155.95 | 54093.25 | 54173.93 | 54049 | 54047.78 | 54044.68 | 53916.42 | 54093.38 |
| 647.0073 | 54158.05 | 54262.23 | 54102.22 | 54215.85 | 54345.58 | 54224.2 | 54231.85 | 54103.83 | 54221.13 | 54024.18 | 54073.33 | 54048.95 | 53912.61 | 54004.63 |
| 647.1399 | 54234.41 | 54248.78 | 54147.19 | 54200.13 | 54332.58 | 54272.73 | 54189.75 | 54119.38 | 54241.6 | 54047.85 | 54075.28 | 54093.08 | 53922.97 | 53991.75 |
| 647.2725 | 54152.11 | 54191.58 | 54188.91 | 54242.75 | 54305.25 | 54221.08 | 54207.83 | 54113.3 | 54217.93 | 54073.83 | 54033.88 | 54110.43 | 53909.55 | 54023.38 |
| 647.4052 | 54201.7 | 54179.15 | 54120.25 | 54210.35 | 54319.65 | 54202.98 | 54237.25 | 54111.83 | 54114.43 | 54102.03 | 54017.63 | 54002.9 | 53898.16 | 53959.38 |
| 647.5378 | 54106.27 | 54235.13 | 54066.09 | 54225 | 54277.3 | 54188.8 | 54229.88 | 54020.03 | 54153.88 | 54083.3 | 54009.85 | 54055.98 | 53899.42 | 53948.44 |
| 647.6704 | 54062.03 | 54206.98 | 54063 | 54279.1 | 54189.93 | 54187.63 | 54212.78 | 54006.83 | 54118.38 | 54046.45 | 54041.65 | 54147.45 | 53893.47 | 54024.06 |
| 647.803 | 54122.97 | 54221.35 | 54150.44 | 54262.2 | 54174.03 | 54195.68 | 54151.7 | 54053.83 | 54083.9 | 54021.35 | 54055.93 | 54097.68 | 53912.74 | 53986.44 |
| 647.9356 | 54068.3 | 54213.4 | 54097.06 | 54165.13 | 54222.3 | 54156.55 | 54157.35 | 54094.1 | 54088.23 | 54014.9 | 54029.15 | 54135.7 | 53910.16 | 53978.69 |
| 648.0682 | 54068.43 | 54174.03 | 54049.97 | 54141.75 | 54272.68 | 54151.58 | 54110.05 | 54077.05 | 54108.78 | 54072.28 | 54061.6 | 54049.45 | 53890.76 | 53978.81 |
| 648.2008 | 54119.57 | 54169 | 54074.53 | 54120.88 | 54261.13 | 54167.65 | 54175.7 | 54087.1 | 54092.15 | 54056.7 | 54059.78 | 54074.1 | 53919.5 | 53961.25 |
| 648.3334 | 54149.68 | 54139.63 | 54040.47 | 54252.63 | 54259.48 | 54171.48 | 54237.18 | 54010.95 | 54115.3 | 54071.23 | 54010.6 | 54060.98 | 53903.29 | 53976.75 |
| 648.4659 | 54113.03 | 54131.33 | 54065.38 | 54226.33 | 54344.5 | 54180.3 | 54234.18 | 53972.65 | 54080.78 | 53992.8 | 54021.18 | 54110 | 53855.16 | 54003.88 |
| 648.5986 | 54073.3 | 54062.65 | 54046.5 | 54198.95 | 54340.85 | 54125.53 | 54226.98 | 53952.13 | 54074.28 | 53995.13 | 54028.6 | 54081.35 | 53865.92 | 53943.5 |
| 648.7311 | 54092.22 | 54069.35 | 54007.41 | 54147.43 | 54234.25 | 54220.85 | 54164.18 | 54018.88 | 54181.2 | 54027.6 | 54036.15 | 54048.35 | 53894.34 | 53920.88 |
| 648.8637 | 54064.41 | 54094.1 | 54086.59 | 54133.33 | 54178.9 | 54155.63 | 54182.1 | 54000.35 | 54095.85 | 54028.95 | 54009.7 | 54039.3 | 53891.89 | 53942 |
| 648.9963 | 54030.62 | 54124.98 | 54086.66 | 54136.3 | 54212.98 | 54100.13 | 54135.73 | 54032.8 | 54088.18 | 54020.58 | 54040.55 | 54025.15 | 53898.34 | 53869.06 |
| 649.1289 | 54054 | 54099.35 | 54068.63 | 54043.2 | 54223.23 | 54128.85 | 54106.1 | 53951.23 | 54120.25 | 53998.78 | 54031.88 | 54079.08 | 53897.13 | 53845.69 |
| 649.2614 | 54074.38 | 54143.08 | 54052.72 | 54075.55 | 54241.1 | 54085.85 | 54099.88 | 53993.7 | 54085.6 | 53986.43 | 53983.48 | 54068.1 | 53878.21 | 53937.19 |
| 649.3939 | 54075.92 | 54164.53 | 54095.63 | 54066.73 | 54225.7 | 54105.43 | 54058.25 | 53945.55 | 54043.18 | 54010.13 | 53977.5 | 54013.75 | 53848.55 | 53941.19 |
| 649.5265 | 54110.68 | 54160.38 | 54060.69 | 54066.08 | 54253.08 | 54117.2 | 54104.13 | 53922.43 | 54079.65 | 54001.08 | 54033.65 | 54013.05 | 53884.26 | 53932.94 |
| 649.6591 | 54134.62 | 54118.13 | 54067.41 | 54066.1 | 54190.05 | 54162.95 | 54098.9 | 53964.5 | 54110.7 | 53947.25 | 53991.75 | 53952.78 | 53910.55 | 53953.44 |
| 649.7916 | 54092.95 | 54093.93 | 54081.72 | 54128.45 | 54260.53 | 54114.43 | 54038.9 | 54010.5 | 54082.88 | 53979.3 | 54043.43 | 54018.95 | 53859.08 | 53967.81 |
| 649.9241 | 54149.81 | 54163.2 | 54044.88 | 54079.8 | 54222.33 | 54121.3 | 54073.7 | 53952.78 | 54046.18 | 53946.48 | 53996.05 | 54018.15 | 53858.39 | 53965.5 |
| 650.0566 | 54123.38 | 54149.5 | 54093.53 | 54068.15 | 54211.15 | 54166.85 | 54139.93 | 53992.75 | 54013.68 | 53934.75 | 53965.83 | 53982.9 | 53835.71 | 53923.31 |
| 650.1892 | 54084.41 | 54155.15 | 54014.25 | 54088.88 | 54310.88 | 54205.13 | 54094.33 | 53977.05 | 54099.43 | 53951.4 | 53978.7 | 53995.73 | 53815.97 | 53892.63 |
| 650.3217 | 54065.08 | 54100.7 | 54012.41 | 54117.78 | 54248.15 | 54190.05 | 54113.55 | 53914.28 | 54074.35 | 53979 | 53951.9 | 53986 | 53911.03 | 53870.5 |
| 650.4542 | 54066.16 | 54104 | 53986 | 54095.95 | 54218.78 | 54151.28 | 54132.25 | 53927.05 | 54103.45 | 53963.38 | 54021.38 | 53985.8 | 53931.05 | 53905 |
| 650.5867 | 54051.27 | 54101.25 | 53986.91 | 54075.88 | 54154.45 | 54107.08 | 54102.78 | 53935.6 | 54059.48 | 53974.55 | 54014.4 | 54020 | 53896.84 | 53913.13 |
| 650.7192 | 54085.49 | 54095.13 | 53949.91 | 54078.03 | 54163.93 | 54041.5 | 54085.05 | 53990.6 | 54052 | 53946.18 | 54014.15 | 53997.58 | 53855.24 | 53872.5 |
| 650.8518 | 54067.35 | 54071.53 | 53940.41 | 54041.45 | 54178.03 | 54057.93 | 54024.95 | 53997.6 | 54095.83 | 53912.1 | 53957 | 53960.1 | 53881.63 | 53859.06 |
| 650.9842 | 54091.03 | 54038.68 | 53951.75 | 54038.53 | 54164.98 | 54080.5 | 54066.73 | 53947.75 | 54212.23 | 53988.88 | 53940.15 | 54023.68 | 53867.61 | 53839.94 |
| 651.1167 | 54045.57 | 54087.23 | 53963.75 | 53999.38 | 54121.6 | 54067.68 | 54054.98 | 53976.83 | 54137.4 | 53929.35 | 53919.1 | 53987.13 | 53925.82 | 53853.94 |
| 651.2492 | 54109.49 | 54097.75 | 54015.81 | 54013.73 | 54072.88 | 54062.73 | 54053.65 | 53945.38 | 54100.45 | 53935.9 | 53931.23 | 54025.75 | 53896.63 | 53855.44 |
| 651.3817 | 53991.62 | 54107.93 | 53992.16 | 54030.15 | 54115.05 | 54078.53 | 54050.1 | 53926.95 | 54026.13 | 53919.68 | 53910.03 | 53975.43 | 53879.03 | 53837.06 |
| 651.5142 | 54000.38 | 54039.8 | 53980.44 | 54088.05 | 54139.35 | 54042.95 | 54051.4 | 54009.83 | 54025.05 | 53955.43 | 53905.7 | 53977.28 | 53906.68 | 53820 |
| 651.6466 | 53989.78 | 54031.53 | 53981.59 | 54096.93 | 54134.83 | 54068 | 54041.63 | 53967.28 | 53992.45 | 53919.1 | 53947.38 | 53952.3 | 53886.58 | 53894.31 |
| 651.7791 | 54030.57 | 54046.5 | 53980.81 | 54047.78 | 54120.88 | 54065.73 | 54023.63 | 53942.45 | 54015.7 | 53905.55 | 54006.35 | 53969.78 | 53915.53 | 53918.19 |
| 651.9116 | 54023.49 | 54062.35 | 53985 | 54038.88 | 54175.28 | 54038.53 | 54006.98 | 53992.03 | 54003.95 | 53962.4 | 54012.15 | 53974.58 | 53887.97 | 53853.44 |
| 652.044 | 54009.43 | 54019.98 | 53984.38 | 53978.6 | 54145.05 | 53996.4 | 54007.85 | 53987.38 | 53987.03 | 53926.6 | 53989.48 | 53982.78 | 53858.08 | 53811.5 |
| 652.1765 | 54132.16 | 54046.63 | 53954.31 | 53949.23 | 54134.85 | 54009.58 | 53973.65 | 53979.43 | 54007.08 | 53975.15 | 53962.73 | 53974.3 | 53870.84 | 53834.44 |
| 652.309 | 54022.95 | 54095.05 | 53883.06 | 53983.53 | 54151.03 | 54090.6 | 53972.15 | 53936.93 | 54004.13 | 53949.13 | 53997.18 | 53928.85 | 53876.76 | 53855.25 |
| 652.4414 | 53999.03 | 54091.45 | 53901.25 | 54027.45 | 54059.8 | 54129.58 | 54004.38 | 53918.65 | 53937.05 | 53962.95 | 53950.63 | 53986.25 | 53880.68 | 53849.38 |
| 652.5738 | 53982.65 | 54064.48 | 53985.06 | 54030.68 | 54063.25 | 54049 | 54015.4 | 53891.4 | 53928.85 | 53949.03 | 53952.48 | 53931.6 | 53860.68 | 53820.25 |
| 652.7062 | 53969.51 | 54061.25 | 53926.31 | 53997.4 | 54104 | 54085.65 | 54003.28 | 53893.45 | 53952.2 | 53930.15 | 53935.38 | 53959.83 | 53818.84 | 53922.06 |
| 652.8387 | 53955.65 | 54075.93 | 53860.63 | 54006.73 | 54056.95 | 54045.58 | 53988.13 | 53932.55 | 53959.65 | 53944.35 | 53919.5 | 53981.33 | 53844.92 | 53952.19 |
| 652.9711 | 54054.76 | 54004.33 | 53911.69 | 54008.85 | 54130.28 | 54029.85 | 53997.3 | 53921.68 | 53949.8 | 53954.4 | 53903.98 | 53988.48 | 53819.42 | 53854.88 |
| 653.1035 | 54022.43 | 54041.73 | 53895.63 | 53995.03 | 54144.9 | 54028.93 | 53999.6 | 53904.55 | 53930.3 | 53971.78 | 53899.58 | 53971.75 | 53842.74 | 53898.25 |
| 653.236 | 53972.24 | 54084.95 | 53891.5 | 53980.33 | 54065.7 | 53995.45 | 53991.28 | 53952.25 | 53943.48 | 53953.73 | 53915.85 | 53960.85 | 53852.95 | 53897.5 |
| 653.3684 | 53953.97 | 54001.25 | 53875.88 | 54019.68 | 54091.08 | 54031.08 | 53963.88 | 53907.85 | 53979.38 | 53908.63 | 53972.68 | 53927.63 | 53832.26 | 53951.31 |
| 653.5008 | 53985.11 | 54006.68 | 53923.59 | 54072.85 | 54064.1 | 53990.53 | 54020.23 | 53852.65 | 53967.98 | 53926.48 | 53925.93 | 53956.5 | 53823.32 | 53944.5 |
| 653.6332 | 53979.95 | 54064.1 | 53911.66 | 54016.08 | 54026.7 | 53978.45 | 53969.63 | 53841.88 | 53952.23 | 53929.23 | 53934.28 | 53943.53 | 53843.55 | 53987.25 |
| 653.7656 | 54045.84 | 54044.83 | 53912.25 | 53961.73 | 54029.48 | 54021.35 | 53976.45 | 53892.63 | 53936.7 | 53924.25 | 53935.85 | 53917.45 | 53830.24 | 53873.31 |
| 653.898 | 53973.49 | 54014.5 | 53947.5 | 53988.3 | 54060.73 | 54022.15 | 53984.18 | 53884.08 | 53978.2 | 53925.65 | 53948.4 | 53941.08 | 53867.08 | 53945.31 |
| 654.0303 | 53962.54 | 53996.7 | 53952.28 | 54042.85 | 54040.5 | 53964.9 | 54007.9 | 53913.65 | 53968.35 | 53905.88 | 53923.35 | 53962.78 | 53838.97 | 53891.81 |
| 654.1627 | 53947.86 | 54030.95 | 53951.38 | 54154.3 | 54039.45 | 54001.48 | 54048.13 | 53886.53 | 53989.13 | 53898.73 | 53959.28 | 53968.7 | 53843.87 | 53930.31 |
| 654.2951 | 53968.89 | 54012.38 | 54025.16 | 54075.25 | 54086.78 | 54025.58 | 54022.43 | 53896.85 | 53946.53 | 53900.78 | 53980.8 | 53986.53 | 53831.84 | 53932.38 |
| 654.4275 | 53959.05 | 54049.4 | 54014.5 | 53990.23 | 54027.9 | 54020.85 | 53986.23 | 53891.55 | 53968.98 | 53900.88 | 53956.78 | 53961.98 | 53827.18 | 53939.38 |
| 654.5599 | 53951.73 | 54041.93 | 53984.78 | 54038.48 | 54080.53 | 54052.38 | 53948.98 | 53916.9 | 53961.98 | 53892.35 | 53898.85 | 53970.43 | 53866.29 | 53834.56 |
| 654.6922 | 53882.19 | 53974.98 | 53935.63 | 54033.15 | 54049.53 | 54023.08 | 54016.95 | 53871.8 | 53964.5 | 53917.9 | 53904.93 | 53961.23 | 53830.76 | 53828.5 |
| 654.8246 | 53947.3 | 54071.8 | 53951.84 | 54055.15 | 54011.1 | 54055.1 | 53991.6 | 53855.4 | 53967.9 | 53945.05 | 53903.65 | 53932.88 | 53847.95 | 53879.38 |
| 654.957 | 53939.81 | 54072.3 | 53976.94 | 54004.98 | 54065.4 | 54102.7 | 53960.1 | 53893.5 | 54015.68 | 53970.53 | 53948.08 | 53929.38 | 53816.03 | 53897.81 |
| 655.0893 | 53994.73 | 54051.5 | 53962.34 | 53928.08 | 54012.55 | 54043.93 | 53953.45 | 53880.13 | 53936.45 | 53908.68 | 53937.4 | 53980.65 | 53813.82 | 53860.06 |
| 655.2216 | 53961.08 | 54056.7 | 53994.5 | 53939.5 | 54081.43 | 54029.83 | 53963.78 | 53878.48 | 53989.08 | 54005.28 | 53911.75 | 54014.78 | 53826.63 | 53866.56 |
| 655.354 | 53923.57 | 54052.1 | 53928.06 | 53945.68 | 54145.8 | 53988.63 | 53942.25 | 53915.1 | 53973.13 | 54224.98 | 53889.38 | 54013.73 | 53868.89 | 53840 |
| 655.4863 | 53952.24 | 53985.83 | 53965.06 | 53915.13 | 54092.68 | 54017.6 | 53993.2 | 53946.45 | 53971.08 | 53982.43 | 53913.6 | 53981.18 | 53844.89 | 53840 |
| 655.6187 | 53999.73 | 53962.2 | 53950.13 | 53957.18 | 54113.58 | 54005.9 | 53974.25 | 53933.53 | 53938.7 | 53915.7 | 53938.6 | 53972.1 | 53880.61 | 53832.63 |
| 655.751 | 53990.95 | 53980.53 | 53979.06 | 53996.83 | 54054.13 | 54030.13 | 53936.33 | 53886.7 | 53945.95 | 53912.48 | 53917.98 | 53925.45 | 53895.13 | 53907.31 |
| 655.8833 | 54024.97 | 53978.88 | 53987.69 | 53955.98 | 53997.55 | 54012.68 | 53958.05 | 53861.4 | 53979.5 | 53977.38 | 53951.13 | 53977.5 | 53844.61 | 53884.69 |
| 656.0156 | 54000.22 | 54005.7 | 53992.59 | 53974.05 | 54036.48 | 54032.23 | 54000.33 | 53873.5 | 53958.88 | 53894.28 | 53901.23 | 53951.33 | 53834.76 | 53901.94 |
| 656.148 | 54052.03 | 53927.73 | 53943.63 | 54010.35 | 54004.9 | 54037.98 | 54029.65 | 53924.6 | 53959.95 | 53910.18 | 53976.28 | 53878.08 | 53849.29 | 53964.81 |
| 656.2802 | 54013.32 | 53967.88 | 53936.72 | 54095.55 | 53997.5 | 54027.43 | 53985.65 | 53938.48 | 53991.25 | 53918.48 | 53947.53 | 53908.15 | 53812.03 | 54016.75 |
| 656.4125 | 53951.89 | 53962.23 | 53924.16 | 53980.5 | 54011.23 | 54008.78 | 53969.85 | 53908.18 | 54006.85 | 53923.78 | 53903.58 | 53940.28 | 53814.05 | 53876 |
| 656.5448 | 53957.81 | 53939.9 | 53895.34 | 53955.25 | 53986.2 | 54003.15 | 54007.8 | 53930.03 | 53977.23 | 53917.8 | 53946.38 | 53940.68 | 53841.34 | 53883.56 |
| 656.6771 | 53995.19 | 53948.18 | 53890.06 | 53959.45 | 54017.13 | 53964.5 | 53953.85 | 53927.73 | 54030.23 | 53928.95 | 53950.83 | 53942.23 | 53847.16 | 53895.63 |
| 656.8094 | 53919.86 | 54027.55 | 53859.75 | 53931.85 | 54009.48 | 53935.43 | 53948.6 | 53850.48 | 54021.2 | 53871.38 | 53909.3 | 53922.48 | 53825.29 | 53846.63 |
| 656.9417 | 53952.95 | 54013.6 | 53859.81 | 54035.98 | 54011.18 | 53996.93 | 53921.98 | 53858.35 | 53932.15 | 53893.2 | 53884.7 | 53943 | 53881.34 | 53836.81 |
| 657.074 | 53932.86 | 53999.93 | 53888.75 | 54032.65 | 53973.78 | 53994.85 | 53911.68 | 53940.43 | 53910.53 | 53877.7 | 53903.65 | 53964.15 | 53868.08 | 53857.94 |
| 657.2062 | 53929.35 | 53967.68 | 53945.03 | 54066.85 | 54019.03 | 54000.23 | 53960.28 | 53937.8 | 53986.98 | 53918.2 | 53902.18 | 53911.05 | 53837.32 | 53876.38 |
| 657.3385 | 53925.41 | 53976.85 | 53947.66 | 54009.48 | 54016.45 | 53966.38 | 53953.33 | 53889.2 | 53990.1 | 53877.08 | 53848.3 | 53894.68 | 53815.61 | 53890.75 |
| 657.4708 | 53948.59 | 53990.65 | 53940.94 | 53995.85 | 54033.88 | 53933.28 | 53925.8 | 53876.98 | 53981.6 | 53838.68 | 53852.9 | 53929.28 | 53876.79 | 53848.94 |
| 657.603 | 53935.43 | 54041.08 | 53936.13 | 54060.25 | 54056.68 | 53939.8 | 53908.05 | 53893.53 | 53978.9 | 53923.08 | 53885.3 | 53894.75 | 53818.74 | 53891.75 |
| 657.7353 | 53939.24 | 54023.23 | 53911.72 | 54017.75 | 54047.63 | 54014.2 | 53905.93 | 53931.3 | 53976.85 | 53930.7 | 53908.88 | 53908.35 | 53833.34 | 53902.38 |
| 657.8676 | 53871.97 | 53961 | 53903.81 | 53977.53 | 53984.05 | 54006.33 | 53922.43 | 53882.98 | 53996.55 | 53878.38 | 53920.58 | 53925.5 | 53846.39 | 53907.06 |
| 657.9998 | 53927.89 | 54018.93 | 53893.88 | 54059.03 | 54033.03 | 53977.05 | 53938.18 | 53893.98 | 53994.8 | 53870.28 | 53906.43 | 53928.95 | 53838.16 | 53880.25 |
| 658.132 | 54008.3 | 53966.98 | 53914.38 | 53997.18 | 54046.2 | 53959.88 | 53976.45 | 53892.8 | 54013.65 | 53887.45 | 53922.88 | 53935.18 | 53847.74 | 53931.25 |
| 658.2643 | 53972.08 | 53915.7 | 53930.88 | 53958.95 | 54035.83 | 53966.53 | 53977.65 | 53948.03 | 53949.33 | 53949.75 | 53903.23 | 53920.53 | 53858.45 | 53787.69 |
| 658.3965 | 53945.7 | 53994.43 | 53902.59 | 53982.08 | 54061.13 | 53988.4 | 53954.05 | 53912.6 | 53952.4 | 53920.18 | 53865.85 | 53948.43 | 53843.18 | 53801.56 |
| 658.5287 | 53969.22 | 53998.78 | 53868.22 | 54061.45 | 54059.05 | 54027.1 | 53923 | 53898.15 | 53982.33 | 53882.83 | 53902.05 | 53931.85 | 53830.13 | 53920.38 |
| 658.661 | 53918 | 54014.63 | 53848.94 | 54022.5 | 54078.78 | 53982.48 | 53940.35 | 53901.93 | 53973.03 | 53895.15 | 53928.1 | 53895.45 | 53800.34 | 53860.56 |
| 658.7932 | 53947.73 | 53983.48 | 53882.13 | 54020.1 | 54071.08 | 54000.25 | 53941.93 | 53858.48 | 53980.55 | 53894.6 | 53890.78 | 53932.95 | 53808.53 | 53913.44 |
| 658.9254 | 54017.84 | 53989.05 | 53902.13 | 54018.05 | 54005.85 | 54004.25 | 53935.2 | 53868.63 | 53929.7 | 53940.6 | 53952.78 | 53951.25 | 53817.18 | 53909.88 |
| 659.0576 | 54037.41 | 54009.75 | 53856.09 | 53985.88 | 54077.23 | 53931.25 | 53948.9 | 53902.2 | 53967.3 | 53932.65 | 53930.55 | 53996.43 | 53818.92 | 53928.19 |
| 659.1898 | 54006.89 | 53974 | 53861.69 | 53992.4 | 54041.4 | 53939.45 | 53978.6 | 53883.08 | 53968.75 | 53920.68 | 53936.53 | 53945.6 | 53851.63 | 53994.06 |
| 659.322 | 53976.73 | 53958.58 | 53899.88 | 54014.43 | 54006.95 | 53954.15 | 53979.98 | 53867.4 | 53935.03 | 53892.3 | 53930.13 | 53908.73 | 53871.66 | 53878.81 |
| 659.4542 | 54061.22 | 54010.4 | 53912.03 | 53980.6 | 53989.03 | 53968.7 | 53977.08 | 53822.8 | 53891.53 | 53878.73 | 53920.23 | 53925.23 | 53869.55 | 53916.13 |
| 659.5863 | 54000.38 | 53948.88 | 53950.75 | 53993.13 | 54014.43 | 53984.18 | 53952.4 | 53874.65 | 53902.83 | 53919.25 | 53949.25 | 53897.7 | 53824.89 | 53899.69 |
| 659.7185 | 53978.65 | 53987.1 | 53931.63 | 53996.48 | 54061.13 | 53983.28 | 54005.58 | 53849.73 | 53969.03 | 53932.95 | 53947.28 | 53943.33 | 53834.89 | 53833.81 |
| 659.8507 | 53968.03 | 54008.53 | 53925.31 | 53986.33 | 53983.45 | 54011.05 | 53979.93 | 53864.25 | 53981.3 | 53868.85 | 53920.65 | 53915.03 | 53834.71 | 53873 |
| 659.9829 | 53919.59 | 53977.53 | 53864.09 | 53988.63 | 54032.03 | 54057.13 | 53976.25 | 53856.05 | 53978.35 | 53889.4 | 53909.83 | 53890.58 | 53853.21 | 53832.75 |
| 660.115 | 53959.19 | 53999.05 | 53929.44 | 54021.5 | 54007.85 | 54004.95 | 53971.58 | 53905.6 | 53971.38 | 53891.23 | 53922.8 | 53855.15 | 53830.21 | 53852.5 |
| 660.2472 | 53887.05 | 53969.8 | 53972.88 | 54014.93 | 53987.68 | 54009.78 | 53982.6 | 53888.9 | 53950.4 | 53846.98 | 53909.48 | 53885.5 | 53873.39 | 53825.06 |
| 660.3793 | 53942 | 54044.9 | 53896.53 | 53977.88 | 54004.45 | 54033.43 | 53982.45 | 53893.9 | 53926.7 | 53904.35 | 53913.1 | 53903.78 | 53849.32 | 53840.81 |
| 660.5115 | 53948.51 | 54040.08 | 53891.72 | 54010.95 | 54064.83 | 53967.63 | 53956.78 | 53871.3 | 53964.5 | 53963.4 | 53896.13 | 53914.48 | 53829.82 | 53855.63 |
| 660.6436 | 53902.43 | 54051.63 | 53881.78 | 54031 | 54019 | 53958.6 | 53980.78 | 53857.93 | 53914.83 | 53919.93 | 53909.03 | 53963.53 | 53813.68 | 53900.63 |
| 660.7758 | 53909.89 | 54035.63 | 53967.13 | 53927.65 | 54043.15 | 53906 | 53970.28 | 53940.45 | 53945.58 | 53858.13 | 53895.33 | 53936.1 | 53831.76 | 53931.31 |
| 660.9079 | 53910.51 | 54015.28 | 53948.06 | 53948.58 | 54004.63 | 53980.9 | 53919.45 | 53945.25 | 53963.13 | 53861.75 | 53945.3 | 53936.85 | 53919.03 | 53936.31 |
| 661.04 | 53911.73 | 54061.93 | 53954.84 | 54011.65 | 54048.23 | 54076.05 | 53959.68 | 53886.85 | 53931.78 | 53838.78 | 53943.95 | 53954.6 | 54126.26 | 53955.94 |
| 661.1722 | 53930.16 | 54008.18 | 53978.59 | 53995.73 | 54072.4 | 54095.03 | 53987.75 | 53878.05 | 53954.4 | 53866.8 | 53902.35 | 53958.68 | 53943.95 | 54046.88 |
| 661.3043 | 53946.16 | 53986.98 | 53948.31 | 53958.23 | 54017.08 | 54093.73 | 53949.33 | 53928.73 | 53910.83 | 53935.75 | 53992.43 | 53963.35 | 53926.42 | 53953.31 |
| 661.4364 | 53938.92 | 53998.8 | 53962.88 | 54003.23 | 54022.45 | 54071.23 | 53986.05 | 53911.15 | 53934.4 | 53894.2 | 54010.05 | 53923.18 | 53915.71 | 53913.56 |
| 661.5685 | 53910.68 | 53993.85 | 53974.94 | 53999.03 | 54029.58 | 54008.35 | 53998.63 | 53861.73 | 53941.43 | 53861.95 | 53986.98 | 53908.53 | 53852.68 | 53854.13 |
| 661.7006 | 54011.24 | 54024.5 | 53915.66 | 53996.58 | 54077.5 | 54018.05 | 53978.55 | 53865.98 | 53974.13 | 53875.15 | 54038.65 | 53955.35 | 53914.84 | 53873.44 |
| 661.8327 | 54002.92 | 54041.18 | 53909.5 | 54088.23 | 54040.95 | 53979.98 | 53939.03 | 53887.68 | 53967.25 | 53900.85 | 53991.5 | 53955.28 | 53869.24 | 53897 |
| 661.9648 | 53977.68 | 53954.85 | 53884.53 | 54057.15 | 54093.18 | 53956.93 | 53951.9 | 53877.98 | 53920.65 | 53914.28 | 54100.78 | 53951.8 | 53855.13 | 53888.13 |
| 662.0969 | 53989.05 | 53956.05 | 53894.56 | 54059.43 | 54021.43 | 53948.3 | 53949.35 | 53955.48 | 53885.35 | 53842.5 | 53995.85 | 53930.25 | 53890.39 | 53885.94 |
| 662.229 | 54004 | 53941.2 | 53896.91 | 54068.9 | 54056.95 | 53958 | 53957.83 | 53953.58 | 53937.25 | 53827.3 | 53970.55 | 53910.85 | 53871.21 | 53841.56 |
| 662.3611 | 53931.97 | 53965.08 | 53936.03 | 54021.35 | 54045.95 | 53960.63 | 53931.03 | 53902.03 | 53955 | 53866.03 | 53980.63 | 53946.85 | 53870.03 | 53870.25 |
| 662.4932 | 53948.51 | 53977.88 | 53953.25 | 54005.95 | 54063.33 | 53979.63 | 53953.3 | 53915.85 | 53941.43 | 53934.3 | 53933.1 | 53909.23 | 53914.24 | 53837.06 |
| 662.6252 | 53981.81 | 54010.6 | 53922.81 | 54033.15 | 54032.75 | 54007.65 | 53981.2 | 53855.55 | 53973.9 | 53936.25 | 53936.93 | 53926.45 | 53882.82 | 53845.75 |
| 662.7573 | 53927.05 | 53967.78 | 53971.22 | 54054.9 | 54032.43 | 54031.85 | 53949.53 | 53871.8 | 53924.6 | 53924.68 | 53963.98 | 53969.95 | 53839.66 | 53887.19 |
| 662.8893 | 53995.89 | 53988.98 | 53949.66 | 54112.9 | 53990.08 | 54043.8 | 53921.58 | 53904.4 | 53893.25 | 53888.3 | 53983.55 | 53966.65 | 53830.16 | 53982.56 |
| 663.0214 | 54055.78 | 53962.43 | 53894.19 | 54076.1 | 54029.9 | 54033.93 | 53969.65 | 53913.2 | 53897.55 | 53890.23 | 53950.13 | 54031.45 | 53830.21 | 53884.75 |
| 663.1534 | 53996.08 | 54000.78 | 53914.81 | 54015.13 | 54027.75 | 53948.9 | 53984.13 | 53922 | 53924.75 | 53960.1 | 53939.58 | 53970.2 | 53833.61 | 53920.75 |
| 663.2855 | 53946.54 | 54043.23 | 53920.94 | 54032.43 | 53994.93 | 54013.38 | 53973.5 | 53905.45 | 53911.88 | 53945.7 | 53967.25 | 53955 | 53884.55 | 53955.13 |
| 663.4175 | 53955.81 | 54043.98 | 53933.63 | 53987.93 | 54062.13 | 54029 | 53977.48 | 53905.65 | 53934.73 | 53944.9 | 53935.95 | 53958 | 53836.97 | 53911.75 |
| 663.5496 | 53963.32 | 54062.25 | 53935.97 | 53986.73 | 54039.08 | 54057.15 | 53945.35 | 53878.55 | 53963.1 | 53936.88 | 53951.88 | 53920.23 | 53891.47 | 53970.19 |
| 663.6816 | 53980.19 | 54067.83 | 53955.94 | 53981.3 | 54044.83 | 54062.13 | 53966.55 | 53852.88 | 53973.33 | 53937.33 | 53960.68 | 53985.68 | 53860.26 | 54030.44 |
| 663.8137 | 53963.3 | 54025.85 | 53934.66 | 53983.25 | 54022.08 | 54075.65 | 53968.93 | 53840.35 | 53969.95 | 53872.95 | 53988.05 | 54014.08 | 53893.45 | 53871 |
| 663.9457 | 53948.62 | 53997.73 | 53909.75 | 53994.33 | 54075.05 | 54083.85 | 54028.95 | 53857.15 | 53988.08 | 53896.03 | 54011.28 | 54009.7 | 53911.18 | 53881.06 |
| 664.0777 | 54005.86 | 54012.38 | 53944.66 | 53958.2 | 54017.8 | 54051.4 | 54040.13 | 53894.4 | 53977 | 53887.8 | 53929.48 | 53952.33 | 53901.42 | 53905.56 |
| 664.2097 | 53962.11 | 53991.83 | 53975.19 | 53967.48 | 54043.75 | 54085.4 | 54040.85 | 53913.4 | 54012.33 | 53890.6 | 53938.48 | 53943.23 | 53940.68 | 53844.88 |
| 664.3417 | 53955.68 | 54003.9 | 53972.69 | 53964.85 | 54082.35 | 54098.53 | 54050.93 | 53911.45 | 53946.9 | 53930.55 | 53962.6 | 53949.03 | 53955.26 | 53876.88 |
| 664.4737 | 54042.41 | 53966.68 | 53954.22 | 53998.18 | 54131.88 | 54067.8 | 54029.55 | 53885.7 | 54037.73 | 53944.38 | 54011.38 | 53993.45 | 53868.79 | 53930.06 |
| 664.6057 | 54058.19 | 54036.63 | 53961.09 | 54037.35 | 54042.4 | 54045.75 | 54008.8 | 53945.4 | 54059.4 | 53901.83 | 53942.18 | 53964.93 | 53854.16 | 53923.31 |
| 664.7377 | 54024.51 | 54025.35 | 54021.5 | 54017.78 | 53990.53 | 54044.73 | 54056.78 | 53943.5 | 53984.38 | 53945.45 | 53967.23 | 53998.55 | 53872.39 | 53907.69 |
| 664.8697 | 54023.22 | 53981.38 | 53991.53 | 54032.75 | 54057.28 | 54007.2 | 54033.88 | 53892.4 | 54036.35 | 53933.13 | 53995.35 | 53974.8 | 53890.79 | 53885.13 |
| 665.0017 | 54003.49 | 54000.8 | 53986.19 | 54041.88 | 54048.45 | 53955.6 | 53955.8 | 53984.55 | 53968.8 | 54016.28 | 53941.73 | 53956.48 | 53913.84 | 53907.06 |
| 665.1336 | 54019.35 | 53998.73 | 53950.91 | 54034.18 | 54108.13 | 53991.6 | 53968.38 | 53987.05 | 53972.55 | 53956.48 | 53939.43 | 53967.95 | 53914.82 | 53964.69 |
| 665.2656 | 53945.32 | 54035.53 | 53972.53 | 54005.18 | 54064.63 | 53971.33 | 53997.83 | 53904.9 | 54023.63 | 53896.15 | 53930.78 | 53979.05 | 53895.92 | 53957.75 |
| 665.3976 | 53958.03 | 54061 | 53990.69 | 53974.98 | 54076.48 | 53993.75 | 53962.73 | 53929.23 | 54011.23 | 53925.15 | 53977.35 | 53994.18 | 53906.37 | 53909.06 |
| 665.5295 | 54042.41 | 54053.85 | 53910.66 | 53996.78 | 54047 | 54020 | 53970.1 | 53960.25 | 53965.43 | 53974.4 | 54021.98 | 53989.65 | 53936.87 | 54004.19 |
| 665.6615 | 54030.51 | 54020.2 | 53954.47 | 54010.18 | 54008.75 | 54047.83 | 54004.95 | 53932.65 | 53995.28 | 53939.6 | 54048.13 | 53989.63 | 53884.34 | 53977.81 |
| 665.7934 | 54107.32 | 54098.33 | 53986.78 | 54026.1 | 54086.9 | 54046.55 | 53986.43 | 53908.13 | 53986.9 | 53945.33 | 54021.85 | 53990.1 | 53916.47 | 54002.63 |
| 665.9254 | 54414.49 | 53998.8 | 53988 | 54034.35 | 54109.75 | 54048.1 | 53974.5 | 53917 | 54002.05 | 53933.93 | 53989.73 | 54036.68 | 53906.92 | 53943.94 |
| 666.0573 | 54094.11 | 54028.85 | 53966.94 | 54003.8 | 54143.98 | 54075.2 | 54019.33 | 53953.18 | 53986.7 | 53988.5 | 53967.48 | 53992.38 | 53941.87 | 53912.25 |
| 666.1893 | 54024.76 | 54085.18 | 53939.38 | 54058.05 | 54043.63 | 54072.48 | 53986.98 | 53942.75 | 53981.78 | 54009.68 | 53937.43 | 53967.9 | 53876.26 | 54019.25 |
| 666.3212 | 54028.19 | 54043.75 | 53948.66 | 54035.9 | 54043.35 | 54090.98 | 54013.08 | 53918.25 | 53999.43 | 53966.8 | 53978.13 | 53975.18 | 53896.82 | 54081.69 |
| 666.4531 | 54067.46 | 54020.85 | 53980.28 | 54026.58 | 54084.05 | 54109.23 | 54073.4 | 53949.95 | 54037.03 | 53943.6 | 54016.28 | 53981.8 | 53943.61 | 53958.31 |
| 666.585 | 54058.24 | 53968.43 | 53977.44 | 54073.58 | 53994.95 | 54119.4 | 53981.18 | 53929.7 | 54001.83 | 53971.88 | 54010.35 | 53972.05 | 53964.66 | 53902.31 |
| 666.7169 | 54107.78 | 53998.35 | 54023.94 | 54090.2 | 54040.18 | 54170.85 | 53981.03 | 53953.05 | 54051.05 | 53979.23 | 54021.78 | 53965.68 | 53973.92 | 53988.75 |
| 666.8488 | 54046.41 | 54068.38 | 54031.06 | 54091 | 54018.88 | 54119.63 | 54012.5 | 53918.23 | 54007.83 | 53956.53 | 54063.5 | 54016.6 | 53962.32 | 53985.69 |
| 666.9808 | 54035.54 | 54091.05 | 54004.56 | 54141.6 | 54044.8 | 54140.28 | 54041.88 | 53910.33 | 53979.55 | 53951.6 | 54033.05 | 53987.7 | 53904.97 | 53973.69 |
| 667.1127 | 54086.38 | 54159.25 | 54092.25 | 54056.65 | 54051.2 | 54043.4 | 54016.83 | 54012.05 | 53997.98 | 53955 | 54012.33 | 53995.68 | 53906.61 | 53991.88 |
| 667.2446 | 54104.19 | 54093.83 | 54040.59 | 54036.5 | 54017.15 | 54106.43 | 54038.05 | 53973.33 | 54005.55 | 53960.78 | 53988.23 | 54061.2 | 53895.29 | 53908.94 |
| 667.3765 | 54053.11 | 54051.85 | 53988.53 | 54049.15 | 54037.78 | 54045.78 | 53989.85 | 53966.9 | 54023.68 | 53998.08 | 54018.1 | 54039.98 | 53895.08 | 53884.13 |
| 667.5083 | 54054.78 | 54056.98 | 53981.69 | 54051.13 | 54123.6 | 54000.58 | 54067.7 | 53936.2 | 54097.95 | 54038.18 | 53976.55 | 54008.03 | 53949.71 | 53911.5 |
| 667.6402 | 53993.86 | 54095.23 | 53917.59 | 54006.05 | 54070.03 | 54020.9 | 54052.58 | 53988.15 | 54081 | 54033.85 | 54036.25 | 54014.93 | 53919.89 | 53952.06 |
| 667.7721 | 54051.62 | 54058.6 | 53937.94 | 54000.85 | 54076.53 | 54044.13 | 54003.78 | 53997.35 | 54044.93 | 54020.6 | 54010.78 | 54013.65 | 53926.53 | 54013.38 |
| 667.9039 | 54049.59 | 54102.25 | 53997.31 | 54067.48 | 54003.43 | 54065.75 | 54052.7 | 53975.33 | 54018.03 | 53958.95 | 54023.85 | 54025.28 | 53922.05 | 53981.25 |
| 668.0358 | 54061.59 | 54054.85 | 53943.88 | 54025.95 | 54035.58 | 54073.2 | 54031.93 | 53940.4 | 53990.4 | 53985.33 | 54006.3 | 54039.08 | 53902 | 54033.44 |
| 668.1677 | 54035.7 | 54121.08 | 54018.84 | 54046.98 | 54097.2 | 54037.08 | 54068.35 | 53965.83 | 53990.08 | 53955.75 | 54022.55 | 54094.43 | 53976.55 | 54034.88 |
| 668.2995 | 54067.81 | 54071.68 | 53979.38 | 53986.68 | 54055.4 | 54058.65 | 54041.65 | 53945.28 | 53990.98 | 54016.38 | 54034.25 | 53987.05 | 53901.95 | 53956.88 |
| 668.4314 | 54027.24 | 54059.63 | 53990.25 | 54004.05 | 54117.08 | 54112.55 | 54019.45 | 54011.48 | 54016 | 53970.23 | 54056.55 | 53975.58 | 53952.08 | 53930.31 |
| 668.5632 | 54025.84 | 54046.68 | 54011.16 | 53990.23 | 54018.35 | 54047.8 | 54068.03 | 53960.13 | 54048.33 | 53902.78 | 53988.28 | 54029.48 | 53959.16 | 53983.94 |
| 668.6951 | 53979.78 | 54090.68 | 54030.22 | 54000.88 | 54067.68 | 54035.68 | 54063.1 | 53961.25 | 54034.3 | 53961.15 | 53991.98 | 54050 | 53981.21 | 53900.56 |
| 668.8269 | 53937 | 54066.85 | 53975.97 | 54028.1 | 54065.05 | 54061.3 | 54065.08 | 53919.45 | 53973.48 | 53994.85 | 53996.25 | 54042.45 | 53990.53 | 53982.31 |
| 668.9587 | 54130.7 | 54017.43 | 54008.16 | 54108.38 | 54095.13 | 54129.63 | 54065.58 | 53938.3 | 53991.2 | 53960.03 | 54006.35 | 54048.73 | 53952.66 | 54021.56 |
| 669.0905 | 54120.95 | 54039.68 | 54014.91 | 54001.23 | 54109.53 | 54071.08 | 54097.38 | 53926.6 | 53951.98 | 53950.13 | 53997.18 | 54060.98 | 53964.08 | 53943.56 |
| 669.2224 | 54072.73 | 54013.5 | 54008.16 | 53955.78 | 54090.5 | 54036.8 | 54120.6 | 53971.5 | 53974.7 | 53980.93 | 54075.18 | 54072.28 | 53957.29 | 53979.69 |
| 669.3542 | 54039.19 | 54056.45 | 54019.13 | 54017.23 | 54063.53 | 54036.15 | 54056.85 | 53994.93 | 53974.15 | 53954.83 | 53997.25 | 54075.33 | 53939.18 | 53992.69 |
| 669.486 | 54062.08 | 54041.4 | 54000.31 | 54015.3 | 54054.9 | 54045.48 | 54054.38 | 54013.48 | 54010.05 | 53900.63 | 54006.18 | 54004.58 | 53952.74 | 53913.81 |
| 669.6178 | 54033.11 | 54041.33 | 53976.06 | 54032.33 | 54064.03 | 54044.13 | 54059.85 | 53960.6 | 54007.68 | 53981.33 | 54002.8 | 54021.63 | 53955.82 | 53928.69 |
| 669.7496 | 54050.62 | 54017.73 | 54038.94 | 54039.68 | 54102.5 | 54026.33 | 54055.88 | 53966.93 | 54023.93 | 53939.48 | 54019.85 | 54054.6 | 54000.11 | 53969.19 |
| 669.8814 | 54067.14 | 54048.68 | 53974.09 | 54045.78 | 54137.6 | 54062.38 | 54054.6 | 53996.1 | 53951.9 | 54001.68 | 54017.95 | 54052.53 | 53986.24 | 53942.56 |
| 670.0131 | 54012.62 | 54085.73 | 54004.63 | 54049.7 | 54112.85 | 54029.65 | 54084.3 | 54004.68 | 54034 | 53962.65 | 54036.35 | 53995.75 | 53988.92 | 53932.81 |
| 670.145 | 53999.16 | 54049.2 | 54059.47 | 54017.95 | 54086.95 | 54047.35 | 54069.98 | 53995.83 | 54023.55 | 53946.5 | 54063.43 | 54004.65 | 53967.74 | 53945.63 |
| 670.2767 | 53988.19 | 54065 | 54080.84 | 54031.05 | 54095.1 | 54117.63 | 54122.6 | 53985.1 | 54029.43 | 53964.88 | 54029.48 | 54074.75 | 53935.84 | 53912.13 |
| 670.4085 | 54021.19 | 54057.53 | 54076.56 | 54098.88 | 54159.58 | 54091.2 | 54131.75 | 54047.3 | 54074.38 | 53966.8 | 54021.08 | 54062.88 | 53975.26 | 53894.19 |
| 670.5402 | 54086.22 | 54060.5 | 54067.69 | 54049.08 | 54126.9 | 54016 | 54126.63 | 54015.4 | 54016.18 | 53984 | 54002.98 | 54050.65 | 53950.89 | 53999.38 |
| 670.672 | 54137.41 | 54096.9 | 54075.03 | 54038.63 | 54098.58 | 54070.03 | 54065.25 | 53978.73 | 54045.33 | 53994.78 | 53998.33 | 54097.18 | 53954.05 | 53979.94 |
| 670.8038 | 54133.62 | 54121.6 | 54049.44 | 54099.85 | 54049.83 | 54060.18 | 54088.6 | 53982.5 | 54054.95 | 54002.65 | 54002.63 | 54027.55 | 53968.58 | 53981.06 |
| 670.9355 | 54104.86 | 54055.88 | 54053.31 | 54089.8 | 54107.05 | 54050.03 | 54098.88 | 53965.6 | 54024.7 | 53989.3 | 54046.23 | 54017.13 | 53948.47 | 53983.63 |
| 671.0673 | 54114.46 | 54110.05 | 54109.44 | 54125.93 | 54189.13 | 54013.2 | 54039.6 | 53996.4 | 54054.93 | 53964.95 | 54073.9 | 54001.45 | 53949.45 | 53939.44 |
| 671.199 | 54098.84 | 54104.63 | 54109.91 | 54105.08 | 54122.33 | 54063 | 54069.65 | 53997.93 | 54081.95 | 53983.93 | 54145.05 | 54010.05 | 53960.84 | 54018.06 |
| 671.3308 | 54025.22 | 54124.4 | 54032.75 | 54074.45 | 54114.53 | 54081.33 | 54071.08 | 53979.5 | 54065.8 | 54008.53 | 54087.75 | 54072.48 | 53952.24 | 53991.56 |
| 671.4625 | 54084.65 | 54143.93 | 54079.94 | 54087 | 54209.78 | 54029.95 | 54097.5 | 54019.95 | 54043.28 | 53980.2 | 54155.4 | 54042.25 | 53962.13 | 53913.63 |
| 671.5942 | 54126.81 | 54164.98 | 54175.56 | 54089.4 | 54214.13 | 54089.6 | 54142.88 | 54034.3 | 54080.6 | 53977.25 | 54101.88 | 54041.4 | 54005.68 | 53923.63 |
| 671.7259 | 54102.62 | 54184.33 | 54116.88 | 54055 | 54184.18 | 54128.28 | 54170.85 | 53966.6 | 54070.05 | 54021.2 | 54047.23 | 54048.45 | 53994.26 | 53981.69 |
| 671.8576 | 54069.49 | 54164.43 | 54081.63 | 54040.85 | 54139.18 | 54147.88 | 54128.38 | 53982.45 | 54013.95 | 54031.85 | 54068.7 | 54110.25 | 53970.92 | 53932.38 |
| 671.9893 | 54023.76 | 54110.18 | 54148.16 | 54053.63 | 54171.68 | 54077.35 | 54065.4 | 54010.58 | 53987.93 | 54009 | 54072.65 | 54051.93 | 53963.29 | 53950.38 |
| 672.121 | 54082.59 | 54127.63 | 54060.16 | 54064.3 | 54119.65 | 54004.03 | 54025.15 | 53979.95 | 54041.53 | 54042.58 | 54072.2 | 53998.13 | 53949.24 | 53960.38 |
| 672.2528 | 54115.51 | 54102.08 | 54015.34 | 54044.73 | 54137.18 | 54068.03 | 54046.2 | 54001.73 | 54006.18 | 54040.13 | 54011.7 | 53993.15 | 53924.71 | 53944.69 |
| 672.3844 | 54015.27 | 54078.18 | 53986.28 | 54009.73 | 54117.15 | 54049.65 | 54013.48 | 54023.78 | 54023.85 | 54025.25 | 54024.58 | 53995.8 | 53979.68 | 54066 |
| 672.5161 | 54039.32 | 54123.98 | 53993.66 | 54055.9 | 54154.63 | 54062.8 | 54067.55 | 54018.4 | 54013 | 53994.33 | 54051.35 | 54040.03 | 53956.05 | 53923.94 |
| 672.6478 | 54014.59 | 54158.15 | 53977.25 | 54121.9 | 54168.45 | 54052.1 | 54062.4 | 53960.2 | 54003.13 | 53995.55 | 54056.83 | 54082 | 53962.82 | 53977.81 |
| 672.7795 | 53988.16 | 54107.15 | 53987.94 | 54072.93 | 54104.43 | 54039.68 | 54065.58 | 53936.2 | 54022.9 | 54035.93 | 54024.85 | 54020.33 | 53906.55 | 54056.44 |
| 672.9111 | 53966.59 | 54131.75 | 53966.09 | 54079.78 | 54094.43 | 54045.43 | 54025.83 | 53925.08 | 54038.85 | 54038.1 | 54058.95 | 54015.23 | 53940.63 | 54001.06 |
| 673.0429 | 54081.81 | 54160.5 | 54009.41 | 54040.7 | 54089.35 | 54086 | 54052.75 | 53992.65 | 54045.03 | 53989.03 | 54014.93 | 54007.2 | 53927.08 | 53939.81 |
| 673.1745 | 54093.76 | 54149.23 | 54020.38 | 54055.15 | 54080.93 | 54052.43 | 54115.28 | 54014.55 | 54092.95 | 53971.05 | 54033.73 | 54010.9 | 53954.29 | 53978.88 |
| 673.3062 | 54066.89 | 54038.03 | 53991 | 54115.4 | 54142.73 | 54061.6 | 54026 | 54039.1 | 54062.8 | 54015.25 | 54092.03 | 54010.25 | 54029.08 | 54016.88 |
| 673.4378 | 54029.24 | 54116.8 | 54016 | 54095.8 | 54068.85 | 54042.18 | 53994.73 | 54008.68 | 54061.73 | 53995.95 | 54022.08 | 54042.23 | 54008.32 | 54037.69 |
| 673.5695 | 54047.3 | 54092.23 | 53996.25 | 54087.18 | 54042.28 | 54041.8 | 53993.55 | 53981.7 | 54064.45 | 53953.1 | 54018.28 | 54016.95 | 54002.71 | 54028.56 |
| 673.7011 | 54021.11 | 54052.68 | 53940.53 | 54080.33 | 54081.95 | 54065.73 | 54043.03 | 53985 | 54063.03 | 53939.53 | 54046.18 | 54049.3 | 54003.21 | 54059.75 |
| 673.8327 | 54085.57 | 54062.98 | 53959.78 | 54168.03 | 54076.45 | 54118 | 54114.05 | 54010.18 | 54037.23 | 53978.58 | 54075.75 | 54038.83 | 54005.45 | 53998.13 |
| 673.9644 | 54093.16 | 54068.65 | 53983.84 | 54099.4 | 54071.03 | 54134.6 | 54092.68 | 54051.3 | 54062.6 | 53970.3 | 54039.95 | 54057.88 | 54006.55 | 54014.56 |
| 674.096 | 54048.19 | 54089.1 | 54015.94 | 54065.95 | 54038.58 | 54144.03 | 54056.43 | 54085.85 | 54033.1 | 54019.33 | 54036.35 | 54059.78 | 54038.11 | 53959.5 |
| 674.2276 | 54048.24 | 54122.3 | 54042 | 54089.45 | 54103.25 | 54117.2 | 54111.2 | 54055.95 | 54108.08 | 53985.65 | 54070.53 | 54069.08 | 53992.24 | 54004.38 |
| 674.3592 | 54125.46 | 54055.03 | 54030.16 | 54073.85 | 54106.6 | 54137.3 | 54049.75 | 54017.13 | 54062.98 | 54001.35 | 54056.03 | 54049.73 | 53990.11 | 53999.06 |
| 674.4908 | 54096.51 | 54032.43 | 53997.84 | 54003.7 | 54047.03 | 54124.05 | 54016.55 | 53948.85 | 54021.25 | 54018.85 | 54009.48 | 54072.63 | 53972.79 | 54075.13 |
| 674.6224 | 54025.24 | 54071.65 | 54005.47 | 54113.65 | 54102.68 | 54094.6 | 54079.83 | 54017 | 54051.05 | 54031.78 | 54054.58 | 53970.15 | 53972 | 54069.75 |
| 674.754 | 54075.51 | 54099.18 | 53977.72 | 54050.65 | 54103.83 | 54067.05 | 54019.9 | 54033.1 | 54105.7 | 54046.58 | 54031.85 | 54060.48 | 54011.45 | 54020.19 |
| 674.8856 | 54064.19 | 54108.2 | 54025.38 | 54042.58 | 54054.2 | 54110.78 | 53982.38 | 53976.45 | 54043.45 | 54020 | 54008.8 | 54073.63 | 53941.76 | 53951.75 |
| 675.0172 | 54165.57 | 54134.28 | 53998 | 53968.63 | 54083.63 | 54082.43 | 54003.58 | 53933.95 | 54024.63 | 54038.98 | 54061.08 | 54066.03 | 53917.89 | 53994.56 |
| 675.1488 | 54166.86 | 54152.7 | 54049.59 | 54046.05 | 54138.4 | 54092.28 | 53991.55 | 53937.43 | 54031.63 | 54040.68 | 54017.35 | 54096.95 | 53979.08 | 53980.5 |
| 675.2803 | 54177.97 | 54075.53 | 54054.19 | 54060.73 | 54103.78 | 54073.88 | 53998.88 | 53913.38 | 54028.5 | 53953.48 | 54025.2 | 54033.65 | 53992.63 | 53937.31 |
| 675.4119 | 54072.49 | 54074.98 | 53990.31 | 54114.1 | 54054.93 | 54025 | 53990.1 | 53942.8 | 54006 | 53996.03 | 54044.85 | 54045.55 | 54041.37 | 53908.81 |
| 675.5435 | 54085.27 | 54062.48 | 54006.13 | 54076.05 | 54061.58 | 54060.7 | 54011.4 | 53923.45 | 54028.05 | 53994 | 54037.75 | 54071.93 | 53995 | 53947.13 |
| 675.6751 | 54096.57 | 54134.93 | 54042.19 | 54049.63 | 54087.63 | 54122.83 | 54013.65 | 53942.63 | 54075.15 | 53964.88 | 54117.88 | 54068.75 | 53963 | 54016.38 |
| 675.8066 | 54051.38 | 54098.55 | 54099.19 | 54085.2 | 54109.13 | 54068 | 54058.98 | 53953.95 | 54084.8 | 53970.9 | 54083.48 | 54006.05 | 53978.53 | 54055.63 |
| 675.9382 | 54032.76 | 54072.73 | 54000.41 | 54070.95 | 54108.33 | 54114.45 | 54060.83 | 53963.13 | 54030.5 | 54028.43 | 54031.15 | 53994.6 | 53973.76 | 53988 |
| 676.0697 | 54032.49 | 54087.23 | 54051.44 | 54000.53 | 54135.93 | 54088.78 | 54126.48 | 54004.05 | 54036.45 | 53994.55 | 54041.88 | 54038.45 | 53959.92 | 53988.75 |
| 676.2012 | 53998.89 | 54043.43 | 54024.28 | 54020.3 | 54065.48 | 54124.53 | 54000.78 | 54016.4 | 54007.35 | 53945.55 | 54031.98 | 54020.18 | 54005.5 | 54000.69 |
| 676.3328 | 53988.73 | 54046.83 | 54055.44 | 54038.93 | 54063 | 54151.7 | 54035.1 | 53958.1 | 53977.3 | 53969.13 | 54070.5 | 54015.38 | 54027 | 54015.31 |
| 676.4643 | 53980.84 | 54007.65 | 54063.56 | 54025.2 | 54121.33 | 54115.38 | 54079.18 | 53936.25 | 53974.6 | 53981.28 | 54097.3 | 54064.63 | 53984.87 | 54072.25 |
| 676.5958 | 54016.05 | 54059.35 | 54011 | 54040.2 | 54146.18 | 54143.4 | 54078.73 | 54021.1 | 53992.35 | 53971.78 | 54072.3 | 54084.78 | 53929.87 | 54032.88 |
| 676.7274 | 54079.38 | 54066.75 | 53991.56 | 54036.48 | 54128.73 | 54090.65 | 53980.05 | 53968.75 | 53993.18 | 54003.6 | 54057.25 | 54062.5 | 53960.71 | 54033.38 |
| 676.8589 | 54108.84 | 54079.65 | 54054.63 | 53984.5 | 54083.83 | 54085.25 | 53982.9 | 53977.75 | 54047.65 | 53989.4 | 54060.6 | 54062.85 | 53946.11 | 53958.06 |
| 676.9904 | 54012.3 | 54079.4 | 54043.78 | 53938.83 | 54054 | 54063.38 | 54045.13 | 53964.65 | 54074.38 | 53952.25 | 54019.18 | 54071.7 | 53990.05 | 53960.81 |
| 677.1219 | 53974.46 | 54056.4 | 53992.78 | 54003.45 | 54112.1 | 54103.73 | 54036.95 | 53954.2 | 54056.3 | 53994.58 | 54024.5 | 54046.6 | 54014.47 | 54044.88 |
| 677.2534 | 54011.97 | 54093.73 | 53988.47 | 54051.95 | 54106.23 | 54062.7 | 54013.98 | 53980.85 | 54006.33 | 53995.35 | 54051.45 | 54016.1 | 53990.74 | 54040.5 |
| 677.3849 | 54049.65 | 54085.33 | 54054.16 | 54047.23 | 54059.05 | 54050.85 | 54012.4 | 53965.85 | 54000.98 | 54066.5 | 54041.23 | 54017.55 | 53933.37 | 53988.94 |
| 677.5164 | 54078 | 54078.65 | 53970.75 | 54063.4 | 54071.35 | 54031.08 | 54023.48 | 53991.28 | 54009.65 | 53959.13 | 54005.3 | 54037.18 | 53968.32 | 54023.38 |
| 677.6478 | 54085.73 | 54038.88 | 53957.72 | 54084.18 | 54066.33 | 54054.38 | 54066.55 | 53982.9 | 53999.33 | 53959.23 | 54010.65 | 53999.95 | 54019.05 | 54054.75 |
| 677.7793 | 54076.97 | 54031.95 | 53944.06 | 54037.78 | 54089.05 | 54019.25 | 54027 | 53956.28 | 54014.33 | 54000.03 | 54070.73 | 53979.38 | 53986.32 | 54085.88 |
| 677.9108 | 54093.19 | 54062.98 | 53942.91 | 54080.6 | 54144.25 | 54063.23 | 54014.73 | 53912.1 | 54091.88 | 53960.13 | 54031.55 | 54041.15 | 53998.32 | 54101.81 |
| 678.0422 | 54097.38 | 54076.98 | 53982.13 | 54085.48 | 54098.75 | 54032 | 54033.8 | 53916.3 | 54010.23 | 53995.88 | 54022.88 | 54036.28 | 54005.68 | 54114.06 |
| 678.1737 | 54058.46 | 54097.95 | 53952.94 | 54108.5 | 54089.83 | 54043.25 | 54051.68 | 53928.6 | 54009.48 | 53953.25 | 54012.6 | 54133.6 | 54021.79 | 54132.5 |
| 678.3052 | 54012.62 | 54145.2 | 53956.66 | 54066.23 | 54098.68 | 54085.15 | 54029.13 | 53930.33 | 54029.03 | 53937.93 | 53973.85 | 54027.53 | 53974.95 | 54106.69 |
| 678.4367 | 54022.54 | 54083.08 | 53948.97 | 54012.03 | 54074.25 | 54065.33 | 53971.55 | 53984.38 | 54036.25 | 53933.05 | 54024.58 | 54035.5 | 54027.82 | 54001.63 |
| 678.5681 | 53995.54 | 54048.15 | 53978.53 | 54007.98 | 54057.45 | 54113.78 | 53989.53 | 53997.88 | 54061.4 | 53923.08 | 54024.7 | 54038.85 | 54026.26 | 54042.88 |
| 678.6995 | 54067 | 54081.9 | 54015.16 | 54063 | 54104.48 | 54032.23 | 54017.25 | 53988.55 | 54046.25 | 54019.58 | 53983.05 | 54056.23 | 54023.66 | 54016.63 |
| 678.8309 | 54022.27 | 54069.25 | 54039.59 | 53998.45 | 54081.58 | 54054.53 | 54035.33 | 53971.98 | 54007.68 | 54037.6 | 54023.93 | 54003.38 | 53990.08 | 53956.63 |
| 678.9623 | 54005.65 | 54024.73 | 54006.94 | 53985.93 | 54154.23 | 54059.68 | 54000.05 | 53989.58 | 53974.78 | 53963.13 | 54040.95 | 54088.83 | 54005.39 | 53973.19 |
| 679.0938 | 54011.24 | 54083.63 | 53971.91 | 53993.43 | 54039.78 | 54022.98 | 53988.98 | 53992.98 | 53983.05 | 53953.73 | 54067.53 | 54044.1 | 54003.66 | 54014 |
| 679.2252 | 54016.54 | 54042.45 | 53993.47 | 54030.25 | 54012.2 | 53995.85 | 53973.93 | 54000.58 | 53990.25 | 53956.48 | 54035.8 | 54049.05 | 53998.42 | 54025.38 |
| 679.3566 | 54010.14 | 54085.73 | 53978.34 | 53987.68 | 54127.35 | 54045.58 | 54036.05 | 53936.48 | 53967.35 | 53978.78 | 53964.58 | 54003.53 | 53937.63 | 54009.5 |
| 679.488 | 54048.73 | 54110.98 | 53962.91 | 54053.08 | 54103.95 | 54036.3 | 54092.35 | 53953.73 | 53970.63 | 53985.58 | 54030.58 | 54045.6 | 53928.61 | 54009.19 |
| 679.6195 | 53992.57 | 54091.68 | 53993.19 | 54065.15 | 54132.05 | 54044.1 | 54032.5 | 53986.55 | 53985.88 | 53982.2 | 54092.6 | 54041.63 | 53955.42 | 53942.75 |
| 679.7509 | 54051.27 | 54046.08 | 53936.5 | 54001.93 | 54088.68 | 54016.53 | 54104.13 | 53973.43 | 54024.68 | 53975.9 | 54080.6 | 53981.8 | 53914.55 | 53938.56 |
| 679.8822 | 53990.51 | 53968.53 | 53921.94 | 54034.58 | 54093.23 | 54000.25 | 54027.63 | 53934.15 | 54038.9 | 53956.6 | 54075.15 | 53985.3 | 54007.24 | 53945.81 |
| 680.0136 | 53992.35 | 53983.83 | 53964.47 | 54049.78 | 54087.6 | 53986.7 | 54034.53 | 53941.25 | 54024.13 | 53996.6 | 54041.83 | 54065.18 | 53994.74 | 54004.69 |
| 680.145 | 54009.49 | 54056.53 | 53992.13 | 54059.4 | 54051.83 | 54033.78 | 54022.75 | 53973.58 | 54040.53 | 53973.38 | 53984.05 | 54041.48 | 53943.47 | 53975.13 |
| 680.2764 | 54011.43 | 54049.48 | 53966.69 | 54028.28 | 54071.78 | 54050.5 | 54018.88 | 53949.68 | 53975.7 | 54036 | 54011.35 | 54028.13 | 53949.03 | 53961.94 |
| 680.4077 | 53958.43 | 54015.78 | 53897.03 | 54053.53 | 54121.55 | 54040.18 | 54050.53 | 53916.83 | 54010.98 | 53985.83 | 53991.45 | 54029.05 | 53964.97 | 54046.81 |
| 680.5391 | 53989.65 | 54056.88 | 53937.5 | 54000.3 | 54148.83 | 54077.53 | 54090.5 | 53896.83 | 53974.2 | 53960.38 | 54036.7 | 54051.2 | 53974.92 | 54017.94 |
| 680.6705 | 54013.59 | 54089.45 | 53934.03 | 53990.35 | 54097.55 | 54013.65 | 54088.03 | 53928.2 | 53947.18 | 53952.88 | 54060.1 | 54028.43 | 53915.5 | 53936 |
| 680.8018 | 54026.16 | 54069.53 | 54033.22 | 54004.73 | 54025.4 | 54022.9 | 54027.13 | 53962.15 | 54001.55 | 53990.4 | 54010.65 | 53962.9 | 53933.87 | 53990.13 |
| 680.9332 | 53977.14 | 54082.83 | 54012.13 | 54034.13 | 54008.18 | 54043.95 | 53963.78 | 53924.88 | 54010.55 | 53952.08 | 54034.98 | 53988.68 | 53941.13 | 53988.31 |
| 681.0645 | 54019.11 | 54077.78 | 53994.91 | 54076.93 | 54067.8 | 54057.18 | 54004.83 | 53957.33 | 53957.63 | 53922.7 | 53974.45 | 54031.5 | 53958.03 | 53964.94 |
| 681.1958 | 54101.35 | 54039.5 | 53992.53 | 54077.35 | 54040.7 | 54055.03 | 53985.1 | 53951.13 | 53930.58 | 53959.78 | 53988.55 | 54023.6 | 53972.08 | 53956.25 |
| 681.3272 | 54108.3 | 53960.93 | 53992.44 | 54103.28 | 54057.9 | 53996.6 | 54030.18 | 54029.58 | 53929.35 | 53962.78 | 53985.23 | 53964.1 | 53969.95 | 53995.25 |
| 681.4585 | 54053.59 | 54019.6 | 53964.78 | 54063.53 | 54025.63 | 54006.35 | 54064.03 | 54029.95 | 53949.83 | 53962.45 | 54006.95 | 53986.55 | 53924.82 | 53993.38 |
| 681.5898 | 54047.54 | 54045.58 | 53936.06 | 54083.93 | 54034.83 | 54041.43 | 53993.48 | 53987.15 | 54013.55 | 53979.75 | 53973.83 | 53950.25 | 53901.5 | 54040 |
| 681.7211 | 54006.14 | 54032.05 | 53963.44 | 53981.48 | 54039.2 | 54050.2 | 53930.43 | 53952.1 | 53973.2 | 53961.33 | 53952.85 | 54008.55 | 53901.58 | 53911.94 |
| 681.8524 | 54039.19 | 53993.35 | 53919.5 | 53987.95 | 54014.73 | 54075.95 | 53943.88 | 53899.73 | 53919.23 | 53928.05 | 53983.13 | 54098.15 | 53904.87 | 53930.13 |
| 681.9837 | 54000 | 53965.88 | 53906.22 | 53992.93 | 54052.88 | 54020.08 | 53999.23 | 53924.88 | 53919.6 | 53924.38 | 53979.88 | 54018.15 | 53905.5 | 53899.38 |
| 682.1151 | 53981.14 | 54021.73 | 53928.84 | 53985.85 | 54059.05 | 53998.65 | 53967.88 | 53920.95 | 53912.83 | 53921.58 | 53933.05 | 54025 | 53909.18 | 53926.5 |
| 682.2463 | 54020.54 | 53987.15 | 53941.31 | 53979.28 | 54038.2 | 54019.93 | 53986.93 | 53886.88 | 53898.38 | 53947.23 | 53938.18 | 54020.18 | 53892.05 | 53929.88 |
| 682.3776 | 53996.38 | 53997.15 | 53931.88 | 53969 | 54000.23 | 54008.88 | 53982.63 | 53890.88 | 53922.45 | 53920.73 | 53989.08 | 54044.48 | 53905.24 | 53918 |
| 682.5089 | 54042.3 | 54000.03 | 53983.22 | 53903.4 | 53983.15 | 53969.03 | 53983.55 | 53907.88 | 53955.23 | 53904.45 | 54030.33 | 53957.4 | 53920.61 | 53966.13 |
| 682.6401 | 54033.08 | 53982.63 | 53931.47 | 53913.05 | 54019.13 | 54009.75 | 54011.4 | 53899.83 | 53978.03 | 53858.88 | 54013 | 54007.23 | 53912.71 | 53927.75 |
| 682.7714 | 53974.24 | 54051.65 | 53916.69 | 53986.43 | 54076.15 | 53975.6 | 53993.85 | 53928.58 | 53977.7 | 53876.13 | 53958.3 | 54000.93 | 53894.39 | 54005.13 |
| 682.9027 | 53941.49 | 54062.13 | 53942.56 | 53981.68 | 54075.88 | 54054.68 | 53974.88 | 53904.18 | 53962.8 | 53916.18 | 53941.13 | 54028.25 | 53930.03 | 53921.69 |
| 683.0339 | 54011.46 | 54024.33 | 53890.88 | 53965.5 | 54074.18 | 54024.33 | 53952.25 | 53928 | 53973.58 | 53932.48 | 53981.5 | 54038.05 | 53922.18 | 53920.31 |
| 683.1652 | 54032.57 | 54064.15 | 53882.63 | 53975.65 | 54069.3 | 53916.08 | 53976.88 | 53934.85 | 53892.83 | 53929.05 | 53968.93 | 53962.6 | 53945.68 | 53902.13 |
| 683.2965 | 53975.51 | 54075.33 | 53899.75 | 53978.9 | 53968.25 | 53940.1 | 53944.23 | 53852.3 | 53909.85 | 53929.05 | 53935.93 | 54009.53 | 53877.05 | 53863.13 |
| 683.4277 | 53964.73 | 54091.43 | 53909.84 | 53981.98 | 53993.05 | 53969.35 | 53960.18 | 53883.65 | 53962.95 | 53901.6 | 53950.03 | 53960.3 | 53916.45 | 53867.88 |
| 683.559 | 53957.62 | 54056.7 | 53971.59 | 53984.18 | 54046.93 | 54031.58 | 53951.08 | 53906.58 | 53947.18 | 53890.1 | 54009.53 | 53958.75 | 53934.45 | 53892 |
| 683.6902 | 53948.76 | 54013.18 | 53983.66 | 53976.05 | 54042.63 | 54046.08 | 53940.48 | 53904.9 | 53913.65 | 53884.55 | 53925.33 | 53913.18 | 53955.68 | 53889.94 |
| 683.8214 | 53927.03 | 53974.15 | 53935.69 | 54038.65 | 53993.2 | 54020.7 | 53991.28 | 53966.18 | 53908.18 | 53867.1 | 53916.3 | 53941.88 | 53943.45 | 53913.63 |
| 683.9526 | 53977.05 | 53980.65 | 53960.78 | 54020.7 | 54029.63 | 54002.95 | 53950.2 | 53880.25 | 53905.93 | 53878.98 | 53935.03 | 53919.25 | 53964.68 | 53902.63 |
| 684.0839 | 53990.05 | 53943.08 | 53927.47 | 53936 | 54006.3 | 53967.7 | 53942.88 | 53902.58 | 53923 | 53879.03 | 53883.05 | 53944.78 | 53944.34 | 53979.56 |
| 684.215 | 53996.08 | 53954.85 | 53923.19 | 54011.05 | 54011.65 | 53962.23 | 53911.83 | 53885.55 | 54012.08 | 53890.63 | 53962.9 | 53962.53 | 53915.55 | 53925.88 |
| 684.3463 | 53913.62 | 53958.78 | 53928 | 54010.38 | 54017.58 | 53962.8 | 53980.33 | 53839.5 | 53953.2 | 53951.43 | 53931.1 | 53972.55 | 53895.95 | 53895.69 |
| 684.4775 | 53916.35 | 53982.55 | 53902.56 | 53905.75 | 54000.1 | 53988.33 | 53929.5 | 53873.05 | 53898.7 | 53900.65 | 53947 | 53941 | 53889.34 | 53821.81 |
| 684.6086 | 53941.11 | 53990.55 | 53895.69 | 53909.78 | 54024.7 | 53912.35 | 53928.35 | 53904.05 | 53909.6 | 53915.1 | 54008.88 | 53944.78 | 53905.89 | 53901.06 |
| 684.7398 | 53969.7 | 53992.78 | 53907.41 | 53937.55 | 54034.75 | 53965.23 | 53960.43 | 53919.18 | 53941.35 | 53893.15 | 53942.48 | 53989.53 | 53928.76 | 54005.69 |
| 684.871 | 53996.92 | 53990.13 | 53877.34 | 53955.95 | 54024.23 | 54012.33 | 53883.9 | 53899.55 | 53945.8 | 53845.28 | 53873 | 53946.75 | 53897.71 | 53889.63 |
| 685.0022 | 53980.05 | 53960.4 | 53849.28 | 53945.8 | 54051.63 | 53947.88 | 53885.88 | 53911.9 | 53853.38 | 53843.58 | 53953.45 | 54003.9 | 53858.42 | 53934 |
| 685.1334 | 53930.54 | 53975.55 | 53940.5 | 53921.4 | 54043.53 | 53968.15 | 53918.8 | 53920.85 | 53913.58 | 53835.33 | 53949.58 | 53935.95 | 53867.76 | 53868 |
| 685.2645 | 53929.54 | 53978.33 | 53918.06 | 53921.6 | 53998.78 | 53955.23 | 53879.2 | 53942.23 | 53877.35 | 53818.9 | 53941.7 | 53933.18 | 53884.66 | 53857 |
| 685.3957 | 54001.78 | 53915.98 | 53937.44 | 53928.48 | 53961.03 | 53946.5 | 53863.25 | 53914.03 | 53918.53 | 53887.15 | 53923.83 | 53977.75 | 53863.5 | 53885.38 |
| 685.5269 | 53958.89 | 53955.13 | 53900.63 | 53974.45 | 53941.43 | 53924.88 | 53906.85 | 53888.6 | 53898.45 | 53884.03 | 53899 | 53922.03 | 53856.37 | 53889.69 |
| 685.658 | 53903.49 | 53904.78 | 53887.75 | 53899.88 | 53930.75 | 53950.7 | 53888.28 | 53908.95 | 53864.33 | 53850.45 | 53901.48 | 53915.35 | 53862.05 | 53905.94 |
| 685.7891 | 53905.16 | 53908.15 | 53930.69 | 53924.78 | 53932.95 | 53941.28 | 53869.48 | 53850.73 | 53905.73 | 53880.48 | 53941.45 | 53886.7 | 53877.71 | 53951.13 |
| 685.9203 | 53896.32 | 53917.53 | 54028.25 | 53924.2 | 53943.93 | 53975.23 | 53886.88 | 53867.33 | 53903.6 | 53906.43 | 53912.85 | 53894.75 | 53869.55 | 53929.19 |
| 686.0514 | 53901.54 | 53898.35 | 54006.41 | 53911.5 | 53997.88 | 53957.58 | 53942.93 | 53870.08 | 53905 | 53894.38 | 53945.35 | 53953.4 | 53860.45 | 53990.75 |
| 686.1826 | 53909.84 | 53899.98 | 53900.97 | 53935.73 | 53988.6 | 53967.08 | 53931.58 | 53909.58 | 53903.8 | 53862.4 | 53942.55 | 53908.13 | 53848.08 | 54000.25 |
| 686.3137 | 53887.92 | 53942.8 | 53868.44 | 53911.38 | 53965.73 | 53936.93 | 53938.13 | 53857.03 | 53907.15 | 53875.53 | 53957.9 | 53917.93 | 53843.24 | 53960.25 |
| 686.4448 | 53894.84 | 53908.68 | 53875.72 | 53928.78 | 53950.58 | 53920.68 | 53921.8 | 53828.35 | 53886.88 | 53833.63 | 53902.75 | 53919.25 | 53853.61 | 53893.13 |
| 686.5759 | 53882.46 | 53873.73 | 53920.63 | 53955.18 | 54002.63 | 53885.58 | 53890.5 | 53847.13 | 53864 | 53837.3 | 53916.48 | 53927.8 | 53886.29 | 53844.06 |
| 686.707 | 53911.65 | 53922.58 | 53936.59 | 53917.8 | 54051.73 | 53901.43 | 53870.68 | 53844.45 | 53871.3 | 53853.3 | 53909.6 | 53906.38 | 53874.66 | 53913.13 |
| 686.8381 | 53910.62 | 53904.3 | 54261.31 | 53900.83 | 54014.88 | 53918.65 | 53878.65 | 53843.35 | 53906.58 | 53853.95 | 53910.18 | 53923.18 | 53871.29 | 53865.19 |
| 686.9692 | 53900.76 | 53927.78 | 54139.34 | 53877.3 | 53948.5 | 53916.15 | 53877.88 | 53829.05 | 53905.88 | 53846.1 | 53900.08 | 53934.35 | 53863.24 | 53887.56 |
| 687.1003 | 53927.89 | 53946.4 | 53969.09 | 53904.38 | 53992 | 53999.9 | 53908.4 | 53810 | 53903.98 | 53836.75 | 53922.08 | 53883.53 | 53870.82 | 53881.19 |
| 687.2313 | 53886.97 | 53954.7 | 53951.06 | 53938.75 | 53924.3 | 53980.73 | 53907.13 | 53847.3 | 53889.35 | 53884.23 | 53945.8 | 53883.1 | 53859.29 | 53844.56 |
| 687.3624 | 53883.08 | 53974.45 | 53966.44 | 53901.35 | 53890.98 | 53965.83 | 53914.53 | 53811.85 | 53957.83 | 53883.03 | 53952.48 | 53881.6 | 53860.97 | 53847.88 |
| 687.4935 | 53891.57 | 53908.05 | 54038.41 | 53907.28 | 53933.33 | 53925.58 | 53897.75 | 53826.23 | 53865.88 | 53866.35 | 53915.98 | 53874.1 | 53881.32 | 53893.31 |
| 687.6246 | 53904.14 | 53911.15 | 53930.34 | 53887.28 | 53938.55 | 53953.28 | 53867.25 | 53848.98 | 53874.53 | 53853.13 | 53943.63 | 53903.6 | 53871.63 | 53840.81 |
| 687.7556 | 53881.76 | 53919.9 | 53881.69 | 53893.5 | 53948.28 | 53947.45 | 53877.98 | 53831.08 | 53918.3 | 53856.83 | 53885.25 | 53895.45 | 53855.89 | 53875 |
| 687.8867 | 53879.27 | 53940.23 | 53855 | 53920.88 | 53960.5 | 53920.35 | 53835.5 | 53804.73 | 53880.8 | 53863.53 | 53974.88 | 53887.35 | 53880.95 | 53957.63 |
| 688.0177 | 53882.65 | 53917.23 | 53836.16 | 53886.05 | 53928.68 | 53919.33 | 53833.3 | 53825.8 | 53882.1 | 53846.93 | 53885.93 | 53868.43 | 53815.16 | 53858.81 |
| 688.1487 | 53928.97 | 53917.85 | 53851.28 | 53868.78 | 53913.48 | 53919.93 | 53880.93 | 53829.35 | 53872 | 53837.8 | 53892.7 | 53870.15 | 53805.26 | 53873.31 |
| 688.2798 | 53893.59 | 53885.18 | 53824.94 | 53863.18 | 53890.23 | 53925.15 | 53891.8 | 53818.2 | 53845.9 | 53833.3 | 53896.43 | 53897.55 | 53867.53 | 53912.69 |
| 688.4108 | 53889.97 | 53935.48 | 53806.66 | 53887.65 | 53897.35 | 53934.88 | 53901.65 | 53810.08 | 53872.93 | 53831.9 | 53885.65 | 53873.18 | 53837.5 | 53838.94 |
| 688.5419 | 53880.89 | 53918.18 | 53865.69 | 53879.18 | 53932.28 | 53879.15 | 53861.53 | 53819.68 | 53890.13 | 53839.9 | 53882.9 | 53920.95 | 53826.89 | 53805.44 |
| 688.6729 | 53887.84 | 53913.4 | 53853.94 | 53884.63 | 53912.8 | 53852.4 | 53874.75 | 53861.23 | 53849.13 | 53825 | 53930.65 | 53887.4 | 53790.87 | 53819.75 |
| 688.8039 | 53924.62 | 53935.83 | 53827.69 | 53911.38 | 53885.73 | 53888.15 | 53867.25 | 53811.08 | 53832.45 | 53828.1 | 53891.68 | 53878.88 | 53811.61 | 53823.88 |
| 688.9349 | 53879.19 | 53944.83 | 53846.56 | 53925.6 | 53894.58 | 53886 | 53888.9 | 53794.3 | 53850.73 | 53818.63 | 53867.73 | 53894.23 | 53792.74 | 53788.88 |
| 689.0659 | 53834.35 | 53936 | 53847.25 | 53878.9 | 53919.7 | 53887.68 | 53867.95 | 53822.08 | 53867.03 | 53805.1 | 53862.4 | 53877.75 | 53812.84 | 53789.44 |
| 689.1969 | 53849.11 | 53930.48 | 53824.06 | 53853.78 | 53903.18 | 53885.93 | 53858.98 | 53813.18 | 53823.63 | 53799.45 | 53883.08 | 53846.43 | 53806.37 | 53840.31 |
| 689.3279 | 53871.54 | 53874.35 | 53776.78 | 53858.75 | 53902.23 | 53900.03 | 53914.83 | 53774.18 | 53836.5 | 53817.53 | 53865.7 | 53826.58 | 53804.34 | 53887.31 |
| 689.4589 | 53874.3 | 53932.55 | 53802.28 | 53839.7 | 53950.08 | 53894.85 | 53904.03 | 53826.03 | 53835.13 | 53798.05 | 53854.75 | 53862.4 | 53809.79 | 53857.13 |
| 689.5899 | 53859.92 | 53922.58 | 53821.53 | 53864.23 | 53881.7 | 53850.68 | 53926.98 | 53806.98 | 53855.03 | 53806.25 | 53846.35 | 53870.28 | 53849.95 | 53800.63 |
| 689.7208 | 53916.19 | 53943.78 | 53822.75 | 53839.78 | 53839.75 | 53836.4 | 53851.48 | 53799.83 | 53831.13 | 53809.58 | 53874.78 | 53868.15 | 53842.63 | 53813.5 |
| 689.8518 | 53894.22 | 53930.98 | 53768.13 | 53870.53 | 53883.03 | 53908.65 | 53856.33 | 53800.15 | 53859.5 | 53790.65 | 53874.18 | 53880.03 | 53871.97 | 53848.06 |
| 689.9828 | 53913.38 | 53883.2 | 53812.72 | 53886.8 | 53878.2 | 53871.63 | 53889 | 53813.48 | 53856.03 | 53810.88 | 53892.73 | 53872.88 | 53858.37 | 53867.06 |
| 690.1138 | 53889.84 | 53893.18 | 53868.25 | 53847.13 | 53869.7 | 53852.55 | 53899.1 | 53788.18 | 53850.45 | 53808.98 | 53940.08 | 53869.13 | 53826.42 | 53830.38 |
| 690.2447 | 53844.49 | 53907.03 | 53836.84 | 53828.93 | 53904.15 | 53816.43 | 53881.53 | 53813.53 | 53833.95 | 53800.4 | 53924.15 | 53887.43 | 53809.53 | 53858.56 |
| 690.3757 | 53861.38 | 53884.93 | 53828.44 | 53839.8 | 53901.93 | 53870.95 | 53881.7 | 53857.68 | 53861.15 | 53791.53 | 53937.43 | 53873.88 | 53757.84 | 53890.38 |
| 690.5066 | 53849.14 | 53879.08 | 53837.31 | 53826.23 | 53892.85 | 53863.5 | 53902.78 | 53874.83 | 53845.53 | 53800.48 | 53905.83 | 53859.58 | 53788.29 | 53907.44 |
| 690.6375 | 53835.32 | 53874.4 | 53817.56 | 53846.03 | 53931.3 | 53837.25 | 53833.23 | 53860.7 | 53823.8 | 53809.1 | 53877.05 | 53866.43 | 53830.84 | 53865.19 |
| 690.7684 | 53842.32 | 53854.65 | 53793.59 | 53864.48 | 53908.65 | 53878.03 | 53845.95 | 53791.03 | 53866.6 | 53828.03 | 53867.88 | 53890.68 | 53838.58 | 53833.5 |
| 690.8994 | 53843.49 | 53860.75 | 53832.75 | 53866.4 | 53887.33 | 53883.85 | 53851.48 | 53803.3 | 53834.85 | 53847.15 | 53851.5 | 53873.83 | 53773.68 | 53798 |
| 691.0303 | 53788.22 | 53857.18 | 53785.16 | 53863.88 | 53917.3 | 53879.83 | 53817.8 | 53769.98 | 53820.15 | 53841.08 | 53855.15 | 53842.6 | 53768.42 | 53792.88 |
| 691.1612 | 53830.59 | 53819.25 | 53828.47 | 53841.78 | 53903 | 53858.9 | 53821.53 | 53806.55 | 53801.8 | 53808.35 | 53867.48 | 53853.53 | 53864.47 | 53837.56 |
| 691.2921 | 53843.97 | 53849.55 | 53778.94 | 53806.8 | 53883.63 | 53902.4 | 53834.25 | 53810 | 53823.5 | 53817.08 | 53870.28 | 53818.2 | 53818.32 | 53830.94 |
| 691.423 | 53824.76 | 53862.8 | 53779.44 | 53840.98 | 53906.1 | 53853.93 | 53808.45 | 53804.75 | 53822 | 53843.45 | 53876.4 | 53820.4 | 53825.76 | 53788.06 |
| 691.5539 | 53788.51 | 53862 | 53811.88 | 53823.33 | 53924.3 | 53855.1 | 53842.15 | 53759.45 | 53820.63 | 53827.08 | 53863.58 | 53815.08 | 53779.11 | 53804.13 |
| 691.6848 | 53839.35 | 53821.25 | 53778.88 | 53821.98 | 53863.05 | 53900.5 | 53857.53 | 53792.1 | 53845.13 | 53855.7 | 53855.13 | 53775.25 | 53771.42 | 53792.31 |
| 691.8157 | 53832.38 | 53881.78 | 53776.66 | 53867.43 | 53856.83 | 53938.58 | 53809.7 | 53816.18 | 53818.65 | 53822.45 | 53850.7 | 53802.8 | 53835.97 | 53817.63 |
| 691.9466 | 53821.62 | 53894.43 | 53802.16 | 53847.15 | 53848.03 | 53855.58 | 53815.45 | 53799.98 | 53797 | 53821.53 | 53841.9 | 53830.35 | 53805.42 | 53830 |
| 692.0775 | 53809.32 | 53839.4 | 53799.88 | 53855.98 | 53838.53 | 53838.03 | 53840.08 | 53802.8 | 53834.38 | 53829.4 | 53824.78 | 53847.58 | 53781.18 | 53815.13 |
| 692.2083 | 53839.35 | 53827.98 | 53828.88 | 53824.8 | 53883.4 | 53856.35 | 53810.85 | 53799.43 | 53868.83 | 53769.65 | 53841.45 | 53864.8 | 53797.13 | 53871.44 |
| 692.3392 | 53820.3 | 53828.38 | 53810.97 | 53828.48 | 53884.7 | 53831.13 | 53828.28 | 53792 | 53827.98 | 53771.8 | 53838.9 | 53833.75 | 53827.08 | 53958.94 |
| 692.47 | 53812.14 | 53848.83 | 53840.28 | 53816.3 | 53904.8 | 53853.55 | 53807.55 | 53762.68 | 53773 | 53799.85 | 53856.2 | 53836.3 | 53861.53 | 53872.5 |
| 692.6009 | 53842.51 | 53823.88 | 53831.25 | 53824.18 | 53868 | 53837.45 | 53802.68 | 53751.13 | 53779.7 | 53806.2 | 53826.8 | 53847.95 | 53806.79 | 53852.5 |
| 692.7318 | 53810.22 | 53842.85 | 53785.53 | 53836.2 | 53893.88 | 53892.4 | 53821.48 | 53818.08 | 53778.55 | 53761.93 | 53859.45 | 53846.43 | 53748.76 | 53806.13 |
| 692.8626 | 53807.46 | 53864.88 | 53845.5 | 53833.08 | 53927.18 | 53861.3 | 53816.25 | 53854.25 | 53812.85 | 53780.38 | 53849.5 | 53844.03 | 53763.47 | 53828 |
| 692.9934 | 53801.65 | 53815.43 | 53852.84 | 53829 | 53898.75 | 53860.98 | 53839.33 | 53772.1 | 53829.78 | 53793.48 | 53817.95 | 53831.7 | 53774 | 53779.81 |
| 693.1242 | 53794.92 | 53804.08 | 53839.31 | 53869.15 | 53925.63 | 53838.25 | 53847.85 | 53761.3 | 53795.5 | 53781.08 | 53799.55 | 53796.5 | 53753.87 | 53804.44 |
| 693.2551 | 53850.11 | 53824.85 | 53792.91 | 53839.8 | 53946.45 | 53833.13 | 53820.25 | 53771.3 | 53816.83 | 53792.05 | 53823.53 | 53815.28 | 53762.84 | 53792.81 |
| 693.3859 | 53881.27 | 53855.65 | 53790.69 | 53843.83 | 53898.85 | 53885.75 | 53814.73 | 53751.28 | 53844.48 | 53754.13 | 53847.35 | 53818.1 | 53790.13 | 53849.81 |
| 693.5167 | 53816.19 | 53836.23 | 53818.47 | 53843.9 | 53898.23 | 53841.98 | 53814.9 | 53768.1 | 53849.45 | 53807.4 | 53819 | 53837.8 | 53742.39 | 53820.25 |
| 693.6475 | 53826.11 | 53842.88 | 53816.22 | 53790.73 | 53874.8 | 53834.63 | 53793.55 | 53757.63 | 53837.33 | 53814.9 | 53817.5 | 53813.6 | 53779.08 | 53775.5 |
| 693.7783 | 53833.03 | 53849.15 | 53816.41 | 53833.13 | 53814.88 | 53821.33 | 53803.85 | 53806.13 | 53808.8 | 53855.98 | 53878.75 | 53771.03 | 53782.24 | 53799.94 |
| 693.9091 | 53774.59 | 53852.23 | 53787.63 | 53807.65 | 53831.03 | 53820.18 | 53829.13 | 53787.35 | 53851.53 | 53881.13 | 53847.28 | 53797.08 | 53758.37 | 53796.44 |
| 694.0399 | 53763 | 53879.23 | 53771.13 | 53769.18 | 53843.2 | 53804.4 | 53807.98 | 53803.9 | 53844.48 | 53822.33 | 53852.68 | 53844.1 | 53762.08 | 53823.38 |
| 694.1707 | 53784.27 | 53841.35 | 53769.03 | 53802.98 | 53839.15 | 53800.8 | 53802.33 | 53806.95 | 53808.83 | 53831.83 | 53831.63 | 53817.8 | 53802.61 | 53801.31 |
| 694.3015 | 53789.65 | 53869.2 | 53751.81 | 53784.03 | 53830.23 | 53819.55 | 53816.15 | 53807.05 | 53793.73 | 53787.98 | 53836.35 | 53837.35 | 53768.63 | 53815.94 |
| 694.4322 | 53810.86 | 53887.95 | 53741.63 | 53818.15 | 53832.68 | 53813.5 | 53825.03 | 53777.48 | 53811.3 | 53770.03 | 53808.98 | 53819.65 | 53767.32 | 53821.94 |
| 694.563 | 53826.54 | 53870.38 | 53786.44 | 53851.48 | 53859.2 | 53835.68 | 53819.98 | 53761.6 | 53769.93 | 53741.6 | 53806.78 | 53827.18 | 53771.26 | 53826.19 |
| 694.6937 | 53802.54 | 53846.28 | 53773.16 | 53839.43 | 53829.3 | 53843.25 | 53780.1 | 53755.7 | 53771.58 | 53740.98 | 53852.35 | 53812.68 | 53759.08 | 53831.56 |
| 694.8245 | 53762.73 | 53847.38 | 53754.41 | 53784.18 | 53848.2 | 53843.33 | 53741.23 | 53791.28 | 53783.53 | 53738.73 | 53818.53 | 53827.73 | 53791.47 | 53884 |
| 694.9552 | 53789.51 | 53852.58 | 53754.09 | 53753.35 | 53851.18 | 53787.65 | 53780.25 | 53783.85 | 53795.1 | 53740.58 | 53817.4 | 53876.83 | 53781.61 | 53824.31 |
| 695.0859 | 53811.22 | 53844.78 | 53751.69 | 53764.83 | 53833.75 | 53798.33 | 53794.85 | 53783.9 | 53784.4 | 53754.5 | 53831 | 53907.3 | 53826.71 | 53852.88 |
| 695.2167 | 53806 | 53831.43 | 53745.53 | 53819.88 | 53820.45 | 53785.63 | 53789.68 | 53733.7 | 53783.78 | 53760.73 | 53796.65 | 53848.53 | 53844.53 | 53821.31 |
| 695.3474 | 53801.59 | 53845.65 | 53732.13 | 53813.05 | 53838.48 | 53817.08 | 53825.45 | 53714.53 | 53797.4 | 53757.43 | 53813.58 | 53850.65 | 53799.92 | 53796.88 |
| 695.4782 | 53831.92 | 53850.15 | 53755.13 | 53846.63 | 53802.83 | 53873.43 | 53829.4 | 53733.2 | 53806.53 | 53802.88 | 53823.1 | 53828.23 | 53755.32 | 53775.44 |
| 695.6089 | 53837.81 | 53816.65 | 53773.78 | 53822.95 | 53837.1 | 53851.88 | 53812.35 | 53776.4 | 53807.38 | 53775.35 | 53756.33 | 53817.08 | 53745.05 | 53743.31 |
| 695.7396 | 53827.22 | 53847.63 | 53785.03 | 53804.45 | 53849.1 | 53836.5 | 53835.4 | 53750.93 | 53833.15 | 53755.98 | 53795.8 | 53800 | 53799.45 | 53759 |
| 695.8703 | 53795.62 | 53840.95 | 53762.53 | 53797.53 | 53848.28 | 53824.13 | 53824.4 | 53727.68 | 53764.18 | 53787.88 | 53836.03 | 53828.88 | 53757.13 | 53734.06 |
| 696.001 | 53753.59 | 53850.3 | 53777.78 | 53794.5 | 53820.13 | 53876.08 | 53826.13 | 53765.65 | 53767.65 | 53800.65 | 53818.73 | 53838.9 | 53776.68 | 53715.63 |
| 696.1317 | 53797.97 | 53834.9 | 53783.59 | 53805.03 | 53794.88 | 53881.85 | 53864.08 | 53770.75 | 53775.45 | 53774.58 | 53863.53 | 53815.15 | 53767.29 | 53752.25 |
| 696.2624 | 53764.78 | 53849.33 | 53766.41 | 53819.75 | 53820.88 | 53841.58 | 53837.9 | 53756.83 | 53765.75 | 53790.6 | 53815.45 | 53804.28 | 53763.16 | 53795.44 |
| 696.3931 | 53734.27 | 53824.7 | 53741.5 | 53824.8 | 53827.93 | 53868.65 | 53824.85 | 53733.75 | 53769.98 | 53763.68 | 53801.23 | 53814.78 | 53751.89 | 53775.69 |
| 696.5237 | 53784.84 | 53845.33 | 53732.06 | 53824.13 | 53839.3 | 53875.18 | 53770.05 | 53737.43 | 53804.45 | 53784.9 | 53771.35 | 53791.08 | 53750.16 | 53789.44 |
| 696.6544 | 53765.54 | 53809.5 | 53720.56 | 53800.85 | 53838.4 | 53850.93 | 53776.08 | 53749.63 | 53783.28 | 53794.7 | 53792.7 | 53810 | 53754.29 | 53760.56 |
| 696.7851 | 53766 | 53797.35 | 53752.81 | 53791.08 | 53843.05 | 53870.53 | 53781.15 | 53772.98 | 53752.7 | 53811.13 | 53788.2 | 53782.9 | 53746.05 | 53766.31 |
| 696.9157 | 53760.11 | 53808.2 | 53732.5 | 53786.43 | 53829.08 | 53852.38 | 53773.18 | 53765.93 | 53747.38 | 53748 | 53787.93 | 53816.08 | 53755.24 | 53789.06 |
| 697.0464 | 53795.14 | 53816.8 | 53751.91 | 53805.83 | 53802 | 53784.43 | 53790.08 | 53751.05 | 53760.48 | 53752.2 | 53767.68 | 53850.8 | 53779.24 | 53786 |
| 697.1771 | 53772.46 | 53822.18 | 53763.78 | 53816.25 | 53865.53 | 53781.63 | 53803.33 | 53766.13 | 53764.9 | 53798.73 | 53786.58 | 53819.2 | 53785.61 | 53772.69 |
| 697.3077 | 53717.05 | 53846.43 | 53762.91 | 53776.48 | 53840 | 53797.98 | 53885.05 | 53738.75 | 53759.43 | 53740.78 | 53800.38 | 53819.08 | 53785.29 | 53747 |
| 697.4383 | 53691.11 | 53792.68 | 53798.16 | 53711.43 | 53818.15 | 53829.43 | 53860.73 | 53723.75 | 53742.35 | 53777.23 | 53808.03 | 53808.55 | 53765.53 | 53753.94 |
| 697.569 | 53745.84 | 53776.13 | 53774.19 | 53742.88 | 53803.4 | 53838.2 | 53815.15 | 53748.65 | 53779.13 | 53826.7 | 53819.33 | 53814.1 | 53739.87 | 53719.19 |
| 697.6996 | 53793.7 | 53798.1 | 53759.34 | 53765.93 | 53818.4 | 53842.45 | 53835.75 | 53754.38 | 53787.1 | 53767.78 | 53814.03 | 53780.3 | 53742.61 | 53803.81 |
| 697.8302 | 53754.92 | 53793.48 | 53740.88 | 53773 | 53816 | 53808.88 | 53782.98 | 53753.7 | 53785.25 | 53727.1 | 53785.23 | 53774.55 | 53727.08 | 53782.38 |
| 697.9608 | 53745.51 | 53811.9 | 53729.72 | 53778.1 | 53818.73 | 53787.68 | 53755.68 | 53768.18 | 53819.43 | 53740.83 | 53794.7 | 53793.5 | 53768.29 | 53741.06 |
| 698.0914 | 53763 | 53803.98 | 53761.81 | 53740.9 | 53806.3 | 53805.05 | 53803.23 | 53753.38 | 53753.63 | 53724.28 | 53827.73 | 53813.65 | 53737.76 | 53757.31 |
| 698.222 | 53766.22 | 53810.78 | 53743.94 | 53730.75 | 53785.03 | 53774.03 | 53774.83 | 53723.88 | 53743.65 | 53741.03 | 53797.03 | 53779 | 53713.89 | 53719.44 |
| 698.3526 | 53757.14 | 53819.4 | 53726.31 | 53794.03 | 53782.88 | 53802.35 | 53784.93 | 53747.1 | 53755.4 | 53731.18 | 53789.13 | 53764.55 | 53727.66 | 53708.5 |
| 698.4832 | 53733.41 | 53774.4 | 53729.59 | 53771.45 | 53805.58 | 53802.98 | 53731.83 | 53746 | 53767.33 | 53709.33 | 53773.98 | 53767.75 | 53719.95 | 53743.5 |
| 698.6138 | 53737.97 | 53795.25 | 53690.41 | 53777.08 | 53806.75 | 53751.88 | 53726.43 | 53764.1 | 53752.8 | 53727.15 | 53753.28 | 53788.1 | 53756.97 | 53770.19 |
| 698.7444 | 53754.11 | 53765.33 | 53712.16 | 53752.3 | 53773.45 | 53735.98 | 53753.68 | 53759.18 | 53743.85 | 53728.65 | 53746.13 | 53785.78 | 53713.08 | 53736.5 |
| 698.8749 | 53715.92 | 53751.8 | 53691.78 | 53731.5 | 53766.48 | 53708.53 | 53719.9 | 53720.05 | 53747.13 | 53702.73 | 53796.48 | 53784.43 | 53708.29 | 53750.88 |
| 699.0055 | 53723.14 | 53785.48 | 53678.03 | 53709.8 | 53769.2 | 53736.2 | 53720.93 | 53703.18 | 53717.53 | 53729.83 | 53753.55 | 53757.1 | 53701.74 | 53770.19 |
| 699.1361 | 53728.76 | 53767.88 | 53717.03 | 53744.28 | 53742.53 | 53743.05 | 53751.75 | 53676.58 | 53704.38 | 53716.93 | 53744.13 | 53730.15 | 53735.76 | 53714.38 |
| 699.2666 | 53723.11 | 53769.33 | 53697.91 | 53748.6 | 53740.68 | 53742.55 | 53702.88 | 53666.58 | 53733.13 | 53696.8 | 53737.88 | 53741.78 | 53682.79 | 53713.56 |
| 699.3972 | 53710.65 | 53736.1 | 53662.25 | 53717.9 | 53736.45 | 53724.9 | 53707.48 | 53671.7 | 53740.6 | 53680.8 | 53742.4 | 53762.85 | 53695.63 | 53759.19 |
| 699.5277 | 53666.76 | 53725.23 | 53637.19 | 53682 | 53727.08 | 53741.05 | 53686.98 | 53661 | 53700.78 | 53676.93 | 53711.38 | 53740 | 53689.84 | 53747.69 |
| 699.6583 | 53669.41 | 53703.55 | 53671 | 53715.68 | 53740.5 | 53733.8 | 53673.88 | 53654.35 | 53661.93 | 53664.25 | 53676.68 | 53730.7 | 53666.26 | 53718.88 |
| 699.7888 | 53668.38 | 53695.75 | 53648.66 | 53681.8 | 53728.78 | 53741.15 | 53653.35 | 53652.35 | 53655.78 | 53648.43 | 53703.18 | 53698.73 | 53682.63 | 53708.44 |
| 699.9193 | 53662.68 | 53693.83 | 53608.75 | 53679.48 | 53685 | 53704.33 | 53662.23 | 53639.03 | 53671.55 | 53645.8 | 53709.85 | 53691.38 | 53670.84 | 53671.94 |
| 700.0498 | 53690.81 | 53693.43 | 53611.09 | 53673.93 | 53670.53 | 53691.18 | 53667.23 | 53666.93 | 53654.3 | 53660.73 | 53690.43 | 53712.6 | 53649.11 | 53668.94 |
| 700.1804 | 53684.22 | 53687.55 | 53628.69 | 53657.53 | 53676.53 | 53698.8 | 53659.6 | 53609.48 | 53664 | 53638.68 | 53647.5 | 53687.48 | 53637.97 | 53670 |
| 700.3109 | 53660.92 | 53683.8 | 53621.78 | 53655.8 | 53660.98 | 53653.65 | 53643.9 | 53608.58 | 53637.83 | 53628.65 | 53660.48 | 53681.6 | 53626.13 | 53658.69 |
| 700.4414 | 53631.35 | 53636.75 | 53583.94 | 53645.1 | 53637.53 | 53635.88 | 53640.7 | 53597.9 | 53634.38 | 53597.93 | 53645.43 | 53653.65 | 53602.79 | 53645.06 |
| 700.5718 | 53601.11 | 53621.43 | 53556.91 | 53611.5 | 53610.78 | 53626.03 | 53581.93 | 53590.33 | 53613.15 | 53570.43 | 53620.3 | 53624.75 | 53599.42 | 53647.25 |
| 700.7023 | 53570.89 | 53598.88 | 53535.13 | 53578.45 | 53596.68 | 53577.88 | 53557.28 | 53563.65 | 53590.03 | 53552.55 | 53593.15 | 53612.33 | 53586.61 | 53599.88 |
| 700.8328 | 53549.03 | 53575.45 | 53525.97 | 53561.48 | 53583.43 | 53568.33 | 53549.58 | 53546.38 | 53571.05 | 53546.8 | 53585.98 | 53602.48 | 53570.42 | 53588.88 |
| 700.9633 | 53546.3 | 53568.55 | 53526.88 | 53558 | 53574.45 | 53563.4 | 53546.45 | 53538.63 | 53567.53 | 53542.9 | 53590.9 | 53598.85 | 53570.24 | 53585.13 |
| 701.0938 | 53534.11 | 53565.2 | 53521.06 | 53548.53 | 53568.05 | 53556.6 | 53542.33 | 53537.83 | 53557.3 | 53534.93 | 53584.08 | 53591.03 | 53564.11 | 53579.69 |
| 701.2242 | 53530.68 | 53561.15 | 53514.53 | 53546.1 | 53566.05 | 53553.25 | 53536.75 | 53534 | 53558.3 | 53530.95 | 53579.95 | 53589.28 | 53561.55 | 53579.56 |
| 701.3547 | 53534.95 | 53564.53 | 53518.19 | 53547.6 | 53568.43 | 53556.33 | 53539.4 | 53532.18 | 53559.28 | 53536.33 | 53585.15 | 53590.33 | 53567 | 53582.56 |
| 701.4852 | 53535.24 | 53563.63 | 53515.31 | 53546.08 | 53568.2 | 53555.3 | 53536.13 | 53534.4 | 53559.55 | 53532.78 | 53583.03 | 53589.1 | 53563.92 | 53578.69 |
| 701.6156 | 53529.24 | 53557.3 | 53512.63 | 53548.9 | 53562.88 | 53551.18 | 53534.45 | 53538.35 | 53560.55 | 53526.25 | 53585.93 | 53584.6 | 53564.18 | 53580.38 |
| 701.746 | 53531.59 | 53559.98 | 53513.34 | 53545.13 | 53562.93 | 53552.48 | 53536.95 | 53530.73 | 53554.3 | 53533.4 | 53579.08 | 53588.1 | 53559.26 | 53576.63 |
| 701.8765 | 53529.43 | 53557.13 | 53513.44 | 53542 | 53562.35 | 53550.45 | 53532.1 | 53527.8 | 53554.98 | 53527.33 | 53577.48 | 53584.65 | 53557.92 | 53574.44 |
| 702.0069 | 53529.32 | 53558.9 | 53512.94 | 53543.38 | 53563.73 | 53552.58 | 53533.13 | 53529.8 | 53552.1 | 53526.43 | 53578.05 | 53588.73 | 53556.92 | 53577.06 |
| 702.1373 | 53525.59 | 53555.05 | 53519.22 | 53541.55 | 53559.78 | 53549 | 53529.68 | 53524.83 | 53549.75 | 53527.98 | 53576.13 | 53580.6 | 53558.55 | 53567.56 |
| 702.2678 | 53532.22 | 53556.33 | 53515.56 | 53541.7 | 53564.28 | 53550.65 | 53532.35 | 53529.98 | 53552.65 | 53530.73 | 53575.28 | 53586 | 53557.24 | 53574.5 |
| 702.3982 | 53532 | 53555.28 | 53511.13 | 53540.4 | 53561.78 | 53545.25 | 53533.4 | 53530.28 | 53551.7 | 53528.23 | 53577.83 | 53588.93 | 53557.05 | 53575 |
| 702.5286 | 53530.62 | 53557.13 | 53512.13 | 53543.43 | 53563.35 | 53549.98 | 53535.63 | 53524.53 | 53550.7 | 53530.5 | 53579.35 | 53585.95 | 53558.55 | 53578.06 |
| 702.659 | 53527.32 | 53555.2 | 53510.75 | 53540.75 | 53561.43 | 53545.8 | 53531.9 | 53526.55 | 53551.95 | 53526.48 | 53575.95 | 53582.5 | 53553.66 | 53576.31 |
| 702.7894 | 53524.95 | 53554.38 | 53511.97 | 53539.55 | 53556.45 | 53544.15 | 53532.28 | 53524.15 | 53549.23 | 53526.18 | 53572.83 | 53581.65 | 53557.71 | 53570.63 |
| 702.9197 | 53527.84 | 53557.83 | 53512.38 | 53544.48 | 53562.6 | 53547.83 | 53536.1 | 53526.9 | 53552.4 | 53529.28 | 53580.33 | 53586.08 | 53561.16 | 53573.75 |
| 703.0501 | 53527.41 | 53556.3 | 53511.47 | 53541.2 | 53561 | 53550.75 | 53532.55 | 53528.1 | 53553.08 | 53527.95 | 53577.63 | 53587.3 | 53561.16 | 53571.25 |
| 703.1805 | 53528.14 | 53559.43 | 53517.03 | 53546.33 | 53566.43 | 53553.25 | 53539.23 | 53530.4 | 53553.98 | 53531.45 | 53580.33 | 53590.28 | 53562.16 | 53579.38 |
| 703.3109 | 53530.38 | 53558.3 | 53513.88 | 53545.45 | 53562.55 | 53550.23 | 53533 | 53531.83 | 53552.08 | 53531.33 | 53578.58 | 53584.33 | 53559.92 | 53572.81 |
| 703.4412 | 53529.76 | 53555.35 | 53514.31 | 53544.75 | 53562.13 | 53548.08 | 53535.45 | 53529.43 | 53552.8 | 53530.73 | 53578 | 53588.28 | 53558.97 | 53576.88 |
| 703.5716 | 53528.65 | 53557.95 | 53513.28 | 53543.13 | 53565.15 | 53550.7 | 53537.33 | 53529.55 | 53553.1 | 53530.75 | 53579.93 | 53587.63 | 53561.89 | 53577.19 |
| 703.702 | 53528.05 | 53551.05 | 53512.56 | 53540.23 | 53559.5 | 53544.28 | 53531.58 | 53526.6 | 53549.38 | 53527.33 | 53574.88 | 53582.43 | 53559.21 | 53576.19 |
| 703.8323 | 53531.19 | 53557.75 | 53515.34 | 53546.68 | 53565.63 | 53552.83 | 53534.25 | 53529.9 | 53555.15 | 53531.28 | 53580.93 | 53586.43 | 53563.26 | 53575.5 |
| 703.9627 | 53523.11 | 53550.1 | 53506.56 | 53542.83 | 53555.85 | 53543.65 | 53524.93 | 53525.35 | 53548.28 | 53524.9 | 53573.2 | 53584.73 | 53555.66 | 53573 |

**Spectral Range 3: 665 – 800 nm**


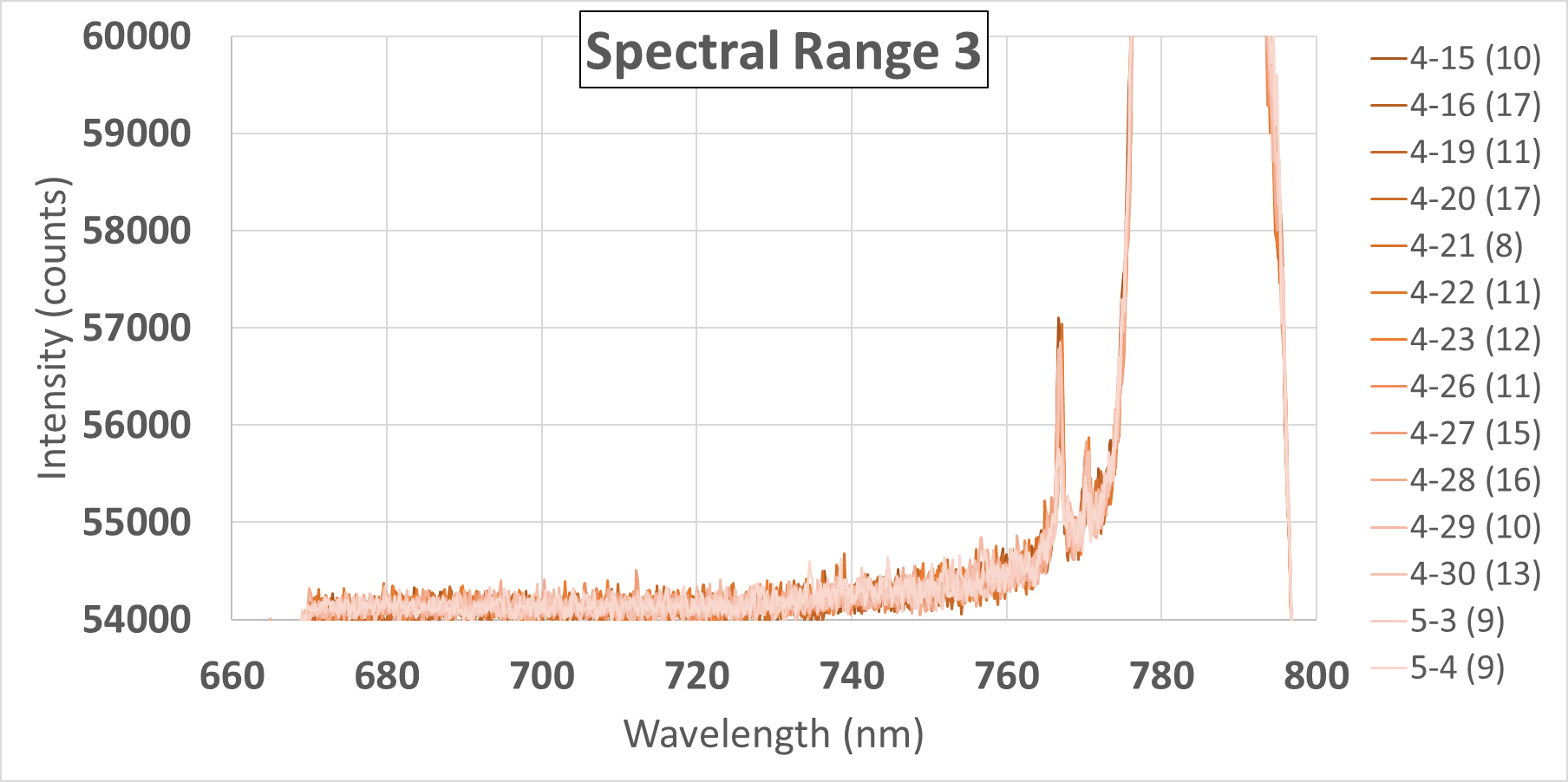

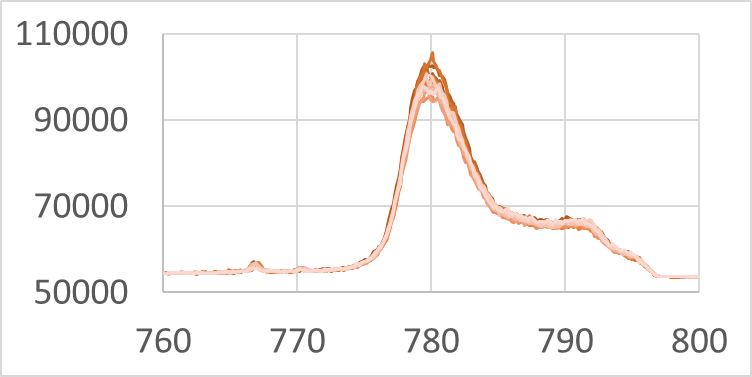


**Figure S3:** Range 3 - Data collected between 4-15 and 5-4 showing potassium emission lines. Inset: Broadband luminescence covering the 775 – 795 nm range. This broad feature appears to originate from the optics.

**Table S3:** Range 3 - Column labels indicate the collection date and number of useable spectra included in the average.

| **wL(nm)** | **4-15 (10)** | **4-16 (17)** | **4-19 (11)** | **4-20 (17)** | **4-21 (8)** | **4-22 (11)** | **4-23 (12)** | **4-26 (11)** | **4-27 (15)** | **4-28 (16)** | **4-29 (10)** | **4-30 (13)** | **5-3 (9)** | **5-4 (9)** |
| --- | --- | --- | --- | --- | --- | --- | --- | --- | --- | --- | --- | --- | --- | --- |
| 664.9585 | 53885.1 | 53862.24 | 53863.36 | 53906.53 | 53882 | 53867.82 | 53932.42 | 53879.64 | 53876.6 | 54014 | 53908.9 | 53936.15 | 53924.78 | 53931.89 |
| 665.0905 | 53831.7 | 53892.65 | 53921.45 | 53892 | 53929.75 | 53836.18 | 53907.5 | 53840.91 | 53815.2 | 53885.5 | 53865.5 | 53907.15 | 53930.44 | 53906.11 |
| 665.2225 | 53847.2 | 53872.29 | 53969.18 | 53848.41 | 53885.38 | 53894 | 53902.67 | 53890.36 | 53876.07 | 53847.5 | 53895 | 53901 | 53883.67 | 53887 |
| 665.3545 | 53855.7 | 53863.94 | 53859.18 | 53868.24 | 53872.13 | 53882.36 | 53928.92 | 53882.91 | 53890.53 | 53815.75 | 53911.2 | 53851.23 | 53863 | 53877.78 |
| 665.4865 | 53856.4 | 53848.29 | 53830.09 | 53883.65 | 53851.88 | 53867.18 | 53932.08 | 53878.64 | 53883.53 | 53843.63 | 53905.7 | 53846.15 | 53870.78 | 53886.78 |
| 665.6185 | 53852.6 | 53837.12 | 53833.18 | 53902.06 | 53824.75 | 53860 | 53924.08 | 53870.91 | 53862.93 | 53865.63 | 53907.3 | 53856.69 | 53882.78 | 53893.78 |
| 665.7505 | 53846.3 | 53841.76 | 53828 | 53908.82 | 53817.13 | 53858.36 | 53914 | 53865.27 | 53859.93 | 53875.13 | 53902.3 | 53878.38 | 53882 | 53907 |
| 665.8825 | 53841.7 | 53837.82 | 53814.36 | 53908.71 | 53807.25 | 53856.73 | 53900.25 | 53858.09 | 53855.47 | 53861.75 | 53887.1 | 53883.77 | 53884.78 | 53907 |
| 666.0145 | 53836.2 | 53839.94 | 53813.27 | 53906.76 | 53800.5 | 53856.73 | 53901.25 | 53858.73 | 53860.2 | 53880.75 | 53890 | 53883.85 | 53884.33 | 53919 |
| 666.1465 | 53835 | 53836.18 | 53806.91 | 53901.06 | 53801.13 | 53852.36 | 53887.42 | 53851.27 | 53855.13 | 53878.13 | 53883.3 | 53885.31 | 53881.11 | 53908.56 |
| 666.2785 | 53830.5 | 53837.65 | 53805.91 | 53902.53 | 53803 | 53853.91 | 53893.33 | 53858.09 | 53850.87 | 53875.63 | 53878.5 | 53888.85 | 53875.44 | 53902.89 |
| 666.4105 | 53831.4 | 53837.41 | 53801.45 | 53894.71 | 53798.63 | 53852.82 | 53877.83 | 53852.09 | 53844.87 | 53870.38 | 53879.5 | 53886.46 | 53875.44 | 53905.56 |
| 666.5425 | 53824.8 | 53838.47 | 53798.82 | 53890.12 | 53799.88 | 53844.45 | 53881.33 | 53845.73 | 53849.13 | 53882.5 | 53873.2 | 53886.31 | 53876.67 | 53904.11 |
| 666.6745 | 53824.3 | 53828.12 | 53794.18 | 53887.24 | 53792.38 | 53842.55 | 53879.75 | 53848.27 | 53845.47 | 53875.75 | 53870 | 53878.92 | 53866.44 | 53903.33 |
| 666.8065 | 53831.7 | 53833.82 | 53799.36 | 53897.12 | 53802.25 | 53848.55 | 53882.92 | 53842 | 53851.93 | 53877.5 | 53865.8 | 53891.46 | 53869.33 | 53901.78 |
| 666.9385 | 53828.6 | 53840 | 53800.73 | 53897.35 | 53806.63 | 53857.82 | 53879.33 | 53853.09 | 53848.07 | 53874.25 | 53877.9 | 53886.92 | 53879.78 | 53907.56 |
| 667.0705 | 53810 | 53824.29 | 53781.73 | 53875.71 | 53787.5 | 53836.09 | 53867.5 | 53840.55 | 53837.4 | 53860.88 | 53866 | 53875.38 | 53868.22 | 53898.44 |
| 667.2025 | 53820.2 | 53824.29 | 53789.45 | 53875.24 | 53793.5 | 53837.55 | 53868.33 | 53841.18 | 53836.67 | 53863.88 | 53866.5 | 53878.54 | 53855.22 | 53899 |
| 667.3345 | 53817.8 | 53823.94 | 53787.64 | 53877.59 | 53785.88 | 53833.36 | 53865.5 | 53829.27 | 53831.73 | 53864.88 | 53863.9 | 53877.85 | 53865.44 | 53887.89 |
| 667.4665 | 53819.6 | 53824.88 | 53787.82 | 53879.18 | 53785.88 | 53839.18 | 53868.83 | 53845.55 | 53839.27 | 53863 | 53863.9 | 53873.08 | 53865.67 | 53900.33 |
| 667.5985 | 53811.2 | 53818.47 | 53782.18 | 53871.71 | 53784.63 | 53827.09 | 53862.17 | 53830.73 | 53834.07 | 53861 | 53869.3 | 53874.69 | 53859.89 | 53888.67 |
| 667.7305 | 53812.4 | 53821.29 | 53782.09 | 53880.24 | 53792.63 | 53830.18 | 53863.92 | 53835.27 | 53837.4 | 53862.5 | 53863.6 | 53875.23 | 53861.44 | 53890.22 |
| 667.8626 | 53814.4 | 53822.53 | 53784.36 | 53878.47 | 53789.75 | 53829.91 | 53858.83 | 53831.64 | 53830.87 | 53862 | 53857.6 | 53865.62 | 53866.56 | 53890 |
| 667.9945 | 53814.9 | 53820.71 | 53785.27 | 53876.65 | 53792.13 | 53840.27 | 53868.33 | 53832.45 | 53836.33 | 53860 | 53859.5 | 53869.31 | 53865.67 | 53888.33 |
| 668.1265 | 53813.5 | 53815.47 | 53780.55 | 53873.41 | 53777.13 | 53836.27 | 53861.5 | 53828.18 | 53827.93 | 53855.13 | 53855.8 | 53863.46 | 53862.11 | 53885.56 |
| 668.2585 | 53819.2 | 53821.41 | 53780.91 | 53873.06 | 53782.75 | 53836.45 | 53863.17 | 53832.36 | 53833.8 | 53862.63 | 53859.1 | 53874.38 | 53861.78 | 53888.22 |
| 668.3905 | 53820.7 | 53820.53 | 53786.55 | 53880.41 | 53791.13 | 53840.18 | 53863 | 53837.73 | 53832.47 | 53867.13 | 53864 | 53870.92 | 53861.56 | 53894.67 |
| 668.5225 | 53819.8 | 53817.29 | 53781.45 | 53880.76 | 53793 | 53841.55 | 53867.58 | 53832.55 | 53841 | 53865.63 | 53861.7 | 53875.54 | 53866.22 | 53890.22 |
| 668.6545 | 53848.5 | 53828.06 | 53790.27 | 53900.88 | 53803.5 | 53860.82 | 53876.42 | 53854 | 53863.87 | 53882.5 | 53875.9 | 53893.38 | 53888.89 | 53903.56 |
| 668.7866 | 53903.4 | 53845.24 | 53819.18 | 53938 | 53867.25 | 53898.91 | 53936 | 53901.18 | 53981.27 | 53925.38 | 53897 | 53945.08 | 53904.78 | 53981.44 |
| 668.9185 | 53968.6 | 53874.18 | 53823.73 | 53966.65 | 53924.88 | 53958.55 | 53986.08 | 53971.73 | 53898.73 | 53953.25 | 53983 | 53977.77 | 53954.33 | 53992.67 |
| 669.0505 | 53999.7 | 53892.35 | 53880.82 | 54005.94 | 53919.88 | 54003 | 53986.83 | 53937.73 | 53921 | 53971.25 | 53957.7 | 53971.54 | 54095.33 | 53975.78 |
| 669.1826 | 54058.4 | 53897.88 | 53942.45 | 53971.82 | 53933.13 | 54047.64 | 53961.25 | 53933.36 | 53921.6 | 54012 | 54050.1 | 54010 | 53972.33 | 53966.11 |
| 669.3145 | 53999.3 | 53905.12 | 53989.73 | 54010.41 | 53971.63 | 54020.27 | 54028.83 | 53937.18 | 53978.4 | 53991.63 | 54014.2 | 53983.62 | 53955.78 | 54008.89 |
| 669.4465 | 54036.7 | 53924 | 53937.73 | 54063.06 | 53966.75 | 54024.55 | 53996.67 | 53980.55 | 53978.33 | 53990.13 | 53996.8 | 53988.15 | 54012.67 | 54013.67 |
| 669.5786 | 53924 | 53971.12 | 53973.55 | 54080.41 | 54024.63 | 54025.82 | 54097.67 | 53998.18 | 54020.47 | 54035.5 | 54009.3 | 53974.31 | 54000 | 54063.44 |
| 669.7106 | 53955.7 | 54050.06 | 53947.18 | 54047.47 | 54109.88 | 54035.18 | 54057.17 | 54012.27 | 53960.53 | 54111.88 | 54044.1 | 53990.77 | 54036.56 | 54151.11 |
| 669.8425 | 54070.2 | 54056.53 | 53935.36 | 54104 | 54134.38 | 53999.55 | 54185.58 | 53979.91 | 54015.93 | 54214.88 | 53988.1 | 53996.85 | 54058.56 | 54108.56 |
| 669.9746 | 54026.9 | 54029.29 | 53939 | 54128.29 | 54140.25 | 54012.18 | 54043.42 | 53957.91 | 54117.2 | 54322 | 53976.8 | 54066.77 | 54011.11 | 54137.67 |
| 670.1066 | 54087.7 | 53990.53 | 53939.27 | 54176.29 | 54015 | 54092.64 | 54044.58 | 53989.18 | 54142.73 | 54282.13 | 53990.8 | 54078.31 | 54022.56 | 54088.89 |
| 670.2386 | 54103.7 | 54013.18 | 53910.91 | 54089 | 54043.25 | 54095.27 | 54090.92 | 54094.82 | 54055 | 54273.38 | 54024.8 | 54057 | 54090.89 | 54114 |
| 670.3705 | 54111.7 | 54014.18 | 54064.55 | 54110.88 | 54113.75 | 54109.73 | 54133.75 | 54070.09 | 54089.47 | 54070.38 | 54076.8 | 54039.54 | 54151.22 | 54099.33 |
| 670.5026 | 54100.5 | 54036.65 | 54027.73 | 54224.47 | 54113.25 | 54206.27 | 54156.67 | 54081.27 | 54046.93 | 54153.13 | 54141.7 | 54068.54 | 54130.44 | 54089.11 |
| 670.6346 | 54141.8 | 54145.82 | 54024.27 | 54160.47 | 54051.38 | 54116.82 | 54123.5 | 54126.64 | 54085.13 | 54064.88 | 54118.8 | 54185.69 | 54164.22 | 54098.44 |
| 670.7665 | 54126.9 | 54042.71 | 53996.45 | 54139.18 | 54078.5 | 54067.09 | 54149.33 | 54047.91 | 54165.67 | 54153.75 | 54081.1 | 54066.23 | 54124.22 | 54145.67 |
| 670.8986 | 54202.3 | 54025.88 | 54022.18 | 54191.65 | 54103.25 | 54245.91 | 54159.83 | 54065.27 | 54078.93 | 54090.5 | 54067.5 | 54082 | 54065.67 | 54103 |
| 671.0306 | 54108.8 | 54072.12 | 53986.64 | 54229.53 | 54155.5 | 54135.91 | 54096.83 | 53998.36 | 54079.6 | 54160 | 54037.8 | 54144.77 | 54048.56 | 54110.78 |
| 671.1626 | 54153.5 | 54143.82 | 54029.55 | 54178.06 | 54315.63 | 54077.27 | 54056.42 | 54079.82 | 54064.93 | 54053.5 | 54191.8 | 54122.77 | 54077 | 54094.78 |
| 671.2946 | 54027.5 | 54112.82 | 54060.73 | 54214.18 | 54090.5 | 54103.82 | 54124.83 | 53977.18 | 54095 | 54030.13 | 54075.7 | 54119.46 | 54076.56 | 54109.56 |
| 671.4266 | 54105.4 | 54080.12 | 54154.36 | 54170.29 | 54063.88 | 54147.27 | 54160.17 | 54019.82 | 54104.2 | 54162.75 | 54158.3 | 54137.85 | 54141.56 | 54147.11 |
| 671.5586 | 54075.3 | 54028.71 | 54149.27 | 54126.29 | 54060 | 54143.82 | 54042 | 54089.36 | 54116.87 | 54160.88 | 54090.7 | 54097.62 | 54077.44 | 54182.56 |
| 671.6906 | 54165 | 54103.94 | 53960.73 | 54149.82 | 54019.63 | 54227.27 | 54058.33 | 54015.18 | 54120.53 | 54095.5 | 54018.7 | 54150.46 | 54134 | 54132.44 |
| 671.8226 | 54189.9 | 54080.35 | 53932.45 | 54120.71 | 54054.38 | 54185.18 | 54199 | 54075.64 | 54083.4 | 54056 | 54047.3 | 54211.15 | 54121.78 | 54152.33 |
| 671.9546 | 54075.9 | 54039.94 | 53970.18 | 54188.76 | 53964 | 54103.64 | 54138.67 | 54108.36 | 54137.27 | 54149 | 54022.4 | 54105.08 | 54072.11 | 54212.22 |
| 672.0866 | 54105.3 | 54030.94 | 54035.18 | 54254.65 | 53925.38 | 54074.91 | 54118.58 | 54080.91 | 54124.13 | 54121.88 | 54062.4 | 54102.92 | 54064.44 | 54232 |
| 672.2186 | 54059.8 | 54036.24 | 54144.73 | 54226.41 | 53992.75 | 54036.64 | 54083.17 | 54003.82 | 54061.13 | 54061.88 | 54038.2 | 54111.85 | 54131.56 | 54150.11 |
| 672.3506 | 54070.4 | 54055.94 | 54078.45 | 54143 | 54030.63 | 54035.09 | 54108.42 | 54026.27 | 54089.8 | 53972.25 | 54085.1 | 54084.85 | 54162.78 | 54195.22 |
| 672.4826 | 54141.8 | 54057.94 | 53990.82 | 54171.59 | 54002.5 | 54020.09 | 54086.17 | 54047.45 | 54091.53 | 54013.75 | 54053.9 | 54081.31 | 54214.11 | 54198.33 |
| 672.6146 | 54185.6 | 54036.29 | 53961.82 | 54211.47 | 54035 | 53997.09 | 54109 | 54001.45 | 54100.67 | 54004.13 | 54110.4 | 54124.69 | 54180 | 54160.67 |
| 672.7466 | 54104.8 | 54042.76 | 53944.36 | 54187.47 | 54100.88 | 54028.09 | 54144.42 | 54095.64 | 54115.33 | 54081.13 | 54124.4 | 54106.15 | 54130.33 | 54097.22 |
| 672.8786 | 54216.4 | 54094.94 | 54009.64 | 54162.41 | 54119.75 | 54021.91 | 54140.33 | 54063.18 | 54035.8 | 54080 | 54084.6 | 54125.15 | 54112.67 | 54112.33 |
| 673.0106 | 54269.3 | 54054.71 | 54071 | 54183.24 | 54092.88 | 54009.18 | 54103.92 | 54064.18 | 54075.93 | 54069.63 | 54113.6 | 54098.77 | 54050.89 | 54192.56 |
| 673.1426 | 54162.4 | 54152.06 | 54057.45 | 54164.59 | 54056.38 | 54060.91 | 54107 | 54135.09 | 54044.53 | 54052.5 | 54108.2 | 54084.92 | 54170 | 54103.56 |
| 673.2746 | 54192 | 54130.71 | 54038.45 | 54132.41 | 53991.13 | 54065.82 | 54118.33 | 54044.91 | 54078.07 | 54044.5 | 54122.8 | 54179.23 | 54096 | 54091 |
| 673.4066 | 54105.1 | 54022.47 | 54065.36 | 54186.53 | 54045 | 54003.18 | 54157 | 54080.91 | 53992.4 | 54111.63 | 54138.1 | 54168.31 | 54081.33 | 54098.33 |
| 673.5386 | 54075 | 54047.24 | 54014.27 | 54223.12 | 54055.38 | 54114.64 | 54165.75 | 54034.36 | 54028.53 | 54089.88 | 54166.1 | 54205.85 | 54188.56 | 54077.33 |
| 673.6706 | 54135.2 | 54107.18 | 54042.73 | 54186.65 | 54132.75 | 54148 | 54171.67 | 54097.91 | 54088.47 | 54102 | 54160.2 | 54157.15 | 54166.11 | 54051.44 |
| 673.8026 | 54140.9 | 54196.18 | 54087 | 54145.88 | 54137.25 | 54092 | 54142.83 | 54098.64 | 54064.33 | 54190.75 | 54144.3 | 54212.77 | 54019.67 | 54154.56 |
| 673.9346 | 54083.2 | 54171.06 | 54006.18 | 54167.59 | 54065.13 | 54021.64 | 54123.67 | 54085.36 | 54073.67 | 54160.25 | 54162.6 | 54212 | 54043.11 | 54214 |
| 674.0666 | 54175.2 | 54208.24 | 54062.45 | 54157.35 | 54045.75 | 54086.36 | 54109.17 | 54134.18 | 54071 | 54127.25 | 54220.7 | 54132.08 | 54147.33 | 54118.11 |
| 674.1986 | 54161.7 | 54124.35 | 53946.45 | 54161.94 | 54140.25 | 54135.45 | 54118.5 | 54194.82 | 54090.33 | 54110.63 | 54155.2 | 54127.46 | 54073.78 | 54136.67 |
| 674.3306 | 54102.8 | 54209.06 | 53973.18 | 54110.65 | 54175 | 54105.27 | 54252.83 | 54181.55 | 54145.47 | 54050 | 54092.6 | 54051 | 54088.44 | 54149 |
| 674.4627 | 54128.6 | 54101.41 | 53975.73 | 54106.35 | 54106.75 | 54325.55 | 54144.67 | 54108.09 | 54147.27 | 53983.25 | 54182 | 54048.92 | 54098.89 | 54093.89 |
| 674.5946 | 54145.8 | 54014 | 54041.36 | 54152.18 | 54051.88 | 54078.73 | 54129.42 | 54065.73 | 54179.6 | 54040.75 | 54157.6 | 54208.38 | 54078.33 | 54174.56 |
| 674.7266 | 54080.9 | 54070.06 | 53982.73 | 54188.12 | 54226.63 | 53981.36 | 54112.25 | 54052.27 | 54155.93 | 54044.88 | 54231.1 | 54273.08 | 54141.11 | 54197.22 |
| 674.8586 | 54166.9 | 54110.59 | 54039.73 | 54160.82 | 54079 | 54072 | 54085.67 | 54134.55 | 54131.47 | 54050.5 | 54164.9 | 54254.08 | 54162.22 | 54198.44 |
| 674.9906 | 54087.7 | 54028.94 | 54028.27 | 54172.12 | 53980.75 | 54187.91 | 54118.75 | 54120.27 | 54164.87 | 54048.75 | 54088.2 | 54208.69 | 54068.67 | 54167.89 |
| 675.1226 | 54112.6 | 54067.59 | 53978 | 54121 | 54029 | 54126.55 | 54258.17 | 54181.45 | 54126.13 | 54095.38 | 54102.6 | 54143.15 | 54148.78 | 53998.22 |
| 675.2546 | 54105.5 | 54035.65 | 53932.91 | 54181.65 | 54055.13 | 54102.55 | 54200.83 | 54152.09 | 54064.33 | 54072.88 | 54047.9 | 54068.62 | 54120.33 | 54081.11 |
| 675.3867 | 54064.8 | 54048.41 | 53956.55 | 54104.94 | 54134.25 | 54062.36 | 54137.25 | 54183 | 54124.4 | 54077.38 | 54126.8 | 54073.77 | 54153.33 | 54082 |
| 675.5186 | 54108.5 | 54030.18 | 54010.27 | 54149.06 | 54079.38 | 54023.64 | 54140.58 | 54152.36 | 54105.33 | 54075 | 54045 | 54015.92 | 54172 | 54136.22 |
| 675.6506 | 54181.6 | 54028.94 | 54041.64 | 54145.53 | 54086 | 54055.27 | 54157.83 | 54109.64 | 54070.4 | 54075.75 | 54085 | 54016.23 | 54233.78 | 54097.78 |
| 675.7827 | 54071.6 | 54062.59 | 54039.64 | 54202.71 | 54149 | 54140.45 | 54174.5 | 54113.82 | 54070.33 | 54021.63 | 54070.9 | 54041.23 | 54029.33 | 54185.78 |
| 675.9146 | 54144 | 54058.53 | 54014.82 | 54155 | 54078.88 | 54242.73 | 54175.67 | 54183.27 | 54065.07 | 54127.25 | 54070.2 | 54110.85 | 54021.56 | 54085.56 |
| 676.0466 | 54257.6 | 54052.71 | 53976.55 | 54129.71 | 54037.75 | 54177.45 | 54162.5 | 54142.09 | 54038.33 | 54147.75 | 54098.8 | 54196.15 | 54006.89 | 54082 |
| 676.1787 | 54190.5 | 54043.65 | 54008.45 | 54179.47 | 54156 | 54112.18 | 54156.67 | 54042.09 | 54109 | 54158.75 | 54136.7 | 54264.08 | 54035.56 | 54040.89 |
| 676.3107 | 54158.5 | 53993.41 | 54091.18 | 54148.94 | 54092.38 | 54123 | 54110.67 | 54085.09 | 54115.6 | 54077.25 | 54115.4 | 54161.69 | 54064.67 | 54098.44 |
| 676.4426 | 54075.4 | 54023.82 | 53994.91 | 54090.71 | 53999.75 | 54116.36 | 54211.5 | 54150.09 | 54096.53 | 54103.38 | 54161.3 | 54152.77 | 54095.89 | 54230.78 |
| 676.5747 | 54143.4 | 54044.47 | 54101.27 | 54116.71 | 54099.38 | 54094.91 | 54175.92 | 54093.64 | 54067.27 | 54065.88 | 54119.3 | 54058.85 | 54059.22 | 54115.22 |
| 676.7067 | 54153.4 | 54029.24 | 54000.27 | 54133.29 | 54175.63 | 54145.36 | 54072 | 54100.64 | 54049.27 | 54016.13 | 54121.6 | 54151.31 | 54162 | 54175.33 |
| 676.8387 | 54107.8 | 54053.76 | 54007.09 | 54115.12 | 54112.63 | 54115.64 | 54126.75 | 54126.27 | 54082.73 | 54010.75 | 54086 | 54133.77 | 54215.78 | 54143.67 |
| 676.9706 | 54009.3 | 54034.41 | 53964.73 | 54127.41 | 54098 | 54125.36 | 54251.5 | 54081.09 | 54165 | 54045.88 | 54119.7 | 54230.31 | 54036.56 | 54128.11 |
| 677.1027 | 54043.9 | 54139 | 54066.91 | 54155.65 | 54072.63 | 54169 | 54103.08 | 54129.64 | 54155.53 | 54054.25 | 54057.7 | 54241.77 | 54015.33 | 54182.56 |
| 677.2347 | 54106.9 | 54170.47 | 54062.82 | 54131.94 | 54113.88 | 54114.18 | 54095.42 | 54134.45 | 54188.27 | 54031.38 | 54091.7 | 54152.92 | 54104.78 | 54182.89 |
| 677.3666 | 54154.3 | 54139.18 | 53979.91 | 54156.18 | 54061.88 | 54126.82 | 54074.58 | 54084.91 | 54151.67 | 54066 | 54083.5 | 54176.46 | 54083.44 | 54120.33 |
| 677.4987 | 54186.2 | 54145.18 | 53957.09 | 54146.88 | 54058.5 | 54165.36 | 54143.42 | 54033.55 | 54181.27 | 54069.75 | 54053.2 | 54136.31 | 54131.78 | 54166.89 |
| 677.6307 | 54155.9 | 54131.12 | 53954 | 54162.12 | 54105.63 | 54086.27 | 54114.83 | 53995.91 | 54150.47 | 54064.5 | 54173.3 | 54176.38 | 54165.78 | 54137.44 |
| 677.7627 | 54102.3 | 54057.24 | 54038.45 | 54212 | 54080.25 | 54057.64 | 54195.58 | 54051.73 | 54060.8 | 54051.25 | 54179.1 | 54272.77 | 54118.56 | 54143.89 |
| 677.8947 | 54052.1 | 54013.88 | 54087.09 | 54216.35 | 54025.38 | 54120.09 | 54207.33 | 54121.64 | 54143.93 | 54102.75 | 54114.5 | 54243.69 | 54230.78 | 54152.67 |
| 678.0267 | 54105.8 | 54035.71 | 54124.91 | 54171.94 | 54008.38 | 54152.09 | 54126.83 | 54064.64 | 54135.33 | 54081.63 | 54125.1 | 54145.38 | 54173.11 | 54127.78 |
| 678.1587 | 54172.7 | 54056.35 | 54135.45 | 54171.88 | 53968.75 | 54106.36 | 54200.67 | 54091.36 | 54165.4 | 54149 | 54228.8 | 54216.77 | 54056.56 | 54123.56 |
| 678.2907 | 54121.2 | 54140.41 | 54049.09 | 54164.24 | 54028.88 | 54187.73 | 54146.58 | 54071.91 | 54183.73 | 54078.38 | 54189 | 54164.38 | 54121.78 | 54060.11 |
| 678.4227 | 54171.7 | 54114.24 | 53991.91 | 54240.24 | 54013 | 54193.82 | 54124.75 | 54066.91 | 54124.33 | 54092.75 | 54088.7 | 54166.77 | 54004.89 | 54119.89 |
| 678.5547 | 54096.2 | 54063.24 | 54034.18 | 54176.88 | 54026.63 | 54234.64 | 54202.33 | 54078.55 | 54189.27 | 54166.5 | 54087.9 | 54127.77 | 53994.11 | 54194.67 |
| 678.6867 | 54149.5 | 54167.53 | 54011.09 | 54089.47 | 54019.88 | 54141.82 | 54167.17 | 54158.55 | 54106.33 | 54143.38 | 54168 | 54107.62 | 54053.56 | 54244.11 |
| 678.8187 | 54021.9 | 54206.59 | 53936.45 | 54131.24 | 54233.75 | 54076.18 | 54111.25 | 54148.82 | 54128 | 54201.63 | 54172.7 | 54074.69 | 54052.56 | 54189.33 |
| 678.9507 | 54115.5 | 54234.41 | 54051.45 | 54175.35 | 54165.25 | 54048.55 | 54156.42 | 54163.36 | 54116.13 | 54062.13 | 54153 | 54104.46 | 54083.89 | 54149.33 |
| 679.0827 | 54108.9 | 54138.53 | 54020.55 | 54206.47 | 54094.13 | 54098.73 | 54110 | 54141.45 | 54066.47 | 54152.88 | 54205.7 | 54083.54 | 54104.78 | 54114.22 |
| 679.2147 | 54112.5 | 54039.65 | 54049.64 | 54147.47 | 54178.13 | 54172.09 | 54104.5 | 54145 | 54081.73 | 54183.88 | 54096.4 | 54183.62 | 54119.67 | 54132.67 |
| 679.3467 | 54113.1 | 54026.12 | 53936.18 | 54160.35 | 54072.63 | 54245.91 | 54113.5 | 54105.82 | 54017.93 | 54112.13 | 54100 | 54153.31 | 54096.89 | 54169.11 |
| 679.4787 | 54098.4 | 54043.41 | 53930.55 | 54125.71 | 54024.75 | 54299.45 | 54150.83 | 54171 | 54076.13 | 54107.13 | 54233.6 | 54061 | 54178.67 | 54192.33 |
| 679.6107 | 54165.3 | 54070.12 | 54018.45 | 54108.29 | 54085.63 | 54373.27 | 54160.17 | 54119.82 | 54065.87 | 54175.25 | 54160.7 | 54178.62 | 54288.22 | 54196.67 |
| 679.7427 | 54164.2 | 54072.88 | 53955.45 | 54154.12 | 54195.88 | 54172.73 | 54234.67 | 54080.18 | 54157.4 | 54111.13 | 54215.1 | 54263 | 54342.22 | 54114.89 |
| 679.8747 | 54123 | 54154.82 | 53959 | 54201.65 | 54215 | 54241.73 | 54293.08 | 54147.09 | 54142.8 | 54095 | 54192.8 | 54268.54 | 54255.56 | 54185.78 |
| 680.0067 | 54036.3 | 54130.71 | 54026.09 | 54218.29 | 54168.88 | 54239.91 | 54217.67 | 54209.91 | 54163.07 | 54059 | 54175.2 | 54193.08 | 54164.11 | 54223 |
| 680.1387 | 54049.9 | 54052.71 | 54096.45 | 54076.53 | 54095.75 | 54080.64 | 54194.42 | 54099.91 | 54187.2 | 54092.38 | 54152.6 | 54144.85 | 54111.33 | 54193.89 |
| 680.2707 | 54177.9 | 54008.59 | 54015.09 | 54206.06 | 54044.63 | 54097.64 | 54212.58 | 54169.45 | 54169.33 | 54132.88 | 54089.7 | 54228 | 54080.11 | 54120.89 |
| 680.4027 | 54230.4 | 53979.06 | 54020.91 | 54200.88 | 54022.13 | 54165.73 | 54224.92 | 54161.55 | 54128.87 | 54066.75 | 54194.5 | 54259.38 | 54141.67 | 54165.89 |
| 680.5347 | 54206.4 | 54043.82 | 53989.27 | 54186.18 | 54122.88 | 54142.82 | 54161.33 | 54125.82 | 54104.07 | 54166.38 | 54144.9 | 54156.77 | 54142.22 | 54156.67 |
| 680.6667 | 54180.5 | 54174.65 | 54050.91 | 54176.65 | 54345.63 | 54102.91 | 54183.33 | 54156.36 | 54144.47 | 54070.63 | 54079.9 | 54171.92 | 54101.89 | 54138.89 |
| 680.7987 | 54186.7 | 54184.53 | 54156.18 | 54192.88 | 54133.25 | 54094.45 | 54339.25 | 54161.82 | 54155.47 | 54064.63 | 54055.8 | 54143.54 | 54134.78 | 54117 |
| 680.9307 | 54175.9 | 54075.65 | 54111.73 | 54187.06 | 54050.38 | 54023.91 | 54262.83 | 54099.18 | 54080.6 | 54012.63 | 54059 | 54113.46 | 54035.89 | 54226.67 |
| 681.0627 | 54119.2 | 54120.94 | 54060.18 | 54298.41 | 54113.13 | 54044.36 | 54255 | 54192.36 | 53997.13 | 54025.5 | 54058.2 | 54103.77 | 54116.56 | 54242.56 |
| 681.1947 | 54195.8 | 54106.82 | 54045.91 | 54273 | 54153.63 | 54029.55 | 54223.67 | 54136.91 | 54020 | 54041.88 | 54069.3 | 54097.85 | 54128.56 | 54104.33 |
| 681.3267 | 54187.8 | 54075.47 | 54047.18 | 54196.47 | 54173.25 | 54114.82 | 54187.92 | 54093.64 | 54097.73 | 54066.75 | 54076.7 | 54131.08 | 54112.44 | 54123.11 |
| 681.4587 | 54135.1 | 54108.94 | 54077.82 | 54154.41 | 54089.38 | 54276.45 | 54133.17 | 54157.91 | 54095 | 54033.13 | 54134.1 | 54119.54 | 54030.56 | 54202.11 |
| 681.5907 | 54139.4 | 54092.24 | 54095.09 | 54223.06 | 54028 | 54173.73 | 54233.5 | 54105.45 | 54086 | 53998.75 | 54149.4 | 54181.62 | 53983.56 | 54316.78 |
| 681.7227 | 54070.4 | 54066.76 | 54090.55 | 54181.41 | 54071 | 54075.18 | 54234.83 | 54056.45 | 54048 | 54117.63 | 54144.3 | 54074.62 | 54019.33 | 54220.33 |
| 681.8547 | 54242.3 | 54105.65 | 54010.45 | 54152.18 | 54050.5 | 54085.55 | 54272.75 | 54024.55 | 54115.67 | 54126.75 | 54110.2 | 54188.23 | 54064.67 | 54119.22 |
| 681.9868 | 54254.6 | 54103.18 | 54106.82 | 54202.53 | 54058.5 | 54030.09 | 54181.58 | 54020.36 | 54119.4 | 54085.88 | 54052.4 | 54171.31 | 54079.89 | 54119.44 |
| 682.1187 | 54211.2 | 54159.29 | 53995.36 | 54170.94 | 54086.75 | 54107.36 | 54187.58 | 54028.09 | 54174.4 | 54184.5 | 54151.4 | 54180.31 | 54074 | 54148.89 |
| 682.2507 | 54140.6 | 54123.29 | 54087.27 | 54120.18 | 53985.38 | 54129.45 | 54215.75 | 53956.73 | 54121.67 | 54131.63 | 54130 | 54156 | 54067.33 | 54120.56 |
| 682.3828 | 54030.9 | 54133.76 | 54082.55 | 54119.41 | 54056.5 | 54091.82 | 54152.42 | 54043.36 | 54048.87 | 54235.5 | 54068.3 | 54186.31 | 54093.78 | 54062.33 |
| 682.5147 | 54128.5 | 54210.53 | 54011.73 | 54131.53 | 54108.63 | 54069.27 | 54248.5 | 54105.64 | 54064.93 | 54217.5 | 54212.7 | 54150.92 | 54202.78 | 54031.44 |
| 682.6467 | 54078 | 54071.41 | 53974.36 | 54171 | 54208.25 | 54072.09 | 54267.83 | 54085.64 | 54113.53 | 54139.25 | 54304 | 54126.23 | 54051.11 | 54067.89 |
| 682.7788 | 54172.8 | 54140.24 | 54028.09 | 54200.35 | 54205.13 | 54006.55 | 54187.25 | 54038.55 | 54191.87 | 54118.63 | 54141.3 | 54135.77 | 54090.22 | 54120.22 |
| 682.9108 | 54142 | 54117.88 | 54108.82 | 54165.53 | 54077.63 | 54036.91 | 54232.5 | 54088.73 | 54158.07 | 54143.13 | 54040 | 54096.85 | 54101 | 54168.67 |
| 683.0427 | 54130.2 | 54055.35 | 54034.64 | 54213.65 | 54042.75 | 54031.73 | 54178 | 54156.18 | 54119.4 | 54062.13 | 54086.7 | 54081 | 54102.44 | 54175.33 |
| 683.1747 | 54158 | 54109.88 | 54034.55 | 54220.88 | 54002.38 | 54175.45 | 54179.75 | 54140.09 | 54018.87 | 54055.75 | 54082.1 | 54140.92 | 54106.56 | 54143.67 |
| 683.3068 | 54036.5 | 54028.29 | 54074 | 54156.88 | 53953.5 | 54150.82 | 54131.67 | 54158.27 | 54110.27 | 54069.63 | 54059.4 | 54212.23 | 54123.33 | 54200.78 |
| 683.4388 | 54184.5 | 54062.29 | 54075.27 | 54223.06 | 54010.5 | 54125 | 54244 | 54155 | 54213.87 | 54123.88 | 54126.6 | 54142.85 | 54153.67 | 54189.22 |
| 683.5707 | 54334 | 54042.82 | 54038.64 | 54144.88 | 54066.5 | 54050.82 | 54247.92 | 54081.27 | 54153.67 | 54063 | 54139.1 | 54136.08 | 54095.78 | 54074.56 |
| 683.7028 | 54298.8 | 54011.35 | 53986.27 | 54171.29 | 54118 | 54038 | 54217.33 | 54118 | 54110.4 | 54093.88 | 54112 | 54194.23 | 54088.22 | 54019.33 |
| 683.8348 | 54149.1 | 54024.94 | 53953 | 54145.94 | 54060 | 54069.73 | 54312.83 | 54128.18 | 54069.73 | 54061.38 | 54160.1 | 54230.54 | 54156.11 | 54097.11 |
| 683.9667 | 54171.4 | 54065.18 | 53935.82 | 54219.88 | 54176.25 | 54088.55 | 54215.08 | 54163.18 | 54132.67 | 54106.88 | 54191.6 | 54094.15 | 54163 | 54040.22 |
| 684.0988 | 54107.2 | 54041.47 | 53991.45 | 54143 | 54081.5 | 54113.27 | 54151.25 | 54139.55 | 54151.33 | 54078.75 | 54160.5 | 54079.23 | 54208.56 | 54086.11 |
| 684.2308 | 54116.4 | 54090.47 | 54032.09 | 54205.12 | 54082.38 | 54191.36 | 54151.33 | 54179.55 | 54143.4 | 54070 | 54245.1 | 54098.92 | 54188.78 | 54068.67 |
| 684.3628 | 54093.9 | 54038.71 | 54041.18 | 54118.47 | 54093.13 | 54082.27 | 54202.67 | 54152.36 | 54127.93 | 54101.13 | 54127.7 | 54092.23 | 54111.11 | 54095.56 |
| 684.4948 | 54187.7 | 54126 | 54029.91 | 54212.82 | 54018.13 | 54013.09 | 54182.92 | 54158.45 | 54169.87 | 53963.75 | 54144.6 | 54100.31 | 54095.67 | 54153.67 |
| 684.6268 | 54134.2 | 54079.53 | 53990 | 54186 | 54021.13 | 54140.64 | 54186.17 | 54179.64 | 54313.2 | 54014.75 | 54147.9 | 54076.54 | 54144.67 | 54158 |
| 684.7588 | 54161.5 | 54144.29 | 53936.55 | 54177.41 | 54104.75 | 54145.36 | 54131.5 | 54283.45 | 54226.47 | 53982.38 | 54078.3 | 54188.77 | 54098.89 | 54119.22 |
| 684.8908 | 54290 | 54169 | 54019.18 | 54210.47 | 54217.25 | 54059.73 | 54110.42 | 54196.27 | 54140.93 | 54224 | 54079.3 | 54120.23 | 54138.67 | 54163.78 |
| 685.0228 | 54074.1 | 54123.53 | 54086 | 54158.06 | 54224.13 | 54061.91 | 54175.17 | 54200.64 | 54175.53 | 54289.38 | 54063.7 | 54083.38 | 54124.11 | 54103.78 |
| 685.1548 | 54068.2 | 54022.18 | 54129.73 | 54078.82 | 54024.75 | 54231 | 54239.17 | 54256.64 | 54169.2 | 54254 | 54031.5 | 54100 | 54125.78 | 54137.89 |
| 685.2868 | 54136.1 | 54035.76 | 53968.36 | 54130.88 | 54066.5 | 54157.09 | 54175.08 | 54236.73 | 54124.87 | 54302.13 | 54071.2 | 54119.92 | 54108.11 | 54116.78 |
| 685.4188 | 54143.2 | 54080.53 | 54107.09 | 54251.24 | 54186.5 | 54193.82 | 54250.17 | 54234.45 | 54167.27 | 54221.63 | 54186.5 | 54114.77 | 54162.67 | 54192.67 |
| 685.5508 | 54101.2 | 54122.06 | 53994.36 | 54297.71 | 54172 | 54120.45 | 54153.92 | 54180.55 | 54143.53 | 54162.38 | 54091.1 | 54193.69 | 54148.44 | 54099.11 |
| 685.6828 | 54080.8 | 54086.47 | 54044.27 | 54229.41 | 54108 | 54131.91 | 54115.58 | 54144.91 | 54067.27 | 54206.63 | 54044.1 | 54221.85 | 54184 | 54101.78 |
| 685.8148 | 54192.1 | 54084.41 | 54013.27 | 54279.12 | 54131.88 | 54112.45 | 54085.42 | 54149.91 | 54091.2 | 54043.88 | 54108.5 | 54139 | 54108.33 | 54076.11 |
| 685.9468 | 54246.7 | 54040.06 | 54065.64 | 54271.88 | 54102.63 | 54062.18 | 54125.25 | 54221.82 | 54130.07 | 54089.88 | 54105.6 | 54090.69 | 54118.56 | 54236 |
| 686.0788 | 54138.4 | 54057 | 54018.64 | 54240.18 | 54097.88 | 54037.45 | 54124.67 | 54143.64 | 54090.07 | 54091 | 54062 | 54162.54 | 54117.22 | 54152.56 |
| 686.2108 | 54123.4 | 54102.65 | 54008.27 | 54177.53 | 54227.75 | 54043.36 | 54159.5 | 54250.55 | 54049 | 54053 | 54120.8 | 54171.15 | 54124.56 | 54082 |
| 686.3428 | 54162.7 | 54079.35 | 54107.18 | 54123.59 | 54181.25 | 54110.27 | 54037.08 | 54152.27 | 54010.93 | 53942 | 54217.4 | 54126 | 54211.44 | 54126.11 |
| 686.4748 | 54141 | 54111.47 | 54048.45 | 54182.47 | 54052.88 | 54132.27 | 54126.5 | 54028.64 | 54124.2 | 54007.5 | 54164.6 | 54195 | 54081.11 | 54087.78 |
| 686.6068 | 54121.7 | 54146.41 | 54025.91 | 54216.94 | 54122.63 | 54195.73 | 54065.75 | 54086.09 | 54154.4 | 54126.25 | 54118.8 | 54224.77 | 54084.56 | 54108.33 |
| 686.7388 | 54144.4 | 54103.94 | 53950.45 | 54248.12 | 54153.75 | 54135.36 | 54097.25 | 54114.64 | 54212.27 | 54126.38 | 54086.7 | 54045.77 | 54129.44 | 54185.33 |
| 686.8708 | 54286.6 | 54159.12 | 54075.91 | 54141.65 | 54152.5 | 54186.55 | 54127.25 | 54085.09 | 54173.8 | 54177.88 | 54168.5 | 54117.85 | 54140.22 | 54173.67 |
| 687.0028 | 54248.9 | 54132.29 | 54140.27 | 54165.29 | 54154.13 | 54167.36 | 54158.17 | 54028.18 | 54126.47 | 54144 | 54095.7 | 54126.69 | 54127.11 | 54124.56 |
| 687.1348 | 54207 | 54098.71 | 54071.09 | 54143.41 | 54136.88 | 54110 | 54140.92 | 54046 | 54081.27 | 54198.13 | 54187.5 | 54045.92 | 54071.56 | 54176.67 |
| 687.2668 | 54282.8 | 54106.12 | 54138.18 | 54149.71 | 54068.13 | 54190.55 | 54180.42 | 54127.09 | 54206.6 | 54059.5 | 54168.5 | 54073.23 | 54080.11 | 54241.78 |
| 687.3988 | 54296.4 | 54092.71 | 54110.09 | 54173.18 | 54017.75 | 54310 | 54288.08 | 54082.45 | 54169.13 | 54068.88 | 54095.5 | 54110.15 | 54147.89 | 54185.44 |
| 687.5308 | 54138.1 | 54100.71 | 54161.64 | 54141.24 | 54086 | 54209.55 | 54129.33 | 54076.73 | 54125.27 | 54131.25 | 54155.4 | 54008.54 | 54085.22 | 54170.11 |
| 687.6628 | 54087.4 | 54051.82 | 54149 | 54145.47 | 54214 | 54083.27 | 54101.75 | 54140.27 | 54116.13 | 54225.63 | 54174.7 | 53972.62 | 54089.56 | 54108.56 |
| 687.7948 | 54102.9 | 54155.29 | 54040.18 | 54236.65 | 54165.25 | 54143.73 | 54156.08 | 54141.45 | 54121.4 | 54143.38 | 54084.6 | 54046.92 | 54141.56 | 54100.67 |
| 687.9268 | 54204.2 | 54116.18 | 54056.18 | 54204 | 54025.63 | 54162.55 | 54156.33 | 54094.27 | 54181 | 54157.13 | 53998.1 | 54036.85 | 54167.11 | 54129.33 |
| 688.0588 | 54271.5 | 54045 | 54100.64 | 54342.53 | 54000.63 | 54125.09 | 54187.08 | 54092.18 | 54115.87 | 54037 | 54065.5 | 54074.77 | 54199.44 | 54009.22 |
| 688.1908 | 54309 | 54055.06 | 54037 | 54262.94 | 54053.88 | 54090.36 | 54099.33 | 54038.27 | 54141.13 | 54016.63 | 54101.2 | 54149.23 | 54152.89 | 54111.67 |
| 688.3228 | 54289 | 54056.47 | 54021.55 | 54171.59 | 54064.88 | 54101.55 | 54058.33 | 54055.82 | 54089.07 | 54065.25 | 54124.1 | 54151.15 | 54150.67 | 54091.78 |
| 688.4548 | 54122 | 54083.71 | 54011.55 | 54095.59 | 53989.25 | 54241.82 | 54064.92 | 54088.09 | 54074.27 | 54031.75 | 54119.5 | 54066.92 | 54146 | 54182.56 |
| 688.5869 | 54076.5 | 54018.76 | 54033.64 | 54182.18 | 54039.75 | 54185.45 | 54027.25 | 54119.45 | 54129.33 | 54143.13 | 54211.2 | 54054.23 | 54235.78 | 54076.11 |
| 688.7188 | 54083.3 | 54056.12 | 54020.55 | 54178.88 | 54097.5 | 54112.55 | 54169.92 | 54035.64 | 54083.87 | 54110.88 | 54154.2 | 54078.15 | 54206.44 | 54057.33 |
| 688.8508 | 54135.5 | 54069.12 | 54020.27 | 54268.18 | 54194.63 | 54037.64 | 54250.58 | 54088 | 54103.6 | 54012.38 | 54105.9 | 54133.85 | 54188.67 | 54173.11 |
| 688.9829 | 54142.7 | 54129.29 | 54138.91 | 54163.53 | 54043.75 | 54099.55 | 54051.5 | 54072.27 | 54249 | 54176.13 | 54080.7 | 54045.62 | 54280.78 | 54228.33 |
| 689.1148 | 54156.9 | 54159.88 | 54125.09 | 54150.29 | 54096.88 | 54157.91 | 54141.17 | 54096.55 | 54173.67 | 54168.13 | 54185.1 | 54111.38 | 54162.22 | 54180.33 |
| 689.2468 | 54084.3 | 54186.41 | 54098.09 | 54130.94 | 54208.5 | 54170.91 | 54144.58 | 54197.09 | 54095 | 54073.38 | 54143.9 | 54189.23 | 54165.22 | 54068.11 |
| 689.3789 | 54136.4 | 54150.76 | 54067.91 | 54117.47 | 54159.75 | 54095.09 | 54172.75 | 54117 | 54097.2 | 54133.63 | 54160.6 | 54063.62 | 54265.22 | 54079.89 |
| 689.5109 | 54225.4 | 54085.29 | 54035.18 | 54147.41 | 54319.5 | 54198.73 | 54333.5 | 54051.18 | 54044.07 | 54183.13 | 54232.7 | 54083.15 | 54229.67 | 54100.11 |
| 689.6428 | 54108.2 | 54139.41 | 54118 | 54145.06 | 54100 | 54070.73 | 54124.5 | 54048.27 | 54057.73 | 54139.88 | 54099.4 | 54120 | 54190.56 | 54057.44 |
| 689.7748 | 54136.9 | 54089.82 | 54046.45 | 54188.47 | 53976.13 | 54140.64 | 54353.17 | 54066.55 | 54096.67 | 54057.88 | 54110.3 | 54120.23 | 54050.67 | 54080.11 |
| 689.9069 | 54136 | 54108.76 | 54186.64 | 54119.47 | 53998.63 | 54077.91 | 54128.75 | 54117.45 | 54084.07 | 54080.13 | 54142.8 | 54158.92 | 54035.89 | 53975.33 |
| 690.0389 | 54185.5 | 54133.82 | 54175.91 | 54189.47 | 54108.63 | 54075.73 | 54155.92 | 54020.27 | 54054.47 | 54109.5 | 54305.6 | 54117.69 | 54088.33 | 54045.33 |
| 690.1708 | 54185.1 | 54038.24 | 54127.55 | 54137.12 | 54097.25 | 54152.27 | 54180.75 | 53989.82 | 54100.67 | 54274.75 | 54221.1 | 54238.62 | 54147.67 | 54105.56 |
| 690.3029 | 54108.2 | 54092.59 | 54046.09 | 54116.82 | 54065.88 | 54130.55 | 54276.17 | 54111 | 54087.8 | 54130.38 | 54113.4 | 54114 | 54112.22 | 54040.33 |
| 690.4349 | 54066.4 | 54127.12 | 54026.45 | 54133.59 | 54247 | 54151.73 | 54110.42 | 54068 | 54120 | 54088.75 | 54134.3 | 54094.23 | 54012.33 | 54033.78 |
| 690.5668 | 54087.1 | 54074.59 | 53992.64 | 54142.18 | 54169.5 | 54060.73 | 54056.08 | 54038.27 | 54113.13 | 54036.63 | 54095.6 | 54023.46 | 54129.44 | 54070.11 |
| 690.6989 | 54127 | 54049.71 | 54107.82 | 54159.76 | 54009.75 | 54099.18 | 54096.67 | 54185.45 | 54123.73 | 54008 | 54306.2 | 54091.15 | 54070.22 | 54054.56 |
| 690.8309 | 54129.2 | 54083.59 | 54151.18 | 54094 | 54003.38 | 54125.18 | 54089.17 | 54113.64 | 54149.47 | 54164.88 | 54221.2 | 54139.54 | 54123 | 53994.44 |
| 690.9629 | 54119.6 | 54085.47 | 54129.91 | 54122.18 | 54116.5 | 54075.73 | 54060.83 | 54063.36 | 54171.47 | 54191.38 | 54098.5 | 54121.23 | 54092 | 54092 |
| 691.0949 | 54134.4 | 54014.94 | 54062.45 | 54080.41 | 54243.38 | 54113.73 | 54052.75 | 54098.82 | 54109.47 | 54161.25 | 54128.6 | 54064.85 | 53960.67 | 54085 |
| 691.2269 | 54168.8 | 54065.76 | 54013.45 | 54139.76 | 54179 | 54040.45 | 54066.08 | 54121.27 | 54138.6 | 54155.25 | 54141.9 | 54110.69 | 54027.89 | 54142.78 |
| 691.3589 | 54099.3 | 54118.47 | 54129.09 | 54170 | 54005.25 | 54170.55 | 54180.08 | 54208.82 | 54159.47 | 54033.38 | 54189.1 | 54107.77 | 54118.33 | 54178.11 |
| 691.4908 | 54066.1 | 54068.59 | 54025.91 | 54270.71 | 54146.63 | 54127.09 | 54163.92 | 54273.18 | 54100.47 | 54118.75 | 54107.5 | 54103.38 | 54050.22 | 54137.78 |
| 691.6229 | 54086 | 54087.71 | 54125.36 | 54292.65 | 54110.13 | 54064.82 | 54127.67 | 54216.82 | 54064.73 | 54120.13 | 54108 | 54138 | 54152.67 | 54150.33 |
| 691.7549 | 54033.9 | 54105.12 | 54175.27 | 54172.41 | 54225.88 | 54115.45 | 54076.92 | 54210 | 54149.8 | 54134.5 | 54126.3 | 54198.62 | 54030.33 | 54161.44 |
| 691.8869 | 54109.5 | 54015.71 | 54050.55 | 54120.71 | 54213.25 | 54136.91 | 54176.92 | 54177.64 | 54290.67 | 54057.25 | 54125.2 | 54149.23 | 54182.11 | 54102.22 |
| 692.0189 | 54041.9 | 53999.41 | 54016.36 | 54137.47 | 54146.5 | 54088.64 | 54144.58 | 54206.27 | 54165.6 | 54095.88 | 54106.6 | 54109.46 | 54138.33 | 54073.89 |
| 692.1509 | 54004.4 | 54092.71 | 53998.18 | 54201.53 | 54227.13 | 54137.55 | 54148.58 | 54164 | 54164.2 | 54244.38 | 54061.4 | 54086.77 | 54118.78 | 54125.44 |
| 692.2829 | 54085.4 | 54087.35 | 53991.64 | 54185.06 | 54122.88 | 54123.55 | 54116.67 | 54131.55 | 54084.93 | 54139.63 | 54096.9 | 54197.38 | 54034.22 | 54165.44 |
| 692.4149 | 54212.3 | 54060.94 | 54071.09 | 54192 | 54144.5 | 54196.55 | 54161.58 | 54165.64 | 54182.2 | 54246 | 54117.1 | 54172.92 | 54082.67 | 54170.44 |
| 692.5469 | 54247.3 | 54028.12 | 54176.64 | 54210.59 | 54109.88 | 54211.45 | 54117 | 54065.45 | 54193.6 | 54160 | 54106.8 | 54067.08 | 54077.89 | 54155.67 |
| 692.6789 | 54083.6 | 54044 | 54180.91 | 54287.47 | 54172.5 | 54186.91 | 54191.75 | 54013.55 | 54232.73 | 54054.25 | 54109.7 | 54190.77 | 54196.44 | 54090.11 |
| 692.8109 | 54174.8 | 54037.12 | 54051.82 | 54207.41 | 54056.25 | 54201.73 | 54116 | 54048.09 | 54041.8 | 54132.75 | 54288.6 | 54103.31 | 54198.56 | 54144.78 |
| 692.9429 | 54120.9 | 54071.47 | 54017.18 | 54298.18 | 54047.88 | 54085.45 | 54158.5 | 54121 | 54066.8 | 54233.88 | 54224.5 | 54112.46 | 54123.11 | 54084.11 |
| 693.0749 | 54173.7 | 54094.82 | 54002.64 | 54287.29 | 54092.63 | 54047.82 | 54174 | 54214.45 | 54125.13 | 54142.88 | 54213.8 | 54109.15 | 54143.44 | 54131.67 |
| 693.2069 | 54159.9 | 54047.06 | 54082.09 | 54211.94 | 54147.88 | 54172.27 | 54113.25 | 54284.55 | 54202.8 | 54137.25 | 54163.4 | 54132.54 | 54328.78 | 54140.89 |
| 693.3389 | 54249.4 | 54074.29 | 53989.55 | 54252.06 | 54215.13 | 54116.82 | 54142.58 | 54111.82 | 54129.13 | 54145.13 | 54149.9 | 54172.46 | 54228.89 | 54100.33 |
| 693.4709 | 54252.4 | 54081.53 | 53984.55 | 54151.18 | 54231.38 | 54112.55 | 54138.58 | 54099.45 | 54178.93 | 54060.5 | 54157.4 | 54184.46 | 54089 | 54159.11 |
| 693.6029 | 54218.4 | 54058.29 | 54062.27 | 54233.12 | 54099.5 | 54026.27 | 54096 | 54008.91 | 54092.07 | 54146.25 | 54083.3 | 54217.77 | 54154.67 | 54212 |
| 693.7349 | 54178.2 | 54091 | 54135.45 | 54158.06 | 54134 | 54102.82 | 54094.58 | 54016.18 | 54088.6 | 54234.5 | 54186.8 | 54176.15 | 54089.11 | 54165.44 |
| 693.8669 | 54161.3 | 54046.47 | 54061.91 | 54133.88 | 54027.38 | 54100.27 | 54105.08 | 54089.18 | 54111.67 | 54115.25 | 54299.8 | 54212.77 | 54142.33 | 54140.89 |
| 693.9989 | 54188.2 | 54075.65 | 54032 | 54182.12 | 54033.5 | 54110.09 | 54106.58 | 54115.36 | 54116.67 | 54124 | 54247.2 | 54135.69 | 54084.78 | 54182.11 |
| 694.1309 | 54097.5 | 54060.76 | 54097.91 | 54245.18 | 54058.13 | 54049.55 | 54117.33 | 54164.18 | 54136.53 | 54204.88 | 54099.2 | 54096.62 | 54026.44 | 54154.44 |
| 694.2629 | 54083.2 | 54199.76 | 54123.55 | 54205.06 | 54134 | 54102.55 | 54111.75 | 54145.27 | 54099.27 | 54163.5 | 54177.9 | 54148 | 54066.89 | 54205 |
| 694.3949 | 54187.6 | 54123.65 | 54073.55 | 54198.76 | 54054.25 | 54230.27 | 54281.33 | 54149.45 | 54082.33 | 54182.63 | 54227 | 54200.38 | 54148.89 | 54176.22 |
| 694.5269 | 54161.7 | 54111 | 54050.73 | 54199.94 | 54021.5 | 54143.91 | 54116.75 | 54085.82 | 54075.87 | 54243.63 | 54121.8 | 54226.08 | 54032.44 | 54212.44 |
| 694.6589 | 54160 | 54105.71 | 54037.27 | 54170.71 | 54063.13 | 54165.36 | 54070.58 | 54067.64 | 54098.47 | 54406.25 | 54050.5 | 54242.92 | 54015 | 54201.22 |
| 694.7909 | 54094 | 54097.18 | 54071.36 | 54210 | 54064.25 | 54153.73 | 54019.08 | 54014.45 | 54035.8 | 54231.88 | 54086.5 | 54161.38 | 54090.11 | 54191.78 |
| 694.9229 | 54179.2 | 54086.12 | 54065.36 | 54204.71 | 54068.88 | 54138.91 | 54086.25 | 54053.73 | 54043.4 | 54100.13 | 54104.3 | 54046 | 54107.56 | 54094.56 |
| 695.0549 | 54075.2 | 54055.59 | 53993.91 | 54143.76 | 53954.88 | 54100 | 54115.42 | 54165.73 | 54116.93 | 54038.63 | 54076.6 | 54088.54 | 54042.11 | 54069.67 |
| 695.187 | 54125.7 | 54030.24 | 54095.18 | 54191.76 | 53982.38 | 54186.27 | 54116.92 | 54029.36 | 54069.8 | 53959.63 | 54019.8 | 54077.31 | 54036 | 54324.78 |
| 695.3189 | 54234.3 | 54058.41 | 54039.82 | 54118.24 | 54118.5 | 54159 | 54122.92 | 54070.73 | 54076 | 54026.88 | 54138.6 | 54076.92 | 54095.56 | 54258.67 |
| 695.4509 | 54122.5 | 54087.35 | 54176.91 | 54121.24 | 54154.38 | 54265.09 | 54067.08 | 54025.55 | 54097.8 | 54024.75 | 54249.5 | 54125.15 | 54073.33 | 54285.11 |
| 695.583 | 54193.5 | 54009.94 | 54103.45 | 54211 | 53994.13 | 54219.55 | 54083.08 | 54068.36 | 54094.53 | 54083.88 | 54249.5 | 54157 | 54069.11 | 54221.11 |
| 695.7149 | 54064.2 | 54064.24 | 54013.64 | 54184.18 | 54124.88 | 54028.55 | 54136.33 | 54076.18 | 54096.33 | 54294.38 | 54054.2 | 54095.54 | 54114.78 | 54169.56 |
| 695.8469 | 54190.6 | 54066.12 | 54183.36 | 54158.82 | 54049.13 | 54143.91 | 54120.58 | 54084.36 | 54063.07 | 54052.5 | 54109.6 | 54110.38 | 54022.44 | 54165.44 |
| 695.9789 | 54117.7 | 54118.29 | 54158.36 | 54207.47 | 54107.38 | 54144.45 | 54102 | 54032.27 | 54037 | 54047.25 | 54093.4 | 54110.15 | 54121.22 | 54086.11 |
| 696.111 | 54136.1 | 54095.53 | 54170.82 | 54242.88 | 54065.5 | 54159.64 | 54139.25 | 54093.82 | 54077.13 | 54175 | 54176.8 | 54017.54 | 54022.33 | 54043.44 |
| 696.2429 | 54158.4 | 54102.35 | 53992.73 | 54260.53 | 54047.13 | 54130.55 | 54182.5 | 54114.82 | 54045.13 | 54187.13 | 54162.5 | 54045.69 | 54101.89 | 54064.89 |
| 696.3749 | 54068.1 | 54121.24 | 53998.91 | 54187.94 | 54049.63 | 54056.36 | 54108.92 | 54130.36 | 53968.53 | 54087.5 | 54171.3 | 53999.46 | 54100.67 | 54047.11 |
| 696.507 | 54118.1 | 54009.59 | 54120.55 | 54203.82 | 54080.63 | 54072.27 | 54249.92 | 54277.55 | 54083.47 | 54086.75 | 54282.8 | 54038.62 | 54059.44 | 54017.56 |
| 696.639 | 54182.9 | 54082.06 | 54076.73 | 54195.41 | 54198.63 | 54094 | 54204.33 | 54109.73 | 54061.13 | 54013.38 | 54206.2 | 54166.15 | 54157.56 | 54160.44 |
| 696.7709 | 54156 | 54107.59 | 54059 | 54159.71 | 54097 | 54177.36 | 54219.67 | 54197.82 | 54026.2 | 54146.88 | 54244.4 | 54232.69 | 54138.67 | 54025.56 |
| 696.903 | 54102.5 | 54079.24 | 54103.27 | 54216.47 | 54149.88 | 54138.45 | 54160.33 | 54231.09 | 54150.33 | 54106.88 | 54063.7 | 54256 | 54149.33 | 54102 |
| 697.035 | 54127.8 | 54073.18 | 54132.82 | 54215 | 54072.13 | 54029.18 | 54299.75 | 54207.09 | 54118.6 | 54120.75 | 53996.1 | 54223.23 | 54066.33 | 54140.33 |
| 697.1669 | 53969.2 | 54073.06 | 54154.82 | 54287.06 | 54144.5 | 54053.36 | 54197.08 | 54022.09 | 54135.6 | 54095 | 54034.3 | 54239 | 54064.22 | 54220.78 |
| 697.299 | 53996.1 | 54086.18 | 54051.45 | 54253.94 | 54077.63 | 54071.45 | 54153.92 | 54152 | 54044.27 | 54096.75 | 54149.3 | 54168.92 | 54031 | 54130.22 |
| 697.431 | 54144.3 | 54008.94 | 53996.18 | 54096.82 | 53956 | 53996.18 | 54099.92 | 54081.36 | 54065.07 | 54083.88 | 54119.5 | 54155.62 | 54008.89 | 54130.67 |
| 697.563 | 54120.1 | 54034.65 | 54021.64 | 54094 | 53955 | 54112.55 | 54028.17 | 54071.91 | 54134.47 | 54280.25 | 54125.1 | 54103.69 | 54034.78 | 54138.33 |
| 697.695 | 54087.1 | 54065 | 53995.09 | 54190.18 | 53973 | 54165.27 | 54062.42 | 54134.45 | 54080.13 | 54180.63 | 54119.7 | 54141.77 | 54243.44 | 54116.89 |
| 697.827 | 54158.6 | 54101.71 | 53987.82 | 54133.88 | 54019.63 | 54230.45 | 54182.58 | 54091.82 | 54106 | 54183.38 | 53994.7 | 54099.15 | 54164.56 | 54105.33 |
| 697.959 | 54118.9 | 54297.12 | 54006.55 | 54156.71 | 54119.38 | 54101.64 | 54137.67 | 54108.73 | 54071.27 | 54050.88 | 54139.2 | 54115.69 | 54075.56 | 54142.89 |
| 698.0909 | 54064.2 | 54166.94 | 54094.27 | 54157.53 | 54152.25 | 54207.18 | 54084.08 | 54017 | 54166 | 54070.5 | 54090.9 | 54104.77 | 54096.33 | 54147 |
| 698.223 | 54143.5 | 54113.71 | 54070.09 | 54132.65 | 54083 | 54084.18 | 54107.75 | 54001 | 54157.47 | 54054.75 | 54129.4 | 54048.23 | 54178.89 | 54211.89 |
| 698.355 | 54202.4 | 54140.88 | 54033.55 | 54087.94 | 54248.88 | 54073.82 | 54170 | 54036 | 54089.13 | 53961.5 | 54138.9 | 54167 | 54182.67 | 54120.78 |
| 698.487 | 54079.8 | 54187 | 54113.36 | 54135 | 54149.25 | 54110.91 | 54141.33 | 54087 | 54044.73 | 54072.38 | 54147.5 | 54067.92 | 54219.33 | 54035.11 |
| 698.619 | 54092.9 | 54114.94 | 54080 | 54135.12 | 54158.38 | 54172.09 | 54189.25 | 54099.45 | 53991.8 | 54096.38 | 54115.4 | 54052.62 | 54122.67 | 54068.11 |
| 698.751 | 54111.7 | 54120.82 | 54048.73 | 54123.82 | 54133.13 | 54163.82 | 54082.92 | 54091.45 | 54018.27 | 54086.25 | 54078.2 | 54185 | 54127.11 | 54158.67 |
| 698.883 | 54077.6 | 54018.94 | 54031.45 | 54176.47 | 54081.75 | 54140.55 | 54146.92 | 54026.36 | 53994.07 | 54184.5 | 54063.2 | 54171.92 | 54162.78 | 54148.67 |
| 699.015 | 54147.8 | 54066.41 | 54137.91 | 54195.94 | 54006 | 54138.73 | 54208.75 | 54019.36 | 54078.53 | 53948.25 | 54091.2 | 54256.38 | 54208.78 | 54164.22 |
| 699.147 | 54192.6 | 54208 | 54078.27 | 54222.29 | 54022.25 | 54093.55 | 54244.67 | 54136.18 | 54158.27 | 54118.38 | 54054.5 | 54246.31 | 54028.22 | 54173.11 |
| 699.279 | 54134 | 54109.53 | 54057.27 | 54219.65 | 53996.75 | 54083.09 | 54116.08 | 54191.73 | 54078.2 | 54083.25 | 54060.3 | 54259.54 | 54088.11 | 54048.78 |
| 699.411 | 54085.6 | 54102.82 | 53946.64 | 54275.29 | 54144.63 | 54056.09 | 54131.75 | 54081.18 | 54052.53 | 54202.5 | 54148.2 | 54367.69 | 54190.33 | 54107.56 |
| 699.543 | 54092.1 | 54088.06 | 54000.09 | 54137.53 | 54139.75 | 54078.55 | 54232.92 | 54094.36 | 54041 | 54015.63 | 54098.1 | 54173.31 | 54058.44 | 54003.78 |
| 699.675 | 54042.3 | 54045.94 | 54035.36 | 54082.35 | 54146.38 | 54028.09 | 54119.17 | 54040.18 | 54060.93 | 54153.25 | 54082 | 54149.15 | 54060.67 | 54212.89 |
| 699.807 | 54042.7 | 54082.12 | 54107.36 | 54115.53 | 54117.25 | 54115.45 | 54100.5 | 54107.45 | 54120.93 | 54201.13 | 54076.3 | 54085.46 | 54072.67 | 54101.78 |
| 699.939 | 54066.1 | 54106.65 | 54041.36 | 54142.65 | 54056 | 54207.18 | 54049.25 | 54031.82 | 54087.6 | 54056 | 54144.7 | 54133.38 | 54157.56 | 54105.44 |
| 700.071 | 54074.7 | 54141.65 | 54155.36 | 54149.41 | 53963.5 | 54169.36 | 54047.83 | 54122.45 | 54115.73 | 54286.38 | 54098 | 54103.85 | 54109.11 | 54184.89 |
| 700.203 | 54036.8 | 54184.88 | 54107.18 | 54267.18 | 54026 | 54160.64 | 54062.67 | 54087.73 | 54077.6 | 54119.25 | 54036.4 | 54075.23 | 54081.44 | 54090.22 |
| 700.335 | 54075.5 | 54228.35 | 54092.27 | 54225.65 | 54074.88 | 54182.82 | 54125.33 | 54135.36 | 54108.53 | 54412.13 | 54117.5 | 54060.23 | 54184.56 | 54220.89 |
| 700.467 | 54183.2 | 54114.88 | 54096.36 | 54091.12 | 54095.5 | 54121.45 | 54206 | 54134.36 | 54069.4 | 54299.25 | 54086.9 | 54050.54 | 54308.22 | 54061.33 |
| 700.599 | 54128.3 | 54086.41 | 54069.73 | 54094.94 | 54143.25 | 54060.64 | 54205.58 | 54111.64 | 54082.67 | 54176.63 | 54198.8 | 54053.08 | 54159.44 | 54073 |
| 700.731 | 54225.1 | 54051.06 | 54109 | 54181.65 | 54038.38 | 54009.55 | 54136 | 54047.55 | 54080.67 | 54165.5 | 54210.3 | 54091.92 | 54065.44 | 54034.56 |
| 700.863 | 54191.4 | 54105.76 | 54103.18 | 54205.82 | 54021.38 | 54095.27 | 54041 | 54101.36 | 54103.53 | 54153.75 | 54287.9 | 54191.38 | 54081 | 54026.44 |
| 700.995 | 54187.8 | 54125.29 | 54006.36 | 54185.94 | 54145.25 | 54102 | 54132.42 | 54097.82 | 54076.2 | 54131 | 54102.7 | 54142.62 | 54117.89 | 54083 |
| 701.127 | 54153.5 | 54120.94 | 54045.18 | 54139.06 | 54100.5 | 54093.27 | 54136.5 | 54132.64 | 54051.93 | 54078.5 | 54032.6 | 54268.23 | 54083.22 | 54102.44 |
| 701.259 | 54190.8 | 54238.65 | 54083.64 | 54178.82 | 54021.13 | 54086.36 | 54147 | 54106.36 | 54147.67 | 54175.88 | 54063.5 | 54227.54 | 54123.67 | 54133.78 |
| 701.391 | 54102.2 | 54132.47 | 54056.91 | 54171.53 | 53975.75 | 54176.45 | 54113.5 | 54112 | 54073.73 | 54192.25 | 54156.9 | 54127.69 | 54079.78 | 54102.78 |
| 701.523 | 54124.2 | 54160.29 | 54024 | 54115.29 | 53979.63 | 54227.91 | 54160.92 | 54118.09 | 54090.67 | 54018.38 | 54309.8 | 54238 | 54052.33 | 54165 |
| 701.655 | 54195.7 | 54070.41 | 54077.64 | 54190 | 54107.13 | 54121.55 | 54167.08 | 54212.55 | 54105.2 | 54043 | 54207.9 | 54062.77 | 54048.78 | 54196.11 |
| 701.7871 | 54201.6 | 54053.12 | 54017.09 | 54089.24 | 54062.75 | 54107.36 | 54163.5 | 54161 | 54105.67 | 54051.63 | 54072.8 | 54126 | 54141 | 54174.44 |
| 701.919 | 54146.8 | 54006.35 | 54011.55 | 54077.29 | 54047.63 | 54084.36 | 54191.08 | 54135.36 | 54185.73 | 54152.25 | 54128 | 54159.92 | 54163.11 | 54220.22 |
| 702.051 | 54140.6 | 54068.06 | 54053.82 | 54158.12 | 54090.5 | 54182.27 | 54203.83 | 54174.45 | 54070.27 | 54191.75 | 54129.3 | 54080.54 | 54110 | 54248.89 |
| 702.183 | 54150.2 | 54036.29 | 53973.09 | 54077.29 | 53996.5 | 54096 | 54229.5 | 54143.73 | 54049 | 54136.63 | 54332.4 | 54140.15 | 54199 | 54229.44 |
| 702.315 | 54131.3 | 54077.24 | 54015.82 | 54109.53 | 54035.88 | 54089.27 | 54163.17 | 54110.64 | 54117.27 | 54133 | 54233.3 | 54082.77 | 54119.11 | 54159.11 |
| 702.447 | 54138 | 54106.24 | 54023 | 54128.41 | 54147 | 54098.91 | 54052.17 | 54140.36 | 54164.67 | 54177.5 | 54157.2 | 53987.92 | 54182.78 | 54107.22 |
| 702.579 | 54049.6 | 54147.71 | 54016.45 | 54178.41 | 54165.63 | 53985.27 | 54123 | 54132.73 | 54141.27 | 54030.88 | 54138.8 | 54060.46 | 54205.89 | 54119.22 |
| 702.7111 | 54002.2 | 54074.35 | 54009.55 | 54188.82 | 54029.63 | 54026.55 | 54205.75 | 54116.55 | 54086.27 | 54071.13 | 54035.2 | 54093 | 54086.56 | 54093.33 |
| 702.843 | 53983.7 | 54121.41 | 54078.36 | 54269.47 | 54030.63 | 54099.45 | 54217.83 | 54055.09 | 54046.07 | 54154.88 | 54012.9 | 54157 | 54130.44 | 53997.44 |
| 702.975 | 54001.4 | 54052.24 | 54112.18 | 54196.29 | 53986.38 | 54130.55 | 54391.92 | 54040.45 | 54106.73 | 54099.63 | 54021.4 | 54189.92 | 54032.56 | 54047.89 |
| 703.1071 | 54049.1 | 54044.35 | 54015.09 | 54161.12 | 54070 | 54178.73 | 54258.42 | 54139.64 | 54134.4 | 54109.13 | 54030.9 | 54213.31 | 54049.67 | 54127.44 |
| 703.2391 | 54148.1 | 53976.12 | 54058.27 | 54101.94 | 54045 | 54213.36 | 54167.92 | 54193.82 | 54132.2 | 54115 | 54092.4 | 54153.92 | 54119.78 | 54024.67 |
| 703.371 | 54089.7 | 54050.41 | 54087.36 | 54118.29 | 54133 | 54104 | 54033.75 | 54174.45 | 54191.73 | 54152.25 | 54147.8 | 54042.23 | 54046.11 | 54038.89 |
| 703.5031 | 54151 | 54054.06 | 54032.36 | 54104.76 | 54279.63 | 54029 | 54079.42 | 54126 | 54147.93 | 54151.25 | 54146.3 | 54117.08 | 54159.22 | 54132.22 |
| 703.6351 | 54090.1 | 53973.06 | 54064.55 | 54116.59 | 54075.25 | 54050.09 | 54164.42 | 54172.45 | 54050.13 | 54151.63 | 54136.4 | 53992.85 | 54232.22 | 54038.67 |
| 703.767 | 54111.7 | 53982.94 | 54079 | 54078 | 54141.75 | 54097 | 54141.75 | 54087.73 | 54192.73 | 54093.25 | 54137.8 | 54135.92 | 54077.22 | 54112.89 |
| 703.8991 | 54047.8 | 54023.06 | 54064.36 | 54087.18 | 54128.38 | 54148.45 | 54197.42 | 54023.73 | 54175.6 | 54080.5 | 54154.3 | 54184.31 | 54057.89 | 54295.11 |
| 704.0311 | 54273 | 54007.35 | 53990.91 | 54167.06 | 54192 | 54173.45 | 54186.08 | 54075 | 54119.8 | 54084.75 | 54089 | 54103.31 | 54146 | 54202.44 |
| 704.1631 | 54302.3 | 54029.24 | 54071.73 | 54228.18 | 54063.75 | 54086.18 | 54129.75 | 54158.45 | 54139.13 | 54098.25 | 54137.4 | 54153.54 | 54318 | 54217.22 |
| 704.295 | 54261.6 | 54009.47 | 54044.91 | 54114.76 | 54061.5 | 54171.45 | 54079.75 | 54169.64 | 54146 | 54168.13 | 54085.5 | 54108.08 | 54183 | 54245 |
| 704.4271 | 54246 | 54031.41 | 54032.36 | 54111.82 | 54019.25 | 54156.64 | 54138.33 | 54158.36 | 54039.93 | 54236.25 | 54060.1 | 54044.31 | 54186.44 | 54158.33 |
| 704.5591 | 54142.7 | 54057.47 | 54005.18 | 54211.94 | 54013.88 | 54243.36 | 54116.17 | 54092.45 | 54075.73 | 54167 | 54109.2 | 54100.85 | 54174.11 | 54127.22 |
| 704.691 | 54147.1 | 54048.82 | 54085.27 | 54271.76 | 54105.88 | 54075.36 | 54173.42 | 54217.55 | 54062.2 | 54146.75 | 54089.6 | 54172.23 | 54058.11 | 54069.11 |
| 704.8231 | 54171.7 | 54108.65 | 53974.36 | 54182.65 | 53979.5 | 54052.09 | 54100.17 | 54177.64 | 54096.4 | 54062 | 54125.5 | 54141 | 54015.44 | 54163.89 |
| 704.9551 | 54132.3 | 54069.18 | 54037.55 | 54114.47 | 54053.38 | 54077.73 | 54069.83 | 54170.45 | 54070.47 | 54012.88 | 54190.2 | 54106.54 | 53977.89 | 54128.11 |
| 705.0871 | 54067.7 | 54119.47 | 54138.73 | 54147.06 | 54066.5 | 54144.73 | 54115.75 | 54198.55 | 54031.6 | 54146.5 | 54077 | 54165.85 | 54036.33 | 54077.56 |
| 705.2191 | 54007 | 54112 | 54111.82 | 54178.65 | 54208.88 | 54089.82 | 54054.92 | 54169.73 | 54097.07 | 54217.75 | 54152.4 | 54113.38 | 54081 | 54152.67 |
| 705.3511 | 54058.8 | 54069 | 54040.36 | 54156 | 54023.75 | 54043 | 54097.08 | 54204.73 | 54064 | 54124.88 | 54150.1 | 54151.23 | 54090.67 | 54286.22 |
| 705.4831 | 54110.1 | 54015.35 | 54123.18 | 54187.82 | 54059.88 | 54094.09 | 54055.42 | 54363.73 | 54066.27 | 54078.63 | 54116.2 | 54200.46 | 54088.89 | 54218.78 |
| 705.6151 | 54201.6 | 54074.65 | 54089.73 | 54117.41 | 54045.25 | 54084.45 | 54097.08 | 54372.91 | 54068.13 | 54131.25 | 54210.2 | 54105.08 | 54117.44 | 54228.56 |
| 705.7471 | 54271.8 | 54105.94 | 54080.45 | 54167.59 | 54049.75 | 54018.36 | 54091.5 | 54202.64 | 53997.33 | 53992.63 | 54169.8 | 54100.08 | 54122.33 | 54255.56 |
| 705.8791 | 54156.4 | 54053.24 | 54006.55 | 54211.76 | 54194.13 | 54069.18 | 54169.75 | 54162.91 | 54042.6 | 54052.63 | 54193.3 | 54077.54 | 54044.44 | 54154.22 |
| 706.0111 | 54052.9 | 54101.76 | 53980.64 | 54176.88 | 54179.38 | 54067.73 | 54184.17 | 54184.91 | 54106.2 | 54120.25 | 54242.9 | 54087.15 | 54115.22 | 54127.22 |
| 706.1431 | 54042.7 | 54094.59 | 53981 | 54200 | 54173.63 | 54113.18 | 54116.08 | 54144 | 54137.53 | 54124.5 | 54125.3 | 54127.62 | 54222.22 | 54137.56 |
| 706.2751 | 54034.2 | 54129.35 | 54032.18 | 54182.82 | 54162.75 | 54139.45 | 54212.17 | 54049.73 | 54145.07 | 54030.75 | 54185.4 | 54207.38 | 54059.89 | 54092.89 |
| 706.4071 | 54072.1 | 54013.59 | 54043 | 54120.76 | 54189.25 | 54169 | 54073.42 | 54210.18 | 54147.2 | 54038.88 | 54213.4 | 54093.08 | 54061 | 54192.22 |
| 706.5391 | 54182 | 54054.59 | 54071.09 | 54106.41 | 54078.5 | 54071.27 | 54141.25 | 54226.73 | 54051.33 | 54058.13 | 54299.9 | 54113.31 | 54043.11 | 54117.89 |
| 706.6711 | 54146.8 | 54045.71 | 54046.55 | 54152 | 54032.63 | 54045.27 | 54198.33 | 54122.27 | 54043.67 | 54161.63 | 54177.4 | 54105.08 | 54014.67 | 54185.78 |
| 706.8031 | 54156.9 | 54060.41 | 54059.55 | 54129.41 | 54084.88 | 54115.73 | 54199.08 | 54130.73 | 54096.47 | 54055.25 | 54062.1 | 54074.31 | 54079.11 | 54279.33 |
| 706.9351 | 54119.8 | 54126.59 | 53999.91 | 54181.24 | 54157.75 | 54198.91 | 54209.75 | 54235.64 | 54028.6 | 54126.13 | 54146.3 | 54115.38 | 54107 | 54123.44 |
| 707.0671 | 54076.7 | 54098.18 | 54041.73 | 54233.82 | 54074.63 | 54324.55 | 54167.33 | 54308.91 | 54039 | 54099.38 | 54069.7 | 54163.54 | 54103.56 | 54000.89 |
| 707.1991 | 54138.9 | 54069.06 | 54017 | 54146.18 | 54000.88 | 54263.36 | 54155.5 | 54175 | 54059.93 | 54064.63 | 54082.6 | 54187.69 | 54077.44 | 54041.11 |
| 707.3311 | 54211.2 | 54090.29 | 54019.91 | 54194.65 | 53990.63 | 54127.18 | 54166.17 | 54160.36 | 54032.47 | 54064.13 | 54178.1 | 54190.92 | 54130.78 | 54106.44 |
| 707.4631 | 54202.3 | 54076.76 | 54045.91 | 54216.41 | 54081 | 54201.18 | 54088.17 | 54089.73 | 54046.07 | 54137.38 | 54129.8 | 54116.77 | 54064 | 54138.89 |
| 707.5951 | 54165.4 | 54100.94 | 53971.73 | 54160.47 | 54123.63 | 54292.36 | 54117.17 | 54047.73 | 54147.73 | 54205.88 | 54107.2 | 54183.92 | 54124.89 | 54058.22 |
| 707.7271 | 54184.7 | 54159.24 | 53966.55 | 54127.53 | 54020.63 | 54227 | 54073 | 54124.27 | 54138.33 | 54123.25 | 54039.3 | 54212.46 | 54138.44 | 54169.67 |
| 707.8591 | 54079.8 | 54060.41 | 54028.91 | 54164.41 | 54053.13 | 54177.27 | 54124.33 | 54197.82 | 54138.13 | 54215.25 | 54056 | 54111.54 | 54060.22 | 54179.33 |
| 707.9911 | 54075.8 | 54040 | 53956.09 | 54137.41 | 54167.63 | 54187 | 54198 | 54134.73 | 54057.73 | 54075.88 | 54044.8 | 54177.92 | 54097.56 | 54059.22 |
| 708.1231 | 54140.9 | 54060.59 | 53938.91 | 54148.76 | 54137 | 54209.45 | 54221.25 | 54046.18 | 54202.4 | 54045 | 54122.1 | 54138.85 | 54140.44 | 54119.89 |
| 708.2551 | 54066.4 | 54077.47 | 53991.45 | 54158.71 | 54089.75 | 54135.45 | 54247.67 | 53986.55 | 54110.07 | 54192.75 | 54171.1 | 54121.62 | 54160.33 | 54135 |
| 708.3872 | 54094.5 | 54071.59 | 54067.09 | 54134.12 | 54064.88 | 54023.55 | 54277.17 | 54114.82 | 54047.73 | 54200.75 | 54205.4 | 54091.46 | 54157.56 | 54218.89 |
| 708.5191 | 54035.2 | 54108.18 | 54054.55 | 54169.53 | 54094.13 | 54137.09 | 54404.17 | 54136.09 | 54099.27 | 54303.75 | 54157.8 | 54073.23 | 54177.89 | 54130.44 |
| 708.6511 | 54051.7 | 54071.76 | 54078.82 | 54120.47 | 54084.75 | 54240.36 | 54180.67 | 54139.36 | 54099 | 54323.25 | 54279.2 | 54177.69 | 54136.33 | 54130.78 |
| 708.7831 | 54026.9 | 54004.35 | 54073.36 | 54144.29 | 54058.63 | 54180.45 | 54044.17 | 54131 | 54216.4 | 54159.63 | 54160.9 | 54171.92 | 54120.44 | 54159.11 |
| 708.9151 | 54095.9 | 54023.41 | 54078.36 | 54222.94 | 54110.88 | 54168.82 | 54097.83 | 54144.18 | 54107.33 | 54145.88 | 54068.4 | 54019.46 | 54156.89 | 53988.11 |
| 709.0471 | 54315 | 54108.88 | 54031.73 | 54154.29 | 54062.63 | 54153 | 54107.5 | 54024.91 | 54064.33 | 53976.38 | 54060.1 | 54028.23 | 54294 | 53995.11 |
| 709.1791 | 54134.7 | 54138.18 | 54011.27 | 54164.65 | 54016.38 | 54090.64 | 54058.92 | 54160.82 | 54082.8 | 54038.63 | 54053.6 | 54109.92 | 54103 | 54112.44 |
| 709.3112 | 54089.7 | 54164.18 | 54109.64 | 54191.65 | 53973.5 | 54101.45 | 54179.75 | 54216.73 | 54138.4 | 54250.25 | 54062.8 | 54189.85 | 54182.33 | 54249.44 |
| 709.4431 | 54073.3 | 54171.53 | 54193.09 | 54151.47 | 53993.63 | 54150.09 | 54126.5 | 54126.73 | 54199 | 54047 | 54204.5 | 54186.08 | 54099.33 | 54228.56 |
| 709.5751 | 54161.1 | 54136.06 | 54150.36 | 54167.12 | 54076.38 | 54173 | 54163.25 | 54147.73 | 54262.27 | 54030 | 54112.9 | 54151.15 | 54190.78 | 54149.67 |
| 709.7072 | 54125.9 | 54085.35 | 54181.27 | 54236.53 | 54237.5 | 54146.73 | 54130.75 | 54099.73 | 54201.13 | 54004.13 | 54179.9 | 54059.23 | 54154.67 | 54119.22 |
| 709.8392 | 54277.3 | 54156.41 | 54180.64 | 54365.24 | 54158.13 | 54129.09 | 54021.75 | 54098.09 | 54265.73 | 53943.5 | 54130.1 | 53998.69 | 54064.78 | 54056 |
| 709.9711 | 54200.7 | 54125.06 | 54178.64 | 54212.47 | 54267.38 | 54155.45 | 54058.92 | 54121.82 | 54188 | 54014.75 | 54052.6 | 53972 | 54084.44 | 54091.78 |
| 710.1032 | 54212.2 | 54186.35 | 54201.91 | 54189.29 | 54159 | 54122.45 | 54098.83 | 54028.73 | 54091.13 | 54124.5 | 54211.1 | 53998.69 | 54071.67 | 54007.33 |
| 710.2352 | 54213.2 | 54076 | 54149.91 | 54214.29 | 54172.38 | 54092.73 | 53994.92 | 53974.91 | 54112.73 | 54138.75 | 54255.7 | 54058.46 | 54200.33 | 54115 |
| 710.3671 | 54130.8 | 54093.65 | 54056.91 | 54179.76 | 54109.88 | 54160.64 | 54194.42 | 53998 | 54180 | 54161.5 | 54068.4 | 54011.31 | 54208.89 | 54125.56 |
| 710.4992 | 54131.1 | 54093.59 | 54096 | 54223 | 54076 | 54194.18 | 54136.67 | 53995.36 | 54197.13 | 54098.5 | 54029 | 54041.31 | 54134.11 | 54164.67 |
| 710.6312 | 54104.2 | 54068.12 | 54124 | 54229.82 | 54016.25 | 54259.91 | 54104.83 | 54103.09 | 54223.27 | 54056.5 | 54152.5 | 54110.23 | 54075.22 | 54161.56 |
| 710.7632 | 54064.5 | 54079.76 | 54081.73 | 54221.59 | 53987.88 | 54188.09 | 54084.25 | 54107.09 | 54209.07 | 54174.75 | 54235.9 | 54240.54 | 54115.67 | 54173.44 |
| 710.8951 | 54038.1 | 54118.65 | 54020.64 | 54145.24 | 53991.38 | 54126.45 | 54116.25 | 54100.09 | 54118.53 | 54241.25 | 54206.5 | 54096.54 | 54187.33 | 54177.89 |
| 711.0272 | 54029.7 | 54110.12 | 54106.09 | 54135.12 | 54122.75 | 54137.64 | 54161.75 | 54032 | 54193.27 | 54104.25 | 54059.3 | 54035.69 | 54122.11 | 54127.78 |
| 711.1592 | 54109.4 | 54145.88 | 54023.91 | 54193 | 54065.75 | 54086.36 | 53992.25 | 54089.91 | 54057.73 | 54022 | 54119.4 | 54036.85 | 54097.44 | 54110.78 |
| 711.2911 | 54109.5 | 54090.53 | 54084.73 | 54098.12 | 54160.5 | 54047.45 | 54064.92 | 54190.18 | 54070.33 | 54059.25 | 54125.3 | 54025.38 | 54099.33 | 54111.89 |
| 711.4232 | 54118.3 | 54074.53 | 54064 | 54077.53 | 54266 | 54198.45 | 54025.5 | 54180.09 | 54158.2 | 54074.25 | 54097.1 | 53992.31 | 54197 | 54226.56 |
| 711.5552 | 54066.7 | 54090.47 | 54153.55 | 54034.53 | 54203.38 | 54216.64 | 54161.25 | 54076.73 | 54164.87 | 54106.88 | 54008.8 | 54088.54 | 54120.44 | 54166.44 |
| 711.6872 | 54101.3 | 54062.53 | 54101.27 | 54067.65 | 54255.25 | 54133.18 | 54124.25 | 54093.18 | 54104 | 54130.25 | 54066.3 | 54075.62 | 54194.33 | 54173.33 |
| 711.8192 | 54135.4 | 54125.12 | 54035.09 | 54049.59 | 54290.38 | 54250.27 | 54069.92 | 54068.09 | 54147.8 | 54275.13 | 54049.3 | 54052 | 54232.89 | 54211.33 |
| 711.9512 | 54152.4 | 54080.82 | 54035.55 | 54034.59 | 54202.88 | 54033.36 | 54163.25 | 54117.91 | 54246.4 | 54194.75 | 53993 | 54190.23 | 54198.11 | 54122.33 |
| 712.0832 | 54164.5 | 54083.29 | 54068.27 | 54094.24 | 54179 | 54099.64 | 54114.58 | 54065.09 | 54169.93 | 54012.5 | 54135.5 | 54170 | 54209.44 | 54097.33 |
| 712.2152 | 54186.4 | 54071.06 | 54014 | 54138.06 | 54041.38 | 54204.82 | 54082.83 | 54104.64 | 54506.53 | 54078 | 54237.1 | 54094.77 | 54136.56 | 54117.44 |
| 712.3472 | 54060.4 | 54086.59 | 53974.91 | 54169.94 | 53990 | 54156.27 | 54078 | 54060.91 | 54394.93 | 54113.63 | 54302.4 | 54179.62 | 54278 | 54046.67 |
| 712.4792 | 54148.3 | 54086.65 | 54065.82 | 54133.18 | 54112.38 | 54094.09 | 54230.25 | 53982.45 | 54226.67 | 54058.5 | 54183.7 | 54196.92 | 54185.11 | 54143 |
| 712.6112 | 54041.4 | 54103.35 | 54118.91 | 54163.06 | 54172.75 | 54102.73 | 54150.5 | 53975.82 | 54177.73 | 54001.5 | 54139.1 | 54106.85 | 54233 | 54138 |
| 712.7432 | 54098.7 | 54035.24 | 54038 | 54117.47 | 54027 | 54122.64 | 54196.67 | 54089.73 | 54096.07 | 54006.75 | 54153.4 | 54063.62 | 54188.33 | 54210.89 |
| 712.8752 | 54042.7 | 54027.82 | 54096.64 | 54189.59 | 54091.63 | 54116.64 | 54129.33 | 54077.27 | 54079.73 | 54025.38 | 54093.4 | 54001.92 | 54187.22 | 54232.11 |
| 713.0072 | 54096.3 | 54058.53 | 54093.73 | 54230.82 | 54134.88 | 54122.45 | 54028.83 | 54033.45 | 54063.53 | 54159.88 | 54048.6 | 54032.77 | 54072.56 | 54037 |
| 713.1392 | 54163.6 | 54070.18 | 54038.73 | 54179.65 | 54113.13 | 54184.55 | 54078.75 | 54117.55 | 54111.67 | 54116.5 | 54193.9 | 54106.08 | 54109.56 | 54160.11 |
| 713.2712 | 54186.3 | 54137.76 | 54019.45 | 54148.24 | 54054.75 | 54030.55 | 54134.67 | 54163.55 | 54092.67 | 54118 | 54166.8 | 54247.23 | 54092.67 | 54150.44 |
| 713.4032 | 54138.1 | 54133.53 | 54037 | 54222.94 | 54003.63 | 54051.55 | 54069.25 | 54108 | 54226.73 | 54098.13 | 54145.1 | 54046.23 | 54193.78 | 54133.22 |
| 713.5352 | 54090.7 | 54097.76 | 54096.91 | 54237.53 | 53983.38 | 54196.45 | 54122.83 | 54044 | 54209.53 | 54079.75 | 54164.8 | 54057.31 | 54160.33 | 54120.78 |
| 713.6672 | 54059.6 | 54057.47 | 54161.45 | 54294.65 | 54016.88 | 54117.18 | 54240.75 | 54010.73 | 54143.6 | 54042.88 | 54138.2 | 54019.62 | 54287.56 | 54093.67 |
| 713.7992 | 54128.4 | 54110.94 | 54120.36 | 54159.94 | 54003.75 | 54078.36 | 54299.83 | 53952.64 | 54142.87 | 54121.63 | 54218.6 | 54042.15 | 54226.67 | 54202.44 |
| 713.9312 | 54099.4 | 54090.76 | 54148.82 | 54178 | 54035.38 | 53959 | 54294.17 | 53982.27 | 54071 | 54246.75 | 54109.9 | 54113.69 | 54170.33 | 54174.22 |
| 714.0632 | 54112.8 | 54158.12 | 54055.73 | 54136.12 | 53950.75 | 54071.36 | 54145.83 | 54051.82 | 54114.27 | 54144.13 | 54140 | 54058.08 | 54182.22 | 54106.78 |
| 714.1952 | 54097.9 | 53996.53 | 54135.73 | 54180.53 | 53967.63 | 54091.64 | 54156 | 54049.73 | 54152.2 | 54170 | 54081.5 | 54076.15 | 54100.78 | 54204.11 |
| 714.3272 | 54061.8 | 54003.76 | 54084.64 | 54192.76 | 54001.5 | 53987.55 | 54207.25 | 54067.09 | 54117.07 | 54061.88 | 54127.5 | 54119.31 | 54050.11 | 54122.67 |
| 714.4592 | 54063 | 54136.06 | 54124.64 | 54240.65 | 54024.38 | 54068.82 | 54228 | 54130.36 | 54111.13 | 54194 | 54085.3 | 53973.92 | 54133 | 54060.33 |
| 714.5912 | 54044.3 | 54067.65 | 54154.27 | 54207.06 | 54102.38 | 54096.18 | 54219.67 | 54116.55 | 54065.27 | 54169.88 | 54262.2 | 54002 | 54087.67 | 54118.33 |
| 714.7232 | 54109 | 54069.18 | 54141.73 | 54286.76 | 54139.75 | 54219.45 | 54167.33 | 54028.64 | 54190.27 | 54160.88 | 54269.1 | 54090.77 | 54095.78 | 54078.67 |
| 714.8552 | 54315.3 | 54168.06 | 54347.64 | 54238.29 | 54051.38 | 54213.45 | 54172.92 | 54113.64 | 54215.07 | 54087.13 | 54183 | 54017 | 54073.33 | 54145.67 |
| 714.9872 | 54158.5 | 54082.12 | 54149.18 | 54176.06 | 54118.38 | 54222.18 | 54115.58 | 54112.91 | 54088.27 | 54056.13 | 54214.4 | 54122.38 | 54097 | 54180.11 |
| 715.1192 | 54052.5 | 54131.94 | 54052.73 | 54180.18 | 54203.88 | 54038.27 | 54142.67 | 54118 | 54137.8 | 54084.13 | 54142.5 | 54096.92 | 54231.67 | 54139.11 |
| 715.2512 | 54202.7 | 54130.24 | 54076.64 | 54214.06 | 54179.5 | 54108.36 | 54108.17 | 54126.09 | 54161.53 | 54264.63 | 54123 | 54108.54 | 54305.78 | 54182.11 |
| 715.3832 | 54180.1 | 54196.24 | 54103.82 | 54146.53 | 54238.38 | 54131.91 | 54230.58 | 54098.64 | 54051.33 | 54232.13 | 54092.2 | 53992.46 | 54118.67 | 54136.33 |
| 715.5152 | 54143.8 | 54115.59 | 54164.73 | 54172.29 | 54229.75 | 54142.82 | 54076.42 | 54155.45 | 54089.4 | 54266.25 | 54237.7 | 54070.23 | 54066.56 | 54165.11 |
| 715.6472 | 54123.7 | 54066.82 | 54187.18 | 54226.82 | 54106.25 | 54175.36 | 54210.75 | 54201.91 | 54100.27 | 54174.5 | 54104.2 | 54063.54 | 54031.33 | 54072.89 |
| 715.7792 | 54034.4 | 54049.65 | 54178 | 54338.24 | 54129.38 | 54155.36 | 54204.58 | 54234.91 | 54138.33 | 54127.38 | 54154.3 | 53980.62 | 54047.44 | 54200.22 |
| 715.9113 | 54010.5 | 54077.06 | 54185.91 | 54296.12 | 54007.38 | 54094.82 | 54165 | 54083.45 | 54098.4 | 54196.25 | 54073.8 | 54001.69 | 54106.56 | 54214.22 |
| 716.0432 | 54059 | 54134.71 | 54169.18 | 54234.59 | 54063.38 | 54092.45 | 54038.33 | 54238 | 54138.67 | 54318.38 | 54109.5 | 54067.38 | 54137.44 | 54330 |
| 716.1752 | 54173.5 | 54043.88 | 54116.73 | 54233.76 | 54222.38 | 54185.09 | 54123.08 | 54115.45 | 54165.8 | 54225.25 | 54133.2 | 54231.77 | 54137.78 | 54307.78 |
| 716.3073 | 54190 | 54088.29 | 54187.64 | 54226.18 | 54162.88 | 53981.45 | 54099.17 | 54079 | 54140.87 | 54227.13 | 54154.3 | 54221 | 54105.22 | 54178 |
| 716.4393 | 54139.1 | 53992 | 54195.27 | 54172.76 | 54124.38 | 54043.36 | 54075.33 | 54089.64 | 54091.8 | 54294.63 | 54309.4 | 54080.38 | 54173.78 | 54084.33 |
| 716.5712 | 54067 | 54056.88 | 54197.36 | 54097.71 | 54127.63 | 54031.73 | 54097.83 | 54075 | 54097.07 | 54218.5 | 54095.6 | 54089.54 | 54193.33 | 54118.33 |
| 716.7033 | 53977.9 | 54033.88 | 54227.36 | 54113.18 | 54152.38 | 54102.73 | 54155.5 | 54057.18 | 54167 | 54172.25 | 54038.9 | 54152 | 54055.44 | 54131 |
| 716.8353 | 54139.5 | 54053.47 | 54182.09 | 54164.53 | 54229.88 | 54256.55 | 54142.42 | 54112 | 54223 | 54131.5 | 54115.8 | 54212.54 | 53983.22 | 54136.11 |
| 716.9672 | 54230.8 | 54177.29 | 54105.18 | 54147.53 | 54286.5 | 54126.82 | 54192.5 | 54000 | 54256.4 | 54074.75 | 54088.8 | 54114.31 | 54036.22 | 54124.44 |
| 717.0992 | 54140.6 | 54259.94 | 54135.82 | 54186.88 | 54147.75 | 54238 | 54252.92 | 54004 | 54219.8 | 54083.38 | 54137 | 54004.46 | 54106.22 | 54173.44 |
| 717.2313 | 54174.6 | 54204.94 | 54117.45 | 54158.88 | 54103.13 | 54110.36 | 54250.58 | 54050.27 | 54152.53 | 54025.25 | 54099.7 | 54095.46 | 54081.11 | 54082.56 |
| 717.3633 | 54163.3 | 54173.06 | 54151.18 | 54177.76 | 54064.38 | 54090.64 | 54154.25 | 54081.27 | 54120.4 | 54091.63 | 54266.4 | 54148.31 | 54222.44 | 54143.44 |
| 717.4952 | 54060.9 | 54034.12 | 54110.09 | 54177.35 | 54055.38 | 54126.64 | 54300.33 | 54158.09 | 54169.47 | 54197 | 54156.6 | 54112.92 | 54132.89 | 54158.11 |
| 717.6273 | 54116.6 | 54045.76 | 54171.45 | 54180.53 | 54061.13 | 54033 | 54272.17 | 54203.73 | 54061.73 | 54093 | 54099 | 54194.38 | 54130 | 54147 |
| 717.7593 | 54188.6 | 54059.53 | 54129.82 | 54117.94 | 54018.5 | 54030.36 | 54215.5 | 54139.09 | 54104.47 | 54143.75 | 54124.4 | 54239.15 | 54114.78 | 54125.33 |
| 717.8912 | 54035.4 | 54089.76 | 54123.91 | 54130.24 | 54014.13 | 54199 | 54123 | 54216.91 | 54105.67 | 54122.5 | 54116.8 | 54097.46 | 54129.78 | 54171.56 |
| 718.0233 | 54046.2 | 54153.59 | 54089.55 | 54141.59 | 54117.63 | 54119.27 | 54180.08 | 54155.91 | 54107.8 | 54035.63 | 54045.3 | 54223.08 | 54023.89 | 54128.89 |
| 718.1553 | 54179.1 | 54103.18 | 54033.09 | 54135.53 | 54165.5 | 54095.73 | 54099.08 | 54186.55 | 54125.8 | 54047.5 | 54087.4 | 53969.08 | 54045.44 | 54286 |
| 718.2873 | 54131.3 | 54201.71 | 53959.55 | 54153.41 | 54050.5 | 54140 | 54130.92 | 54155.55 | 54123.67 | 54128.38 | 54083.8 | 54012.62 | 54201.78 | 54335.56 |
| 718.4193 | 54173.1 | 54172.12 | 54069.91 | 54160.12 | 54072.75 | 54176.36 | 54251.33 | 54248.91 | 54186 | 54117.88 | 54040.6 | 54067.62 | 54128 | 54270.78 |
| 718.5513 | 54229.3 | 54131.06 | 54090.45 | 54146.94 | 54145.13 | 54113.18 | 54147.75 | 54269.82 | 54229.67 | 54166.75 | 54086.8 | 54054.54 | 54160 | 54219 |
| 718.6833 | 54244.9 | 54142.76 | 54167.91 | 54198.71 | 54010.63 | 54059.45 | 54243.08 | 54245.09 | 54191 | 54037.5 | 54122.2 | 54096.85 | 54099 | 54198.33 |
| 718.8153 | 54239.2 | 54082.24 | 54029.18 | 54173.29 | 54034.38 | 54086.09 | 54197.33 | 54123.64 | 54251.33 | 54165.88 | 54115.9 | 54167.77 | 54032.67 | 54086.11 |
| 718.9473 | 54116.2 | 54131.65 | 54031.55 | 54168.41 | 54061.25 | 54046.55 | 54285.25 | 54082.45 | 54102.6 | 54197 | 54011.4 | 54029 | 54060.56 | 54081.11 |
| 719.0793 | 54027.4 | 54162.59 | 54092 | 54115.35 | 54160.88 | 54074.36 | 54324 | 54061.55 | 54118.4 | 54094.13 | 54054.6 | 54090.77 | 54042.89 | 54090.22 |
| 719.2113 | 54034.9 | 54109.41 | 54118.45 | 54126.29 | 54195.13 | 54072.36 | 54156.67 | 53993.55 | 54088 | 54085.5 | 54137.9 | 54088.62 | 54076.67 | 54090.89 |
| 719.3433 | 54115.3 | 54069.94 | 54214.45 | 54175.24 | 54172 | 54141.64 | 54139.25 | 53997.36 | 54203.87 | 54046 | 54166 | 54101.85 | 54219.22 | 54068.44 |
| 719.4753 | 54244.9 | 54025.71 | 54174.55 | 54236 | 54167.38 | 54196 | 54099.25 | 53983 | 54180.07 | 54089.25 | 54145.9 | 54058.85 | 54197.22 | 54128.67 |
| 719.6073 | 54232.7 | 54109.82 | 54032.91 | 54196.76 | 54189.13 | 54153.82 | 54051.92 | 54022.82 | 54114.8 | 54112.13 | 54078.5 | 54098.46 | 54050.78 | 54204.11 |
| 719.7393 | 54226.5 | 54104.12 | 54021.36 | 54152.06 | 54103.88 | 54210.73 | 54047.08 | 54131.82 | 54176.67 | 54111.75 | 54150.1 | 54092.92 | 54121.89 | 54124.33 |
| 719.8713 | 54172.6 | 54047.82 | 54105.91 | 54117.47 | 54220.38 | 54095.18 | 54174.58 | 54028.45 | 54089.87 | 54027.88 | 54252.5 | 54134.77 | 54096.78 | 54082.11 |
| 720.0033 | 54178.4 | 54066.35 | 54098.64 | 54135.47 | 54246.38 | 54108.36 | 54170.5 | 53980.64 | 54165.33 | 54108.38 | 54341.4 | 54248.31 | 54269 | 54121.22 |
| 720.1353 | 54117.7 | 54053.71 | 54042.82 | 54224.59 | 54298.88 | 54073.36 | 54179.92 | 53978.18 | 54258.8 | 54137.38 | 54222 | 54138.77 | 54096.44 | 54135.44 |
| 720.2673 | 54175.6 | 54088.59 | 54064.18 | 54191.82 | 54217 | 54081.27 | 54106.75 | 53973.64 | 54192.6 | 54126.25 | 54053.8 | 54140.85 | 54134.44 | 54049.78 |
| 720.3993 | 54215 | 54148 | 54167.91 | 54139.18 | 54097.75 | 54026.82 | 54064.75 | 54002.27 | 54134.8 | 54071.63 | 53978.1 | 54201.77 | 54071.89 | 54069.11 |
| 720.5313 | 54045.4 | 54158.82 | 54201.64 | 54179.29 | 54089.75 | 54032.64 | 54041.42 | 54036.55 | 54110.47 | 54046.75 | 54077 | 54230.54 | 54179.11 | 54191.33 |
| 720.6633 | 54124.7 | 54129.29 | 54219.91 | 54245.71 | 54179.88 | 54127.91 | 54102.83 | 53958 | 54150.93 | 54254.25 | 54078.7 | 54176.38 | 54145 | 54265.33 |
[truncated: 96,430 more chars]
